# Conservation and divergence of regulatory architecture in nitrate-responsive plant gene circuits

**DOI:** 10.1093/plcell/koaf124

**Published:** 2025-05-22

**Authors:** Chao Bian, Gozde S Demirer, M Tufan Oz, Yao-Min Cai, Sam Witham, G Alex Mason, Zhengao Di, Florian Deligne, Ping Zhang, Rachel Shen, Allison Gaudinier, Siobhan M Brady, Nicola J Patron

**Affiliations:** Department of Plant Biology and Genome Center, University of California, Davis, Davis CA 95616, USA; Frontiers Science Center for Molecular Design Breeding (MOE), State Key Laboratory of Maize Bio-Breeding, National Maize Improvement Center, Department of Plant Genetics and Breeding, China Agricultural University, Beijing 100193, P. R. China; Department of Plant Biology and Genome Center, University of California, Davis, Davis CA 95616, USA; Division of Chemistry and Chemical Engineering, California Institute of Technology, Pasadena, CA 91125, USA; Engineering Biology, Earlham Institute, Norwich Research Park, Norwich NR4 7UZ, UK; Engineering Biology, Earlham Institute, Norwich Research Park, Norwich NR4 7UZ, UK; Engineering Biology, Earlham Institute, Norwich Research Park, Norwich NR4 7UZ, UK; Department of Plant Biology and Genome Center, University of California, Davis, Davis CA 95616, USA; Engineering Biology, Earlham Institute, Norwich Research Park, Norwich NR4 7UZ, UK; Department of Plant Biology and Genome Center, University of California, Davis, Davis CA 95616, USA; Department of Plant Biology and Genome Center, University of California, Davis, Davis CA 95616, USA; College of Horticulture, Shanxi Agricultural University, Taigu 03081, P.R. China; Engineering Biology, Earlham Institute, Norwich Research Park, Norwich NR4 7UZ, UK; Department of Plant Biology and Genome Center, University of California, Davis, Davis CA 95616, USA; Department of Plant and Microbial Biology, University of California, Berkeley, Berkeley, CA 94720, USA; Department of Plant Biology and Genome Center, University of California, Davis, Davis CA 95616, USA; Howard Hughes Medical Institute, University of California, Davis, Davis, CA 95616, USA; Engineering Biology, Earlham Institute, Norwich Research Park, Norwich NR4 7UZ, UK; Department of Plant Sciences, University of Cambridge, Downing Street, Cambridge CB2 3EA, UK

## Abstract

Plant roots dynamically respond to nitrogen availability by executing a signaling and transcriptional cascade resulting in altered plant growth that is optimized for nutrient uptake. The NIN-LIKE PROTEIN 7 (NLP7) transcription factor senses nitrogen and, along with its paralog NLP6, partially coordinates transcriptional responses. While the post-translational regulation of NLP6 and NLP7 is well established, their upstream transcriptional regulation remains understudied in Arabidopsis (*Arabidopsis thaliana*) and other plant species. Here, we dissected a known sub-circuit upstream of NLP6 and NLP7 in Arabidopsis, which was predicted to contain multiple multi-node feedforward loops suggestive of an optimized design principle of nitrogen transcriptional regulation. This sub-circuit comprises AUXIN RESPONSE FACTOR 18 (ARF18), ARF9, DEHYDRATION-RESPONSIVE ELEMENT-BINDING PROTEIN 26 (DREB26), Arabidopsis NAC-DOMAIN CONTAINING PROTEIN 32 (ANAC032), NLP6 and NLP7 and their regulation of NITRITE REDUCTASE 1 (NIR1). Conservation and divergence of this circuit and its influence on nitrogen-dependent root system architecture were similarly assessed in tomato (*Solanum lycopersicum*). The specific binding sites of these factors within their respective promoters and their putative cis-regulatory architectures were identified. The direct or indirect nature of these interactions was validated *in planta.* The resulting models were genetically validated in varying concentrations of available nitrate by measuring the transcriptional output of the network revealing rewiring of nitrogen regulation across distinct plant lineages.

## Teaser

Gene circuits that control transcriptional regulation of nitrate-responses have similarities and differences, despite the equivalent effects of nitrogen on tomato and Arabidopsis root morphology.

IN A NUTSHELL Nitrogen (N) is essential for plant growth, but excessive fertilizer use causes environmental harm. Plants sense N availability through signaling pathways that regulate gene expression. Transcription factors (TFs), proteins that bind DNA to activate or repress genes, coordinate these responses. A key protein, NLP7, plays a central role in regulating N responses in the model plant Arabidopsis. While post-translational regulation of NLP7 and its closely related paralog, NLP6, is well established, its upstream transcriptional regulation remains understudied in Arabidopsis and other plant species. We investigated a predicted regulatory circuit upstream of NLP6 and NLP7 focusing on conserved and divergent mechanisms in the crop species tomato (*Solanum lycopersicum*).

**Question:** What are the regulatory genes and pathways controlling NLP6 and NLP7 transcription? And how are these mechanisms conserved or divergent between Arabidopsis and tomato?

**Findings:** Using Arabidopsis and tomato mutants, in vitro DNA-binding assays, and protoplast-based transcriptional reporters, we identified a six-TF sub-circuit in Arabidopsis (AtARF18, AtARF9, AtDREB26, AtANAC032, and AtNLP6/7), which includes a coherent feedforward loop. The circuit is sufficient to regulate the expression of *NITRITE REDUCTASE 1 (NIR1*), a direct target of NLP7, and the nitrogen-dependent transcriptional network. In tomato, orthologs of ARF18, ARF9, and NLP6/7 regulate nitrate-dependent root system architecture, but with key differences in their regulation, DREB26, and NLP7 regulate N-responsive genes, but key differences emerged: DREB26 represses NLP7 in tomato, unlike in Arabidopsis, and ANAC032 (a repressor in Arabidopsis) lacks a clear tomato ortholog. This regulatory model was validated using genetic perturbation in single- and higher-order mutants demonstrating the power of this approach.

**Next Steps:** Future work will test whether engineering conserved TF binding sites or rewiring species-specific interactions (e.g. SlDREB26 repression) can optimize nitrogen use efficiency in crops. Spatial and temporal dynamics of these networks in root cell types also require exploration.

**Bluesky:** @bradylabs.bsky.social @prof-gozde.bsky.social

## Introduction

Nitrogen (N) is essential for plant growth and basic metabolic processes. The supply of N through the energy-intensive Haber-Bosch process and fertilizer application has been critical to increasing crop yields but also has negative ecological and environmental consequences (Savci 2012; Sinha et al. 2017). Knowledge of the diverse regulatory events that crop species employ to respond and adapt to N deficiency can be used to breed plants with increased N-use efficiency and ultimately, to lessen reliance on externally applied N. In response to limiting N, a complex series of molecular events are initiated (Stitt 1999 ; Zhang and Forde 2000; Vidal et al. 2014) including rapid post-transcriptional, calcium- and phosphorylation-dependent signaling cascades that converge on transcriptional regulation of N transporters, assimilation enzymes, N signaling factors, carbon metabolism and hormone pathways (Wang et al. 2003; Krouk et al. 2010b; Marchive et al. 2013; Guan et al. 2014; Liu et al. 2017b; Contreras-López et al. 2022). Below-ground, the outcome of these responses in the root includes altered lateral root elongation to forage for N and concomitant adjustment of N metabolism. Above-ground, changes are manifested in less plant growth and a reduction in yield.

Plant responses to nitrate have best been studied in the model dicot Arabidopsis (*Arabidopsis thaliana*). Evidence indicates that regulation of nitrogen- and nitrogen-associated metabolism is both transcriptional and post-translational in nature. Amongst the TFs identified to coordinate transcriptional responses to nitrate (Zhang and Forde 1998; Gifford et al. 2008; Castaings et al. 2009; Alvarez et al. 2014; Guan et al. 2014; Vidal et al. 2014; Medici et al. 2015; Gaudinier et al. 2018), Arabidopsis NIN-LIKE PROTEIN 7 (AtNLP7), and its paralog, Arabidopsis NIN-LIKE PROTEIN 6 (AtNLP6) (Castaings et al. 2009) play a key role by modulating the expression of downstream target genes following Ca^2+^-sensor protein kinase (CPK)-induced phosphorylation (Liu et al. 2017b ). AtNLP7 also directly binds nitrate (Liu et al. 2022). While our understanding of the post-translational modes by which AtNLP6/7 regulates gene expression, their own transcriptional regulation is less understood but critical to our knowledge of this important factor.

**Figure 1.**
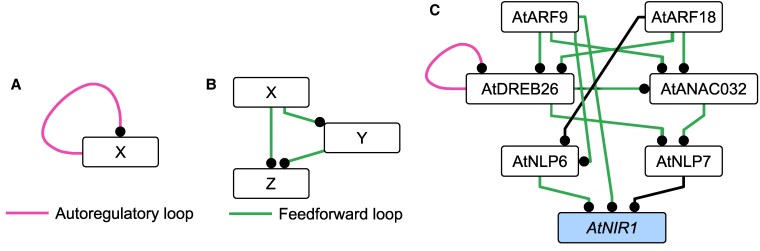
Nitrogen metabolism network sub-circuit in Arabidopsis that acts upstream of AtNLP6, AtNLP7, and AtNIR1 as determined by enhanced yeast one hybrid assays. **A)** Autoregulatory loop. **B)** Feedforward loop. **C)** This gene sub-circuit contains multiple interconnected feedforward loops (green edges) and an autoregulatory interaction (pink edges). *AtNIR1* is indicated in blue as the representative metabolic target gene.

TF networks consist of interconnected transcription factors and their target genes. These biological networks contain several types of regulatory interactions that are significantly over-represented including autoregulatory, feedforward, and negative feedback loops (Shen-Orr et al. 2002). Autoregulatory and feedforward loops are associated with assuring robustness of gene regulation, production of a toggle switch, a pulse of gene expression, and sustained activation of target genes (Fig. 1, A and B) (Lee et al. 2002; Shen-Orr et al. 2002; Atkinson et al. 2003; Mangan and Alon 2003; Milo et al. 2011; Alon 2018; Zong et al. 2023). We previously mapped a network of transcription factors that bind to promoters of nitrogen- and nitrogen-associated metabolic genes (Gaudinier et al. 2018 ). From these, a set of transcription factors, Arabidopsis AUXIN RESPONSE FACTOR 9 (*AtARF9),* Arabidopsis AUXIN RESPONSE FACTOR 18 (AtARF18), Arabidopsis NAC-DOMAIN CONTAINING PROTEIN 32 (AtANAC032) and Arabidopsis DEHYDRATION-RESPONSIVE ELEMENT-BINDING PROTEIN 26 (AtDREB26), form a circuit consisting of multiple loops that are predicted to regulate expression of *AtNLP6, AtNLP7* and *AtNIR1* (Gaudinier et al. 2018) (Fig. 1C). These include autoregulation (*AtDREB26*) and feedforward loops, where 3 genes interact in both a direct and indirect path (*AtARF9/AtDREB26/AtANAC032; AtARF18/AtDREB26/AtANAC032; AtARF9/AtNLP6/AtNIR1; AtDREB26/AtANAC032/AtNLP7*). Given these network motifs, we hypothesized that these genes are critical to transcriptional regulation of nitrogen metabolic gene expression.

Interpreting the influence of these transcription factors and their regulation of each other, as well as on the transcriptional regulation of N metabolism via *AtNIR1*, is non-trivial and depends on the sign of each regulatory interaction (activating or repressing) and the extent to which the gene is regulated by its upstream factors. Furthermore, to deploy the knowledge gained in Arabidopsis to the rational engineering of crop species, it is also necessary to obtain evidence of which aspects, if any, of the network are conserved. While NLP7 is conserved in some crop species (Cao et al. 2017; Wu et al. 2021), we lack evidence for other TFs and the interconnected manner by which they genetically determine nitrate-responses. To date, few molecular players associated with N signaling and metabolism in tomato *(Solanum lycopersicum)* have been identified (Liu et al. 2021b; Machado et al. 2023). Although there is a conservation of radial patterning between Arabidopsis and tomato, there are several cell types present in tomato that are not in Arabidopsis. For one of these, the exodermis, *SlNLP7* was predicted to be a central regulatory factor (Kajala et al. 2021). This difference may be reflected in a change in the underlying regulatory circuitry that controls responses to nitrate.

Transcriptional coordination of nitrogen metabolic gene expression occurs by multiple events, many of which have been mapped via genome-scale assays. These include chromatin accessibility assays (Sullivan et al. 2014; Maher et al. 2018), in vivo binding of individual TFs by chromatin immunopurification coupled with RNA sequencing (Marchive et al. 2013), and transcriptional regulation by RNA sequencing of mutants or by Transient Transformation System for Genome-Wide Transcription Factor Target Discovery (TARGET) using individual TFs (Bargmann et al. 2013; Para et al. 2014; Brooks et al. 2019; Alvarez et al. 2020). Genetic elaboration of these interconnected regulatory interactions is laborious. Systems biology approaches that include gene editing and quantitative protoplast-based assays enable the iterative testing of regulatory interactions between multiple transcription factors and their target regulatory regions in vivo. To date, AtNLP7 has been identified to bind to nitrate-responsive cis-elements in the promoters of nitrate-responsive target genes including *AtNIR1* (Konishi and Yanagisawa 2010). In an effort to better elucidate the genetic architecture of these genes predicted to act upstream of AtNLP6/7 and AtNIR1 with extensive feedforward loops (Fig. 1C), we systematically identified and validated specific binding motifs for TFs and studied their in vivo DNA binding and regulation (Gaudinier et al. 2018). Regions of open chromatin associated with each gene were mined for DNA-binding motifs, and the ability of TFs to bind to these targets was subsequently determined using an in vitro methodology (Cai et al. 2023 ). In vivo TF-binding and regulation are confirmed using a modification of the TARGET assay (Bargmann et al. 2013 ) and protoplast co-expression. In parallel, the conservation of this network in tomatoes was tested using phylogenomics coupled with the modified TARGET system. ARF18/ARF9 and NLP6/7 function at the top and bottom of this circuit, respectively, in both Arabidopsis and tomato. However, in between these factors, gene regulatory interactions differ between species. This network model was iteratively tested and validated using single, pairwise, and higher-order genetic perturbations of all factors in an in vivo assay in multiple concentrations of available nitrogen. Collectively, this systems-level approach illustrates the conservation and divergence of transcriptional regulation of N metabolism in 2 dicot species. Further, it identifies numerous sequences to target for future engineering of nitrate metabolism. Few plant regulatory networks have been genetically characterized to this extent. Our work provides a blueprint of how systems biology techniques can be used to inform synthetic biology approaches to crop engineering by predicting the outcomes of perturbations.

## Results

### In vitro characterization of candidate TF-binding sites supports interactions within a putative transcriptional network regulating Arabidopsis response to nitrate

In previous work, enhanced Yeast-1-Hybrid (eY1H) data was used to infer a putative transcriptional network that regulates the architecture of Arabidopsis root systems in response to N availability (Gaudinier et al. 2018) (Fig. 1). The yeast network contained many interconnected transcription factor-promoter interactions and was enriched for nitrate-responsive gene expression (Gaudinier et al. 2018). Genetic perturbation and mutant phenotyping of TFs predicted to be key in this network based on their connectivity properties showed that these genes regulate multiple aspects of root and shoot architecture. Understanding the cis-regulatory logic of these factors and how they work together in pathways is an important next step to understand how Arabidopsis coordinates the transcriptional response to available nitrogen. While eY1H is a powerful technique for identifying previously unknown interactions between TFs and target genes, it suffers from both false positive and false negative results (Reece-Hoyes et al. 2011 ). In addition, though eY1H can identify candidate target genes, it is unable to identify the cis-regulatory motifs responsible for TF-binding and cannot inform if interactions are likely to result in changes to the expression of target genes. This information is critical for understanding how regulatory networks control quantitative phenotypes, and for predicting the effects of perturbations. To address this, we first looked for evidence of TF-binding by identifying candidate binding sites in the regulatory regions of target genes.

**Figure 2.**
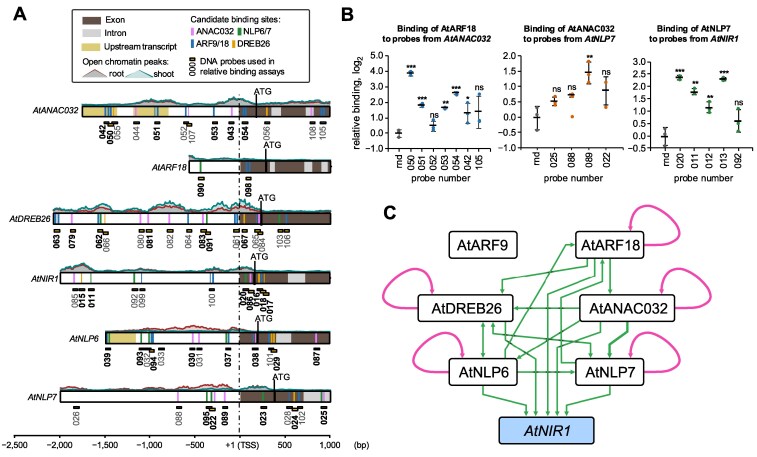
Transcription factor binding motifs and in vitro assays support and elaborate the sub-network **A)** Position weight matrices (PWMs) were used to identify candidate transcription factor-binding sites in the 5′ regions of *AtANAC032*, *AtARF18, AtDREB26, AtNIR1, AtNLP6,* and *AtNLP7* transcripts. Dark/light gray shading indicates exons/introns; upstream transcripts are shaded in sand; regions of open chromatin inferred by ATAC-seq are shown above the gene representations and outlined in green (shoot) and brown (roots); candidate transcription factor-binding sites are indicated by vertical bars ANAC032 (magenta), NLP6/7 (green), ARF9/18 (blue) and DREB26 (yellow). The *x*-axis indicates the position base pair position upstream of (negative) and downstream (positive) of the transcription start site (TSS). Binding of transcription factors (TF)s to short DNA probes containing candidate sites (numbered boxes) was evaluated using an in vitro assay; bold text indicates that significant binding of at least one transcription factor was detected (Supplementary Data S3). The *P*-value used to determine significance is >0.05. **B)** Representative in vitro binding assays showing interactions between AtARF18, AtANAC032, and AtNLP7 and probes from *AtANAC032, AtNLP7, and AtNIR1,* respectively. Error bars = mean and standard deviation; *n* = 3; *P*-values were calculated using an unpaired two-tailed Student's t-test of each sample to the random (rnd) control probe; *P < 0.05, **P < 0.01, ***P < 0.001; ns = not significant **C)** Protein-DNA interactions as determined by the in vitro assay. Edges that are part of a feedforward loop are indicated in green and autoregulatory interactions in pink. AtNIR1 is indicated in blue as the representative metabolic target gene.

To identify candidate TF-binding sites, we performed a systematic analysis of the open chromatin sites upstream and downstream of each gene in the regulatory circuit. Regions for analysis were restricted to 2 kilobase upstream of the start of transcription, or until the next protein-coding gene. In addition, we analyzed one kilobase downstream of the start of transcription. Peaks were mapped from publicly available ATAC-seq data (Potter et al. 2018)(Fig. 2A). Candidate binding sites for AtNLP7 and AtDREB26 were identified using the FIMO package within the MEME software suite (Bailey et al. 2015) using publicly available data to infer position weight matrices (PWMs) (O’Malley et al. 2016) (Supplementary Fig. S1). Equivalent DNA-binding data for AtNLP6, AtARF9, AtARF18, or AtANAC032 was unavailable and PWMs from a closely related TF, for which the DNA-binding domain was confirmed (Supplementary Fig. S2), were used to identify candidate sites (see Methods). This analysis identified candidate binding motifs that supported interactions within the putative eY1H regulatory circuit and identified the presence of further putative interactions (Fig. 2A).

To validate the potential for TF-DNA binding, the ability of each TF to bind to each candidate binding site was experimentally determined using a plate-base method for specific DNA sequences (Cai et al. 2023). To do this, we expressed recombinant proteins of AtARF18, AtANAC032, AtNLP6, AtANLP7, and AtDREB26 with a genetically encoded N-terminal 9xHIS tag and a C-terminal HiBit tag. We were unable to purify sufficient soluble protein of AtARF9. We then tested the ability of purified protein to bind to 80-bp probes containing candidate binding sites, relative to random DNA probes. Example data supporting 3 edges within the network are shown in (Fig. 2B). Data for all interactions is provided in Supplementary Fig. S3 and the sequences of all probes are provided in Supplementary Data Set 1.

From these data, we constructed an in vitro N network (Fig. 2C) and identified several previously known interactions including between AtNLP6/AtNLP7 and the Nitrogen-Responsive Element (NRE) present in the promoter region of *AtNIR1* (Konishi and Yanagisawa 2010), as well as AtNLP7 with itself (Alvarez et al. 2020). These data also support 4 additional edges previously identified by eY1H: AtARF18 to *AtANAC032* and *AtDREB26*; AtANAC032 to *AtNLP7*; and AtDREB26 to *AtNIR1*. In addition, 12 further potential interactions were identified: AtNLP6 and AtNLP7 to *AtARF18* and *AtDREB26*; AtANAC032 to *AtDREB26;* AtDREB26, AtARF18 and AtANAC032 to *AtNIR1*; and AtDREB26 and AtANAC032 to *AtNLP6.* Some of these interactions could not have been identified by the earlier eY1H assay, as the motifs occur beyond the borders of the region used for eY1H assays. Finally, 3 instances of potential autoregulation were identified for AtANAC032, AtARF18, AtDREB26, and AtNLP6. Interestingly, AtNLP6, AtNLP7, AtDREB26, and AtANAC032 were all observed to bind significantly to both *AtNLP6* and *AtNLP7*. While these data indicate the potential for protein-DNA interactions, in vitro binding does not provide evidence that these 28 TF-DNA interactions occur *in planta* or are regulatory in nature.

### Refining the network by protoplast expression assays to determine the regulatory consequences of TF-binding

To investigate if the TFs were able to physically bind to and modulate the expression of predicted targets in vivo, we used a variation of the Transient Transformation System for Genome-Wide Transcription Factor Target Discovery (TARGET) assay, in which glucocorticoid receptor (GR)-tagged TFs are expressed in protoplasts (Bargmann et al. 2013 ). This assay quantifies the expression of endogenous target genes following the treatment of cells with dexamethasone (DEX). Cycloheximide (CHX) in the presence of DEX determines if regulation is direct and regulatory. In TARGET, transcriptional changes were quantified using RNA-seq. However, in this variation, changes to the expression of specific target genes were quantified using qPCR. We additionally investigated predicted direct interactions using a ratiometric transactivation luciferase (LUC) assay in which each target promoter was fused to a nanoluciferase reporter (LucN) and coexpressed in Arabidopsis protoplasts with either a constitutively expressed TF or a constitutively expressed control protein, yellow fluorescent protein (YFP) (Supplementary Fig. S4).

**Figure 3.**
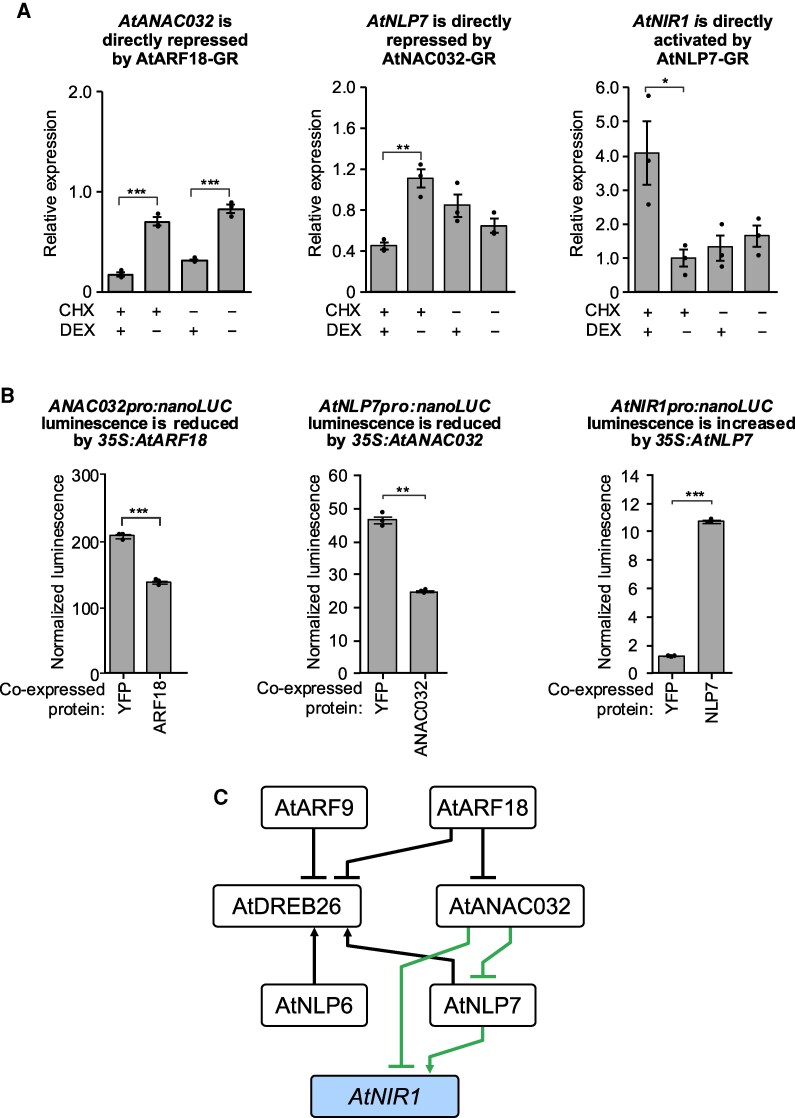
Examples of regulatory consequences of transcription factor expression in protoplasts. **A)** Changes in endogenous expression of target genes in the modified Transient Assay Reporting Genome-wide Effects of Transcription factors (TARGET) assays. Values represent the mean and standard error of 3 biological replicates of which each is the mean of two technical replicates. CHX = cycloheximide; DEX = dexamethasone. **B)** Changes in normalized luminescence of nanoluciferase (LucN) in response to transcription factor overexpression. Values represent the mean and standard error of 3 biological replicates. For both **A**) and **B**) *P*-values were calculated using an unpaired two-tailed Student's *t*-test with a minimal threshold of <0.05. *P < 0.05 **P < 0.01, ***P < 0.001 **C)** Schematic of sub-network illustrating the regulatory consequences (modified TARGET and/or co-expression data) of transcription factors that directly bind their target genes. Edges that are part of a feedforward loop are indicated in green. *AtNIR1* is indicated in blue as the representative metabolic target gene. An arrow indicates an activating interaction, while a perpendicular line indicates a repressive interaction.

Starting at the top of the predicted sub-network and working down, AtARF18 was found to directly repress *AtANAC032;* ANAC032 to directly repress *AtNLP7* and AtNLP7 to directly activate *AtNIR1* (Fig. 3, A–C; Supplementary Fig. S5). The expression of *AtDREB26* could not be monitored in a TARGET assay as CHX represses its expression (Brooks et al. 2019). However, in the transactivation LUC assay, *AtDREB26* expression was activated by AtNLP7 and AtNLP6 and repressed by AtARF18 (Supplementary Fig. S6). Despite the identification of binding sites for AtDREB26 in the regulatory regions of *AtNIR1* and in the coding sequence of *AtNLP7* (Supplementary Fig. S3), we did not identify any instances of direct regulation by AtDREB26 in the TARGET assay. Finally, while in vitro binding to their own promoters was detected for AtNLP6 and AtNLP7 (Supplementary Fig. S3) direct autoregulation was not detected. The resulting regulatory network contains a coherent feedforward loop regulation of *AtNIR1* by AtANAC032 via AtNLP7.

### Testing conservation and divergence of nitrate transcriptional and developmental regulation in tomato

A conserved role for homologs of *AtNLP7* in nitrate responsiveness has been demonstrated in several species including rice (Wu et al. 2021), and maize (Cao et al. 2017). We therefore investigated if this regulatory network is conserved in another dicot lineage. We selected tomato as one of the most important horticultural crops, with a preference for nitrate as an inorganic N source (Errebhi and Wilcox 1990). While tomato homologs of *AtNLP*7 have been identified (Liu et al. 2021b), the molecular mechanisms of nitrate uptake and assimilation are poorly understood in this species. Further, recent data has demonstrated that some aspects of root development and gene regulation are not conserved between Arabidopsis and tomato (Kajala et al. 2021).

**Figure 4.**
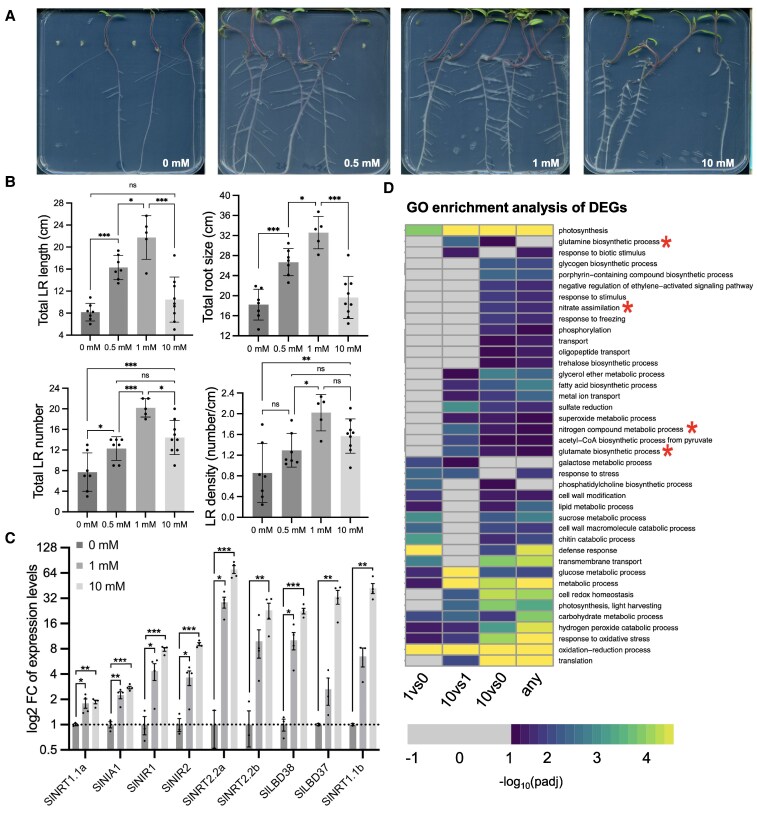
Root growth and gene expression response of tomato (M82) seedlings to potassium nitrate KNO_3_. **A)** Exemplar images of root system architecture (RSA) in increasing nitrate. Plants were imaged 10 days after germination on MS media (no nitrogen) supplemented with 0, 0.5, 1, and 10 mm KNO_3_. Plate dimensions are 120 × 120 mm. **B)** Quantification of root system architecture including total lateral root (LR) length, total root size (sum of main and lateral root lengths, total lateral root (LR) number, and lateral root (LR) density (ratio of lateral root number to main root length from M82 seedlings 10 days after germination (DAG) grown on 0, 0.5, 1, and 10 mm KNO_3_ (*n* = 7 seedlings). Statistical analysis was performed using one-way ANOVA with Tukey post-hoc test (adjusted *P*-value < 0.05). **C)** Log_2_ fold-changes of nitrogen-responsive genes. Dark gray represents expression in 0 mm KNO_3_, light gray in 1 mm KNO_3_, and pale gray in 10 mm KNO_3_. Asterisks indicate differentially expressed genes (DEGs) between N conditions by limma (false discovery rate, <0.05). ***adjusted *P*-value (padj) < 0.001, **P-value < 0.01,*P-value < 0.05. Data represent 4 biological replicates. **D)** GO enrichment analysis of DEGs between nitrate conditions in M82 RNA-seq experiment. Canonical nitrogen-mediated GO terms are marked with asterisks.

We first confirmed that there is a conservation of root system architecture and transcriptional regulatory responses to nitrate. To do this, we germinated and grew seeds for ten days on media supplemented with 0 to 10 mm KNO_3_. We observed that lateral root length and total root size increased from 0 mm to 1 mm with a decrease from 1 mm to 10 mm KNO_3_. However, this is non-linear, with growth decreasing at higher concentrations (Fig. 4, A and B). This is consistent with the scavenging behavior observed in Arabidopsis (Araya et al. 2014). We next tested the transcriptional influence of diverse concentrations of nitrogen. Samples were taken from roots grown in media supplemented with 0 mm, 1 mM, or 10 mm KNO_3_ and RNA was extracted. RNA-seq analysis revealed that canonical N-regulatory genes increased in expression dependent on nitrate availability, including high-affinity nitrate transporters, low-affinity nitrate transporters, nitrate assimilation enzymes such as nitrate reductase and nitrite reductase, and lateral organ boundaries domain-containing proteins (Fig. 4C). Analysis of gene ontology (GO) category enrichment indicates a likely conservation of downstream biological processes including N and nitrate assimilation, glutamine, and glutamate biosynthesis (Fig. 4D and Supplementary Fig. S7). Many of these nitrate-responsive genes were also significantly differentially expressed in response to nitrate in other studies (e.g. tomato NITRATE TRANSPORTER 1.1a *(SlNRT1.1a),* Solyc08g078950; tomato NITRATE TRANSPORTER 1.1b *(SlNRT1.1b),* Solyc08g007430; tomato NITRATE REDUCTASE 1 *(SlNIA1),* Solyc11g013810; tomato NITRATE TRANSPORTER 2.2a *(SlNRT2.2a),* Solyc11g069750; tomato NITRITE REDUCTASE1 *(SlNIR1),* Solyc01g108630; tomato NITRITE REDUCTASE 2 *(SlNIR2),* Solyc10g050890; tomato LATERAL ORGAN BOUNDARIES-DOMAIN 38 *(SlLBD38),* Solyc01g10719*0*) supporting their central nature in the nitrogen transcriptional response (Wang et al. 2001; Julian 2022; Sunseri et al. 2023).

### What genes control the tomato nitrogen response?

Given the conservation in N-mediated changes in root system architecture and transcription, we hypothesized that the underlying regulatory network is likely conserved between Arabidopsis and tomato. Using the elaborated network from Arabidopsis (Fig. 3C) as a base, we investigated if the tomato genome encoded likely orthologs of *AtARF18, AtARF9, AtDREB26, AtANAC032, AtNLP6, AtNLP7* and *AtNIR1.* As expected, given the history of tomato genome triplication (Tomato Genome Consortium 2012), duplicates or triplicates of many of these Arabidopsis genes were identified. Putative orthologs were first identified using phylogenetic trees (23) (Supplementary Fig. S8), and this gene set was refined by identifying the homolog(s) with expression in the tomato root (23). A clear likely ortholog was identified for *AtARF18* (Solyc01g096070), 2 potential *AtARF9* paralogs (Solyc08g008380 and Solyc08g082630), a single *AtDREB26* ortholog (Solyc11g012980), 2 genes corresponding to either *AtNLP6* or *AtNLP7* (Solyc08g008410 and Solyc08g082750), and paralogs of *AtNIR1* whose expression was confirmed to be N concentration-dependent (Solyc10g050890 and Solyc01g108630, Fig. 4C, Supplementary Fig. S8). Solyc08g008380 was expressed in the tomato root, while Solyc08g082630 was not expressed in the root (Supplementary Fig. S8B). Therefore, Solyc08g008380 was chosen for further analysis and was called *SlARF9B,* consistent with the naming in Zouine et al. (2014) . No clear *AtANAC032* ortholog was identifiable given the phylogeny. The first NLP gene, Solyc08g008410 was found to be mis-annotated and to contain 2 NLP duplicates (Supplementary Fig. S9), the latter of which is extremely lowly expressed. The *SlNLP* genes, both of which contain RWP-RK nitrate sensing domains, were named *SlNLP7A* (Solyc08g008410) and *SlNLP7B* (Solyc08g082750), as it was impossible to differentiate which could be the correct ortholog for *AtNLP6* or *AtNLP7* (Supplementary Fig. S9).

**Figure 5.**
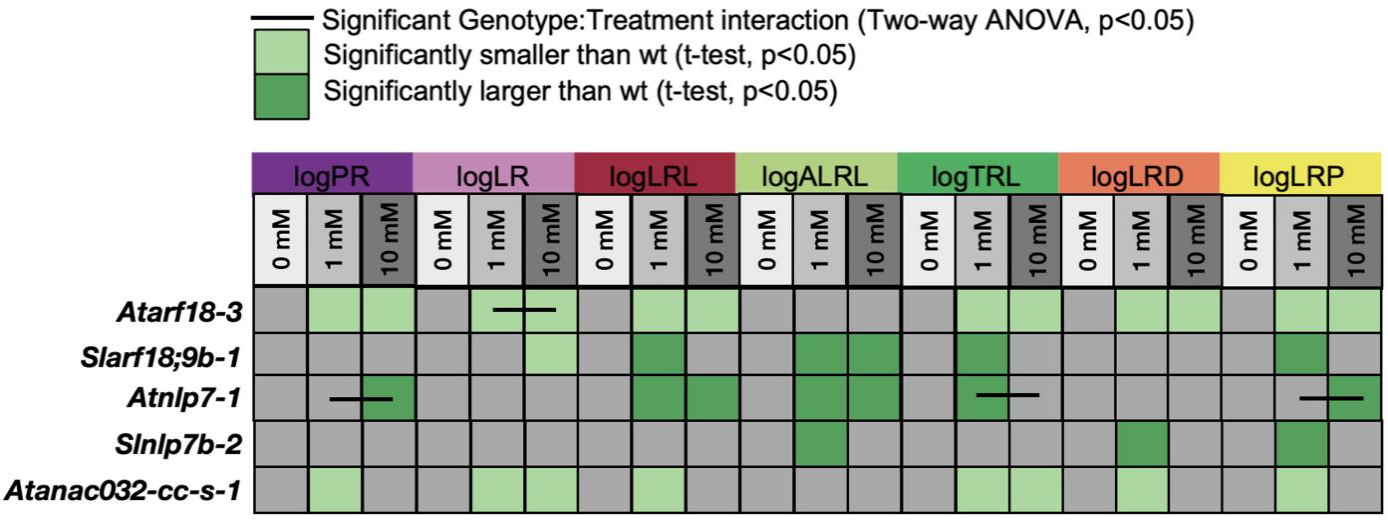
Tomato and Arabidopsis root system architecture traits in orthologous mutant backgrounds. Traits measured include the natural logarithm-transformed primary root length (logPR), number of lateral roots (logLR), total lateral root length (logLRL), average lateral root length (logALRL), total root length (logTRL), lateral root density (logLRD) and the ratio of lateral root length to total root length (logLRP). Traits were measured in 0, 1, and 10 mm KNO_3_ for tomato, and 1 and 10 mm KNO_3_ for Arabidopsis. Light green represents the trait as significantly smaller relative to wild type (wt), dark green represents the trait as significantly larger relative to wild type, white indicates no significant difference and gray indicates that this phenotype was not tested at this concentration (t-test, *P* < 0.05). A black bar indicates a significant genotype × treatment interaction as measured by a two-way ANOVA (*P* < 0.05). Col-0: *n* = 536; *Atarf18-3*: *n* = 34; *Atnlp7-1: n* = 29; *Atanac032-cc-s-1*: *n* = 44; M82: *n* = 61; *Slarf18;9b*: *n* = 33; *Slnlp7b-2*: *n* = 40.

Given the conservation of the wild-type tomato root N response relative to Arabidopsis, and the presence of an additional gene within the circuit in Arabidopsis compared to tomato (AtANAC032), we next tested the degree to which they are genetically conserved. We therefore monitored phenotypes of mutated genes at the top (*AtARF18* and *SlARF18)* and bottom (*AtNLP7* and *SlNLP7A/B)* of the circuit, as well as of *ANAC032* which is present only in Arabidopsis (Gaudinier et al. 2018). Root system architecture traits (primary root length, number of lateral roots, total lateral root length, average lateral root length, total root length, lateral root density, and ratio of lateral root length to total root length) were measured in limiting (1 mm KNO_3_ for Arabidopsis, 0 and 1 mm KNO_3_ for tomato) and sufficient (10 mm KNO_3_) for mutant alleles of these genes in Arabidopsis and tomato. This included 2 previously described mutant alleles of Arabidopsis *AtARF18*, *Atarf18-2* and *Atarf18-*3 (Gaudinier et al. 2018), new CRISPR–Cas9 generated alleles of *AtARF18* (*Atarf18-cc-1*) and *AtANAC032* (*Atnac032-cc-s-1*) (Supplementary Figs. S10 and S11). In tomato, guide RNAs were designed to target *SlARF18* and *SlARF9b* to reduce redundancy (Supplementary Fig. S11); and to *SlNLP7b*. In the Arabidopsis lines containing mutations of At*ARF18* (*Atarf18-2* and *Atarf18-3*) and *AtANAC032* (*Atnac032-1* and *Atnac032-cc-s-1*), a smaller root system was observed relative to the wild type (WT) in at least one concentration of KNO_3_ for any traits (Fig. 5 and Supplementary Fig. S12 and File 1). In contrast with the Arabidopsis *AtARF18* mutant alleles, *Slarf18;9b-1* had a larger root system architecture. This larger root system architecture was also observed in Arabidopsis mutant alleles of AtNLP7 (Fig. 5, Supplementary Fig. S12 and File 1). Given these similarities and differences, we next looked at the degree of conservation of the network at molecular resolution.

### In vitro characterization of tomato cis-regulatory architecture

**Figure 6.**
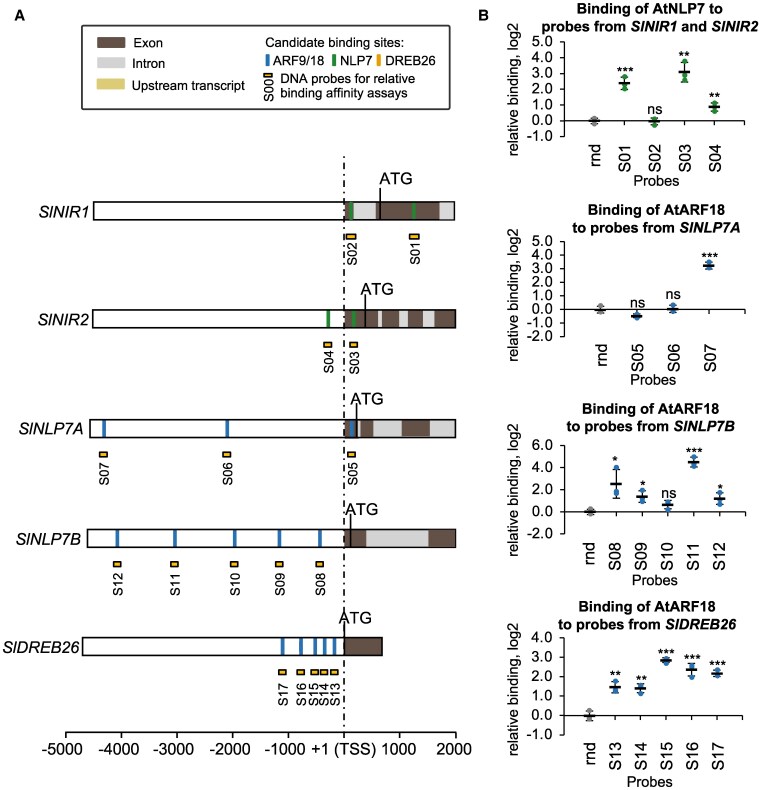
Transcription factor-binding motifs and in vitro assays support some conservation of the regulatory sub-network in tomato **A)** Position weight matrices (PWMs) were used to identify candidate transcription factor binding sites in the 5′ regions (*x*-axis—up to 5 kilobases upstream (−) and 2 kilobase downstream of the transcription start site (TSS) (+)) of SlNIR1, SlNIR2, SlNLP7A, SlNLP7B and SlDREB26. Colors are the same as in Fig. 2. **B)** In vitro relative binding assays showing binding of AtARF18 and AtNLP7 to candidate binding sites in SlNIR1, SlNIR2, SlNLP7A, SlNLP7B, and SlDREB26, respectively. Error bars = mean and standard deviation; *n* = 3 technical replicates; *P*-values were calculated using an unpaired two-tailed Student's *t*-test of each sample to the random (rnd) control probe; *P < 0.05, **P < 0.01, ***P < 0.001; ns = not significant.

To investigate the conservation of the network, we first investigated the upstream regulatory regions of *SlNIR1, SlNIR2, SlNLP7A, SlNLP7B,* and *SlDREB26* to determine if there were candidate binding sites for AtARF18/9; AtDREB26 and AtNLP6/7 TFs by using FIMO with publicly available position weight matrix (PWM) data (Bailey et al. 2015; O’Malley et al. 2016) (Fig. 6A). There is extensive conservation of DNA-binding domains between species (Weirauch et al. 2014; O’Malley et al. 2016), and we tested if this was the case for the DNA-binding domains of the Arabidopsis and tomato TFs within the network. Indeed, extensive conservation between Arabidopsis and tomato was observed for the AtARF, AtDREB, and AtNLP factors (Supplementary Fig. S13). As a proof-of-principle, we tested if Arabidopsis AtNLP7 and AtARF18 transcription factors were able to bind in vitro to candidate sites identified in the *SlNLP7A, SlNLP7B, SlNIR1,* and *SlNIR2* promoters (Fig. 6B). Interactions between AtARF18 and *SlDREB26* as well as *SlNIR1* were conserved, as were interactions between AtNLP7 and *SlNIR1/SlNIR2*. In contrast, AtARF18 was able to bind to the promoters of *SlNLP7A* and *SlNLP7B*—in Arabidopsis, interactions of AtARF18 with *AtNLP6*; as well as AtARF18 with *AtNLP7*, were not observed and represent divergence between Arabidopsis and tomato (Supplementary Fig. S3).

### Regulatory interactions are partially conserved between Arabidopsis and tomato

The in vitro TF-DNA-binding assays, plus the lack of identification of a clear tomato ANAC032 ortholog, suggested distinct differences in the wiring of these N-metabolic regulatory networks both in terms of binding and regulation. To determine which of these represent direct regulatory interactions, we generated glucocorticoid receptor (GR) fusions of the SlARF9B, SlARF18, SlDREB26, SlNLP7A, and SlNLP7B transcription factors. In order to eliminate dosage-dependent effects, we introduced these constructs into their respective mutant backgrounds, as generated by CRISPR–Cas9 gene editing using *R. rhizogenes*-mediated transformation. (Kajala et al. 2021). These roots, hereby called “hairy roots’’ have similar growth and anatomy to primary roots (Ron et al. 2014) and the same cell type expression as observed with transcriptional reporters when compared with *A. tumefaciens*-transformed plant roots. Hairy roots also respond similarly to increasing concentrations of available nitrate, although with higher sensitivity at 1 mm KNO_3_ compared to wild-type roots. This was demonstrated by nitrate-dependent increases in the expression of *SlNIR1* and *SlNIR2* as determined by RT-qPCR, by comparing RNA-seq of M82 roots with RNA-seq from hairy roots, and as observed by nitrate-responsive nuclear-localized GFP expression driven by the *AtNIR1* and *AtNRP* promoters in 0, 1 and 10 mm KNO_3_ in hairy roots, compared to *A. tumefaciens*-transformed *AtNRP*:GFP (Supplementary Fig. S14). CRISPR-edited mutant alleles were confirmed by amplicon-based sequencing (Supplementary Fig. S11). As with the assays in Arabidopsis (Fig. 3, Supplementary Fig. S5), transcription factor activity was induced by application with dexamethasone, and direct targets were confirmed by the addition of cycloheximide.

**Figure 7.**
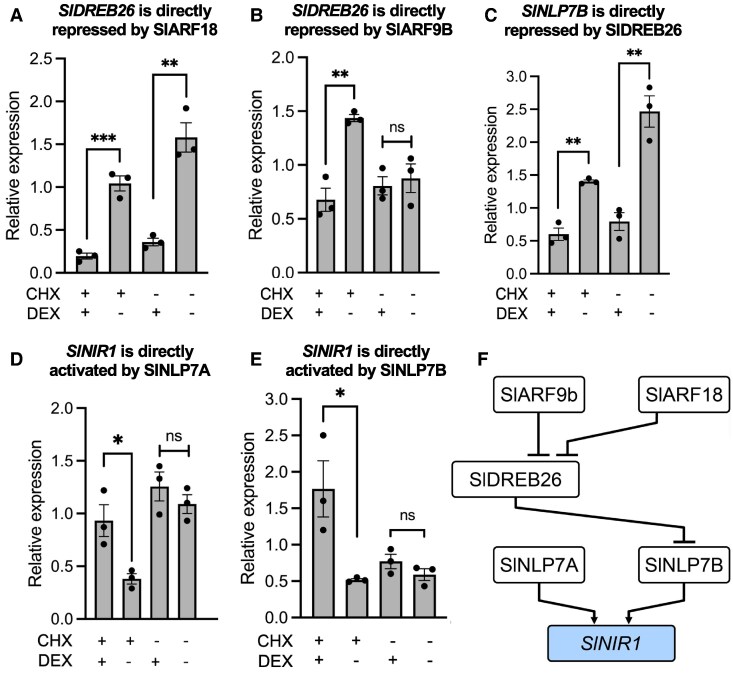
Examples of regulatory consequences of transcription factor expression in tomato root protoplasts via modified TARGET assays. Changes in endogenous gene expression of target genes in modified TARGET assays. *SlDREB26* expression levels are changed by **A)** SlARF18 and **B)** SlARF9B, **C)**  *SlNLP7B* expression levels are changed by SlDREB26, *SlNIR1* levels by **D)** SlNLP7A and **E)** SlNLP7B. DEX = dexamethasone; CHX = cycloheximide. Values represent the mean and standard error of 3 biological replicates of which each is the mean of two technical replicates. *P*-values were calculated using an unpaired two-tailed Student's t-test, *P < 0.05, **P < 0.01, ***P < 0.001, and ****P < 0.0001. *N* = 3. ns = not significant **F)** Schematic of sub-network illustrating the regulatory consequences of transcription factors that directly bind their target genes in tomato. Light blue = metabolic gene (SlNIR1). Arrowhead = activating, perpendicular lines = repressing interactions.

Although the Arabidopsis AtARF18 was able to bind to the *SlNLP7A* and *SlNLP7B* promoters, this was not reflected in the modified TARGET assay. However, an indirect repressive interaction was observed (Supplementary Fig. S15). SlARF18 and SlARF9B directly bound to the promoter of *SlDREB26* and repressed its expression (Fig. 7, A and B). In turn, SlDREB26 was found to directly repress the expression of *SlNLP7B* (Fig. 7C). SlNLP7A and SlNLP7B both bind to and activate the expression of *SlNIR1* (Fig. 7, D and E). In several cases, these represent conserved regulatory interactions between Arabidopsis and tomato. At/SlARF18 and At/SlARF9 both repress the transcription of *At/SlDREB26*, and SlNLP7A/B/AtNLP6/7 activate the transcription of *At/S*l*NIR1*. Major differences in these networks include the tomato SlDREB26 repression of *SlNLP7B* which is not observed in Arabidopsis, the Arabidopsis AtNLP6 and AtNLP7 activation of *SlDREB26* which is not observed in tomato, and the Arabidopsis AtNAC032 interactions which were not able to be tested in tomato due to the lack of a clear ortholog. No feedforward loops were identified in tomato, although they were present in Arabidopsis (Fig. 7F).

### Testing the network models using genetic perturbation

Collectively, these data in both Arabidopsis and tomato provide a predictive model of genetic regulation of plant N metabolism and demonstrate differences in their wiring and components. In summary, SlDREB26 is a key intermediary between SlARF18/9 and SlNLP7A/7B in tomato, as opposed to a target of both AtARF18/9 and AtNLP6/7 in Arabidopsis. A coherent feedforward loop exists between AtANAC032, AtNLP7, and *AtNIR1* in Arabidopsis, but AtANAC032 is likely absent in tomato. AtNLP6 and AtNLP7 activate the expression of *DREB26* and result in interconnected feedforward loops in Arabidopsis (Fig. 3C), but these regulatory interactions are absent in tomato (Fig. 7F).

**Figure 8.**
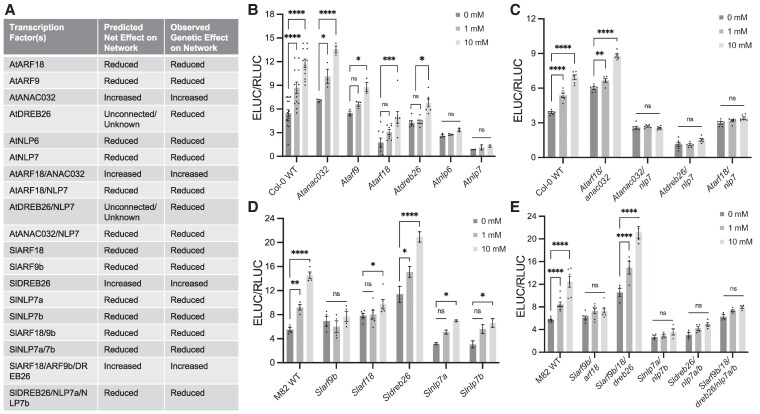
NRP:reporter (PAROT) assay in Arabidopsis and tomato mutants. **A)** Regulatory models from Fig. 3, C and 7, F were used to predict the net effect on transcriptional regulation of nitrogen metabolism in the respective single and higher-order mutant combinations as measured using the NRP reporter and the PAROT assay (predicted net effect on network). The observed output on the network is summarized in the third column. **B–E)** Observed changes in the network compared to wild type as quantified in a PAROT assay in which protoplasts are transformed with a dual vector setup where the nitrate-responsive AtNRP drives the expression of emerald luciferase (ELUC) and the constitutive nopaline synthase promoter drives expression of red luciferase (RLUC). Changes in normalized luminescence of ELUC in response to differing concentrations of nitrate in **B**) Arabidopsis Col-0 WT, *Atanaco32-cc-s-1, Atarf9b, Atarf18-2, Atdreb26, Atnlp6,* and *Atnlp7-1*  **C**) Arabidopsis Col-0 WT, *Atarf18-2/anac032-cc-s-1, Atanac032-cc-s-1/nlp7-1, Atdreb26c/nlp7c*, and *Atarf18-2/nlp7-1*  **D**) Tomato M82 WT, *Slarf9b #8, Slarf18 #2, Sldreb26 #10, Slnlp7a #1,* and *Slnlp7b #13*  **E**) Tomato M82 WT, *Slarf9b/18 #4, Slarf9b/18/dreb26 #9, Slnlp7a/7b #21, Sldreb26/nlp7a/7b #6*, and *Slarf9b/18/dreb26/nlp7a/7b #2* at 0, 1, and 10 mm KNO_3_. Error bars are standard error; *N* = 4 biological replicates (different protoplast batches from hairy roots); *P*-values were calculated using two-way ANOVA with Tukey's multiple comparisons test; *P < 0.05, **P < 0.01, ***P < 0.001; ****P < 0.0001, ns = not significant.

In a classically defined systems biology approach, the predictive nature of models can be tested using genetic perturbation (Ideker et al. 2001). Protoplast-based assays have been highly effective at elucidating multiple aspects of the Arabidopsis nitrogen transcriptional regulatory network (Para et al. 2014; Brooks et al. 2019; Alvarez et al. 2020). We hypothesized that a protoplast-based assay could be used to test multiple Arabidopsis and tomato mutant combinations in different concentrations of available nitrogen. We first tested the nitrate response of Arabidopsis and tomato protoplasts by monitoring the expression of *AtNIR1, AtNIA1, SlNIR1,* and *SlNIA1* via qRT-PCR. Their expression was higher as the concentration of nitrate was higher (Supplementary Fig. S16). As a quantifiable output of the nitrogen network, we used the synthetic *NITRATE-REGULATED PROMOTER (NRP)* (Wang et al. 2010) fused to luciferase (Supplementary Fig. S16). This reporter is robustly activated as concentrations of available nitrate increase and has been used in both Arabidopsis and crop plants (Wang et al. 2010; Guan et al. 2014; Cao et al. 2017) and in tomato (Supplementary Fig. S14). As the *NRP* comprises large promoter fragments that contain other binding sites, we additionally tested an optimized version of a previously reported 4xNRE minimal synthetic promoter (Konishi and Yanagisawa 2010), which exclusively contains 4 copies of an NLP7 binding motif. These reporters were transfected into protoplasts derived from wild-type Arabidopsis or tomato roots. Their expression similarly increased as nitrate concentrations increased (0, 1, and 10 mM KNO_3_, Supplementary Fig. S16). Protoplast viability was maintained at a minimum of 60% at 24 h after transfection (Supplementary Fig. S16). To test the influence of TF genetic perturbation, we monitored NRP and 4XNRE reporter activity in protoplasts from NLP6/7 mutant Arabidopsis and tomato roots (*Atnlp6, Atnlp7-1,* and *Slnlp7a Sl 7b).* Luciferase was quantified relative to Col-0 primary roots or empty vector-transformed M82. In all mutants, the responsiveness of the NRP and the 4XNRE was vastly lower, concordant with *AtNIR1/SlNIR2* expression, respectively (Fig. 8, Supplementary Fig. S16). We named this assay PAROT: Protoplast Assay Reporting Overall Effects of TFs and subsequently used the NRP reporter to monitor the influence of multiple mutant combinations on the transcriptional regulation of nitrogen metabolism in Arabidopsis and tomato.

Ten mutant genotypes were generated for Arabidopsis, and nine for tomato, the latter using hairy roots (Supplementary Figs. S10 and S11). Given the regulatory network architecture in Figs. 3C and 7F, we first predicted the impact of these mutations and their combinations. In both Arabidopsis and tomato, ARF9 and ARF18 participated in a direct repressive and indirect repressive interaction upstream of *NIR1*. We thus predicted that NRP activity would be lower. In both species, NLP6/7 directly activated the expression of *NIR1*, and thus, their predicted effect would also reduce NRP activity. DREB26 was unconnected to NIR1 in Arabidopsis and thus its effect could not be predicted, while in tomato it would act as a repressor and thus NRP activity would be higher. ANAC032 in Arabidopsis directly repressed both *NIR1* and *NLP7*, and its perturbation was thus predicted to increase NRP activity. In predicting higher-order genetic interactions, we assumed that the downstream factor's phenotype would be epistatic. For instance, in an Arabidopsis *Atarf18 At anac032* double mutant, since ANAC032 acts as a repressor, we predicted that NRP activity would be higher. Similarly, in tomato, in an *Slarf18 Slarf 9b Sl dreb26* mutant, the *Sldreb26* phenotype would be epistatic and NRP activity would be higher; however, in a *Sldreb26 Sl nlp7a Sl nlp7b* mutant, the *slnlp7* phenotype would be epistatic and NRP activity would be lower.

**Figure 9.**
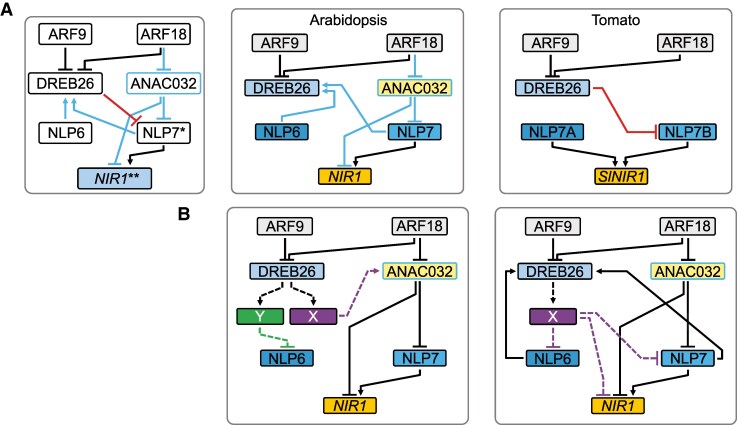
Conserved and species-specific direct regulatory interactions in Arabidopsis and tomato. **A)** Three direct, regulatory interactions are conserved between Arabidopsis and tomato transcription factors and targets (black edges). Species-specific regulatory interactions are depicted by blue edges for Arabidopsis and red edges for tomato. * = SlNLP7a and SlNLP7b, AtNLP7, ** = AtNIR1, and SlNIR1 and SlNIR2. The species-specific circuits are shown to the right, with orthologous genes shown in the same respective colors. Interactions were determined using the modified TARGET assays. **B)** Two potential regulatory models that explain the observed influence on the nitrogen transcriptional network in the Arabidopsis DREB26 mutant allele (Fig. 8) and which incorporate data regarding the indirect repressive interaction of AtDREB26 on *AtNLP6*, *AtNLP7, and AtNIR1* (Fig. 3 and Supplementary Fig. S6). *X* and *Y* indicate 2 as yet unidentified transcription factors. The color scheme represents likely orthologs between Arabidopsis and tomato as in the species-specific panels in **A)**.

In all cases, the experimentally observed interactions matched our predictions and supported the regulatory models. In both Arabidopsis and tomato, a single *Atnlp6* or *Atnlp7* or *Slnlp7a* or *Slnlp7b* mutant alone showed less sensitivity in its NRP response to increasing nitrogen, with the double in tomato showing near-complete insensitivity (Fig. 8, C and E, Supplementary Fig. S17A–D). The NLP7 mutant phenotypes are consistent with AtNLP7 as a major regulator of *AtNIR1* expression in Arabidopsis (Alvarez et al. 2020; Konishi et al. 2021 ) and tomato (Fig. 5). The more extreme phenotype of the *Slnlp7a Sl 7b* mutant and the *Slarf18 Slarf 9b* mutants and SlNLP7 as a major regulator of SlNIR1 in tomato indicates that they both additively contribute to nitrogen responsiveness (Fig. 8, D and E). Our assumption regarding epistasis was also experimentally validated with the *Atanac032* phenotype being epistatic to *Atarf18*, the *Atnlp7* phenotype being epistatic to *Atanac032* and *Atarf18* (Fig. 8C). In tomato the Sl*dreb26* phenotype was epistatic to *Slarf18 Sl arf9b*, and the *Slnlp7a Slnlp7 b* phenotype was epistatic to *Sldreb26*, and even to *Slarf9b/18/dreb26* (Fig. 8, D and E). Collectively, these results support a hierarchy whereby ARF18/9 are at the top of the regulatory network, and NLP6/7 at the bottom of the network. These conserved interactions are demonstrated in Fig. 9A and Supplementary Fig. S18. In Arabidopsis, AtANAC032 acts as an intermediary in the network as does SlDREB26 in tomato (Fig. 9A). Monitoring the expression of *NIR1* as a readout in the modified TARGET assays was also an effective target to explore the output of the transcriptional nitrogen network.

One remaining conundrum was the role of AtDREB26 in the network. In Arabidopsis, AtDREB26 indirectly repressed *AtNLP6, AtNLP7*, and At*NIR1*. In the PAROT assay, mutation of *Atdreb26* resulted in decreased sensitivity of the NRP reporter. Furthermore, the *Atdreb26 At nlp7* double mutant showed increased insensitivity relative to the single *A tdreb26* mutant phenotype, but similar insensitivity to the *Atnlp7* mutant phenotype. We thus predicted 2 potential regulatory scenarios. In the first, AtDREB26 indirectly represses *AtNLP7* and *AtNIR1* through an unknown gene X which activates *AtANAC032*. AtDREB26 also represses *AtNLP6* via an unknown gene Y (Fig. 9B). In a second scenario, AtDREB26 represses *AtNLP6, AtNLP7* and *AtNIR1* through an unknown gene X (Fig. 9B).

## Discussion

Changes in N availability are perceived and transduced via a series of signaling events culminating in rapid changes in transcription followed by changes in plant growth for adaptive purposes (Marchive et al. 2013; Guan et al. 2014). In order to engineer plants with increased N-use efficiency via transcriptional regulation, we need specific knowledge of which transcription factors are involved, and which target sites they bind. We also need to understand the nature of these gene pathways, their direct and indirect interactions, and how transcriptional information flows through these pathways to transcriptionally regulate N metabolism.

NLP7 is a critical transcriptional regulator of N metabolism, and its activity is controlled by multiple factors—it directly senses nitrate, its nuclear localization is controlled by phosphorylation, and an understudied mode of its regulation is by control of its transcript abundance (Marchive et al. 2013; Liu et al. 2022). Increasing transcription of *AtNLP7* results in improved plant growth and C:N balance (Yu et al. 2016), and is used as a measure of the physiological significance of N-regulatory factors (Brooks et al. 2019), demonstrating the importance of its transcriptional regulation Transcriptional activity of AtNLP7 can be read out by changes in transcript abundance of its direct downstream targets, like that of *AtNIR1* (Alvarez et al. 2020; Hummel et al. 2023). NIR1 is a key enzyme in nitrogen assimilation, reducing nitrite to ammonium. Mutation of *AtNIR1*, similar to that of *AtNLP6/NLP7* and *AtNRT1.1*, results in chlorotic plant growth and perturbed nitrogen metabolism (Tsay et al. 1993; Costa-Broseta et al. 2020; Cheng et al. 2023). Therefore, precise and likely subtle changes in the transcription of these genes will exert a meaningful influence on N metabolism and plant growth. Indeed, this is viewed in nature, where differences in transcription of *AtNIR1, AtNIA1,* and *AtNRT1.1* are associated with changes in nitrogen sensitivity in genetically varying Arabidopsis populations (North et al. 2009; Sakuraba et al. 2021).

N-mediated coordination of growth occurs via action in spatially restricted cell types (Zhang and Forde 1998, 2000; Gifford et al. 2008; Vidal et al. 2010). In addition, there is complex interconnection and feedback between N signals, transcription, and hormone-mediated control of growth (Krouk et al. 2010a; Song et al. 2013; Ma et al. 2014; Vega et al. 2019). Given this multi-layered and spatiotemporally diverse regulation, network-focused studies utilizing TARGET, protoplasts, or other heterologous systems prove effective at elucidating the underlying architecture of nitrogen-mediated regulation (Bargmann et al. 2013; Para et al. 2014; Gaudinier et al. 2018; Brooks et al. 2019; Alvarez et al. 2020). Our approach and findings complement these many elegant network-focused studies (Krouk et al. 2010b; Para et al. 2014; Doidy et al. 2016; Varala et al. 2018; Heerah et al. 2019; Alvarez et al. 2020; Brooks et al. 2021; Cheng et al. 2021; Contreras-López et al. 2022) by focusing on a five-gene regulatory circuit predicted to contain multiple feedforward loops and an autoregulatory interaction which acts upstream of the largely understudied transcriptional regulation of *AtNLP6* and *AtNLP7* and its closely related paralog *AtNLP6*.

Dissecting the necessity of cis-regulatory elements or cis-regulatory modules has defined rules for cell type-specific expression in the bundle sheath (Dickinson et al. 2020) and for tomato fruit size and maize yield-related traits (Rodríguez-Leal et al. 2017; Liu et al. 2021a). We first sought to identify cis-regulatory elements that define regulatory relationships within this gene circuit. These transcription factors had not been tested in previous genome-wide TF-directed approaches (Weirauch et al. 2014; O’Malley et al. 2016 ). We decreased the set to those present within accessible chromatin regions (Potter et al. 2018), and then, our in vitro binding assays (Cai et al. 2023) further restricted these to elements which were able to directly recruit the factors under study (Fig. 2B). Collectively these indicated a complex sub-circuit comprising 29 interactions via 46 cis-elements, albeit without the contribution of AtARF9 due to technical limitations (Fig. 2C). The modified TARGET assay dramatically decreased the 29 possible interactions to 8 via 15 putative cis-regulatory elements (Supplementary Fig. S3). This decreased set is highly conservative given that they do not reflect N-inducibility nor cellular context. Transient interactions are also under-represented in this assay type but are prevalent within the N-regulatory network at large (Alvarez et al. 2020). The resulting sub-network architecture contains several interaction types that will be exciting in the future to monitor in the context of generating dynamic, non-linear behavior. *AtANAC032* controls N-mediated regulation of root system architecture (Fig. 5), and it participates in a coherent feedforward loop along with AtNLP7 and AtNIR1 coherent feedforward loop. The final outcome of this feedforward loop in terms of its temporal and spatial consequences remains to be determined. The mode by which AtDREB26 is able to influence *AtNIR1* expression also remains to be elucidated, using the predictive models guided by the experimentally generated genetic data (Figs. 8 and 9). While Arabidopsis serves as an excellent organism to study genome-wide transcriptional regulation of N metabolism, an important question is how research in this species can be translated to a crop like tomato. The molecular genetic nature of tomato N-metabolic gene regulation has been minimally studied (Chapin et al. 1988; Wang et al. 2001; Asins et al. 2017; Julian et al. 2023; Sunseri et al. 2023), likely due to its status as a specialty crop species that is most often grown in smaller-scale agricultural systems with unlimited access to mineral nutrients. Our analysis of root system architecture and the global transcriptional response to differing concentrations of available N indicates a remarkable similarity to the Arabidopsis response. At the molecular level, there were some differences in gene content with at least 2 tomato *NIR*, *NRT* and *ARF9* genes relative to their Arabidopsis counterparts, and the lack of an identifiable *AtANAC032* ortholog. While both the Arabidopsis and tomato At/SlNLP7 mutant alleles demonstrated similar N-dependent changes in root system architecture, those of At/SlARF18/9 demonstrated some similarities and differences, suggesting changes in their regulatory network (Fig. 5). Dissection of the tomato circuit with both the in vitro and in vivo assays provided evidence of likely differences in their genetic architecture. The in vitro assays with the Arabidopsis TFs and tomato-predicted cis-regulatory elements suggested that interactions could exist between the 7 tomato genes via twelve cis-elements (Fig. 6). Modified TARGET assays further confirmed 5 of these interactions in tomato as direct and regulatory (Fig. 7F). The remaining that could not be validated could represent a true lack of interaction, or transient interactions not able to be captured by this system (Alvarez et al. 2020). No feedforward loops were identified in tomato (Fig. 7). Key changes in circuitry between the 2 species include (i) the repressive function of SlDREB26 on *SlNLP7B* while in Arabidopsis AtDREB26 does not regulate any target genes; (ii) in Arabidopsis AtNLP6 and AtNLP7 activate *AtDREB26* transcription with no such interaction in tomato; and (iii) the lack of AtANAC032 in the tomato module. Molecularly, the observed variation in regulatory interactions (Figs. 3C and 7F), and the similarity in N-dependent root system architecture of the tomato and Arabidopsis *At/Slnlp7* mutants suggests convergent function of their respective circuits.

The regulatory models presented in Fig. 3C were experimentally tested by systematic genetic perturbation. We drew on approaches used in Arabidopsis to monitor the transcriptional outcomes of auxin and cytokinin hormone signaling pathways via a synthetic reporter (Ulmasov et al. 1997; Brunoud et al. 2012; Zürcher et al. 2013)—the nitrogen-responsive NRP, and used a refined NRP with 4 repeats of the NLP7 binding site (Supplementary Fig. S16) (Wang et al. 2010). The advantage of this protoplast-based approach is that we could test the N-dependent inducibility of these different genes and their interactions. Remarkably, using single to quintuple mutant combinations, the regulatory models in Figs. 3C and 7F were able to be genetically validated in the PAROT assays. In the future, the spatial and temporal action of these factors remains to be elucidated.

Collectively, these data illustrate the divergence and conservation of an N-mediated transcriptional regulatory circuit in Arabidopsis and tomato. The circuit is undoubtedly more spatially and temporally complex and involves the interaction with many more genes. However, the fact that these assays were used to generate the 2 predicted and then genetically validated models, in terms of NLP7-directed transcriptional activity, suggests that they provide an important avenue to elucidate significant regulators of NLP7. Further, the tomato root system architecture mutant phenotypes demonstrate that these genes are physiologically relevant in a crop species. The protoplast assays reflect a whole-organ response and root system architecture traits are determined by the activities of discrete populations of cells in either xylem pole pericycle cells, lateral root founder cells, and the root stem cell niche. In the future, cellular-resolution assays must be used to untangle these aspects of the network and how they coordinate changes in N concentration with plant growth. These data provide a foundation to engineer robust nitrogen regulatory circuits in both species. The identified cis-regulatory modules serve as potential control points. It remains to be determined in each species exactly which cis-elements function in a given cell type or N concentration, as well as how or if these transcription factors coordinately act together to regulate target gene expression. Is this by distinct affinities of the transcription factor for its target or differing concentrations of the transcription factor itself? Is there competition between transcription factors in the case of feedforward loops? Indeed, many questions remain to be answered, but this work demonstrates how diverse assays can be used to provide blueprints for the elucidation of network architecture and for future synthetic approaches to engineer improved N-use efficiency in plant roots.

## Materials and methods

### Identification of candidate transcription factor-binding sites

Upstream Arabidopsis sequence regions consisting of 2 kilobases (kbs) upstream of the earliest annotated transcription start site (TSS) and 1-kb downstream *of ARABIDOPSIS NAC-DOMAIN CONTAINING PROTEIN 32* (*AtANAC032*; AT1G77450), A*UXIN RESPONSE FACTOR 18* (*AtARF18*; AT3G61830), *AUXIN RESPONSE FACTOR 9* (*AtARF9*; AT4G23980), *NIN-LIKE PROTEIN 6* (*AtNLP6*; AT1G64530), *NIN-LIKE PROTEIN 7* (*AtNLP7;* AT4G24020), *DEHYDRATION-RESPONSIVE ELEMENT-BINDING PROTEIN 26* (*AtDREB26*; AT1G21910), and *NITRITE REDUCTASE* (*AtNIR1*; AT2G15620) were extracted from the Arabidopsis TAIR 10 (Lamesch et al. 2012) genome assembly. These regions were truncated if they overlapped with the transcript of a protein-coding gene. Alternatively, they were extended if a longer region was included in previously reported yeast-1-hybrid data (Gaudinier et al. 2018). ATAC-seq data from (Potter et al. 2018) were used to identify regions of open chromatin in Arabidopsis (*Arabidopsis thaliana)* roots and shoot tissues. Individual .bed files for each replicate were concatenated and BEDTools (v2.29.2) merge (Quinlan and Hall 2010) was used to combine overlapping peaks. Candidate binding sites for AtNLP7 (TGNCYYTT) and AtDREB26 (YCRCCGHC) were identified using PWMs from DAP-seq (O’Malley et al. 2016) in FIMO (v5.4.1) (Grant et al. 2011) with a *P*-value cutoff of 10^−4^ and a zero-order background model created using fasta-get-markov (Bailey et al. 2015). Motifs with *q*-value <0.05 were considered high-confidence binding sites. PWMs are provided in Supplementary Fig. S1. As no empirical binding motif data was available for AtNLP6, AtARF9, AtARF18, or AtANAC032, we identified closely related proteins from the same TF families for which data was available and compared sequence identity within the DNA-binding domains (Supplementary Fig. S2). Finding these domains to be highly conserved, the PWM for AtNLP7 was also used to predict sites for AtNLP6 (TGNCYYTT), and the PWM for ATAF1 (KACGTR) was used to predict binding sites for ANAC032 (Supplementary Fig. S1). The PWM for AtARF2 was used to predict binding sites for AtARF9 and AtARF18. In addition, TGTCTC and TGTCGG motifs were also annotated as candidate sites as they have previously been shown to bind auxin response factors (ARFs) with higher affinity than other auxin response elements (Freire-Rios et al. 2020). Finally, pairs of TGTCNN motifs spaced less than 14 bp apart were also annotated as candidate ARF TFBSs, as paired motifs were previously shown to be enriched and preferentially bound in DAP-seq data (Freire-Rios et al. 2020; Cancé et al. 2022).

For tomato (*Solanum lycopersicum)*, upstream regions consisting of 5-Kbs upstream and 2-Kbs downstream of the annotated TSS of *SlDREB26* (Solyc11g012980), *SlNIR1* (Solyc10g050890), *SlNIR2* (Solyc01g108630), *SlNLP7A* (Solyc08g008410) and *SlNLP7B* (Solyc08g082750) were extracted from *Solanum lycopersicum* ITAG5.0 (https://phytozome-next.jgi.doe.gov/). To confirm similarity, the DNA-binding domains of tomato and Arabidopsis orthologs of ARF9, ARF18, DREB26, NLP6, and NLP7 were aligned (Supplementary Fig. S13). Candidate binding sites in the upstream regions of the tomato genes defined above were then analyzed using the same PWMs and parameters as defined above for Arabidopsis. A custom Jupyter Notebook (Granger and Pérez 2021) was used to create schematics of upstream regions illustrating gene features, candidate binding sites, and open chromatin.

### In vitro transcription factor-binding assays

Coding sequences of AtANAC032, AtARF18, AtARF9, AtNLP6, At NLP7, and AtDREB26 were amplified using primers to introduce the Gateway recombinase sequences attB1 and attB2 and a C-terminal HiBIT tag. Amplicons were cloned into pDONR207 (attP1-ccdB-attP2) to create pENTR clones (attL1-TF:HiBiT-attL2) using GatewayBP ClonaseII Enzyme Mix (Invitrogen). The resulting pENTR clones were combined with the Gateway-ready pDEST vector, pH9GW(attR1-ccdB-attR2), a modified pET28a(+) vector (Novagen), using the GatewayLR ClonaseII Enzyme Mix (Invitrogen). Details of all plasmids are available in Supplementary Data Set 2. Sequence-verified expression clones were transformed into *E. coli* BL21, and cultures were grown and protein purified as described in (Cai et al. 2023). Protein yield was quantified by measuring luminescence with the Nano-Glo HiBiT Extracellular Detection System (N2420, Promega, Madison, WI) and HiBiT Control Protein (N3010, Promega). We were unable to obtain the soluble protein of AtARF9. Details of all plasmids are available in Supplementary Data Set 2. Initially, the binding of each TF to a double-strand probe (DSPs) containing a known binding site was verified (Cai et al. 2023). Subsequently, the ability of each TF to bind to DSPs containing candidate binding sites from Arabidopsis and tomato promoters, relative to random DSPs, was quantified using the same method. Details of all probe sequences are available in Supplementary Data Set 1.

### Modified transient transformation system for genome-wide transcription factor target discovery (TARGET) assay

For Arabidopsis, constructs were generated using the Loop assembly toolkit (Pollak et al. 2019) using one-step restriction-ligation protocols detailed in (Cai et al. 2020). Coding sequences of each TF were cloned (concurrently removing the stop codon as well as any internal instances of BsaI and SapI with point mutations to introduce synonymous codons with similar Arabidopsis codon usage) into pUAP4 (Pollak et al. 2019) to create Level 0 standard parts comparable with the Phytobrick standard (Patron et al. 2015). These level 0 parts were assembled into the level 1 Loop acceptor (pCk2; Addgene #136696) in a one-step restriction-ligation reactions with a double CaMV35s- ΩTMV promoter/5′ UTR (pICH51288; Addgene #50269), a C-terminal GR tag (pEPOZ0CM0137; Addgene #197535) and the CaMV35s terminator (pICH41414). Details of plasmids are in Supplementary Data Set 2. Col-0 seeds were germinated and grown in potting medium (two-part sieved compost to one-part sand) within controlled environment chambers with a 16 h photoperiod at 22 °C with 120 to 180 *μ*mol/m^2^/s light intensity. The photoperiod was decreased to 8 h two days before leaves were harvested for the preparation of mesophyll protoplasts. Mesophyll protoplasts were prepared from *A. thaliana* Col 0 as previously described (Yoo et al. 2007). For each transcription factor, protoplasts were quantified and divided into twelve aliquots of 200 *µ*L in transfection buffer (0.4 m mannitol, 15 mm MgCl_2_, 4 mm MES, pH 5.6), each containing approximately 1×10^4^/mL intact protoplasts. In addition, for each batch of protoplasts, 3 aliquots were transfected with a control plasmid expressing nuclear-localized YFP. Plasmid DNA was prepared using the Plasmid Plus Midi kit (Qiagen, Hilden, Germany) except that 3 additional wash steps were performed before DNA elution from the column. 10 *μ*g of purified DNA in transfection buffer (2 g of PEG Mn 4000 (Sigma, 81240) in 2 mL of 500 mm mannitol and 0.5 mL of 1 m CaCl_2_) was mixed with each aliquot of protoplasts before protoplasts were washed and resuspended in 300 *μ*L of washing buffer (154 mm NaCl, 125 mm, CaCl_2_, 5 mm KCl, 2 mm MES; pH5.6). Following incubation at 24 °C with 70 *µ*mol/m^2^/s constant light, the transformation efficiency of each batch was confirmed to be >70% in the control aliquot by quantification of YFP fluorescence in the nuclei using an inverted fluorescence microscope (Zeiss Axio Observer Z1 or ThermoFisher Evos). Aliquots transfected with TF- GR plasmids were treated with 20 mm KNO3 and 20 mm NH_4_NO_3_ for 2 h. Following this, either 10 *μ*M dexamethasone (in 100% EtOH) for 3 h; 35 *μ*M cycloheximide (in DMSO) for 20 min; 10 *μ*M dexamethasone and 35 *μ*M cycloheximide applied sequentially; or no treatment, were added to 3 aliquots of protoplasts. When a treatment was not given, equivalent volumes of EtOH or DMSO were added. After 3 h, cells were harvested and RNA was extracted using the Sigma Spectrum total RNA kit and cDNA was synthesized using M-MLV reverse transcriptase (Thermo Fisher). Quantitative reverse-transcription-PCR (qRT-PCR) was performed on a QuantStudio 6 Pro Real-Time PCR System (Applied Biosystems A43182) in 384-well plates. Amplifications were done in 10 *μ*L reactions containing 1× SYBR Green JumpStart Taq ReadyMix (Sigma S4438), 0.2 *μ*m each primer, and 6 ng cDNA template. No template and no reverse-transcriptase controls were also performed. Each amplification was performed in duplicate (technical repeat). A QuantStudio 6 Pro 384-well standard, relative quantification with melt program (comparative Ct with melt) was used with the following parameters: 94 °C for 2 min then 40 cycles of 94 °C for 15 s, 58 °C for 1 min. A melt curve was performed at the end of the run to confirm the specificity of the amplification. Primer sequences are provided in Supplementary Fig. S19. The fold-changes in expression of the target gene relative to the housekeeping gene (*A. thaliana* eEF-1α) after dexamethasone (DEX) or cycloheximide (CHX) treatments of the protoplasts transfected with a plasmid vector expressing a transcription factor (TF) or transfected with an empty vector (EV) were investigated using real-time quantitative PCR. The C_T_ values were imported into a spreadsheet. The mean fold change in expression of the target relative to eEF-1α in protoplasts transfected with TF (TF-trf) in comparison to control conditions (transfection with an empty vector, EV-trf) was calculated using the 2⁻^ΔΔCT^ method, where $\Delta \Delta C_{T}=(C_{T,\mathrm{target}}\text{–}C_{T,\mathrm{eEF}-1\alpha }{)}_{[\mathrm{TF}-\mathrm{trf}]}\text{–}(C_{T,\mathrm{target}}\text{–}C_{T,\mathrm{eEF}-1\alpha }{)}_{\left[\mathrm{EV}-\mathrm{trf}\right]}$.

For tomato, TF coding sequences were either amplified from cDNA using Phusion polymerase (Primer sequences are provided in Supplementary Fig. S19) and cloned into pENTR/D-TOPO (ThermoFisher), or synthesized (Genewiz, South Plainfield, NJ). Sequence-verified clones were used in LR (recombination between attL and attR sites) cloning reactions with the Gateway-enabled pBEACON DESTINATION vector (Bargmann et al. 2013 ), which contains a C-terminal GR- tag. The resulting EXPRESSION clones were sequence verified. Details of plasmids are provided in Supplementary Data Set 2. Protoplast isolation from hairy root cultures (see below for methods of hairy root production) was performed as previously described (Yoo et al. 2007) with the following changes: Two weeks after sub-cloning, protoplasts were isolated from hairy root tissues harvested from 3 Petri dishes using 1.5% cellulase R10, 1.5% cellulase RS, 0.4% macerozyme, and 0.13% pectolyase enzyme solution. Hairy roots were lightly chopped with a sharp razor blade using ∼10 strokes and incubated in the enzyme solution for 4 h at 28 to 30℃, in the dark, with shaking at 85 rpm. After incubation, the protoplast solution was filtered with 70 and 40 *µ*m strainers, and centrifuged at 500 g 5 min at 4℃ once using 12 ml cold W5 solution (2 mm MES, 154 mm NaCl, 125 mm CaCl2, 5 mm KCl, pH 5.7) in 14 ml round bottom culture tube, and a second time using 2 ml W5 in a round bottom microcentrifuge tube. For downstream transfection, hairy root protoplasts are resuspended at a ∼4 to 8×10^5^ cells/ml concentration in transfection buffer (0.4 m mannitol, 15 mm MgCl2, 4 mm MES, pH 5.6). Protoplast transfection, RNA purification, and qRT-PCR were carried out as described for Arabidopsis.

### Transactivation luciferase assays

Promoter regions of *AtARF18*, *AtANAC032*, *AtDREB26*, *AtNIR1*, *AtNLP6,* and *AtNLP7* including ∼1000 bp upstream of the TSS and the 5′ UTR, were amplified and cloned into pUAP4 (Pollak et al. 2019) to create Level 0 standard parts comparable with the Phytobrick standard (Patron et al. 2015). During cloning, internal BsaI sites in *ANAC032_P_* (301 bp upstream of TSS) and *ARF18_P_* (258 bp upstream of TSS) promoters were removed by the introduction of a mutation. These mutations did not disrupt any candidate TFBSs. These Level 0 promoter parts were used in one-step digestion-ligation reactions with the Level 1 Loop pCk1 (Addgene #136695) backbones together with parts containing LucN (pEPYC0CM0133, Addgene #154595), a C-terminal 3×FLAG epitope tag (pICSL50007; Addgene #50308), and 3′ UTR and terminator sequences from *Agrobacterium tumefaciens* nopaline synthase (*AtuNOS*_T_) (pICH41421, Addgene #50339). As a batch calibrator, an equivalent plasmid was constructed with *AtuNOS*_P_ (pEPSW1KN0035) and experiment calibrator plasmids (pEPSW1KN0034 and pEPSW1KN0072) for ratiometric quantification were assembled from *CaMV35s*_P_: tobacco mosaic virus (ΩTMV) (pICH51277, Addgene #50268) + firefly luciferase (LucF) (pEPAS0CM0008, Addgene #154594) + *CaMV35s*_T_ (pICH41414), or *AtuNOS*_P_ (pICH42211, Addgene #50255) + firefly luciferase (LucF) (pEPAS0CM0008, Addgene #154594) + *AtuOCS*_T_ (pICH41432, Addgene #50343). Finally, the coding sequence of each transcription factor and the YFP coding sequence were cloned into pUAP4 in one-step restriction-ligation reactions to create Level 0 parts (with stop codons) that were subsequently assembled into the level 1 Loop acceptor (pCk2; Addgene #136696) in one-step restriction-ligation reactions with a *CaMV35s_P_*-ΩTMV (pICH51277; Addgene #50268) and *CaMV35s_T_* (pICH41414). Details of plasmids are in Supplementary Data Set 2.

Arabidopsis mesophyll protoplasts were prepared as described above. For each promoter, 6 protoplast aliquots were prepared. Three aliquots received three plasmids: *TF*_P_-LucN-*AtuNOS*_T_ + *CaMV35s*_P_-ΩTMV-TF-*CaMV35s*_T_ + *CaMV35s*_P_-LucF-*CaMV35s*_T_ at equimolar ratios totaling 1000 fmol. In the other 3 aliquots, *CaMV35s*_P_-ΩTMV-TF-CaMV35s_T_ was substituted for *CaMV35s*_P_-ΩTMV-YFP-*CaMV35s*_T_. The entire experiment was repeated 3 times and, in every experiment, 3 protoplast aliquots were transfected with a batch calibrator (*CaMV35s*_P_-LucN-*AtuNOS*_T_ + *CaMV35s*_P_-LucF-*CaMV35s*_T_). Full experimental details are provided in Supplementary Fig. S4. Protoplasts were transfected as described above and, after 18 h, collected by centrifugation. To each aliquot, 30 *μ*L lysis buffer protease inhibitor mix (1 × passive lysis buffer (Promega E1941) and 1 × protease inhibitor cocktail (Sigma P9599)) was added. After 15 min on ice, debris was collected by centrifugation, and 30 *μ*L of lysate was added to each well of a 4titude 96-well white polystyrene microplate flat bottom plate (Azenta Life Sciences). The NanoGlo Dual-Luciferase Reporter Assay System (Promega N1610) was used to quantify NanoLuc and LucF luminescence in a CLARIOstar Plus plate reader with each well measured for ten seconds at 3600 gain with one second settling time.

### Tomato transcriptome and gene expression analysis

Seeds were germinated on a nitrogen-free MS medium (Caisson labs, MSP07) supplemented with the appropriate concentration of KNO_3_ at 22 °C in constant light. The plates were placed vertically in growth chambers at 22 °C, 16 h light/8 h dark, and whole roots were sampled 9 days post-germination. Tomato roots infected with competent *R. rhizogenes* not carrying any plasmid are referred to as WT hairy roots (see method for hairy root production for details). WT hairy roots were transferred to nitrogen-free MS medium (Caisson labs, MSP07) supplemented with the appropriate concentration of KNO_3_. Plates were placed horizontally in growth chambers at 22 °C, 16-h light/8-h dark. Newly grown hairy roots were sampled 9 days post-transfer and used for both RNA-seq and qPCR experiments. Total RNA was extracted using the Quick-RNA Plant Miniprep Kit (Zymo Research). First-strand cDNA was synthesized using SuperScript IV Reverse Transcriptase (Thermo Fisher Scientific) with 500 ng of total RNA as a template.

RNA-seq libraries were prepared using the QuantSeq 3′ mRNA-Seq Library Prep Kit (Lexogen) according to the manufacturer's protocol. Libraries were submitted to the UC Davis DNA Technologies Core and sequenced with an Illumina single-end HiSeq 4000 SR100. Four biological replicates and 3 technical replicates were used for each sample. After sequencing, reads were pooled, trimmed, and filtered using Trim Galore! (v0.4.5) (Babraham Bioinformatics—Trim Galore!) with the parameter -a GATCGGAAGAGCACA. (Bray et al. 2016) Trimmed reads were pseudo-aligned to the ITAG4.1 transcriptome (cDNA) using Kallisto (v0.43.1), with the parameters -b 100–single -l 200 -s 30. Raw RNA-seq read counts were filtered to remove genes with zero counts across all samples. Reads were converted to count per million (CPM) using the cpm() function in edgeR. Genes that were consistently expressed at low levels across all samples were removed. Differential gene expression analysis was done using limma in R/Bioconductor, with empirical weights estimated using the voomWithQualityWeights function. Quantile normalization was used to account for different RNA inputs and library sizes. The linear model for each gene was specified as N treatment: log(counts per million) of an individual gene ∼0 + N_concentration. Differentially expressed genes were selected based on a false discovery rate < 0.05. Raw sequence data generated during this study have been deposited in the National Center for Biotechnology Information (NCBI) BioProject database under accession number PRJNA975125.

For qPCR from hairy roots, the same cDNA was used in reactions with the SYBR Green Master Mix kit (Thermo Fisher Scientific). For qPCR from protoplasts, RNA was extracted from root protoplasts incubated in appropriate KNO_3_ concentration (see method for Protoplast Assay Reporting Overall Effects of TFs (PAROT) for details of protoplast isolation). RNA was purified as described above and cDNA was synthesized and amplified using the Luna Universal One-Step RT-qPCR Kit (NEB). Reactions were cycled in a CFX384 Touch Real-Time PCR Detection System (Bio-Rad). Three technical replicates were performed for each of the 3 biological replicates. Expression values were normalized to the reference gene SlEXP (Solyc07g025390) using the ΔCT method. Data are presented as mean ± SD. Primer sequences are provided in Supplementary Fig. S18.

### Clustering and gene ontology (GO) enrichment analysis

Genes differentially expressed between N treatments were clustered in groups with similar expression patterns. The log_2_ CPM median across biological replicates was calculated for each gene, and the expression of each gene was then scaled to the median expression across all samples. Hierarchical clustering was performed with the pheatmap v.1.0.12 R package with the Euclidean distance metric to quantify similarity. Gene Ontology (GO) categories assigned to each relevant cluster are listed in Supplementary Fig. S7. GO enrichment analysis was performed with the GOseq v.1.34.1 R package with correction for gene length bias (Young et al. 2010). The odds ratio for each ontology was calculated with the formula: (number of genes in GO category/number of all genes in input)/(number of genes in GO category/number of genes in all clusters). Enriched ontology terms were selected based on a *P*-value < 0.05 and an odds ratio > 1. GO categories enriched in each cluster are listed in Supplementary Fig. S7.

### Phylogenetic tree construction

Phylogenetic trees were generated as previously described (Kajala et al. 2021). Briefly, representative proteomes were downloaded from Phytozome, Ensembl, or consortia sites. Next, BLASTp (Madden 2003) was used to identify homologous sequences within each proteome. To refine this set of sequences, a multiple sequence alignment was generated with MAFFT v7 (Katoh and Standley 2013), and a draft tree was generated with FastTree (Price et al. 2010). A monophyletic subtree containing the relevant sequences of interest was then selected. For the final trees, MAFFT v7 using L-INS-i strategy was used to generate a multiple sequence alignment. Next, trimal was used with the -gappyout option. To generate a phylogenetic tree using maximum likelihood, RAxML was used with the option -m PROTGAMMAAUTO and 100 bootstraps. Finally, bipartitions with bootstrap values less than 25% were collapsed using TreeCollapserCL4 (http://emmahodcroft.com/TreeCollapseCL.html). The resulting trees were rooted on sequences from the earliest-diverging species represented in the tree. All alignments and trees are provided in Supplementary Figs. S8 and Files S2 to S4.

### Assembly of constructs for CRISPR/Cas9 mutagenesis

All constructs were generated using the Loop assembly toolkit (Pollak et al. 2019) using the methods described in (Dudley et al. 2022). New level 0 DNA parts (e.g. promoters, terminators, coding seqeunces) were either synthesized (Twist Bioscience, San Francisco, CA and Genewiz, South Plainfield, NJ) or amplified by PCR with overhangs containing SapI recognition sites and assembled into pUAP4 (Addgene #136079) to create parts compatible with the Phytobrick standard (Patron et al. 2015). Targets for mutation were selected using CRISPR-P 2.0 (Liu et al. 2017a). Spacer sequences were synthesized (Integrated DNA Technologies, Coralville, IA or Twist Biosciences, San Francisco, CA) and integrated into single guide RNA (sgRNA) Level 1 expression constructs as described in (Dudley et al. 2022). For Arabidopsis, each final construct contained the FASTred (Shimada et al. 2010) visual nondestructive selectable marker cassette (*AtOLE*_P_-OLE:RFP-*AtOLE*_T_) in position 1; a Cas9 expression cassette (*AtYAO*_P_-SpCas9-E9_T_) or (*AtYAO*_P_-SaCas9-*E9*_T_) in a reverse position 2; a tandem arrays of 2 to 4 sgRNA cassettes; and a plant kanamycin resistance cassette (*AtuNOS*_P_-nptII-*AtuOsc*_T_). For tomato, each final construct contained the FASTgreen selection cassette (*AtOLE*_P_-OLE:GFP-*AtOLE*_T_) in position 1; the SpCas9 expression cassette (*AtRPS5A*_P_-SpCas9-E9_T_) in reverse position 2; 2 sgRNA expression cassettes in tandem in positions 3 and 4; and a plant kanamycin resistance cassette (*AtuNOS*_P_-nptII-*AtuOsc*_T_) or basta resistance cassette (*CaMV35s*_P_-Bar-*CaMV35s*_T_) in position 5.

### Production of Arabidopsis lines with CRISPR/Cas9-induced mutations

Assembled CRISPR/Cas9 plasmids were transformed into *A. tumefaciens* GV3101 and liquid cultures were grown from single colonies in LB supplemented with 50 mg/mL rifampicin, 25 mg/mL gentamycin, and 50 mg/mL kanamycin at 28 °C. Cells were collected by centrifugation and resuspended to OD_600_ 0.8 in 5% sucrose, and 0.05% Silwet L-77, and sprayed onto the floral tissues of ten Col0 plants. Plants were sealed in black plastic bags for 24 h. Seeds were collected from mature siliques and surface sterilized with 70% EtOH for 10 min followed by 3% to 5% sodium hypochlorite for 10 min. For the selection of T_1_ transgenic plants, sterilized seeds were germinated and grown on MS supplemented with 75 mg/mL kanamycin at 16 h light 22 °C. T_1_ seedlings were transferred to soil and T_2_ seed was collected. Seed batches were examined with a fluorescence microscope to assess the segregation of transgene by expression from the FAST-red marker cassette. Non-fluorescent T_2_ seeds from segregating seed batches were selected and germinated. DNA was extracted, and PCR and sequencing were performed to confirm the absence of the transgene and to identify mutations at the target loci. The genotypes of all lines analyzed in this study are provided in Supplementary Figs. S10 and S11. T_3_ progeny of each line were grown to bulk seed to confirm the stability of the genotype, and T_4_ seeds were used for subsequent analysis.

### Production of *Rhizobium rhizogenes*-induced tomato hairy roots with CRISPR/Cas9 mutations

Tomato hairy root transformation was performed as previously described (Ron et al. 2014). Briefly, competent *R. rhizogenes* was transformed by electroporation with each CRISPR/Cas9 construct, plated on nutrient agar (Difco, BD 247940) with the appropriate antibiotics, and incubated for 2 to 3 days at 28 to 30℃. Single colonies were selected and used to inoculate liquid cultures that were grown overnight at 30℃ with shaking. The resulting cultures were used to transform cotyledons. For each construct, 40 fully expanded cotyledons were cut and dipped into liquid *R. rhizogenes* culture and co-cultivated in the dark on MS agar (MS basal media pH 5.8 with 1X vitamins, 3% w/v sucrose, 1% w/v agar) without antibiotic selection for 3 days. Cotyledons were then transferred to selective MS agar (MS basal media pH 5.8 with 1× vitamins, 3% w/v sucrose, 1% w/v agar, 200 mg/L cefotaxime, 50 mg/L kanamycin). At least 15 independent antibiotic-resistant roots were subcloned for each transformation for genotyping and subsequent analyses. Generally, roots were subcloned on selective MS agar for 2 rounds before maintenance on MS agar with Cefotaxime (MS basal media pH 5.8 with 1X vitamins, 1% w/v sucrose, 1% w/v agar, and 200 mg/L cefotaxime).

For genotyping, samples of root tissues were collected aseptically, and genomic DNA was purified. Target regions were amplified, and adapters were ligated to produce barcoded pools of amplicon libraries. Libraries were normalized, pooled, and sequenced using Illumina chemistry (MGH CCIB DNA Core Facility, Massachusetts General Hospital, Boston). Sequencing reads were filtered, de-multiplexed, mapped to reference sequences using a customized script, and visualized in an Integrative Genomics Viewer genome browser (https://igv.org) to identify mutations. At least 2 independent lines, each with a different mutation, were selected from each transformation. The genotypes of all lines are provided in Supplementary Fig. S11.

### Production of stable tomato lines with CRISPR/Cas9-induced mutations

CRISPR/Cas9 constructs (as noted above) were introduced in *Agrobacterium tumefaciens* and transformed into *Solanum lycopersicum* cultivar M82 (LA3475) at the UC Davis Transformation Facility. For each construct, 12 to 20 first-generation (T0) seedlings were transferred to a potting mix and cultivated in a growth chamber at 22 °C with 16-h light/8-h dark cycles for 14 days before transfer to the greenhouse. Genomic DNA was purified from leaf issues and target regions were amplified and sequenced as described above. T1 seeds were collected from lines in which mutations at the target were identified and the resulting plants were genotyped to identify lines with homozygous or biallelic mutations at target sites in which the T-DNA had segregated away. The genotypes of all lines are provided in Supplementary Fig. S11. Plants were self-pollinated, and progeny seeds were collected for further analysis.

### Root system architecture (RSA) analysis

Arabidopsis seeds were sieved to obtain seeds between 250 and 300 *µ*m, sterilized with 50% (v/v) bleach, and stored at 4 °C for 48 h before germination on sterile growth media supplemented with the appropriate concentration of KNO_3_ as previously described (Gaudinier et al. 2018). Arabidopsis mutants were grown in triplicate with Col-0 controls on the same plate. Nine seeds were placed on each plate. The plates were placed vertically in 22 °C growth chambers with 16-h light/8-h dark cycles for 9 days post germination before the plates were scanned and images processed using SmartRoot (Lobet et al. 2011). Tomato seeds were germinated on a nitrogen-free MS medium (Caisson labs, MSP07) supplemented with Phytagel (Sigma-Aldrich, P8169) and the appropriate amount of KNO_3_ to mimic different nitrogen treatments. Tomato mutant seeds and their M82 control were grown in triplicate separately with 5 seeds placed on each plate. The plates were placed vertically in 22 °C growth chambers with 16-h light/8-h dark cycles. Roots were scanned every day for 14 days after the first germination at 800 dpi with an Epson Perfection V700 scanner. Images were processed in Fiji software. For tomato, RSA traits at 10 days after germination (DAG) were measured. For both species, RSA traits including primary root length, total lateral root number, total lateral root length, average lateral root length, total root length (primary root length + total lateral root length), lateral root density (total number of lateral roots divided by primary root length), and the ratio of total lateral root length to total root length were quantified.

All statistical analyses were performed in R version i386 4.3.0 For RSA assays, 2 statistical tests were performed: unpaired *t*-tests between wild-type and mutant seedlings for each treatment combination as well as a two-way ANOVA to test for genotype × treatment interactions. Statistical tests were performed on the natural log of a given trait for each individual. After log transformation, outliers that were more than 2 standard deviations away from the mean within a genotype-treatment group were removed. To test for wild-type response to nitrogen treatment, a one-way ANOVA was performed using the car package's ANOVA function (anova(lm(log.TraitValue ∼ Treatment + Plate))). To test for significant genotype by treatment interactions, a two-way ANOVA was performed (anova(lm(log.TraitValue ∼ Genotype*Treatment + Plate))). In the case of the tomato RSA analysis, “Plate” and “Genotype” were highly correlated, so the factor “Plate” was set to zero”. Additional t-tests for each pairwise treatment combination were also performed. To test for genotype-specific responses to nitrogen treatment, the natural log of a trait was modeled using a two-way ANOVA (anova(log.TraitValue ∼ Genotype*Treatment + Plate)). For the tomato data, the two-way ANOVA was run as anova(llog.TraitValue ∼ Genotype*Treatment). Please see Supplementary File 1 for all calculated *P*-values.

### Assembly of nitrate-responsive reporter constructs

A promoter fragment containing 1 kb upstream of the start codon of *AtNIR1*_P_ was amplified from genomic DNA isolated from Arabidopsis Col-0 seedlings. In addition, 365 bps of a previously described synthetic Arabidopsis nitrogen-responsive promoter (*AtNRP*_P_) (Wang et al. 2010) was synthesized (Twist Bioscience, San Francisco, CA). An optimized minimal synthetic promoter containing 4 copies of the NRE motifs (4×NRE), truncated to only include NLP7 TFBSs, with 21 bp of random spacer sequence with equal ATCG ratios downstream of each binding site followed by TATATAA fused to a minimal 35-s promoter was synthesized. Promoter fragments were cloned into pENTR/5′-TOPO (ThermoFisher), and the resulting ENTRY clones were used in a LR Clonase II reaction with the binary DESTINATION vector pMR099, which contains the coding sequence of nuclear-targeted GFP (GFP_nu_) and a cassette for constitutive expression of plasma membrane-targeted tagRFP (CaMV35s_P_-TagRFP:LTI6b) (Ron et al. 2014). The resulting expression clones encode *AtNIR*_P_-GFP_nu_ and *AtNRP*_P_-GFP_nu_. Similarly, *AtNRP*_P_ was assembled with the emerald luciferase (ELuc) coding sequence to produce an AtNRP_P_-Eluc-Hsp18-2 expression vector.

### Confocal imaging

All confocal images were captured using a Zeiss Observer Z1 LSM700 (Zeiss) Confocal Laser Scanning Microscopy (water immersion, × 20 objective) with excitation at 488 nm and emission at 493 to 550 nm for GFP and excitation at 555 nm and emission at 560 to 800 nm for mRFP. The tomato seeds or hairy roots were germinated/transferred to MS medium with different KNO_3_ concentrations for 10 days, and root tips of tomato seedlings or hairy roots were collected for imaging. Cell outlines were visualized using a constitutive mRFP expression cassette in the pMR099 vector. Confocal images were false-colored, and brightness/contrast was adjusted in Fiji software.

### Protoplast assay reporting overall effects of TFs (PAROT)

In this assay, vectors encoding a nitrate-responsive green luciferase (AtNRP_P_-Eluc-Hsp18-2) and constitutively expressed red luciferase (pNOS-Rluc-tNOS; pGREAT27, Addgene #170915) are delivered to root protoplasts. To obtain Arabidopsis root tissues, seeds were sterilized with 50% (v/v) bleach and vernalized for 3 days before plating on MS media (10 g agar, 0.5 g MES, 10 g sucrose, 4.33 g MS salts, pH to 5.7 with KOH). Around 800 seeds were densely placed in 2 rows on 120-mm square plates with a Nitex mesh (Sefar) to facilitate the recovery of roots from plates for protoplasting. The plates were placed vertically in growth chambers at 22 °C with 16-h light/8-h dark cycles for 8 to 10 days before harvesting. Protoplasts were isolated as previously described (Birnbaum et al. 2005) with modifications. Briefly, 20 mg pectolyase (Sigma-Aldrich P-5936) and 230 mg cellulase RS (Yakult) were dissolved in 15 mL of protoplast isolation buffer (600 mm mannitol, 20 mm MES, 20 mm KCl, 0.1% BSA, 10 mm CaCl_2_, pH 5.7) and passed through a 0.45-*μ*m filter. Root tissues were harvested from 6 plates, chopped several times, and placed within a 70 *μ*m strainer set within a 6-well petri dish containing 5 ml of the enzyme solution. After 3 h at room temperature (or 25˚C) on an orbital shaker, the solution was passed through a 40-*μ*m cell strainer to remove any remaining tissues. Protoplasts were collected and washed in W5 buffer (2 mm MES, 154 mm NaCl, 125 mm CaCl_2_, 5 mm KCl, pH 5.7) by centrifugation at 4 °C 500 g for 10 min with acceleration and deceleration set to zero. Tomato root protoplasts were prepared as described in the modified TARGET assay section.

Protoplasts of both species were counted with a hemocytometer and diluted in transfection buffer (4 mm MES, 0.4 m mannitol, 15 mm MgCl_2_, pH 5.7) at a concentration of 2 to 8×10^5^ cells/ml for transfection with dual luciferase vectors. Fresh 40% PEG transfection solution (4 g of PEG4000 (Fluka, cat. no. 81240) added into 3 mL of H_2_O, 2.5 mL of 0.8 m mannitol, and 1 mL of 1 m CaCl_2_) was prepared at least one hour before transfection to allow enough time to dissolve. In a round bottom microcentrifuge tube, 10 *μ*g of each plasmid was mixed with 100 *μ*L protoplasts and 110 *μ*L PEG solution and incubated for 15 min. Protoplasts were again washed with W5 after transfection and suspended in WI buffer (4 mm MES, 0.5 m mannitol, 20 mm KCl, pH 5.7) with appropriate KNO_3_ concentration (0, 1, or 10 mm) for 24 h before quantification of luciferase in a plate reader, with each well measured for ten seconds at 3600 gain with one second settling time.

The viability of protoplasts was monitored by staining with an equal volume of 50 *μ*g/mL fluorescein diacetate (FDA). Protoplasts were gently mixed by pipetting and immediately imaged using an EVOS fluorescence microscope (ThermoFisher) with an excitation at 470/22 nm and emission at 525/50 nm. Viability was assessed before transfection, immediately after transfection, and after 24 h (Supplementary Fig. S16).

## Supplementary Material

koaf124_Supplementary_Data

## Data Availability

Raw sequence data generated during this study have been deposited in the National Center for Biotechnology Information (NCBI) BioProject database under accession number PRJNA975125.

## Supplementary data

The following materials are available in the online version of this article.

**Supplementary Figure S1.** Logoplots of position weight matrixes are used to identify candidate transcription factor-binding sites.

**Supplementary Figure S2.** Alignments of the DNA-binding domains of Arabidopsis transcription factors.

**Supplementary Figure S3.** In vitro binding assays showing relative binding of transcription factors to target gene-derived probes.

**Supplementary Figure S4.** Methodology for protoplast transactivation assays.

**Supplementary Figure S5.** Expression levels of Arabidopsis genes in modified TARGET assays.

**Supplementary Figure S6.** Luciferase transactivation assays.

**Supplementary Figure S7.** Nitrate-responsive genes in tomato roots.

**Supplementary Figure S8.** Phylogenetic analysis of transcription factors.

**Supplementary Figure S9.** Domain analysis of tomato orthologs of AtNLP7.

**Supplementary Figure S10.** Table of Arabidopsis and tomato mutant lines.

**Supplementary Figure S11.** Genotypes of Arabidopsis and tomato CRISPR lines.

**Supplementary Figure S12.** Analysis of the root system architecture of Arabidopsis and tomato mutant alleles of *NLP7, ANAC032,* and *ARF18*.

**Supplementary Figure S13.** Alignments of the DNA-binding domains of tomato and Arabidopsis transcription factors.

**Supplementary Figure S14.** Characterization of N responses in hairy roots

**Supplementary Figure S15.** Expression levels of tomato genes in modified TARGET assays.

**Supplementary Figure S16.** Exemplification of the PAROT assay.

**Supplementary Figure S17.** PAROT assay in Arabidopsis and tomato mutants.

**Supplementary Figure S18.** Summary of all evidence for regulatory sub-networks.

**Supplementary Figure S19.** Primers used in this study.

**Supplementary Data Set 1.** Sequences of DNA probes used in transcription factor–DNA-binding assays.

**Supplementary Data Set 2.** Plasmids used in this study.

**Supplementary File 1.** Statistics for the root system architecture analysis

**Supplementary File 2.** Alignment files that support Supplementary Fig. S2.

**Supplementary File 3.** Alignment files and phylogenetic trees that support Supplementary Figs. S8 and S9.

**Supplementary File 4.** Alignment files that support Supplementary Fig. S13.

## Dive Curated Terms

The following phenotypic, genotypic, and functional terms are of significance to the work described in this paper:

ARF9 Gramene: AT4G23980
ARF9 Araport: AT4G23980

NIR1 Gramene: AT2G15620
NIR1 Araport: AT2G15620

ARF18 Gramene: AT3G61830
ARF18 Araport: AT3G61830

AtRPS5A Gramene: AT3G11940
AtRPS5A Araport: AT3G11940

MES CHEBI: CHEBI:39010

NLP7 Gramene: AT4G24020
NLP7 Araport: AT4G24020

PCR Gramene: Polymerase Chain Reaction
PCR Araport: Polymerase Chain Reaction

root AmiGo: PO:0009005

NCBI Gramene: https://www.ncbi.nlm.nih.gov
NCBI Araport: https://www.ncbi.nlm.nih.gov

TAIR Gramene: https://www.arabidopsis.org/
TAIR Araport: https://www.arabidopsis.org/

DREB26 Gramene: At1G21910
DREB26 Araport: At1G21910

## References

[koaf124-B3] Alon U. An introduction to systems biology: design principles of biological circuits . 2nd ed. Boca Raton (FL): CRC Press (Taylor & Francis Group).; 2018. 10.1201/9780429283321

[koaf124-B1] Alvarez JM, Riveras E, Vidal EA, Gras DE, Contreras-López O, Tamayo KP, Aceituno F, Gómez I, Ruffel S, Lejay L, et al Systems approach identifies TGA1 and TGA4 transcription factors as important regulatory components of the nitrate response of *Arabidopsis thaliana* roots. Plant J. 2014:80(1):1–13. 10.1111/tpj.12618 25039575

[koaf124-B2] Alvarez JM, Schinke A-L, Brooks MD, Pasquino A, Leonelli L, Varala K, Safi A, Krouk G, Krapp A, Coruzzi GM. Transient genome-wide interactions of the master transcription factor NLP7 initiate a rapid nitrogen-response cascade. Nat Commun. 2020:11(1):1157. 10.1038/s41467-020-14979-6 32123177 PMC7052136

[koaf124-B4] Araya T, Miyamoto M, Wibowo J, Suzuki A, Kojima S, Tsuchiya YN, Sawa S, Fukuda H, von Wirén N, Takahashi H. CLE-CLAVATA1 peptide-receptor signaling module regulates the expansion of plant root systems in a nitrogen-dependent manner. Proc Natl Acad Sci U S A. 2014:111(5):2029–2034. 10.1073/pnas.1319953111 24449877 PMC3918772

[koaf124-B5] Asins MJ, Albacete A, Martinez-Andujar C, Pérez-Alfocea F, Dodd IC, Carbonell EA, Dieleman JA. Genetic analysis of rootstock-mediated nitrogen (N) uptake and root-to-shoot signalling at contrasting N availabilities in tomato. Plant Sci. 2017:263:94–106. 10.1016/j.plantsci.2017.06.012 28818388

[koaf124-B6] Atkinson MR, Savageau MA, Myers JT, Ninfa AJ. Development of genetic circuitry exhibiting toggle switch or oscillatory behavior in *Escherichia coli*. Cell. 2003:113(5):597–607. 10.1016/S0092-8674(03)00346-5 12787501

[koaf124-B7] Babraham Bioinformatics—Trim Galore! [accessed 2024 Dec 9]. https://www.bioinformatics.babraham.ac.uk/projects/trim_galore/.

[koaf124-B8] Bailey TL, Johnson J, Grant CE, Noble WS. The MEME suite. Nucleic Acids Res. 2015:43(W1):W39–W49. 10.1093/nar/gkv416 25953851 PMC4489269

[koaf124-B9] Bargmann BOR, Marshall-Colon A, Efroni I, Ruffel S, Birnbaum KD, Coruzzi GM, Krouk G. TARGET: a transient transformation system for genome-wide transcription factor target discovery. Mol Plant. 2013:6(3):978–980. 10.1093/mp/sst010 23335732 PMC3660954

[koaf124-B10] Birnbaum K, Jung JW, Wang JY, Lambert GM, Hirst JA, Galbraith DW, Benfey PN. Cell type-specific expression profiling in plants via cell sorting of protoplasts from fluorescent reporter lines. Nat Methods. 2005:2(8):615–619. 10.1038/nmeth0805-615 16170893

[koaf124-B11] Bray NL, Pimentel H, Melsted P, Pachter L. Near-optimal probabilistic RNA-seq quantification. Nat Biotechnol. 2016:34(5):525–527. 10.1038/nbt.3519 27043002

[koaf124-B12] Brooks MD, Cirrone J, Pasquino AV, Alvarez JM, Swift J, Mittal S, Juang C-L, Varala K, Gutiérrez RA, Krouk G, et al Network walking charts transcriptional dynamics of nitrogen signaling by integrating validated and predicted genome-wide interactions. Nat Commun. 2019:10(1):1569. 10.1038/s41467-019-09522-1 30952851 PMC6451032

[koaf124-B13] Brooks MD, Juang C-L, Katari MS, Alvarez JM, Pasquino A, Shih H-J, Huang J, Shanks C, Cirrone J, Coruzzi GM. ConnecTF: a platform to integrate transcription factor-gene interactions and validate regulatory networks. Plant Physiol. 2021:185(1):49–66. 10.1093/plphys/kiaa012 33631799 PMC8133578

[koaf124-B14] Brunoud G, Wells DM, Oliva M, Larrieu A, Mirabet V, Burrow AH, Beeckman T, Kepinski S, Traas J, Bennett MJ, et al A novel sensor to map auxin response and distribution at high spatio-temporal resolution. Nature. 2012:482(7383):103–106. 10.1038/nature10791 22246322

[koaf124-B15] Cai Y-M, Kallam K, Tidd H, Gendarini G, Salzman A, Patron NJ. Rational design of minimal synthetic promoters for plants. Nucleic Acids Res. 2020:48(21):11845–11856. 10.1093/nar/gkaa682 32856047 PMC7708054

[koaf124-B16] Cai Y-M, Witham S, Patron NJ. Tuning plant promoters using a simple split luciferase method to assess transcription factor-DNA interactions. ACS Synth Biol. 2023:12(11):3482–3486. 10.1021/acssynbio.3c00094 37856867 PMC10661027

[koaf124-B17] Cancé C, Martin-Arevalillo R, Boubekeur K, Dumas R. Auxin response factors are keys to the many auxin doors. New Phytol. 2022:235(2):402–419. 10.1111/nph.18159 35434800

[koaf124-B18] Cao H, Qi S, Sun M, Li Z, Yang Y, Crawford NM, Wang Y. Overexpression of the maize ZmNLP6 and ZmNLP8 can complement the Arabidopsis nitrate regulatory mutant nlp7 by restoring nitrate signaling and assimilation. Front Plant Sci. 2017:8:1703. 10.3389/fpls.2017.01703 29051766 PMC5634353

[koaf124-B19] Castaings L, Camargo A, Pocholle D, Gaudon V, Texier Y, Boutet-Mercey S, Taconnat L, Renou J-P, Daniel-Vedele F, Fernandez E, et al The nodule inception-like protein 7 modulates nitrate sensing and metabolism in Arabidopsis. Plant J. 2009:57(3):426–435. 10.1111/j.1365-313X.2008.03695.x 18826430

[koaf124-B20] Chapin FS 3rd, Walter CH, Clarkson DT. Growth response of barley and tomato to nitrogen stress and its control by abscisic acid, water relations and photosynthesis. Planta. 1988:173(3):352–366. 10.1007/BF00401022 24226542

[koaf124-B21] Cheng C-Y, Li Y, Varala K, Bubert J, Huang J, Kim GJ, Halim J, Arp J, Shih H-JS, Levinson G, et al Evolutionarily informed machine learning enhances the power of predictive gene-to-phenotype relationships. Nat Commun. 2021:12(1):5627. 10.1038/s41467-021-25893-w 34561450 PMC8463701

[koaf124-B22] Cheng Y-H, Durand M, Brehaut V, Hsu F-C, Kelemen Z, Texier Y, Krapp A, Tsay Y-F. Interplay between NIN-LIKE PROTEINs 6 and 7 in nitrate signaling. Plant Physiol. 2023:192(4):3049–3068. 10.1093/plphys/kiad242 37073492

[koaf124-B23] Contreras-López O, Vidal EA, Riveras E, Alvarez JM, Moyano TC, Sparks EE, Medina J, Pasquino A, Benfey PN, Coruzzi GM, et al Spatiotemporal analysis identifies ABF2 and ABF3 as key hubs of endodermal response to nitrate. Proc Natl Acad Sci U S A. 2022:119(4):e2107879119. 10.1073/pnas.2107879119 35046022 PMC8794810

[koaf124-B24] Costa-Broseta Á, Castillo M, León J. Nitrite reductase 1 is a target of nitric oxide-mediated post-translational modifications and controls nitrogen flux and growth in Arabidopsis. Int J Mol Sci. 2020:21(19):7270. 10.3390/ijms21197270 33019636 PMC7582248

[koaf124-B25] Dickinson PJ, Kneřová J, Szecówka M, Stevenson SR, Burgess SJ, Mulvey H, Bågman A-M, Gaudinier A, Brady SM, Hibberd JM. A bipartite transcription factor module controlling expression in the bundle sheath of Arabidopsis thaliana. Nat Plants. 2020:6(12):1468–1479. 10.1038/s41477-020-00805-w 33230313

[koaf124-B26] Doidy J, Li Y, Neymotin B, Edwards MB, Varala K, Gresham D, Coruzzi GM. “Hit-and-run” transcription: de novo transcription initiated by a transient bZIP1 “hit” persists after the “run”. BMC Genomics. 2016:17(1):92. 10.1186/s12864-016-2410-2 26843062 PMC4738784

[koaf124-B27] Dudley QM, Raitskin O, Patron NJ. Cas9-mediated targeted mutagenesis in plants. Methods Mol Biol. 2022:2379:1–26. 10.1007/978-1-0716-1791-5_1 35188653

[koaf124-B28] Errebhi M, Wilcox GE. Tomato growth and nutrient uptake pattern as influenced by nitrogen form ratio. J Plant Nutr. 1990:13(8):1031–1043. 10.1080/01904169009364133

[koaf124-B29] Freire-Rios A, Tanaka K, Crespo I, van der Wijk E, Sizentsova Y, Levitsky V, Lindhoud S, Fontana M, Hohlbein J, Boer DR, et al Architecture of DNA elements mediating ARF transcription factor binding and auxin-responsive gene expression in *Arabidopsis*. Proc Natl Acad Sci U S A. 2020:117(39):24557–24566. 10.1073/pnas.2009554117 32929017 PMC7533888

[koaf124-B30] Gaudinier A, Rodriguez-Medina J, Zhang L, Olson A, Liseron-Monfils C, Bågman A-M, Foret J, Abbitt S, Tang M, Li B, et al Transcriptional regulation of nitrogen-associated metabolism and growth. Nature. 2018:563(7730):259–264. 10.1038/s41586-018-0656-3 30356219

[koaf124-B31] Gifford ML, Dean A, Gutierrez RA, Coruzzi GM, Birnbaum KD. Cell-specific nitrogen responses mediate developmental plasticity. Proc Natl Acad Sci U S A. 2008:105(2):803–808. 10.1073/pnas.0709559105 18180456 PMC2206617

[koaf124-B32] Granger BE, Pérez F. Jupyter: thinking and storytelling with code and data. Comput Sci Eng. 2021:23(2):7–14. 10.1109/MCSE.2021.305926335939280

[koaf124-B33] Grant CE, Bailey TL, Noble WS. FIMO: scanning for occurrences of a given motif. Bioinformatics. 2011:27(7):1017–1018. 10.1093/bioinformatics/btr064 21330290 PMC3065696

[koaf124-B34] Guan P, Wang R, Nacry P, Breton G, Kay SA, Pruneda-Paz JL, Davani A, Crawford NM. Nitrate foraging by Arabidopsis roots is mediated by the transcription factor TCP20 through the systemic signaling pathway. Proc Natl Acad Sci U S A. 2014:111(42):15267–15272. 10.1073/pnas.1411375111 25288754 PMC4210337

[koaf124-B35] Heerah S, Katari M, Penjor R, Coruzzi G, Marshall-Colon A. WRKY1 mediates transcriptional regulation of light and nitrogen signaling pathways. Plant Physiol. 2019:181(3):1371–1388. 10.1104/pp.19.00685 31409699 PMC6836853

[koaf124-B36] Hummel NFC, Zhou A, Li B, Markel K, Ornelas IJ, Shih PM. The trans-regulatory landscape of gene networks in plants. Cell Syst. 2023:14(6):501–511.e4. 10.1016/j.cels.2023.05.002 37348464

[koaf124-B37] Ideker T, Galitski T, Hood L. A new approach to decoding life: systems biology. Annu Rev Genomics Hum Genet. 2001:2(1):343–372. 10.1146/annurev.genom.2.1.343 11701654

[koaf124-B38] Julian RS , Patrick RM, Li Y. Organ-specific characteristics govern the relationship between histone code dynamics and transcriptional reprogramming during nitrogen response in tomato. Commun Biol. 2023:6(1):1225. 10.1038/s42003-023-05601-8 PMC10694154 38044380

[koaf124-B39] Kajala K, Gouran M, Shaar-Moshe L, Mason GA, Rodriguez-Medina J, Kawa D, Pauluzzi G, Reynoso M, Canto-Pastor A, Manzano C, et al Innovation, conservation, and repurposing of gene function in root cell type development. Cell. 2021:184(12):3333–3348. 10.1016/j.cell.2021.04.024 34010619

[koaf124-B40] Katoh K, Standley DM. MAFFT multiple sequence alignment software version 7: improvements in performance and usability. Mol Biol Evol. 2013:30(4):772–780. 10.1093/molbev/mst010 23329690 PMC3603318

[koaf124-B41] Konishi M, Okitsu T, Yanagisawa S. Nitrate-responsive NIN-like protein transcription factors perform unique and redundant roles in Arabidopsis. J Exp Bot. 2021:72(15):5735–5750. 10.1093/jxb/erab246 34050740

[koaf124-B42] Konishi M, Yanagisawa S. Identification of a nitrate-responsive cis-element in the Arabidopsis NIR1 promoter defines the presence of multiple cis-regulatory elements for nitrogen response. Plant J. 2010:63(2):269–282. 10.1111/j.1365-313X.2010.04239.x 20444232

[koaf124-B43] Krouk G, Lacombe B, Bielach A, Perrine-Walker F, Malinska K, Mounier E, Hoyerova K, Tillard P, Leon S, Ljung K, et al Nitrate-regulated auxin transport by NRT1.1 defines a mechanism for nutrient sensing in plants. Dev Cell. 2010a:18(6):927–937. 10.1016/j.devcel.2010.05.008 20627075

[koaf124-B44] Krouk G, Mirowski P, LeCun Y, Shasha DE, Coruzzi GM. Predictive network modeling of the high-resolution dynamic plant transcriptome in response to nitrate. Genome Biol. 2010b:11(12):R123. 10.1186/gb-2010-11-12-r123 21182762 PMC3046483

[koaf124-B45] Lamesch P, Berardini TZ, Li D, Swarbreck D, Wilks C, Sasidharan R, Muller R, Dreher K, Alexander DL, Garcia-Hernandez M, et al The Arabidopsis information resource (TAIR): improved gene annotation and new tools. Nucleic Acids Res. 2012:40(D1):D1202–D1210. 10.1093/nar/gkr1090 22140109 PMC3245047

[koaf124-B46] Lee TI, Rinaldi NJ, Robert F, Odom DT, Bar-Joseph Z, Gerber GK, Hannett NM, Harbison CT, Thompson CM, Simon I, et al Transcriptional regulatory networks in Saccharomyces cerevisiae. Science. 2002:298(5594):799–804. 10.1126/science.1075090 12399584

[koaf124-B47] Liu H, Ding Y, Zhou Y, Jin W, Xie K, Chen L-L. CRISPR-P 2.0: an improved CRISPR-Cas9 tool for genome editing in plants. Mol Plant. 2017a:10(3):530–532. 10.1016/j.molp.2017.01.003 28089950

[koaf124-B48] Liu K-H, Liu M, Lin Z, Wang Z-F, Chen B, Liu C, Guo A, Konishi M, Yanagisawa S, Wagner G, et al NIN-like protein 7 transcription factor is a plant nitrate sensor. Science. 2022:377(6613):1419–1425. 10.1126/science.add1104 36137053 PMC9628810

[koaf124-B49] Liu K-H, Niu Y, Konishi M, Wu Y, Du H, Sun Chung H, Li L, Boudsocq M, McCormack M, Maekawa S, et al Discovery of nitrate-CPK-NLP signalling in central nutrient-growth networks. Nature. 2017b:545(7654):311–316. 10.1038/nature22077 28489820 PMC5823009

[koaf124-B50] Liu L, Gallagher J, Arevalo ED, Chen R, Skopelitis T, Wu Q, Bartlett M, Jackson D. Enhancing grain-yield-related traits by CRISPR–Cas9 promoter editing of maize CLE genes. Nat Plants. 2021a:7(3):287–294. 10.1038/s41477-021-00858-5 33619356

[koaf124-B51] Liu M, Zhi X, Wang Y, Wang Y. Genome-wide survey and expression analysis of NIN-like protein (NLP) genes reveals its potential roles in the response to nitrate signaling in tomato. BMC Plant Biol. 2021b:21(1):347. 10.1186/s12870-021-03116-0 34301191 PMC8299697

[koaf124-B52] Lobet G, Pagès L, Draye X. A novel image-analysis toolbox enabling quantitative analysis of root system architecture. Plant Physiol. 2011:157(1):29–39. 10.1104/pp.111.179895 21771915 PMC3165877

[koaf124-B53] Ma W, Li J, Qu B, He X, Zhao X, Li B, Fu X, Tong Y. Auxin biosynthetic gene TAR2 is involved in low nitrogen-mediated reprogramming of root architecture in Arabidopsis. Plant J. 2014:78(1):70–79. 10.1111/tpj.12448 24460551

[koaf124-B54] Machado J, Vasconcelos MW, Soares C, Fidalgo F, Heuvelink E, Carvalho SMP. Young tomato plants respond differently under single or combined mild nitrogen and water deficit: an insight into morphophysiological responses and primary metabolism. Plants. 2023:12(5):1181. 10.3390/plants12051181 36904041 PMC10005627

[koaf124-B55] Madden T . The BLAST sequence analysis tool. The NCBI Handbook. 2003:2(5):425–436.

[koaf124-B56] Maher KA, Bajic M, Kajala K, Reynoso M, Pauluzzi G, West DA, Zumstein K, Woodhouse M, Bubb K, Dorrity MW, et al Profiling of accessible chromatin regions across multiple plant species and cell types reveals common gene regulatory principles and new control modules. Plant Cell. 2018:30(1):15–36. 10.1105/tpc.17.00581 29229750 PMC5810565

[koaf124-B57] Mangan S, Alon U. Structure and function of the feed-forward loop network motif. Proc Natl Acad Sci U S A. 2003:100(21):11980–11985. 10.1073/pnas.2133841100 14530388 PMC218699

[koaf124-B58] Marchive C, Roudier F, Castaings L, Bréhaut V, Blondet E, Colot V, Meyer C, Krapp A. Nuclear retention of the transcription factor NLP7 orchestrates the early response to nitrate in plants. Nat Commun. 2013:4(1):1713. 10.1038/ncomms2650 23591880

[koaf124-B59] Medici A, Marshall-Colon A, Ronzier E, Szponarski W, Wang R, Gojon A, Crawford NM, Ruffel S, Coruzzi GM, Krouk G. AtNIGT1/HRS1 integrates nitrate and phosphate signals at the Arabidopsis root tip. Nat Commun. 2015:6(1):6274. 10.1038/ncomms7274 25723764 PMC4373655

[koaf124-B60] Milo R, Shen-Orr S, Ltzkovitz S, Kashtan N, Chktovskii D, Alan U. Network motifs: simple building blocks of complex networks. The structure and dynamics of networks. Princeton: Princeton University Press. 2011. p. 217–220.

[koaf124-B61] North KA, Ehlting B, Koprivova A, Rennenberg H, Kopriva S. Natural variation in Arabidopsis adaptation to growth at low nitrogen conditions. Plant Physiol Biochem. 2009:47(10):912–918. 10.1016/j.plaphy.2009.06.009 19628403

[koaf124-B62] O’Malley RC, Huang S-SC, Song L, Lewsey MG, Bartlett A, Nery JR, Galli M, Gallavotti A, Ecker JR. Cistrome and epicistrome features shape the regulatory DNA landscape. Cell. 2016:165(5):1280–1292. 10.1016/j.cell.2016.04.038 27203113 PMC4907330

[koaf124-B63] Para A, Li Y, Marshall-Colón A, Varala K, Francoeur NJ, Moran TM, Edwards MB, Hackley C, Bargmann BOR, Birnbaum KD, et al Hit-and-run transcriptional control by bZIP1 mediates rapid nutrient signaling in Arabidopsis. Proc Natl Acad Sci U S A. 2014:111(28):10371–10376. 10.1073/pnas.1404657111 24958886 PMC4104873

[koaf124-B64] Patron NJ, Orzaez D, Marillonnet S, Warzecha H, Matthewman C, Youles M, Raitskin O, Leveau A, Farré G, Rogers C, et al Standards for plant synthetic biology: a common syntax for exchange of DNA parts. New Phytol. 2015:208(1):13–19. 10.1111/nph.13532 26171760

[koaf124-B65] Pollak B, Cerda A, Delmans M, Álamos S, Moyano T, West A, Gutiérrez RA, Patron NJ, Federici F, Haseloff J. Loop assembly: a simple and open system for recursive fabrication of DNA circuits. New Phytol. 2019:222(1):628–640. 10.1111/nph.15625 30521109

[koaf124-B66] Potter KC, Wang J, Schaller GE, Kieber JJ. Cytokinin modulates context-dependent chromatin accessibility through the type-B response regulators. Nat Plants. 2018:4(12):1102–1111. 10.1038/s41477-018-0290-y 30420712

[koaf124-B67] Price MN, Dehal PS, Arkin AP. FastTree 2–approximately maximum-likelihood trees for large alignments. PLoS One. 2010:5(3):e9490. 10.1371/journal.pone.0009490 20224823 PMC2835736

[koaf124-B68] Quinlan AR, Hall IM. BEDTools: a flexible suite of utilities for comparing genomic features. Bioinformatics. 2010:26(6):841–842. 10.1093/bioinformatics/btq033 20110278 PMC2832824

[koaf124-B69] Reece-Hoyes JS, Diallo A, Lajoie B, Kent A, Shrestha S, Kadreppa S, Pesyna C, Dekker J, Myers CL, Walhout AJM. Enhanced yeast one-hybrid assays for high-throughput gene-centered regulatory network mapping. Nat Methods. 2011:8(12):1059–1064. 10.1038/nmeth.1748 22037705 PMC3235803

[koaf124-B70] Rodríguez-Leal D, Lemmon ZH, Man J, Bartlett ME, Lippman ZB. Engineering quantitative trait variation for crop improvement by genome editing. Cell. 2017:171(2):470–480.e8. 10.1016/j.cell.2017.08.030 28919077

[koaf124-B71] Ron M, Kajala K, Pauluzzi G, Wang D, Reynoso MA, Zumstein K, Garcha J, Winte S, Masson H, Inagaki S, et al Hairy root transformation using Agrobacterium rhizogenes as a tool for exploring cell type-specific gene expression and function using tomato as a model. Plant Physiol. 2014:166(2):455–469. 10.1104/pp.114.239392 24868032 PMC4213079

[koaf124-B72] Sakuraba Y, Chaganzhana, Mabuchi A, Iba K, Yanagisawa S. Enhanced NRT1.1/NPF6.3 expression in shoots improves growth under nitrogen deficiency stress in Arabidopsis. Commun Biol. 2021:4(1):256. 10.1038/s42003-021-01775-1 33637855 PMC7910545

[koaf124-B73] Savci S . Investigation of effect of chemical fertilizers on environment. APCBEE Procedia. 2012:1:287–292. 10.1016/j.apcbee.2012.03.047

[koaf124-B74] Shen-Orr SS, Milo R, Mangan S, Alon U. Network motifs in the transcriptional regulation network of *Escherichia coli*. Nat Genet. 2002:31(1):64–68. 10.1038/ng881 11967538

[koaf124-B75] Shimada TL, Shimada T, Hara-Nishimura I. A rapid and non-destructive screenable marker, FAST, for identifying transformed seeds of *Arabidopsis thaliana*. Plant J. 2010:61(3):519–528. 10.1111/j.1365-313X.2009.04060.x 19891705

[koaf124-B76] Sinha E, Michalak AM, Balaji V. Eutrophication will increase during the 21st century as a result of precipitation changes. Science. 2017:357(6349):405–408. 10.1126/science.aan2409 28751610

[koaf124-B77] Song W, Sun H, Li J, Gong X, Huang S, Zhu X, Zhang Y, Xu G. Auxin distribution is differentially affected by nitrate in roots of two rice cultivars differing in responsiveness to nitrogen. Ann Bot. 2013:112(7):1383–1393. 10.1093/aob/mct212 24095838 PMC3806541

[koaf124-B78] Stitt M . Nitrate regulation of metabolism and growth. Curr Opin Plant Biol. 1999:2(3):178–186. 10.1016/S1369-5266(99)80033-8 10375569

[koaf124-B79] Sullivan AM, Arsovski AA, Lempe J, Bubb KL, Weirauch MT, Sabo PJ, Sandstrom R, Thurman RE, Neph S, Reynolds AP, et al Mapping and dynamics of regulatory DNA and transcription factor networks in *A. thaliana*. Cell Rep. 2014:8(6):2015–2030. 10.1016/j.celrep.2014.08.019 25220462

[koaf124-B80] Sunseri F, Aci MM, Mauceri A, Caldiero C, Puccio G, Mercati F, Abenavoli MR. Short-term transcriptomic analysis at organ scale reveals candidate genes involved in low N responses in NUE-contrasting tomato genotypes. Front Plant Sci. 2023:14:1125378. 10.3389/fpls.2023.1125378 36938018 PMC10020590

[koaf124-B81] Tomato Genome Consortium . The tomato genome sequence provides insights into fleshy fruit evolution. Nature. 2012:485(7400):635–641. 10.1038/nature11119 22660326 PMC3378239

[koaf124-B82] Tsay YF, Schroeder JI, Feldmann KA, Crawford NM. The herbicide sensitivity gene CHL1 of Arabidopsis encodes a nitrate-inducible nitrate transporter. Cell. 1993:72(5):705–713. 10.1016/0092-8674(93)90399-B 8453665

[koaf124-B83] Ulmasov T, Murfett J, Hagen G, Guilfoyle TJ. Aux/IAA proteins repress expression of reporter genes containing natural and highly active synthetic auxin response elements. Plant Cell. 1997:9(11):1963–1971. 10.1105/tpc.9.11.1963 9401121 PMC157050

[koaf124-B84] Varala K, Marshall-Colón A, Cirrone J, Brooks MD, Pasquino AV, Léran S, Mittal S, Rock TM, Edwards MB, Kim GJ, et al Temporal transcriptional logic of dynamic regulatory networks underlying nitrogen signaling and use in plants. Proc Natl Acad Sci U S A. 2018:115(25):6494–6499. 10.1073/pnas.1721487115 29769331 PMC6016767

[koaf124-B85] Vega A, O’Brien JA, Gutiérrez RA. Nitrate and hormonal signaling crosstalk for plant growth and development. Curr Opin Plant Biol. 2019:52:155–163. 10.1016/j.pbi.2019.10.001 31726384

[koaf124-B86] Vidal EA, Araus V, Lu C, Parry G, Green PJ, Coruzzi GM, Gutiérrez RA. Nitrate-responsive miR393/AFB3 regulatory module controls root system architecture in *Arabidopsis thaliana*. Proc Natl Acad Sci U S A. 2010:107(9):4477–4482. 10.1073/pnas.0909571107 20142497 PMC2840086

[koaf124-B87] Vidal EA, Moyano TC, Canales J, Gutiérrez RA. Nitrogen control of developmental phase transitions in *Arabidopsis thaliana*. J Exp Bot. 2014:65(19):5611–5618. 10.1093/jxb/eru326 25129132

[koaf124-B88] Wang R, Guan P, Chen M, Xing X, Zhang Y, Crawford NM. Multiple regulatory elements in the Arabidopsis NIA1 promoter act synergistically to form a nitrate enhancer. Plant Physiol. 2010:154(1):423–432. 10.1104/pp.110.162586 20668061 PMC2938143

[koaf124-B89] Wang R, Okamoto M, Xing X, Crawford NM. Microarray analysis of the nitrate response in Arabidopsis roots and shoots reveals over 1,000 rapidly responding genes and new linkages to glucose, trehalose-6-phosphate, iron, and sulfate metabolism. Plant Physiol. 2003:132(2):556–567. 10.1104/pp.103.021253 12805587 PMC166997

[koaf124-B90] Wang YH, Garvin DF, Kochian LV. Nitrate-induced genes in tomato roots. Array analysis reveals novel genes that may play a role in nitrogen nutrition. Plant Physiol. 2001:127(1):345–359. 10.1104/pp.127.1.345 11553762 PMC117990

[koaf124-B91] Weirauch MT, Yang A, Albu M, Cote AG, Montenegro-Montero A, Drewe P, Najafabadi HS, Lambert SA, Mann I, Cook K, et al Determination and inference of eukaryotic transcription factor sequence specificity. Cell. 2014:158(6):1431–1443. 10.1016/j.cell.2014.08.009 25215497 PMC4163041

[koaf124-B92] Wu J, Zhang Z-S, Xia J-Q, Alfatih A, Song Y, Huang Y-J, Wan G-Y, Sun L-Q, Tang H, Liu Y, et al Rice NIN-LIKE PROTEIN 4 plays a pivotal role in nitrogen use efficiency. Plant Biotechnol J. 2021:19(3):448–461. 10.1111/pbi.13475 32876985 PMC7955889

[koaf124-B93] Yoo S-D, Cho Y-H, Sheen J. Arabidopsis mesophyll protoplasts: a versatile cell system for transient gene expression analysis. Nat Protoc. 2007:2(7):1565–1572. 10.1038/nprot.2007.199 17585298

[koaf124-B94] Young MD, Wakefield MJ, Smyth GK, Oshlack A. Gene ontology analysis for RNA-seq: accounting for selection bias. Genome Biol. 2010:11(2):R14. 10.1186/gb-2010-11-2-r14 20132535 PMC2872874

[koaf124-B95] Yu L-H, Wu J, Tang H, Yuan Y, Wang S-M, Wang Y-P, Zhu Q-S, Li S-G, Xiang C-B. Overexpression of Arabidopsis NLP7 improves plant growth under both nitrogen-limiting and -sufficient conditions by enhancing nitrogen and carbon assimilation. Sci Rep. 2016:6(1):27795. 10.1038/srep27795 27293103 PMC4904239

[koaf124-B96] Zhang H, Forde BG. An Arabidopsis MADS box gene that controls nutrient-induced changes in root architecture. Science. 1998:279(5349):407–409. 10.1126/science.279.5349.407 9430595

[koaf124-B97] Zhang H, Forde BG. Regulation of Arabidopsis root development by nitrate availability. J Exp Bot. 2000:51(342):51–59. 10.1093/jxb/51.342.51 10938795

[koaf124-B98] Zong DM, Sadeghpour M, Molinari S, Alnahhas RN, Hirning AJ, Giannitsis C, Ott W, Josić K, Bennett MR. Tunable dynamics in a multistrain transcriptional pulse generator. ACS Synth Biol. 2023:12(12):3531–3543. 10.1021/acssynbio.3c00434 38016068

[koaf124-B99] Zouine M, Fu Y, Chateigner-Boutin A-L, Mila I, Frasse P, Wang H, Audran C, Roustan J-P, Bouzayen M. Characterization of the tomato ARF gene family uncovers a multi-levels post-transcriptional regulation including alternative splicing. PLoS One. 2014:9(1):e84203. 10.1371/journal.pone.0084203 24427281 PMC3888382

[koaf124-B100] Zürcher E, Tavor-Deslex D, Lituiev D, Enkerli K, Tarr PT, Müller B. A robust and sensitive synthetic sensor to monitor the transcriptional output of the cytokinin signaling network in planta. Plant Physiol. 2013:161(3):1066–1075. 10.1104/pp.112.211763 23355633 PMC3585579

