# Supplementary material for: Conservation and divergence of regulatory architecture in nitrate-responsive plant gene circuits
Source: Plant Cell. 2025 May 22;37(6):koaf124. doi: 10.1093/plcell/koaf124 (PMC12205479; doi:10.1093/plcell/koaf124)
Supplement: koaf124_Supplementary_Data [file koaf124_supplementary_data.zip › SupplementaryFileS3.pdf]

```
CLUSTAL O(1.2.4) multiple sequence alignment


AtNLP7       MCEPDDN---------------------
SARNGVTTQPSRSRELLMDVDDLDLDGSWPLD        39
SlNLP7A
MSEPEEEMNFIFRSKPKDFVHPPPATAAAAQQQQHAVGENHRDSLMMDLDLDLDASWSFD    60
SlNLP7B      MSEPGGGMTQNHLPKS---------------
KELTPATVTERESMMMDLDFDIDASWSFD   45
             *.**                          :         *: :*
*:*:*.** :*

AtNLP7       QIPYLSS--
SNRMISPIFVSSSSEQPCSPLWAFSDGGGNGFHHATSGGDDEKISSVSGVP     97
SlNLP7A      QIFAAAAS-ASNPMSPFLVSAASEQPCSPLWAFSDENEDKPN-----------
GNALSTG 108
SlNLP7B      QIFAAAAAVSSNPASPFL-------PCSPLWAFPDDNDEKPA-----------
GNGLS-G 86
             **   ::  :..  **::       ********
* . :              ..   .

AtNLP7       SFRLAEYPLFLPYSSPSAAEN--
TTEKHNSFQFPSPLMSLVPPENTDNYCVIKERMTQAL   155
SlNLP7A
SLRLSNYPRFVTYANEHEAAPETVSVTDDKKRIPPPIKGLAPLDYLDSSCIIKERMTQAL    168
SlNLP7B
ALRISGHPRFVAYTGDLEATTETISVNTDKGRLTSPISGLLPGDNPEGSCIIKERMTQAL    146
             ::*:: :* *: *:.   *     : . :. ::  *: .* * :  :.
*:*********

AtNLP7
RYFKESTEQHVLAQVWAPVRKNGRDLLTTLGQPFVLNPNGNGLNQYRMISLTYMFSVDSE    215
SlNLP7A
RYFKESTGERVLAQVWAPVKNGGRYVLTTSGQPFVLDPDCNGLHQYRMVSLMYMFSVDGE    228
SlNLP7B
RYLKETSGERVLAQVWAPVKEAGRSVLTTSGQPFVLDPECNGLHQYRTVSLMYMFAADGE    206
             **:**:: ::*********:: ** :*** ******:*: ***:*** :**
***:.*.*

AtNLP7
SDVELGLPGRVFRQKLPEWTPNVQYYSSKEFSRLDHALHYNVRGTLALPVFNPSGQSCIG    275
SlNLP7A
TDGVLGLPGRVYRKKLPEWTPNVQYYSSKEFPRLNHALDYNVRGTLALPVFEPSGQSCVG    288
SlNLP7B
TDGVLGLPGRVFRLKLPEWTPNVQYYSSKEFPRLDHALNYNVRGTLALPVFEPSGRSCVG    266
             :*  *******:* *****************
**:***.***********:***:**:*

AtNLP7       VVELIMTSEKIHYAPEVDKVCKALEAVNLKSSEILDHQTT---------
QICNESRQNAL     326
```

SlNLP7A       VLELIMTSQKINYAPEVDKVCKALEAVNLKSSEILDYPNH---------
QICNEGRQNAL     339
SlNLP7B
VLELIMTSQKINYAAEVDKVCKALEAVNLKSSDILDHPNTQVYVMGYMNQICNEGRQNAL    326
              *:******:**:** ****************:***: .
*****.*****

AtNLP7
AEILEVLTVVCETHNLPLAQTWVPCQHGSVLANGGGLKKNCTSFDGSCMGQICMSTTDMA    386
SlNLP7A
VEILEILTAVCETYKLPLAQTWVPCRHRSVLADGGGFKKSCSSFDGSCMGQVCMSTTDVA    399
SlNLP7B
VDILEILTAVCETYKLPLAQTWVPCRHRSVLADGGGLRKSCSSFDGSCMGQICMSTTDVA    386
              .:***:**.****::**********:*
****:***::*.*:*********:******:*

AtNLP7
CYVVDAHVWGFRDACLEHHLQKGQGVAGRAFLNGGSCFCRDITKFCKTQYPLVHYALMFK    446
SlNLP7A
FYVVDAHMWGFREACAEHHLQKGQGVAGRAYASQKSCFCEDIGKFCKTEYPLVHYARLFG    459
SlNLP7B
FYVVDAHMWGFRDACAEHHLQRGQGVAGRAYASRKSCYCEDITQFCKTEYPLVHYARMFG    446
               ******:****:** *****:********: .
**:*.** :****:******* :*

AtNLP7
LTTCFAISLQSSYTGDDSYILEFFLPSSITDDQEQDLLLGSILVTMKEHFQSLRVASGVD    506
SlNLP7A
LSRCFAICLRSTHTGNDDYILEFFLPPNDGDYTDQLALLNSLLLTMKQHFRSLRVASGEE    519
SlNLP7B
LTSCFAICLRSSHTANDDYILEFFLPPNSGDYSDQPALLNSLLLTMKQHFRSLSIASGEE    506
              *: ****.*:*::*.:*.******** .  *  :*
**.*:*:***:**:** :*** :

AtNLP7        FGEDDDKLSFEIIQALPDKKVHSKIESIRVPFSGFKSNA-
TETMLIPQPVVQSSD-----    560
SlNLP7A       LEHDW--
GSVEIIKASTEEKLGSRFDSVPTTKSLPQSASVANGRRHPDLMEEQHSTV---   574
SlNLP7B       LEHDW--
GSVEIIQASMEEKIDAKPESVPTAKTSPQLTSLPNGWVHLDPVGEQQSAVGSN   564
              : .*    *.***:*  ::*: :: :*: .  :  :  :  :     : : :. .

AtNLP7        ---------
PVNEKINVATVNGVVKEKKKTEKKRGKTEKTISLDVLQQYFTGSLKDAAKS     611
SlNLP7A
-AKGAEGVNVTAEAHNHASVPQNKQTGKKSERKRGKAEKTISLEVLQQYFAGSLKDAAKS    633
SlNLP7B
VSKGARSTSGTGEAPNNVSNSDNKTSGKKSERKRGKAEKTISLEVLQQYFAGSLKDAAKS    624
                       . *  * .:
**:*:****:******:******:*********

```
AtNLP7
LGVCPTTMKRICRQHGISRWPSRKIKKVNRSITKLKRVIESVQGTDGGLDLTSMAVS---    668
SlNLP7A
LGVCPTTMKRICRQHGISRWPSRKINKVNRSLSKLKRVIESVQGADGTFSLTSLAPNSLP    693
SlNLP7B
LGVCPTTMKRICRQHGISRWPSRKINKVNRSLSKLKCVIESVQGAEGAFTLTSLAPNSLP    684
             ***********************:*****::*** *******::* : ***:* .

AtNLP7       ----SIPWTHGQTSAQPLNSPNGSKPPELPNTNNSPNHWSSDHS--
PNEPN---------  713
SlNLP7A      VAVGSISWPAGINGS-P------
CKASEYQEEKNEFSNHGTPGSHEEAEPTDQMLGSRII   746
SlNLP7B      AAVSSISWPAGANVS-
NLPSSPSSKPSVFPEEKNEFFHHGTPESHIEAEPSNQMLGGRVA    743
                 ** *  * . :        .*     : :*.  : .:  *    **.

AtNLP7       GSPELPP-------SNGHKR----
SRTVDESAGTPTSHGSCDGNQLDEPKVP--------    754
SlNLP7A      GNEELSPKLNGFVR-
EGSHRSRTGSFSREESTGTPTSHGSCQGSPSPANESSPQNELLNS   805
SlNLP7B
RKEEFTPMQNGFLHAEGTHKSRTGSVSREESAGTPTSHGSCQGSPCAGNGFSPQNELVNS    803
              . *: *        :* ::    * : :**:*********:*.

AtNLP7       -NQDPLFTVGGSPGLLFPPYSRDH-DVSAASFAMPNRLLG--
SIDHFRGMLIEDAGSSKD      810
SlNLP7A      PTQESVMKVEGSLEP---
ARQTTGELNLSTAFLMPGLFIPEHTHQQFRGMLVEDAGSSHD      862
SlNLP7B      PAHESCMKVGGSLEA---
ARQTTAEINLSSAFLMPQPIIPKHTQEPFGGMLVEDAGSSHD      860
               ::  :.* **        .       :::* **  ::   : : *
***:******:*

AtNLP7       LRNLCPTA--AFDDKFQDTNWMNNDNN---
SNNNLYAPPKEEAIANVACEPSGSEMRTVT  865
SlNLP7A      LRNLCPAGETMFDERVPEYSWTNPPCSNGIATNQVPLPVEK-----
MPQFSSRPEVTSVT  917
SlNLP7B      LRNLCSPRDALVDERVPDYNLTNPPFSDAIAKDPVYVPPDT-----
IQQYSAWPEVTSVT  915
             *****      .*::. : .  *   .   :.: :  * .      :    :
*: :**

AtNLP7
IKASYKDDIIRFRISSGSGIMELKDEVAKRLKVDAGTFDIKYLDDDNEWVLIACDADLQE    925
SlNLP7A
IKATYREDIIRFRLCLNSGIYKLKEEVSKRLKLEMGTFDIKYLDDDHEWVLIACDADLQE    977
SlNLP7B
IKATYKEDIIRFRLCLSSGIVKLKEEVAKRLKLELGTFYIKYLDDDLEFVPISCDADLQE    975
             ***:*::******:. .*** :**:**:****:: *** ******* *:*
```

```
*:*******

AtNLP7       CLEIPRSSRTKIVRLLVHDVTTNLGSSCESTGEL 959
SlNLP7A      CIDISSSSGSNVVRLLVHDIMPNLGSSCESSGE- 1010
SlNLP7B      CVDISRSSGSSIVRLLIHDIMSNLGSSCESSGK- 1008
             *::*  ** :.:****:**:  ********:*:
```

RaxML_bipartitions_25coll.AtARF9redux.mafft.trimal.tre

```
#NEXUS
begin taxa;
	dimensions ntax=45;
	taxlabels
	'AT1G34310.1'
	'AT1G34390.1'
	'AT1G34410.1'
	'AT1G35240.1'
	'AT1G35520.1'
	'AT1G35540.1'
	'AT4G23980.1'[&!color=#058011]
	Bol006390
	Bol020075
	Bol024901
	Bol027367
	Bol032178
	Bol032614
	Bol042142
	CA01g00820
	CA01g34570
	'Carub.0001s3254.1.p'
	'Carub.0001s3290.1.p'
	'Carub.0001s3328.1.p'
	'Carub.0005s0941.1.p'
	'Carub.0007s1711.1.p'
	Cc08_g16330
	'Cp4.1LG01g00450.1'
	'Cp4.1LG05g07840.1'
	'Cp4.1LG13g00970.1'
	'Cp4.1LG14g02660.1'
	DCAR_006517
	HanXRQChr06g0166341
	HanXRQChr07g0190781
	HanXRQChr13g0414871
	HanXRQChr17g0535011
	MD06G1111100
	MD14G1131900
	'Medtr4g021580.1'
	'Medtr7g062540.1'
	'Migut.A00611.1.p'
	OIT02046
	PGSC0003DMP400021708
	'Peaxi162Scf00074g00537.1'
	'Potri.001G088600.3.p'
	'Potri.003G142100.1.p'
	'Solyc08g008380.4.1'[&!color=#800a12]
	'Solyc08g082630.3.1'[&!color=#800a12]
```

```
        'Thecc.03G301500.1.p'
        'VIT_202s0025g01740.1'
;
end;

begin trees;
        tree tree_1 = [&R] (((Bol027367:0.09477,Bol006390:0.033568)
[&label=100.0]:0.064538,Bol024901:0.378084)[&label=89.0]:0.069322,
((Bol032614:0.105789,Bol032178:0.093078)[&label=100.0]:0.196402,
((('Carub.0005s0941.1.p':0.082642,
(('Carub.0001s3290.1.p':0.01631,'Carub.0001s3328.1.p':0.024174)
[&label=95.0]:0.016097,'Carub.0001s3254.1.p':0.058676)
[&label=70.0]:0.015455)[&label=100.0]:0.272712,
('AT1G35540.1':0.066334,('AT1G34390.1':0.039215,
('AT1G34310.1':0.060507,('AT1G35520.1':0.046955,
('AT1G35240.1':0.028084,'AT1G34410.1':0.036141)[&label=99.0]:0.025535)
[&label=77.0]:0.016076)[&label=57.0]:0.01183)[&label=100.0]:0.037471)
[&label=100.0]:0.131651)[&label=100.0]:0.180748,
((('Potri.001G088600.3.p':0.051822,'Potri.003G142100.1.p':0.06173)
[&label=100.0]:0.082428,(((('Migut.A00611.1.p':0.279534,
(Cc08_g16330:0.230189,
((CA01g34570:0.160125,'Solyc08g008380.4.1':0.088516)
[&label=100.0]:0.172804,('Peaxi162Scf00074g00537.1':0.087117,
((CA01g00820:0.152549,
('Solyc08g082630.3.1':0.103202,PGSC0003DMP400021708:0.03071)
[&label=100.0]:0.090858)[&label=100.0]:0.071565,OIT02046:0.06135)
[&label=55.0]:0.009721)[&label=100.0]:0.08816)[&label=100.0]:0.108064)
[&label=45.0]:0.02055)[&label=90.0]:0.054679,
((HanXRQChr13g0414871:0.191856,
((HanXRQChr06g0166341:0.099508,HanXRQChr17g0535011:0.235435)
[&label=100.0]:0.058076,HanXRQChr07g0190781:0.23058)
[&label=88.0]:0.029646)[&label=100.0]:0.082121,DCAR_006517:0.267786)
[&label=75.0]:0.034978)
[&label=100.0]:0.107527,'VIT_202s0025g01740.1':0.152821)
[&label=89.0]:0.041415,
(((('Cp4.1LG14g02660.1':0.073942,'Cp4.1LG01g00450.1':0.121209)
[&label=100.0]:0.199275,
('Cp4.1LG13g00970.1':0.06022,'Cp4.1LG05g07840.1':0.090235)
[&label=100.0]:0.278402)[&label=70.0]:0.030008,
('Medtr7g062540.1':0.210418,'Medtr4g021580.1':0.17969)
[&label=100.0]:0.106424)[&label=39.0]:0.023261,
(MD14G1131900:0.038775,MD06G1111100:0.047565)[&label=100.0]:0.138346)
[&label=31.0]:0.014215)
[&label=30.0]:0.037527,'Thecc.03G301500.1.p':0.166338)
[&label=100.0]:0.233837,(Bol042142:0.200737,
('AT4G23980.1':0.052212,'Carub.0007s1711.1.p':0.047982)
[&label=97.0]:0.031811)[&label=100.0]:0.161701)[&label=100.0]:0.36674)
[&label=100.0]:0.281439)[&label=99.0]:0.107832,Bol020075:0.289554);
end;
```

```
begin figtree;
	set appearance.backgroundColorAttribute="Default";
	set appearance.backgroundColour=#ffffff;
	set appearance.branchColorAttribute="User selection";
	set appearance.branchColorGradient=false;
	set appearance.branchLineWidth=1.0;
	set appearance.branchMinLineWidth=0.0;
	set appearance.branchWidthAttribute="Fixed";
	set appearance.foregroundColour=#000000;
	set appearance.hilightingGradient=false;
	set appearance.selectionColour=#2d3680;
	set branchLabels.colorAttribute="User selection";
	set branchLabels.displayAttribute="Branch times";
	set branchLabels.fontName="sansserif";
	set branchLabels.fontSize=8;
	set branchLabels.fontStyle=0;
	set branchLabels.isShown=false;
	set branchLabels.significantDigits=4;
	set layout.expansion=0;
	set layout.layoutType="RECTILINEAR";
	set layout.zoom=0;
	set legend.attribute="label";
	set legend.fontSize=10.0;
	set legend.isShown=false;
	set legend.significantDigits=4;
	set nodeBars.barWidth=4.0;
	set nodeBars.displayAttribute=null;
	set nodeBars.isShown=false;
	set nodeLabels.colorAttribute="User selection";
	set nodeLabels.displayAttribute="label";
	set nodeLabels.fontName="sansserif";
	set nodeLabels.fontSize=8;
	set nodeLabels.fontStyle=0;
	set nodeLabels.isShown=true;
	set nodeLabels.significantDigits=4;
	set nodeShapeExternal.colourAttribute="User selection";
	set nodeShapeExternal.isShown=false;
	set nodeShapeExternal.minSize=10.0;
	set nodeShapeExternal.scaleType=Width;
	set nodeShapeExternal.shapeType=Circle;
	set nodeShapeExternal.size=4.0;
	set nodeShapeExternal.sizeAttribute="Fixed";
	set nodeShapeInternal.colourAttribute="User selection";
	set nodeShapeInternal.isShown=true;
	set nodeShapeInternal.minSize=10.0;
	set nodeShapeInternal.scaleType=Width;
	set nodeShapeInternal.shapeType=Circle;
	set nodeShapeInternal.size=4.0;
	set nodeShapeInternal.sizeAttribute="Fixed";
	set polarLayout.alignTipLabels=false;
```

```
	set polarLayout.angularRange=0;
	set polarLayout.rootAngle=0;
	set polarLayout.rootLength=100;
	set polarLayout.showRoot=true;
	set radialLayout.spread=0.0;
	set rectilinearLayout.alignTipLabels=false;
	set rectilinearLayout.curvature=0;
	set rectilinearLayout.rootLength=100;
	set scale.offsetAge=0.0;
	set scale.rootAge=1.0;
	set scale.scaleFactor=1.0;
	set scale.scaleRoot=false;
	set scaleAxis.automaticScale=true;
	set scaleAxis.fontSize=8.0;
	set scaleAxis.isShown=false;
	set scaleAxis.lineWidth=1.0;
	set scaleAxis.majorTicks=1.0;
	set scaleAxis.minorTicks=0.5;
	set scaleAxis.origin=0.0;
	set scaleAxis.reverseAxis=false;
	set scaleAxis.showGrid=true;
	set scaleBar.automaticScale=true;
	set scaleBar.fontSize=10.0;
	set scaleBar.isShown=true;
	set scaleBar.lineWidth=1.0;
	set scaleBar.scaleRange=0.0;
	set tipLabels.colorAttribute="User selection";
	set tipLabels.displayAttribute="Names";
	set tipLabels.fontName="sansserif";
	set tipLabels.fontSize=12;
	set tipLabels.fontStyle=0;
	set tipLabels.isShown=true;
	set tipLabels.significantDigits=4;
	set trees.order=false;
	set trees.orderType="increasing";
	set trees.rooting=false;
	set trees.rootingType="User Selection";
	set trees.transform=false;
	set trees.transformType="cladogram";
end;
```

RAxML_bipartitions_25coll.AtARF18redux.mafft.trimal.tre

```
#NEXUS
begin taxa;
	dimensions ntax=22;
	taxlabels
	'AT2G46530.3'
	'AT3G61830.1'[&!color=#0c8008]
	Bol045672
	CA00g64660
	'Carub.0004s2924.1.p'
	'Carub.0005s2857.1.p'
	Cc02_g39520
	'Cp4.1LG17g10130.1'
	DCAR_005400
	DCAR_025262
	MD01G1083400
	MD07G1152100
	'Medtr8g027440.1'
	'Migut.B01886.1.p'
	OIT07754
	PGSC0003DMP400000262
	'Peaxi162Scf00345g00027.1'
	'Potri.002G172800.3.p'
	'Potri.014G100100.5.p'
	'Solyc01g096070.3.1'[&!color=#800915]
	'Thecc.01G340700.1.p'
	'VIT_215s0046g00290.2'
;
end;

begin trees;
	tree tree_1 = [&R] ('AT2G46530.3':0.073315,
(('Thecc.01G340700.1.p':0.126789,
((('Potri.002G172800.3.p':0.058639,'Potri.014G100100.5.p':0.064684)
[&label=100.0]:0.118513,(('VIT_215s0046g00290.2':0.160175,
((Cc02_g39520:0.269858,(OIT07754:0.055794,
('Peaxi162Scf00345g00027.1':0.058948,(CA00g64660:0.050652,
('Solyc01g096070.3.1':0.037048,PGSC0003DMP400000262:0.012664)
[&label=100.0]:0.02094)[&label=100.0]:0.035829)[&label=51.0]:0.007398)
[&label=100.0]:0.174495)[&label=86.0]:0.046979,
((DCAR_025262:0.242221,DCAR_005400:0.251059)
[&label=100.0]:0.207849,'Migut.B01886.1.p':0.353438)
[&label=72.0]:0.044804)[&label=100.0]:0.073943)[&label=93.0]:0.045273,
((MD01G1083400:0.034839,MD07G1152100:0.062954)
[&label=100.0]:0.154955,'Medtr8g027440.1':0.401764)
[&label=49.0]:0.027582)[&label=32.0]:0.017698)
[&label=29.0]:0.00659,'Cp4.1LG17g10130.1':0.416705)
[&label=87.0]:0.045711)[&label=100.0]:0.331673,
('Carub.0005s2857.1.p':0.064618,
```

```
(Bol045672:0.166094,'AT3G61830.1':0.05614)[&label=50.0]:0.026629)
[&label=100.0]:0.2418)
[&label=100.0]:0.155378,'Carub.0004s2924.1.p':0.077482);
end;

begin figtree;
	set appearance.backgroundColorAttribute="Default";
	set appearance.backgroundColour=#ffffff;
	set appearance.branchColorAttribute="User selection";
	set appearance.branchColorGradient=false;
	set appearance.branchLineWidth=1.0;
	set appearance.branchMinLineWidth=0.0;
	set appearance.branchWidthAttribute="Fixed";
	set appearance.foregroundColour=#000000;
	set appearance.hilightingGradient=false;
	set appearance.selectionColour=#2d3680;
	set branchLabels.colorAttribute="User selection";
	set branchLabels.displayAttribute="Branch times";
	set branchLabels.fontName="sansserif";
	set branchLabels.fontSize=8;
	set branchLabels.fontStyle=0;
	set branchLabels.isShown=false;
	set branchLabels.significantDigits=4;
	set layout.expansion=0;
	set layout.layoutType="RECTILINEAR";
	set layout.zoom=0;
	set legend.attribute="label";
	set legend.fontSize=10.0;
	set legend.isShown=false;
	set legend.significantDigits=4;
	set nodeBars.barWidth=4.0;
	set nodeBars.displayAttribute=null;
	set nodeBars.isShown=false;
	set nodeLabels.colorAttribute="User selection";
	set nodeLabels.displayAttribute="label";
	set nodeLabels.fontName="sansserif";
	set nodeLabels.fontSize=8;
	set nodeLabels.fontStyle=0;
	set nodeLabels.isShown=true;
	set nodeLabels.significantDigits=4;
	set nodeShapeExternal.colourAttribute="User selection";
	set nodeShapeExternal.isShown=false;
	set nodeShapeExternal.minSize=10.0;
	set nodeShapeExternal.scaleType=Width;
	set nodeShapeExternal.shapeType=Circle;
	set nodeShapeExternal.size=4.0;
	set nodeShapeExternal.sizeAttribute="Fixed";
	set nodeShapeInternal.colourAttribute="User selection";
	set nodeShapeInternal.isShown=true;
	set nodeShapeInternal.minSize=10.0;
```

```
        set nodeShapeInternal.scaleType=Width;
        set nodeShapeInternal.shapeType=Circle;
        set nodeShapeInternal.size=4.0;
        set nodeShapeInternal.sizeAttribute="Fixed";
        set polarLayout.alignTipLabels=false;
        set polarLayout.angularRange=0;
        set polarLayout.rootAngle=0;
        set polarLayout.rootLength=100;
        set polarLayout.showRoot=true;
        set radialLayout.spread=0.0;
        set rectilinearLayout.alignTipLabels=false;
        set rectilinearLayout.curvature=0;
        set rectilinearLayout.rootLength=100;
        set scale.offsetAge=0.0;
        set scale.rootAge=1.0;
        set scale.scaleFactor=1.0;
        set scale.scaleRoot=false;
        set scaleAxis.automaticScale=true;
        set scaleAxis.fontSize=8.0;
        set scaleAxis.isShown=false;
        set scaleAxis.lineWidth=1.0;
        set scaleAxis.majorTicks=1.0;
        set scaleAxis.minorTicks=0.5;
        set scaleAxis.origin=0.0;
        set scaleAxis.reverseAxis=false;
        set scaleAxis.showGrid=true;
        set scaleBar.automaticScale=true;
        set scaleBar.fontSize=10.0;
        set scaleBar.isShown=true;
        set scaleBar.lineWidth=1.0;
        set scaleBar.scaleRange=0.0;
        set tipLabels.colorAttribute="User selection";
        set tipLabels.displayAttribute="Names";
        set tipLabels.fontName="sansserif";
        set tipLabels.fontSize=12;
        set tipLabels.fontStyle=0;
        set tipLabels.isShown=true;
        set tipLabels.significantDigits=4;
        set trees.order=false;
        set trees.orderType="increasing";
        set trees.rooting=false;
        set trees.rootingType="User Selection";
        set trees.transform=true;
        set trees.transformType="cladogram";
end;
```

RAxML_bipartitions_25coll.AtDREB26redux.mafft.trimal.tre

```
#NEXUS
begin taxa;
	dimensions ntax=41;
	taxlabels
	'AT1G21910.1'[&!color=#0e8008]
	'AT1G44830.1'
	'AT1G77640.1'[&!color=#0e8008]
	Bol009696
	Bol027544
	Bol044935
	CA04g07350
	'Carub.0001s2138.1.p'
	'Carub.0001s3535.1.p'
	'Carub.0002s2453.1.p'
	Cc10_g10960
	'Cp4.1LG01g20460.1'
	'Cp4.1LG02g03240.1'
	'Cp4.1LG06g06280.1'
	DCAR_014103
	DCAR_017405
	HanXRQChr03g0083341
	HanXRQChr05g0163451
	HanXRQChr13g0415751
	HanXRQChr15g0485281
	'LOC_Os06g10780.1'
	MA_162045g0010
	MD08G1166100
	MD15G1352400
	'Medtr3g072610.1'
	'Medtr5g058470.1'
	'Migut.C01143.1.p'
	'Migut.N03295.1.p'
	OIT32132
	PGSC0003DMP400001723
	'Peaxi162Scf00497g00041.1'
	'Potri.002G085600.1.p'
	'Seita.1G332600.1.p'
	'Sobic.004G310600.1.p'
	'Sobic.010G080400.1.p'
	'Solyc11g012980.1.1'[&!color=#801220]
	'Thecc.08G122200.1.p'
	'VIT_218s0001g03240.1'
	Zm00001d018191_P001
	Zm00001d037165_P001
	Zm00001d052152_P001
;
end;
```

```
begin trees;
	tree tree_1 = [&R]
(((('Cp4.1LG02g03240.1':0.123171,'Cp4.1LG06g06280.1':0.097692)
[&label=92.0]:0.112286,MA_162045g0010:1.24001)[&label=38.0]:0.179298,
((Zm00001d052152_P001:1.0E-6,((Zm00001d018191_P001:0.108252,
((Zm00001d037165_P001:0.131997,'Sobic.010G080400.1.p':0.066235)
[&label=100.0]:0.17459,'LOC_Os06g10780.1':0.041831)
[&label=97.0]:0.155376)[&label=39.0]:0.021829,
('Seita.1G332600.1.p':0.043707,'Sobic.004G310600.1.p':0.026771)
[&label=33.0]:0.008764)[&label=67.0]:0.031727)
[&label=99.0]:0.495401,'Cp4.1LG01g20460.1':0.272508)
[&label=44.0]:0.232517,((('Carub.0001s2138.1.p':0.033345,
(Bol009696:0.096831,'AT1G21910.1':0.03471)[&label=65.0]:0.030088)
[&label=86.0]:0.113831,(Bol027544:0.034745,
('Carub.0002s2453.1.p':0.078933,'AT1G77640.1':0.064213)
[&label=78.0]:0.030029)[&label=97.0]:0.12765)[&label=96.0]:0.176744,
(DCAR_017405:0.198633,DCAR_014103:0.164908)[&label=100.0]:0.302893)
[&label=75.0]:0.227873,(MD15G1352400:0.05649,MD08G1166100:0.039252)
[&label=79.0]:0.150386,
('Medtr3g072610.1':0.208875,'Medtr5g058470.1':0.145691)
[&label=72.0]:0.187859,
('Thecc.08G122200.1.p':0.023392,'Potri.002G085600.1.p':0.102727)
[&label=57.0]:0.10637,
('Migut.C01143.1.p':0.379255,Cc10_g10960:0.048031)
[&label=57.0]:0.346146,(HanXRQChr15g0485281:0.317699,
(HanXRQChr05g0163451:0.416835,
(HanXRQChr03g0083341:0.345315,HanXRQChr13g0415751:0.166773)
[&label=87.0]:0.180392)[&label=62.0]:0.049256)[&label=72.0]:0.390912,
('Peaxi162Scf00497g00041.1':0.120589,((CA04g07350:0.030361,
(PGSC0003DMP400001723:0.015916,'Solyc11g012980.1.1':0.047429)
[&label=76.0]:0.026752)[&label=63.0]:0.014571,OIT32132:0.067968)
[&label=30.0]:0.021837)
[&label=99.0]:0.476741,'Migut.N03295.1.p':0.436392,'VIT_218s0001g03240
.1':0.28768)[&label=100.0]:0.201906,
('AT1G44830.1':0.025732,'Carub.0001s3535.1.p':0.017435)
[&label=46.0]:0.040125,Bol044935:0.115847);
end;

begin figtree;
	set appearance.backgroundColorAttribute="Default";
	set appearance.backgroundColour=#ffffff;
	set appearance.branchColorAttribute="User selection";
	set appearance.branchColorGradient=false;
	set appearance.branchLineWidth=1.0;
	set appearance.branchMinLineWidth=0.0;
	set appearance.branchWidthAttribute="Fixed";
	set appearance.foregroundColour=#000000;
	set appearance.hilightingGradient=false;
	set appearance.selectionColour=#2d3680;
	set branchLabels.colorAttribute="User selection";
```

```
set branchLabels.displayAttribute="Branch times";
set branchLabels.fontName="sansserif";
set branchLabels.fontSize=8;
set branchLabels.fontStyle=0;
set branchLabels.isShown=false;
set branchLabels.significantDigits=4;
set layout.expansion=0;
set layout.layoutType="RECTILINEAR";
set layout.zoom=0;
set legend.attribute="label";
set legend.fontSize=10.0;
set legend.isShown=false;
set legend.significantDigits=4;
set nodeBars.barWidth=4.0;
set nodeBars.displayAttribute=null;
set nodeBars.isShown=false;
set nodeLabels.colorAttribute="User selection";
set nodeLabels.displayAttribute="label";
set nodeLabels.fontName="sansserif";
set nodeLabels.fontSize=8;
set nodeLabels.fontStyle=0;
set nodeLabels.isShown=true;
set nodeLabels.significantDigits=4;
set nodeShapeExternal.colourAttribute="User selection";
set nodeShapeExternal.isShown=false;
set nodeShapeExternal.minSize=10.0;
set nodeShapeExternal.scaleType=Width;
set nodeShapeExternal.shapeType=Circle;
set nodeShapeExternal.size=4.0;
set nodeShapeExternal.sizeAttribute="Fixed";
set nodeShapeInternal.colourAttribute="User selection";
set nodeShapeInternal.isShown=false;
set nodeShapeInternal.minSize=10.0;
set nodeShapeInternal.scaleType=Width;
set nodeShapeInternal.shapeType=Circle;
set nodeShapeInternal.size=4.0;
set nodeShapeInternal.sizeAttribute="Fixed";
set polarLayout.alignTipLabels=false;
set polarLayout.angularRange=0;
set polarLayout.rootAngle=0;
set polarLayout.rootLength=100;
set polarLayout.showRoot=true;
set radialLayout.spread=0.0;
set rectilinearLayout.alignTipLabels=false;
set rectilinearLayout.curvature=0;
set rectilinearLayout.rootLength=100;
set scale.offsetAge=0.0;
set scale.rootAge=1.0;
set scale.scaleFactor=1.0;
set scale.scaleRoot=false;
```

```
	set scaleAxis.automaticScale=true;
	set scaleAxis.fontSize=8.0;
	set scaleAxis.isShown=false;
	set scaleAxis.lineWidth=1.0;
	set scaleAxis.majorTicks=1.0;
	set scaleAxis.minorTicks=0.5;
	set scaleAxis.origin=0.0;
	set scaleAxis.reverseAxis=false;
	set scaleAxis.showGrid=true;
	set scaleBar.automaticScale=true;
	set scaleBar.fontSize=10.0;
	set scaleBar.isShown=true;
	set scaleBar.lineWidth=1.0;
	set scaleBar.scaleRange=0.0;
	set tipLabels.colorAttribute="User selection";
	set tipLabels.displayAttribute="Names";
	set tipLabels.fontName="sansserif";
	set tipLabels.fontSize=12;
	set tipLabels.fontStyle=0;
	set tipLabels.isShown=true;
	set tipLabels.significantDigits=4;
	set trees.order=false;
	set trees.orderType="increasing";
	set trees.rooting=false;
	set trees.rootingType="User Selection";
	set trees.transform=true;
	set trees.transformType="cladogram";
end;
```

RAxML_bipartitions_25coll.AtNAC32redux.mafft.trimal.tre

```
#NEXUS
begin taxa;
	dimensions ntax=75;
	taxlabels
	'AT1G01720.1'[&!color=#0000ff]
	'AT1G77450.1'[&!color=#0000ff]
	'AT5G08790.1'[&!color=#0000ff]
	'AT5G63790.2'[&!color=#0000ff]
	'AmTr_v1.0_scaffold00009.309p'
	'AsparagusV1_09.665p'
	Bol008793
	Bol019020
	Bol024596
	Bol040672
	Bol043763
	CA00g03050
	CA05g04410
	CA06g11310
	'Carub.0001s0049.1.p'
	'Carub.0002s2434.1.p'
	'Carub.0006s0776.1.p'
	'Carub.0008s2456.1.p'
	Cc02_g33930
	Cc10_g12200
	'Cp4.1LG00g11300.1'
	'Cp4.1LG01g20600.1'
	'Cp4.1LG02g02980.1'
	'Cp4.1LG05g15540.1'
	'Cp4.1LG06g06180.1'
	'Cp4.1LG13g04550.1'
	'Cp4.1LG16g01180.1'
	DCAR_013176
	DCAR_016661
	GSMUA_Achr10P04570_001
	GSMUA_Achr6P18720_001
	GSMUA_Achr7P23250_001
	HanXRQChr09g0265531
	HanXRQChr12g0357431
	HanXRQChr15g0474211
	HanXRQChr17g0547571
	'LOC_Os01g66120.1'
	'LOC_Os05g34830.1'
	'LOC_Os11g08210.1'
	MD15G1136600
	MD15G1344900
	'Medtr3g088110.2'
	'Medtr3g096920.2'
```

```
        'Medtr8g094580.1'
        'Migut.C01110.1.p'
        'Migut.H00825.1.p'
        'Migut.H00826.1.p'
        'Migut.L00568.1.p'
        OIT02385
        OIT05436
        PGSC0003DMP400016317
        PGSC0003DMP400030689
        PGSC0003DMP400055618
        'Peaxi162Scf00051g01410.1'
        'Peaxi162Scf00842g00211.1'
        'Potri.002G081000.1.p'
        'Potri.005G069500.1.p'
        'Potri.005G180200.1.p'
        'Potri.007G099400.2.p'
        'Seita.3G237100.1.p'
        'Seita.5G405100.1.p'
        'Seita.8G060900.1.p'
        'Sobic.003G379700.1.p'
        'Sobic.005G064600.2.p'
        'Sobic.009G142200.1.p'
        'Solyc04g009440.3.1'[&!color=#800915]
        'Solyc06g060230.3.1'[&!color=#800915]
        'Solyc11g017470.2.1'[&!color=#800915]
        'Thecc.01G140500.1.p'
        'Thecc.08G129000.1.p'
        'VIT_207s0031g02610.1'
        'VIT_218s0001g02300.1'
        Zm00001d000112_P001
        Zm00001d038221_P001
        Zm00001d042609_P001
;
end;

begin trees;
        tree tree_1 = [&R] (((Bol040672:0.040629,
('AT1G01720.1':0.05066,'Carub.0001s0049.1.p':0.032359)
[&label=60.0]:0.042724)
[&label=100.0]:0.296037,'Thecc.08G129000.1.p':0.085963)
[&label=50.0]:0.153081,
(('Potri.002G081000.1.p':0.059273,'Potri.005G180200.1.p':0.051156)
[&label=99.0]:0.107423,
('Cp4.1LG02g02980.1':0.182739,'Cp4.1LG06g06180.1':0.091873)
[&label=100.0]:0.250738)[&label=45.0]:0.097858,(Bol019020:0.098886,
('Carub.0002s2434.1.p':0.085643,'AT1G77450.1':0.035435)
[&label=98.0]:0.044026)[&label=100.0]:0.706424,
('Cp4.1LG13g04550.1':0.081005,'Cp4.1LG01g20600.1':0.114011)
[&label=100.0]:0.312066,'VIT_218s0001g02300.1':0.207926,
((('AsparagusV1_09.665p':0.198617,((GSMUA_Achr6P18720_001:0.111745,
```

(GSMUA_Achr7P23250_001:0.324322,GSMUA_Achr10P04570_001:0.248361)
[&label=87.0]:0.05729)[&label=99.0]:0.186525,
('LOC_Os01g66120.1':0.027678,((('LOC_Os05g34830.1':0.137072,
('Seita.3G237100.1.p':0.064536,
('Sobic.009G142200.1.p':0.063241,Zm00001d038221_P001:0.071563)
[&label=52.0]:0.018435)[&label=100.0]:0.130487)
[&label=100.0]:0.170103,'Seita.5G405100.1.p':0.041079)
[&label=51.0]:0.020536,
('Sobic.003G379700.1.p':1.0E-6,Zm00001d042609_P001:0.043749)
[&label=81.0]:0.011706)[&label=65.0]:0.066052)[&label=98.0]:0.209418)
[&label=41.0]:0.031401)[&label=81.0]:0.221644,
('LOC_Os11g08210.1':0.156671,('Seita.8G060900.1.p':0.071778,
('Sobic.005G064600.2.p':0.030754,Zm00001d000112_P001:0.028585)
[&label=99.0]:0.066258)[&label=76.0]:0.067232)[&label=100.0]:0.733163)
[&label=88.0]:0.166862,('VIT_207s0031g02610.1':0.286808,
(((('AT5G63790.2':0.100428,'Carub.0008s2456.1.p':0.041827)
[&label=90.0]:0.087382,
(('Carub.0006s0776.1.p':0.055779,'AT5G08790.1':0.020861)
[&label=61.0]:0.018679,((Bol024596:0.098464,Bol008793:0.138)
[&label=39.0]:0.009782,Bol043763:0.044033)[&label=35.0]:0.017752)
[&label=93.0]:0.112096)[&label=100.0]:0.508974,
('Thecc.01G140500.1.p':0.166383,
(('Potri.005G069500.1.p':0.069317,'Potri.007G099400.2.p':0.052125)
[&label=99.0]:0.159235,((MD15G1136600:0.320421,
('Cp4.1LG05g15540.1':0.061981,
('Cp4.1LG00g11300.1':0.00187,'Cp4.1LG16g01180.1':0.039616)
[&label=100.0]:0.266568)[&label=100.0]:0.309744)
[&label=54.0]:0.056914,
('Medtr8g094580.1':0.252898,'Medtr3g096920.2':0.257531)
[&label=64.0]:0.121183)[&label=47.0]:0.041776)[&label=33.0]:0.043977)
[&label=61.0]:0.039681)[&label=35.0]:0.039159,(Cc02_g33930:0.261474,
(('Peaxi162Scf00842g00211.1':0.055863,(OIT05436:0.05623,
(('Solyc04g009440.3.1':0.006334,PGSC0003DMP400055618:0.027355)
[&label=100.0]:0.085781,CA05g04410:0.047505)[&label=93.0]:0.059842)
[&label=87.0]:0.034394)[&label=100.0]:0.196167,
('Migut.L00568.1.p':0.335343,
('Migut.H00825.1.p':0.060554,'Migut.H00826.1.p':0.043382)
[&label=100.0]:0.16437)[&label=64.0]:0.082484)[&label=70.0]:0.059517)
[&label=56.0]:0.093633,(DCAR_013176:0.272816,
((HanXRQChr12g0357431:0.142478,HanXRQChr09g0265531:0.095148)
[&label=100.0]:0.116593,
(HanXRQChr17g0547571:0.035669,HanXRQChr15g0474211:0.136465)
[&label=97.0]:0.085503)[&label=99.0]:0.154803)[&label=32.0]:0.079892)
[&label=37.0]:0.055106)[&label=81.0]:0.12352)[&label=26.0]:0.138701,
(((((((PGSC0003DMP400030689:0.027203,'Solyc06g060230.3.1':0.011367)
[&label=98.0]:0.013302,CA06g11310:0.065315)
[&label=99.0]:0.044445,OIT02385:0.012437)
[&label=82.0]:0.028111,'Peaxi162Scf00051g01410.1':0.020749)
[&label=94.0]:0.08588,(CA00g03050:0.091963,
(PGSC0003DMP400016317:0.007472,'Solyc11g017470.2.1':0.060082)

```
[&label=99.0]:0.055141)[&label=99.0]:0.20427)[&label=54.0]:0.05042,
('Migut.C01110.1.p':0.291259,Cc10_g12200:0.162396)
[&label=81.0]:0.076898)[&label=40.0]:0.026973,DCAR_016661:0.59807)
[&label=48.0]:0.097728,'AmTr_v1.0_scaffold00009.309p':0.796538,MD15G13
44900:0.231409,'Medtr3g088110.2':0.217031);
end;

begin figtree;
	set appearance.backgroundColorAttribute="Default";
	set appearance.backgroundColour=#ffffff;
	set appearance.branchColorAttribute="User selection";
	set appearance.branchColorGradient=false;
	set appearance.branchLineWidth=1.0;
	set appearance.branchMinLineWidth=0.0;
	set appearance.branchWidthAttribute="Fixed";
	set appearance.foregroundColour=#000000;
	set appearance.hilightingGradient=false;
	set appearance.selectionColour=#2d3680;
	set branchLabels.colorAttribute="User selection";
	set branchLabels.displayAttribute="Branch times";
	set branchLabels.fontName="sansserif";
	set branchLabels.fontSize=8;
	set branchLabels.fontStyle=0;
	set branchLabels.isShown=false;
	set branchLabels.significantDigits=4;
	set layout.expansion=0;
	set layout.layoutType="RECTILINEAR";
	set layout.zoom=0;
	set legend.attribute="label";
	set legend.fontSize=10.0;
	set legend.isShown=false;
	set legend.significantDigits=4;
	set nodeBars.barWidth=4.0;
	set nodeBars.displayAttribute=null;
	set nodeBars.isShown=false;
	set nodeLabels.colorAttribute="User selection";
	set nodeLabels.displayAttribute="label";
	set nodeLabels.fontName="sansserif";
	set nodeLabels.fontSize=8;
	set nodeLabels.fontStyle=0;
	set nodeLabels.isShown=true;
	set nodeLabels.significantDigits=4;
	set nodeShapeExternal.colourAttribute="User selection";
	set nodeShapeExternal.isShown=false;
	set nodeShapeExternal.minSize=10.0;
	set nodeShapeExternal.scaleType=Width;
	set nodeShapeExternal.shapeType=Circle;
	set nodeShapeExternal.size=4.0;
	set nodeShapeExternal.sizeAttribute="Fixed";
	set nodeShapeInternal.colourAttribute="User selection";
```

```
        set nodeShapeInternal.isShown=false;
        set nodeShapeInternal.minSize=10.0;
        set nodeShapeInternal.scaleType=Width;
        set nodeShapeInternal.shapeType=Circle;
        set nodeShapeInternal.size=4.0;
        set nodeShapeInternal.sizeAttribute="Fixed";
        set polarLayout.alignTipLabels=false;
        set polarLayout.angularRange=0;
        set polarLayout.rootAngle=0;
        set polarLayout.rootLength=100;
        set polarLayout.showRoot=true;
        set radialLayout.spread=0.0;
        set rectilinearLayout.alignTipLabels=true;
        set rectilinearLayout.curvature=0;
        set rectilinearLayout.rootLength=100;
        set scale.offsetAge=0.0;
        set scale.rootAge=1.0;
        set scale.scaleFactor=1.0;
        set scale.scaleRoot=false;
        set scaleAxis.automaticScale=true;
        set scaleAxis.fontSize=8.0;
        set scaleAxis.isShown=false;
        set scaleAxis.lineWidth=1.0;
        set scaleAxis.majorTicks=1.0;
        set scaleAxis.minorTicks=0.5;
        set scaleAxis.origin=0.0;
        set scaleAxis.reverseAxis=false;
        set scaleAxis.showGrid=true;
        set scaleBar.automaticScale=true;
        set scaleBar.fontSize=10.0;
        set scaleBar.isShown=true;
        set scaleBar.lineWidth=1.0;
        set scaleBar.scaleRange=2.0;
        set tipLabels.colorAttribute="User selection";
        set tipLabels.displayAttribute="Names";
        set tipLabels.fontName="sansserif";
        set tipLabels.fontSize=12;
        set tipLabels.fontStyle=0;
        set tipLabels.isShown=true;
        set tipLabels.significantDigits=4;
        set trees.order=false;
        set trees.orderType="increasing";
        set trees.rooting=false;
        set trees.rootingType="User Selection";
        set trees.transform=true;
        set trees.transformType="cladogram";
end;
```

RAxML_bipartitions_25coll.AtNIR1redux.mafft.trimal.tre

```
#NEXUS
begin taxa;
	dimensions ntax=27;
	taxlabels
	'AT2G15620.1'[&!color=#068006]
	Bol019359
	Bol031898
	CA00g41210
	CA02g10210
	CA10g07200
	'Carub.0003s2939.1.p'
	Cc02_g28520
	'Cp4.1LG14g04230.1'
	DCAR_029058
	HanXRQChr04g0128191
	HanXRQChr09g0259251
	MD05G1081500
	'Medtr4g086020.1'
	'Migut.B00351.1.p'
	OIT07237
	OIT32709
	PGSC0003DMP400014494
	PGSC0003DMP400044761
	'Peaxi162Scf00003g01630.1'
	'Peaxi162Scf00016g01734.1'
	'Potri.004G140800.1.p'
	'Potri.009G101601.1.p'
	'Solyc01g108630.3.1'[&!color=#800a10]
	'Solyc10g050890.2.1'[&!color=#800a10]
	'Thecc.02G101200.1.p'
	'VIT_203s0063g00370.1'
;
end;

begin trees;
	tree tree_1 = [&R] ((Bol019359:0.049855,
(('Potri.004G140800.1.p':0.034221,'Potri.009G101601.1.p':0.134117)
[&label=89.0]:0.012771,(('Thecc.02G101200.1.p':0.090095,
('Medtr4g086020.1':0.112089,MD05G1081500:0.105915)
[&label=63.0]:0.018182)[&label=47.0]:0.016895,
((((DCAR_029058:0.066707,
(HanXRQChr09g0259251:0.108271,HanXRQChr04g0128191:0.063594)
[&label=100.0]:0.069712)[&label=80.0]:0.030246,
((('Peaxi162Scf00003g01630.1':0.055699,
((('Solyc10g050890.2.1':0.019523,PGSC0003DMP400014494:0.00434)
[&label=96.0]:0.017184,(CA10g07200:0.010527,CA02g10210:0.025821)
[&label=100.0]:0.018236)[&label=55.0]:0.007906,OIT32709:0.027853)
```

```
[&label=38.0]:0.002341)[&label=100.0]:0.039235,
('Peaxi162Scf00016g01734.1':0.037358,(OIT07237:0.011434,
(CA00g41210:0.034902,
(PGSC0003DMP400044761:0.004272,'Solyc01g108630.3.1':0.004757)
[&label=96.0]:0.010233)[&label=65.0]:0.013645)[&label=45.0]:0.010229)
[&label=100.0]:0.063138)[&label=71.0]:0.035937,
('Migut.B00351.1.p':0.079479,Cc02_g28520:0.076844)
[&label=40.0]:0.010723)[&label=66.0]:0.016357)
[&label=56.0]:0.019701,'VIT_203s0063g00370.1':0.106515)
[&label=50.0]:0.011824,'Cp4.1LG14g04230.1':0.198953)
[&label=34.0]:0.007606)[&label=63.0]:0.032306)[&label=100.0]:0.132402)
[&label=75.0]:0.01683,
('AT2G15620.1':0.016155,'Carub.0003s2939.1.p':0.013718)
[&label=92.0]:0.022803,Bol031898:0.022573);
end;

begin figtree;
	set appearance.backgroundColorAttribute="Default";
	set appearance.backgroundColour=#ffffff;
	set appearance.branchColorAttribute="User selection";
	set appearance.branchColorGradient=false;
	set appearance.branchLineWidth=1.0;
	set appearance.branchMinLineWidth=0.0;
	set appearance.branchWidthAttribute="Fixed";
	set appearance.foregroundColour=#000000;
	set appearance.hilightingGradient=false;
	set appearance.selectionColour=#2d3680;
	set branchLabels.colorAttribute="User selection";
	set branchLabels.displayAttribute="Branch times";
	set branchLabels.fontName="sansserif";
	set branchLabels.fontSize=8;
	set branchLabels.fontStyle=0;
	set branchLabels.isShown=false;
	set branchLabels.significantDigits=4;
	set layout.expansion=0;
	set layout.layoutType="RECTILINEAR";
	set layout.zoom=0;
	set legend.attribute="label";
	set legend.fontSize=10.0;
	set legend.isShown=false;
	set legend.significantDigits=4;
	set nodeBars.barWidth=4.0;
	set nodeBars.displayAttribute=null;
	set nodeBars.isShown=false;
	set nodeLabels.colorAttribute="User selection";
	set nodeLabels.displayAttribute="label";
	set nodeLabels.fontName="sansserif";
	set nodeLabels.fontSize=8;
	set nodeLabels.fontStyle=0;
	set nodeLabels.isShown=true;
```

```
set nodeLabels.significantDigits=4;
set nodeShapeExternal.colourAttribute="User selection";
set nodeShapeExternal.isShown=false;
set nodeShapeExternal.minSize=10.0;
set nodeShapeExternal.scaleType=Width;
set nodeShapeExternal.shapeType=Circle;
set nodeShapeExternal.size=4.0;
set nodeShapeExternal.sizeAttribute="Fixed";
set nodeShapeInternal.colourAttribute="User selection";
set nodeShapeInternal.isShown=true;
set nodeShapeInternal.minSize=10.0;
set nodeShapeInternal.scaleType=Width;
set nodeShapeInternal.shapeType=Circle;
set nodeShapeInternal.size=4.0;
set nodeShapeInternal.sizeAttribute="Fixed";
set polarLayout.alignTipLabels=false;
set polarLayout.angularRange=0;
set polarLayout.rootAngle=0;
set polarLayout.rootLength=100;
set polarLayout.showRoot=true;
set radialLayout.spread=0.0;
set rectilinearLayout.alignTipLabels=false;
set rectilinearLayout.curvature=0;
set rectilinearLayout.rootLength=100;
set scale.offsetAge=0.0;
set scale.rootAge=1.0;
set scale.scaleFactor=1.0;
set scale.scaleRoot=false;
set scaleAxis.automaticScale=true;
set scaleAxis.fontSize=8.0;
set scaleAxis.isShown=false;
set scaleAxis.lineWidth=1.0;
set scaleAxis.majorTicks=1.0;
set scaleAxis.minorTicks=0.5;
set scaleAxis.origin=0.0;
set scaleAxis.reverseAxis=false;
set scaleAxis.showGrid=true;
set scaleBar.automaticScale=true;
set scaleBar.fontSize=10.0;
set scaleBar.isShown=true;
set scaleBar.lineWidth=1.0;
set scaleBar.scaleRange=0.0;
set tipLabels.colorAttribute="User selection";
set tipLabels.displayAttribute="Names";
set tipLabels.fontName="sansserif";
set tipLabels.fontSize=12;
set tipLabels.fontStyle=0;
set tipLabels.isShown=true;
set tipLabels.significantDigits=4;
set trees.order=false;
```

```
        set trees.orderType="increasing";
        set trees.rooting=false;
        set trees.rootingType="User Selection";
        set trees.transform=true;
        set trees.transformType="cladogram";
end;
```

RAxML_bipartitions_25coll.AtNLP7redux.mafft.trimal.tre

```
#NEXUS
begin taxa;
	dimensions ntax=51;
	taxlabels
	'AT1G64530.1'[&!color=#0c8008]
	'AT4G24020.1'[&!color=#0c8008]
	'AmTr_v1.0_scaffold00058.115p'
	'AmTr_v1.0_scaffold00080.66p'
	'AsparagusV1_01.2950p'
	Bol009587
	Bol042145
	CA01g00740
	CA01g34600
	CA08g17390
	CA10g15930
	'Carub.0002s0022.1.p'
	'Carub.0007s1707.1.p'
	Cc08_g16400
	'Cp4.1LG01g01380.1'
	'Cp4.1LG14g02590.1'
	DCAR_029869
	GSMUA_Achr3P03250_001
	GSMUA_Achr3P25240_001
	GSMUA_Achr8P04280_001
	GWHPAAYW003211
	GWHPAAYW019427
	HanXRQChr02g0047821
	HanXRQChr05g0133711
	HanXRQChr05g0133731
	HanXRQChr07g0189401
	'LOC_Os01g13540.1'
	MD06G1111900
	MD14G1133200
	'Medtr0022s0430.1'
	'Medtr1g100970.1'
	'Migut.E00644.1.p'
	OIS99570
	OIT38492
	PGSC0003DMP400010273
	PGSC0003DMP400021692
	'Peaxi162Scf00074g00543.1'
	'Peaxi162Scf00102g00189.1'
	'Potri.001G087900.1.p'
	'Potri.003G143000.1.p'
	'Seita.3G084600.1.p'
	'Seita.5G004100.1.p'
	'Sobic.003G003600.1.p'
```

```
		'Solyc08g008410.3.1'[&!color=#800917]
		'Solyc08g082750.3.1'[&!color=#800917]
		'Thecc.03G302500.1.p'
		'VIT_202s0025g02060.1'
		'VIT_202s0025g02070.1'
		Zm00001d009017_P002
		Zm00001d037786_P001
		Zm00001d039266_P004
;
end;

begin trees;
		tree tree_1 = [&R] ((('AsparagusV1_01.2950p':0.595552,
((((Zm00001d037786_P001:0.204352,'Seita.3G084600.1.p':0.125746)
[&label=100.0]:0.442075,('LOC_Os01g13540.1':0.091946,
('Seita.5G004100.1.p':0.046825,('Sobic.003G003600.1.p':0.019841,
(Zm00001d039266_P004:0.082434,Zm00001d009017_P002:0.032894)
[&label=78.0]:0.007521)[&label=100.0]:0.031594)[&label=100.0]:0.06076)
[&label=100.0]:0.078328)
[&label=100.0]:0.293945,GSMUA_Achr3P03250_001:0.539793)
[&label=93.0]:0.169641,
(GSMUA_Achr3P25240_001:0.237848,GSMUA_Achr8P04280_001:0.287131)
[&label=100.0]:0.267255)[&label=80.0]:0.053842)[&label=98.0]:0.151022,
((((DCAR_029869:0.3469,
((HanXRQChr05g0133711:0.0224,HanXRQChr05g0133731:0.00326)
[&label=100.0]:0.158637,
(HanXRQChr02g0047821:0.651206,HanXRQChr07g0189401:0.034745)
[&label=87.0]:0.043778)[&label=100.0]:0.336897)[&label=90.0]:0.058331,
(Cc08_g16400:0.344789,('Migut.E00644.1.p':0.328609,
(('Peaxi162Scf00102g00189.1':0.114959,(OIT38492:0.072914,
(CA01g34600:0.13387,
('Solyc08g008410.3.1':0.024837,PGSC0003DMP400010273:0.017224)
[&label=100.0]:0.051223)[&label=91.0]:0.020001)[&label=75.0]:0.01622)
[&label=100.0]:0.085502,
(((PGSC0003DMP400021692:0.010559,'Solyc08g082750.3.1':0.019507)
[&label=100.0]:0.034808,CA01g00740:0.046165)[&label=98.0]:0.025592,
(OIS99570:0.041292,'Peaxi162Scf00074g00543.1':0.074978)
[&label=65.0]:0.011681)[&label=100.0]:0.076868)
[&label=100.0]:0.123272)[&label=41.0]:0.01795)[&label=89.0]:0.051842)
[&label=91.0]:0.065258,
(((('Potri.003G143000.1.p':0.094952,'Potri.001G087900.1.p':0.094309)
[&label=100.0]:0.125534,
((('AT1G64530.1':0.040645,'Carub.0002s0022.1.p':0.048925)
[&label=100.0]:0.513152,('Carub.0007s1707.1.p':0.013404,
('AT4G24020.1':0.021912,(Bol009587:0.052982,Bol042145:0.057353)
[&label=29.0]:0.009428)[&label=52.0]:0.012067)[&label=100.0]:0.412482)
[&label=100.0]:0.159588,'Thecc.03G302500.1.p':0.133206)
[&label=82.0]:0.044293)[&label=89.0]:0.035047,
((MD06G1111900:0.063746,MD14G1133200:0.113298)[&label=100.0]:0.253981,
(('Medtr0022s0430.1':0.314531,'Medtr1g100970.1':0.238234)
```

```
[&label=100.0]:0.164452,
('Cp4.1LG01g01380.1':0.117824,'Cp4.1LG14g02590.1':0.07722)
[&label=100.0]:0.291395)[&label=40.0]:0.026054)[&label=94.0]:0.03937)
[&label=64.0]:0.035519,
('VIT_202s0025g02070.1':0.067937,'VIT_202s0025g02060.1':0.684855)
[&label=98.0]:0.109828)[&label=58.0]:0.048556)[&label=52.0]:0.090236,
(CA08g17390:0.09318,CA10g15930:0.414838)[&label=100.0]:1.62492)
[&label=76.0]:0.119627)[&label=69.0]:0.091021,
(GWHPAAYW003211:0.933944,
('AmTr_v1.0_scaffold00080.66p':0.986116,GWHPAAYW019427:1.730262)
[&label=96.0]:0.300767)
[&label=37.0]:0.084368,'AmTr_v1.0_scaffold00058.115p':0.517515);
end;

begin figtree;
	set appearance.backgroundColorAttribute="Default";
	set appearance.backgroundColour=#ffffff;
	set appearance.branchColorAttribute="User selection";
	set appearance.branchColorGradient=false;
	set appearance.branchLineWidth=1.0;
	set appearance.branchMinLineWidth=0.0;
	set appearance.branchWidthAttribute="Fixed";
	set appearance.foregroundColour=#000000;
	set appearance.hilightingGradient=false;
	set appearance.selectionColour=#2d3680;
	set branchLabels.colorAttribute="User selection";
	set branchLabels.displayAttribute="Branch times";
	set branchLabels.fontName="sansserif";
	set branchLabels.fontSize=8;
	set branchLabels.fontStyle=0;
	set branchLabels.isShown=false;
	set branchLabels.significantDigits=4;
	set layout.expansion=0;
	set layout.layoutType="RECTILINEAR";
	set layout.zoom=0;
	set legend.attribute="label";
	set legend.fontSize=10.0;
	set legend.isShown=false;
	set legend.significantDigits=4;
	set nodeBars.barWidth=4.0;
	set nodeBars.displayAttribute=null;
	set nodeBars.isShown=false;
	set nodeLabels.colorAttribute="User selection";
	set nodeLabels.displayAttribute="label";
	set nodeLabels.fontName="sansserif";
	set nodeLabels.fontSize=8;
	set nodeLabels.fontStyle=0;
	set nodeLabels.isShown=true;
	set nodeLabels.significantDigits=4;
	set nodeShapeExternal.colourAttribute="User selection";
```

```
set nodeShapeExternal.isShown=true;
set nodeShapeExternal.minSize=10.0;
set nodeShapeExternal.scaleType=Width;
set nodeShapeExternal.shapeType=Circle;
set nodeShapeExternal.size=4.0;
set nodeShapeExternal.sizeAttribute="Fixed";
set nodeShapeInternal.colourAttribute="User selection";
set nodeShapeInternal.isShown=false;
set nodeShapeInternal.minSize=10.0;
set nodeShapeInternal.scaleType=Width;
set nodeShapeInternal.shapeType=Circle;
set nodeShapeInternal.size=4.0;
set nodeShapeInternal.sizeAttribute="Fixed";
set polarLayout.alignTipLabels=false;
set polarLayout.angularRange=0;
set polarLayout.rootAngle=0;
set polarLayout.rootLength=100;
set polarLayout.showRoot=true;
set radialLayout.spread=0.0;
set rectilinearLayout.alignTipLabels=false;
set rectilinearLayout.curvature=0;
set rectilinearLayout.rootLength=100;
set scale.offsetAge=0.0;
set scale.rootAge=1.0;
set scale.scaleFactor=1.0;
set scale.scaleRoot=false;
set scaleAxis.automaticScale=true;
set scaleAxis.fontSize=8.0;
set scaleAxis.isShown=false;
set scaleAxis.lineWidth=1.0;
set scaleAxis.majorTicks=1.0;
set scaleAxis.minorTicks=0.5;
set scaleAxis.origin=0.0;
set scaleAxis.reverseAxis=false;
set scaleAxis.showGrid=true;
set scaleBar.automaticScale=true;
set scaleBar.fontSize=10.0;
set scaleBar.isShown=true;
set scaleBar.lineWidth=1.0;
set scaleBar.scaleRange=0.0;
set tipLabels.colorAttribute="User selection";
set tipLabels.displayAttribute="Names";
set tipLabels.fontName="sansserif";
set tipLabels.fontSize=10;
set tipLabels.fontStyle=0;
set tipLabels.isShown=true;
set tipLabels.significantDigits=4;
set trees.order=false;
set trees.orderType="increasing";
set trees.rooting=false;
```

```
        set trees.rootingType="User Selection";
        set trees.transform=true;
        set trees.transformType="cladogram";
end;
```

SnapGeneÆ{"aligners":[{"name":"MUSCLE","options":{"-clw":"true"}}],"alignments":
[{"name":"MUSCLE","rawAlignment":0}],"created":"2025-05-01T07:11:36Z",
"description":"","modified":"2025-05-01T07:11:36Z","profiles":
[],"seqType":"prot","sequenceIDs":[0,1,2],"sequences":
[{"ID":0,"name":"SlNLP7A","use":true},
{"ID":1,"name":"SlNLP7B","use":true},
{"ID":2,"name":"SlNLP7C","use":true}],"uuid":"942029e4-bb20-46ef-9e03-b493a28c9a20","version":"1"}
AÙMSEPEEEMNFIFRSKPKDFVHPPPATAAAAQQQQHAVGENHRDSLMMDLDLDLDASWSFDQIFAAAAS
ASNPMSPFLVSAASEQPCSPLWAFSDENEDKPNGNALSTGSLRLSNYPRFVTYANEHEAAPETVSVTDDK
KRIPPPIKGLAPLDYLDSSCIIKERMTQALRYFKESTGERVLAQVWAPVKNGGRYVLTTSGQPFVLDPDC
NGLHQYRMVSLMYMFSVDGETDGVLGLPGRVYRKKLPEWTPNVQYYSSKEFPRLNHALDYNVRGTLALPV
FEPSGQSCVGVLELIMTSQKINYAPEVDKVCKALEAVNLKSSEILDYPNHQICNEGRQNALVEILEILTA
VCETYKLPLAQTWVPCRHRSVLADGGGFKKSCSSFDGSCMGQVCMSTTDVAFYVVDAHMWGFREACAEHH
LQKGQGVAGRAYASQKSCFCEDIGKFCKTEYPLVHYARLFGLSRCFAICLRSTHTGNDDYILEFFLPPND
GDYTDQLALLNSLLLTMKQHFRSLRVASGEELEHDWGSVEIIKASTEEKLGSRFDSVPTTKSLPQSASVA
NGRRHPDLMEEQHSTVAKGAEGVNVTAEAHNHASVPQNKQTGKKSERKRGKAEKTISLEVLQQYFAGSLK
DAAKSLGVCPTTMKRICRQHGISRWPSRKINKVNRSLSKLKRVIESVQGADGTFSLTSLAPNSLPVAVGS
ISWPAGINGSPCKASEYQEEKNEFSNHGTPGSHEEAEPTDQMLGSRIIGNEELSPKLNGFVREGSHRSRT
GSFSREESTGTPTSHGSCQGSPSPANESSPQNELLNSPTQESVMKVEGSLEPARQTTGELNLSTAFLMPG
LFIPEHTHQQFRGMLVEDAGSSHDLRNLCPAGETMFDERVPEYSWTNPPCSNGIATNQVPLPVEKMPQFS
SRPEVTSVTIKATYREDIIRFRLCLNSGIYKLKEEVSKRLKLEMGTFDIKYLDDDHEWVLIACDADLQEC
IDISSSSGSNVVRLLVHDIMPNLGSSCESSGE*?<Notes>
<UUID>df46457a-a30b-4937-965a-5571616b1f06</UUID>
<Type>Synthetic</Type>
<ConfirmedExperimentally>0</ConfirmedExperimentally>
<Created UTC="7:10:42">2025.5.1</Created>
<LastModified UTC="7:10:42">2025.5.1</LastModified>
<SequenceClass>UNA</SequenceClass>
<TransformedInto>unspecified</TransformedInto>
</Notes>
?
ÚMSEPGGGMTQNHLPKSKELTPATVTERESMMMDLDFDIDASWSFDQIFAAAAAVSSNPASPFLPCSPLW
AFPDDNDEKPAGNGLSGALRISGHPRFVAYTGDLEATTETISVNTDKGRLTSPISGLLPGDNPEGSCIIK
ERMTQALRYLKETSGERVLAQVWAPVKEAGRSVLTTSGQPFVLDPECNGLHQYRTVSLMYMFAADGETDG
VLGLPGRVFRLKLPEWTPNVQYYSSKEFPRLDHALNYNVRGTLALPVFEPSGRSCVGVLELIMTSQKINY
AAEVDKVCKALEAVNLKSSDILDHPNTQVYVMGYMNQICNEGRQNALVDILEILTAVCETYKLPLAQTWV
PCRHRSVLADGGGLRKSCSSFDGSCMGQICMSTTDVAFYVVDAHMWGFRDACAEHHLQRGQGVAGRAYAS
RKSCYCEDITQFCKTEYPLVHYARMFGLTSCFAICLRSSHTANDDYILEFFLPPNSGDYSDQPALLNSLL
LTMKQHFRSLSIASGEELEHDWGSVEIIQASMEEKIDAKPESVPTAKTSPQLTSLPNGWVHLDPVGEQQS
AVGSNVSKGARSTSGTGEAPNNVSNSDNKTSGKKSERKRGKAEKTISLEVLQQYFAGSLKDAAKSLGVCP
TTMKRICRQHGISRWPSRKINKVNRSLSKLKCVIESVQGAEGAFTLTSLAPNSLPAAVSSISWPAGANVS
NLPSSPSSKPSVFPEEKNEFFHHGTPESHIEAEPSNQMLGGRVARKEEFTPMQNGFLHAEGTHKSRTGSV
SREESAGTPTSHGSCQGSPCAGNGFSPQNELVNSPAHESCMKVGGSLEAARQTTAEINLSSAFLMPQPII
PKHTQEPFGGMLVEDAGSSHDLRNLCSPRDALVDERVPDYNLTNPPFSDAIAKDPVYVPPDTIQQYSAWP
EVTSVTIKATYKEDIIRFRLCLSSGIVKLKEEVAKRLKLELGTFYIKYLDDDLEFVPISCDADLQECVDI
SRSSGSSIVRLLIHDIMSNLGSSCESSGK*?<Notes>
<UUID>f55ad156-36e2-4602-a524-220b2aba21e7</UUID>
<Type>Synthetic</Type>

<ConfirmedExperimentally>0</ConfirmedExperimentally>
<Created UTC="7:10:42">2025.5.1</Created>
<LastModified UTC="7:10:42">2025.5.1</LastModified>
<SequenceClass>UNA</SequenceClass>
<TransformedInto>unspecified</TransformedInto>
</Notes>
…|
VKEKIKLALQRIETSQVILLQFWGLENIEGRKFLSTSGQPFGLRYLYKGLCWYRKHCQGYKYSVDKGENH
EQGMEKTHLFGPPTRVFQQKLPESSTHVGYYTNEEFPMRDHVLQCGVRTYLALPVFEPVDKNCIGVIELV
TVWKGGYLTCEVERVLNPLEAVDLKCPKIFLNKDRKVQAEKYNEGEEFKRMLKIVRETHKLPFIRVWIPC
VNLEMDHNGMYVGCTELAMSASNEVYFVADEEMDANDHVYDDYYYDDMLCFRDISKLQPLQKDQGVVGKA
FSSGKLCYCTNITEFSIIEYPLVHYARWCGLTTSFAICLKSRDDAYILELFLPPDNGDPNMLLGSILTTM
GQHFQNFKFASGQELGNDSSVQVVKASSDKNVDYFHIYQTRDSSFMPEVLHVEDSRNQLMEGNENHMEKK
EDRKQSMVQSNVLSIDSHVISEKQCVSVSHLQKESRTRTKDSVNYDDLKQHFDKNLSDAAKSLQISRSTL
KRLCRKYGIRRWPLSKRKKSSESDQQTLTKVSKKNKSIGSETTTQKDHESCSSSFTVKATFGDDMMKFKL
YTFSRKDDLDNEVSKRLKLPIGRFRINYMDEDNDWIWIACDDDLSDCFNNAQSLGNNTIKMLVLPAAINH
HFEL*?<Notes>
<UUID>0eb00c1a-1bcb-4ae7-b65e-7c97b2bf1a0c</UUID>
<Type>Synthetic</Type>
<ConfirmedExperimentally>0</ConfirmedExperimentally>
<Created UTC="7:10:42">2025.5.1</Created>
<LastModified UTC="7:10:42">2025.5.1</LastModified>
<SequenceClass>UNA</SequenceClass>
<TransformedInto>unspecified</TransformedInto>
</Notes>

ë*!-------------------------------------------------------------------
----------------------------------------------------------------------
-----------------------VKEKIKLALQRIE--
TSQVILLQFWGLENIEGRKFLSTSGQPFGLRYLYKGLCWYRKHCQGYKYSVDKGENHEQGMEKTHLFGPP
TRVFQQKLPESSTHVGYYTNEEFPMRDHVLQCGVRTYLALPVFEPVDKNCIGVIELVTVWKGGYLTCEVE
RVLNPLEAVDLKCPKIFLNKDRKV-----QAEKYNEGE-----
EFKRMLKIVRETHKLPFIRVWIPCVNLEM--DHNGMYVGCT--------
ELAMSASNEVYFVADEEMDANDHVYDDYYYDDMLCFRDISKLQPLQKDQGVVGKAFSSGKLCYCTNITEF
SIIEYPLVHYARWCGLTTSFAICLKSR---DDAYILELFLPPDNG---
DPNMLLGSILTTMGQHFQNFKFASGQELGND-
SSVQVVKASSDKNVDYFHIYQTRDSSFMPEVLHVEDSRNQLMEGNENHMEKKEDRKQSMVQSNVLSIDSH
VISEKQCVSVSHLQKESRTRTKDSVNYDDLKQHFDKNLSDAAKSLQISRSTLKRLCRKYGIRRWPLSKRK
KSS-------------------------------------------------------------------
--------------------------------------
ESDQQTLTKVSKKNKSIGSETT---------------------
TQKDHESC--------------------------------------------------------------
---------------------------------------
SSSFTVKATFGDDMMKFKLYTFSRKDDLDNEVSKRLKLPIGRFRINYMDEDNDWIWIACDDDLSDCFNNA
QSLGNNTIKMLV------LPAAINHHFEL*!
MSEPEEEMNFIFRSKPKDFVHPPPATAAAAQQQQHAVGENHRDSLMMDLDLDLDASWSFDQIFAAAAS-
ASNPMSPFLVSAASEQPCSPLWAFSDENEDKPNGNALSTGSLRLSNYPRFVTYANEHEAAPETVSVTDDK
KRIPPPIKGLAPLDYLDSSCIIKERMTQALRYFKESTGERVLAQVWAPVKNGGRYVLTTSGQPFVLDPDC
NGLHQYRMVSLMYMFSVDG--------
ETDGVLGLPGRVYRKKLPEWTPNVQYYSSKEFPRLNHALDYNVRGTLALPVFEPSGQSCVGVLELIMTSQ

KINYAPEVDKVCKALEAVNLKSSEILDYPNHQI---------
CNEGRQNALVEILEILTAVCETYKLPLAQTWVPCRHRSVLADGGGFKKSCSSFDGSCMGQVCMSTTDVAF
YVVDAHMWG---------------
FREACAEHHLQKGQGVAGRAYASQKSCFCEDIGKFCKTEYPLVHYARLFGLSRCFAICLRSTHTGNDDYI
LEFFLPPNDGDYTDQLALLNSLLLTMKQHFRSLRVASGEELEHDWGSVEIIKASTEEKLGS-
RFDSVPTTKSLPQSASVANGRRHPDLMEEQH----
STVAKGAEGVNVTAEAHNHASVPQNKQTGKKSERKRGKAEKTISLEVLQQYFAGSLKDAAKSLGVCPTTM
KRICRQHGISRWPSRKINKVNRSLSKLKRVIESVQGADGTFSLTSLAPNSLPVAVGSISWPAGIN-----
-GSPCKASEYQEEKNEFSNHGTPGSHEEAEPTDQMLGSRIIGNEELSPKLNGFVR-
EGSHRSRTGSFSREESTGTPTSHGSCQGSPSPANESSPQNELLNSPTQESVMKVEGSLEPARQTTGELNL
STAFLMPGLFIPEHTHQQFRGMLVEDAGSSHDLRNLCPAGETMFDERVPEYSWTNPPCSNGIATNQVPLP
VEKMPQFSSRPEVTSVTIKATYREDIIRFRLCLNSGIYKLKEEVSKRLKLEMGTFDIKYLDDDHEWVLIA
CDADLQECIDISSSSGSNVVRLLVHDIMPNLGSSCESSGE-*!MSEPGGGMTQNHLPKSKELT---
PATVT------------ERESMMMDLDFDIDASWSFDQIFAAAAAVSSNPASPFL-------
PCSPLWAFPDDNDEKPAGNGLS-
GALRISGHPRFVAYTGDLEATTETISVNTDKGRLTSPISGLLPGDNPEGSCIIKERMTQALRYLKETSGE
RVLAQVWAPVKEAGRSVLTTSGQPFVLDPECNGLHQYRTVSLMYMFAADG--------
ETDGVLGLPGRVFRLKLPEWTPNVQYYSSKEFPRLDHALNYNVRGTLALPVFEPSGRSCVGVLELIMTSQ
KINYAAEVDKVCKALEAVNLKSSDILDHPNTQVYVMGYMNQICNEGRQNALVDILEILTAVCETYKLPLA
QTWVPCRHRSVLADGGGLRKSCSSFDGSCMGQICMSTTDVAFYVVDAHMWG---------------
FRDACAEHHLQRGQGVAGRAYASRKSCYCEDITQFCKTEYPLVHYARMFGLTSCFAICLRSSHTANDDYI
LEFFLPPNSGDYSDQPALLNSLLLTMKQHFRSLSIASGEELEHDWGSVEIIQASMEEKIDA-
KPESVPTAKTSPQLTSLPNGWVHLDPVGEQQSAVGSNVSKGARSTSGTGEAPNNVSNSDNKTSGKKSERK
RGKAEKTISLEVLQQYFAGSLKDAAKSLGVCPTTMKRICRQHGISRWPSRKINKVNRSLSKLKCVIESVQ
GAEGAFTLTSLAPNSLPAAVSSISWPAGANVSNLPSSPSSKPSVFPEEKNEFFHHGTPESHIEAEPSNQM
LGGRVARKEEFTPMQNGFLHAEGTHKSRTGSVSREESAGTPTSHGSCQGSPCAGNGFSPQNELVNSPAHE
SCMKVGGSLEAARQTTAEINLSSAFLMPQPIIPKHTQEPFGGMLVEDAGSSHDLRNLCSPRDALVDERVP
DYNLTNPPFSDAIAKDPVYVPPDTIQQYSAWPEVTSVTIKATYKEDIIRFRLCLSSGIVKLKEEVAKRLK
LELGTFYIKYLDDDLEFVPISCDADLQECVDISRSSGSSIVRLLIHDIMSNLGSSCESSGK-
