# Supplementary material for: Conservation and divergence of regulatory architecture in nitrate-responsive plant gene circuits
Source: Plant Cell. 2025 May 22;37(6):koaf124. doi: 10.1093/plcell/koaf124 (PMC12205479; doi:10.1093/plcell/koaf124)
Supplement: koaf124_Supplementary_Data [file koaf124_supplementary_data.zip › CombinedSupplementalData_04_02_2025_REPLACEMENT.pdf]

Supplementary Figure S1. Logoplots of position weight matrices used to identify candidate binding sites. Nucleotides are colored according to their identity. Supports Figure 2.

<table>
<tr><th>TF</th><th>Gene ID</th><th>Family</th><th>PWM logo and data source</th></tr>
<tr><td>NLP7</td><td>AT4G24020</td><td>RW-PRK</td><td rowspan="2">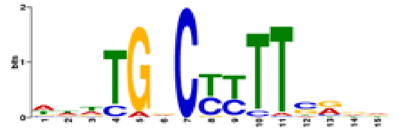
NLP7 (O’Malley et al 2016)</td></tr>
<tr><td>NLP6</td><td>AT1G65430</td><td>RW-PRK</td></tr>
<tr><td>ARF9</td><td>AT4G23980</td><td>ARF</td><td rowspan="2">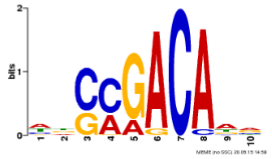
ARF2 (AT5G62000)
(O’Malley et al 2016)</td></tr>
<tr><td>ARF18</td><td>AT3G61830</td><td>ARF</td></tr>
<tr><td>ANAC032</td><td>AT1G77450</td><td>NAC</td><td>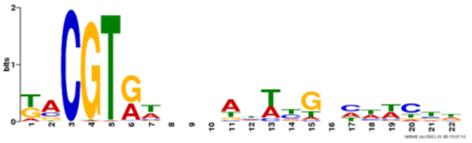
ATAF1 (AT1G01720)</td></tr>
<tr><td>DREB26</td><td>AT1G21910</td><td>ERF</td><td>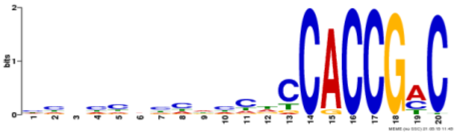
DREB 26 (O’Malley et al 2016)</td></tr>
</table>

Supplementary Figure S2.
Alignments of the DNA binding domains of Arabidopsis transcription factors. Grey highlight indicates divergent amino acids. Alignment files are present in Supplementary File S2. Supports Figure 2.

Alignment of DNA binding domains of AtNLP6 and AtNLP7

```
AtNLP7        ----KKKTEKKRGKTEKTISLDVLQQYFTGSLKDAAKSLGVCPTTMKRICRQHGISRWPS
AtNLP6        EAKTVKKSERKRGKTEKTISLEVLQQYFAGSLKDAAKSLGVCPTTMKRICRQHGISRWPS

AtNLP7        RKIKKVNRSITKLKRVIESVQGTDGG
AtNLP6        RKINKVNRSLTRLKHVIDSVQGADGS
```

Alignment of DNA binding domains of AtARF9 and AtARF18 and AtARF2

```
AtARF9        FSKVLTASDTSTHGGFSVLRKHATECLPPLDMTQQTPTQELVAEDVHGYQWKFKHIFRGQ
AtARF18       FVKILTASDTSTHGGFSVLRKHATECLPSLDMTQATPTQELVTRDLHGFEWRFKHIFRGQ
AtARF2        FCKTLTASDTSTHGGFSVLRRHADECLPPLDMSRQPPTQELVAKDLHANEWRFRHIFRGQ

AtARF9        PRRHLLTTGWSTFVTSKRLVAGDTFVFLRGENGELRVGVRRAN
AtARF18       PRRHLLTTGWSTFVSSKRLVAGDAFVFLRGENGDLRVGVRRLA
AtARF2        PRRHLLQSGWSVFVSSKRLVAGDAFIFLRGENGELRVGVRRAM
```

Alignment of DNA binding domains of AtANAC032 and ATAF1

```
ANAC032       FPPGFRFHPTDEELVLMYLCRKCASQPIPAPIITELDLYRYDPWDLPDMALYGEKEWYFF
ATAF1         LPPGFRFHPTDEELVMHYLCRKCASQSIAVPIIAEIDLYKYDPWELPGLALYGEKEWYFF

ANAC032       SPRDRKYPNGSRPNRAAGTGYWKATGADKPIGRPKPVGIKKALVFYSGKPPNGEKTNWIM
ATAF1         SPRDRKYPNGSRPNRSAGSGYWKATGADKPIGLPKPVGIKKALVFYAGKAPKGEKTNWIM

ANAC032       HEYRLADVDRSVR-KKNSLRLDDWVLCRIYNK
ATAF1         HEYRLADVDRSVRKKKNSLRLDDWVLCRIYNK
```

Supplementary Figure S3. *In vitro* binding assays showing relative binding of transcription factors to target gene-derived probes. Within the long rectangle, brown indicates an exon, grey an intron, yellow a non-coding transcript. On top of the rectangle, chromatin accessibility peaks are demonstrated for the root (grey with red border) and shoot (blue with blue border). Probes targeting candidate binding sites are indicated in dark orange (ANAC032), green (NLP6/7), blue (ARF9/18), light orange (DREB26) skinny rectangles within the gene model. At the bottom of the gene model, the probe designators are described with orange horizontally displayed rectangles and correspond to the probe binding in the graphs below. Note: this dataset includes the three panels shown in Main Figure 2.

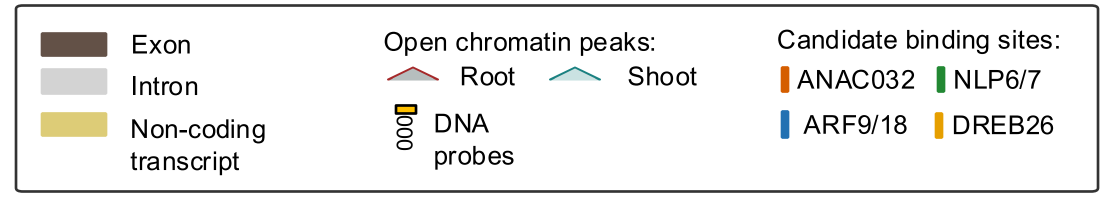

Relative binding of AtDREB26, AtARF18 and AtANAC032 for candidate sites in *AtANAC032*. Error bars = mean and standard deviation; n=3; P-values were calculated using an unpaired two-tailed Student's t-test of each sample to the random (rnd) control probe; *p<0.05, ** p<0.01, *** p<0.001; ns = not significant.

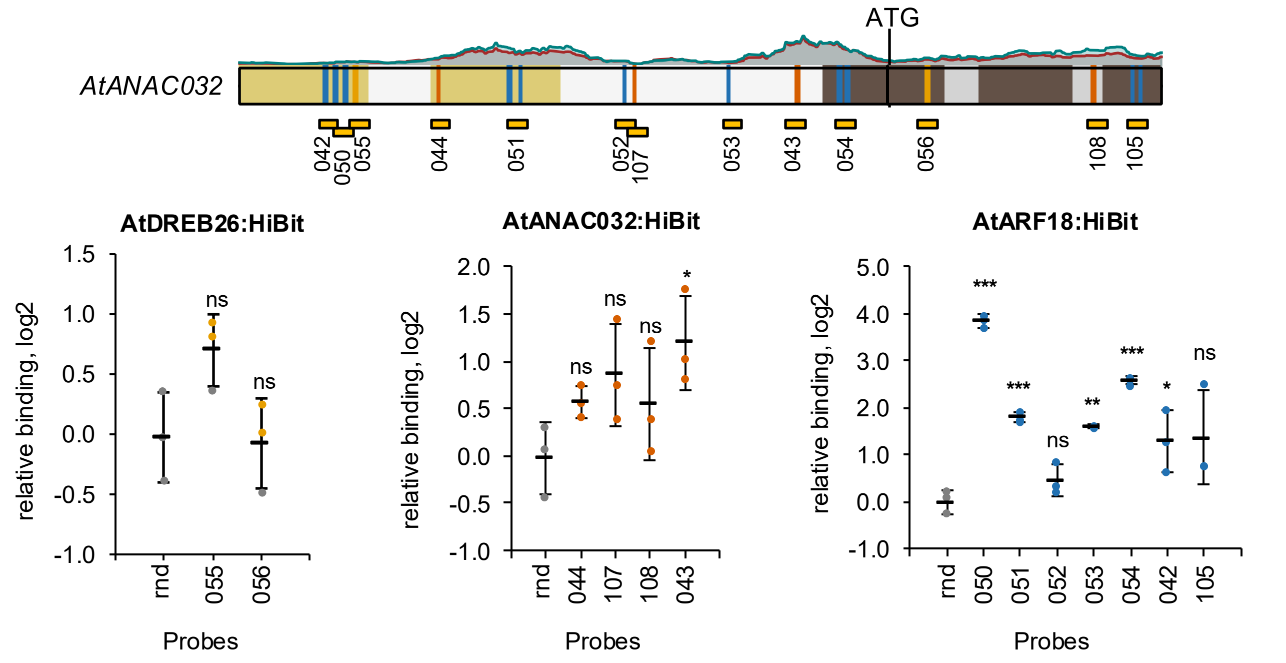

Relative binding of AtNLP7, ATNLP6 and AtARF18 for candidate sites in *AtARF18*. Error bars = mean and standard deviation; n=3; P-values were calculated using an unpaired two-tailed Student's t-test of each sample to the random (rnd) control probe; *p<0.05, ** p<0.01, *** p<0.001; ns = not significant.

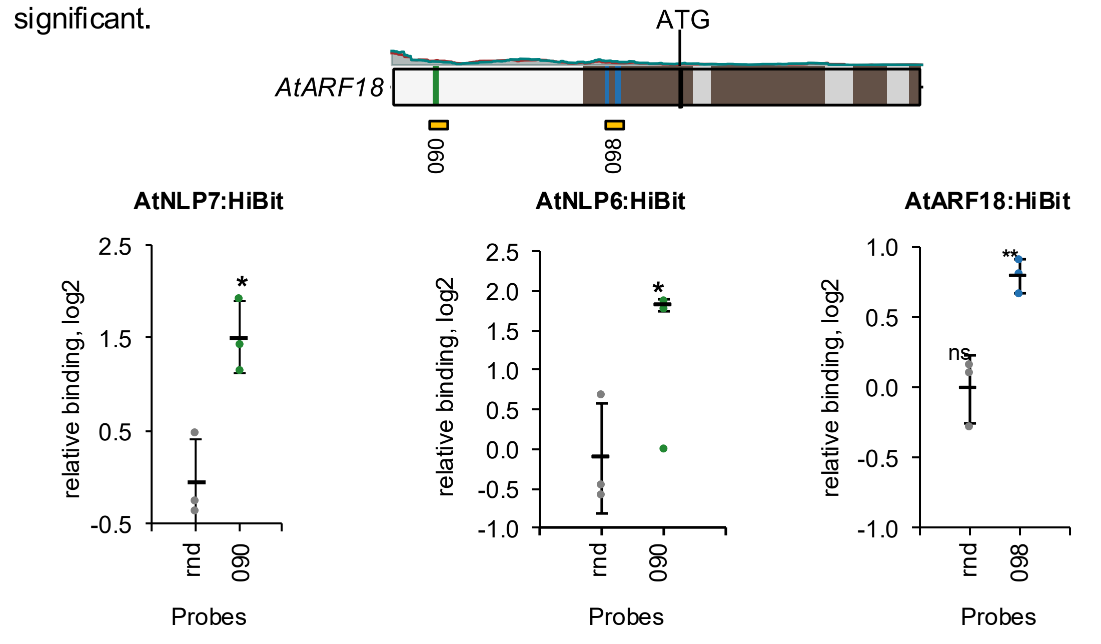

Relative binding of AtNLP7, ATNLP6, AtDREB26, AtARF18 and AtANAC032 for candidate sites in *AtDREB26*. Error bars = mean and standard deviation; n=3; P-values were calculated using an unpaired two-tailed Student's t-test of each sample to the random (rnd) control probe; *p<0.05, ** p<0.01, *** p<0.001; ns = not significant.

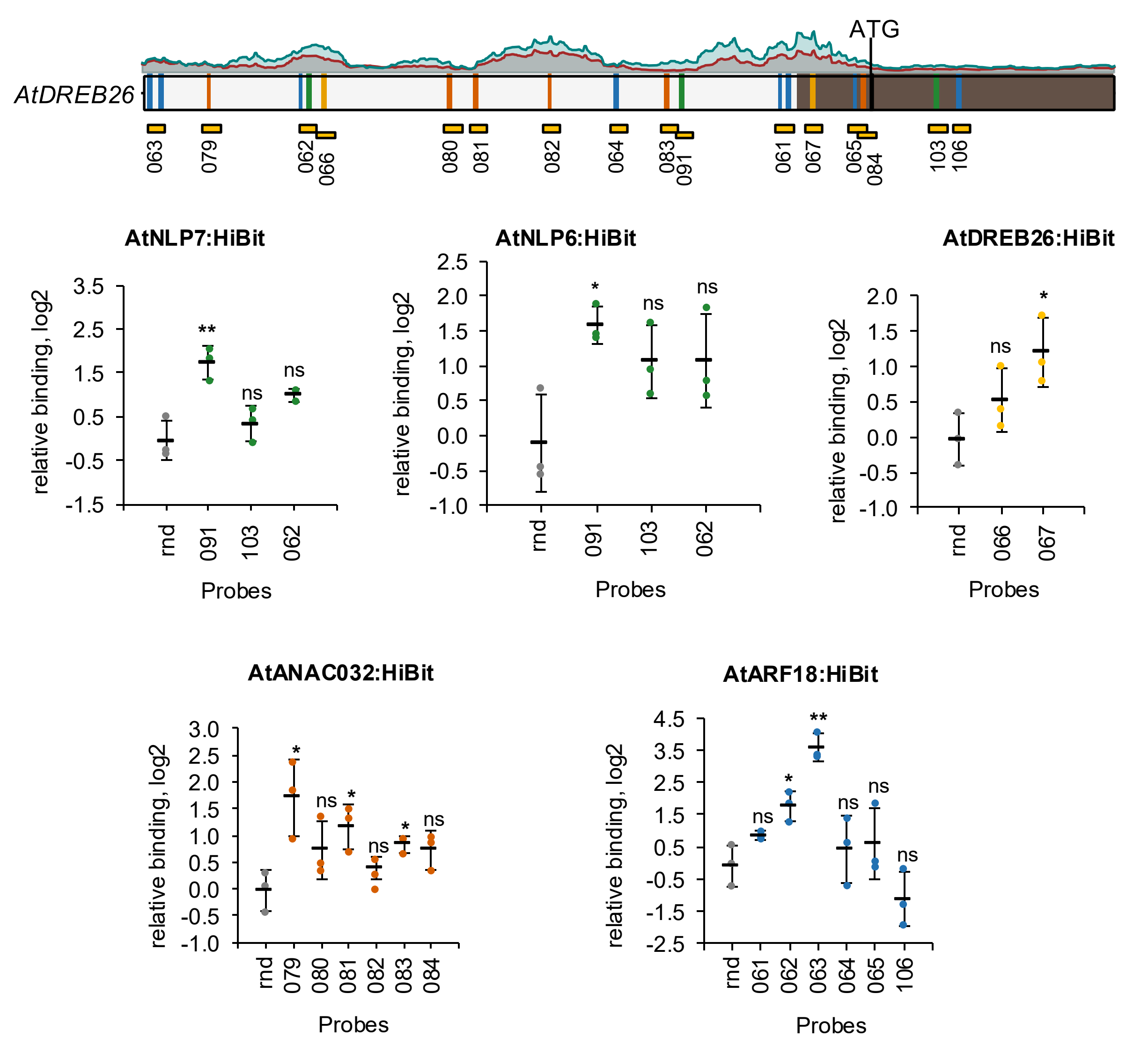

Relative binding of AtNLP7, ATNLP6, AtDREB26, AtARF18 and AtANAC032 for candidate sites in *AtNIR1*. Error bars = mean and standard deviation; n=3; P-values were calculated using an unpaired two-tailed Student's t-test of each sample to the random (rnd) control probe; *p<0.05, ** p<0.01, *** p<0.001; ns = not significant. Supports Figure 2.

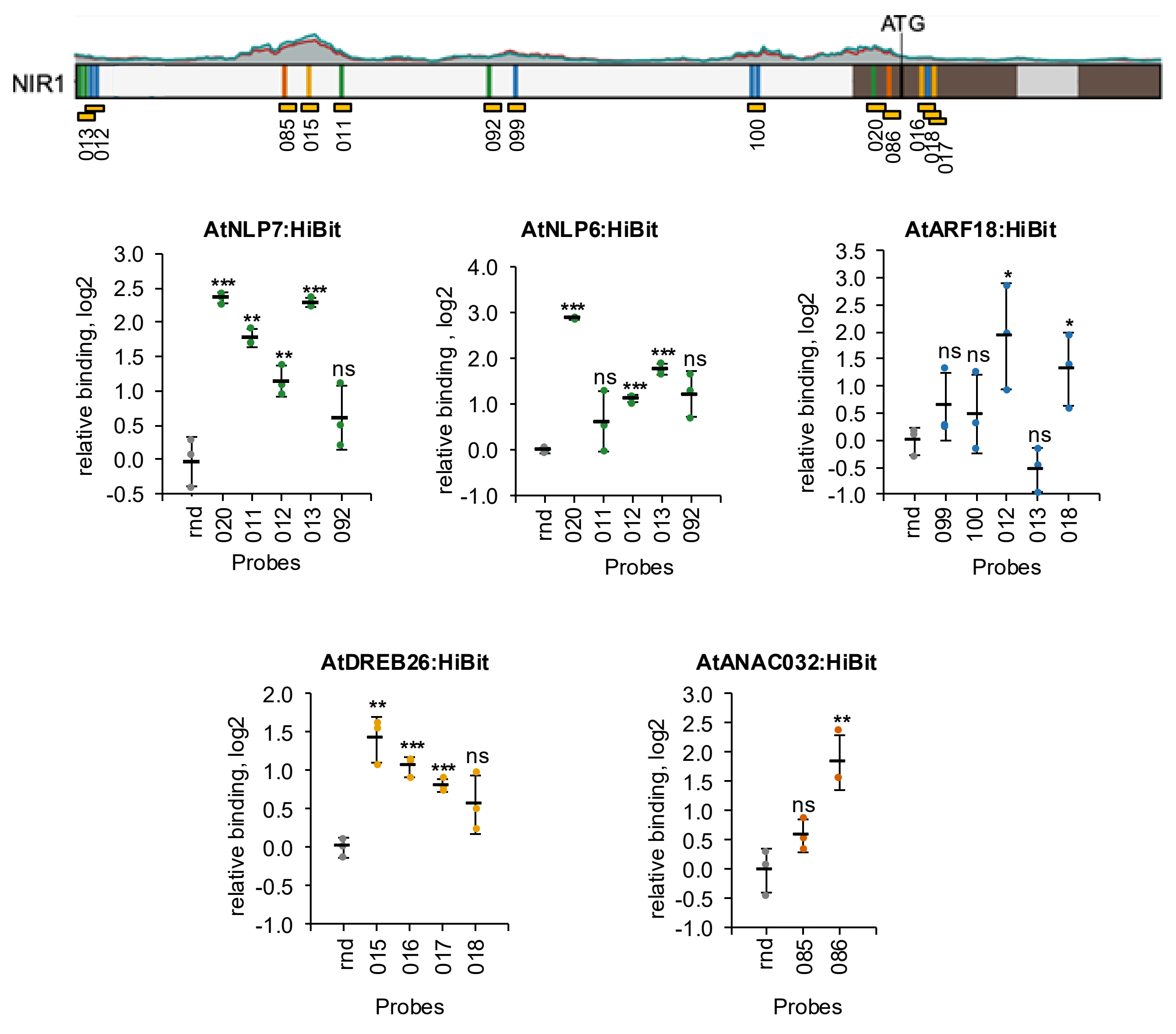

Relative binding of AtNLP7, ATNLP6, AtDREB26, AtARF18 and AtANAC032 for candidate sites in *AtNLP6*. Error bars = mean and standard deviation; n=3; P-values were calculated using an unpaired two-tailed Student's t-test of each sample to the random (rnd) control probe; *p<0.05, ** p<0.01, *** p<0.001; ns = not significant.

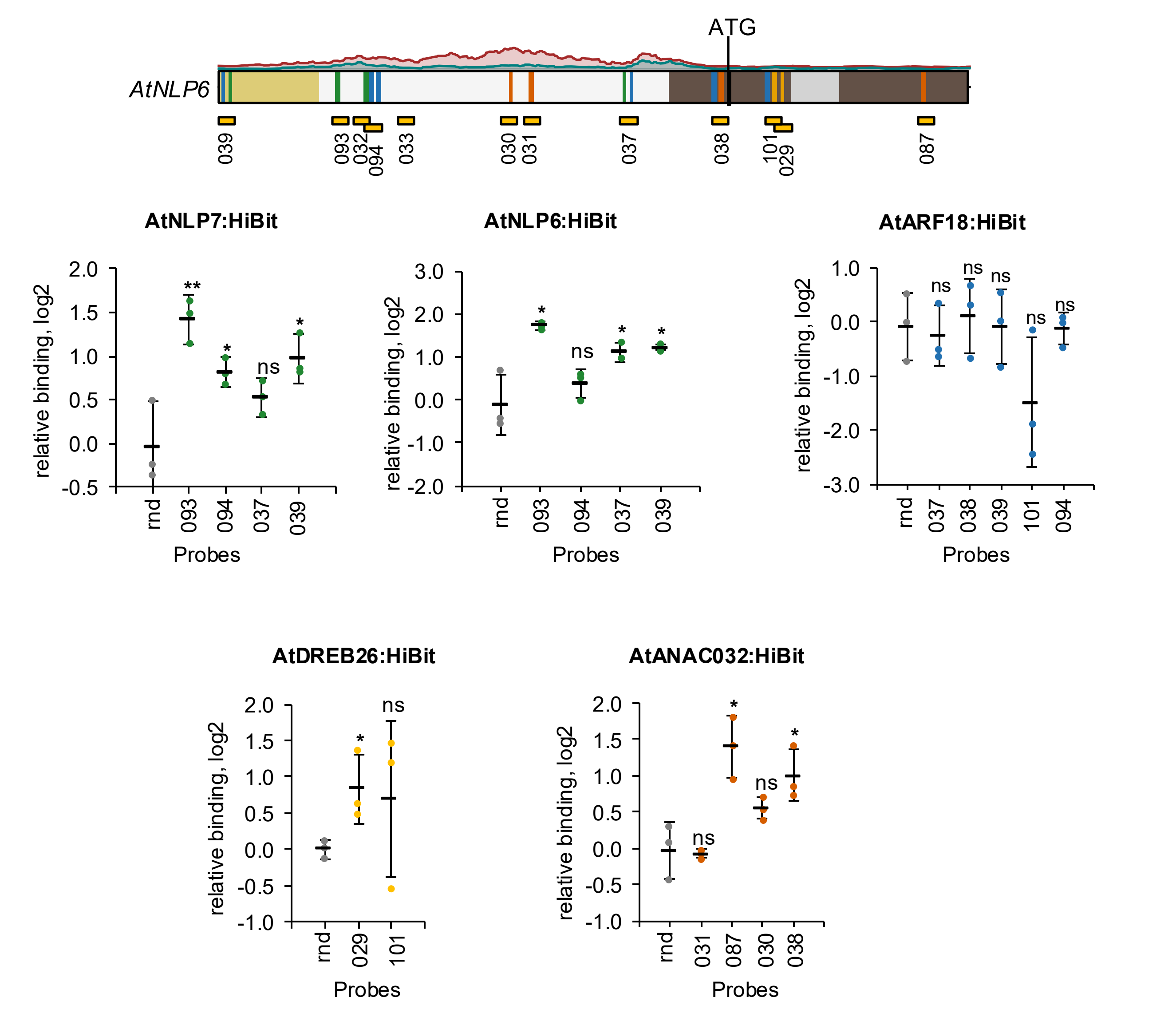

Relative binding of AtNLP7, ATNLP6, AtDREB26, AtARF18 and AtANAC032 for candidate sites in *AtNLP7*. Error bars = mean and standard deviation; n=3; P-values were calculated using an unpaired two-tailed Student's t-test of each sample to the random (rnd) control probe; *p<0.05, ** p<0.01, *** p<0.001; ns = not significant.

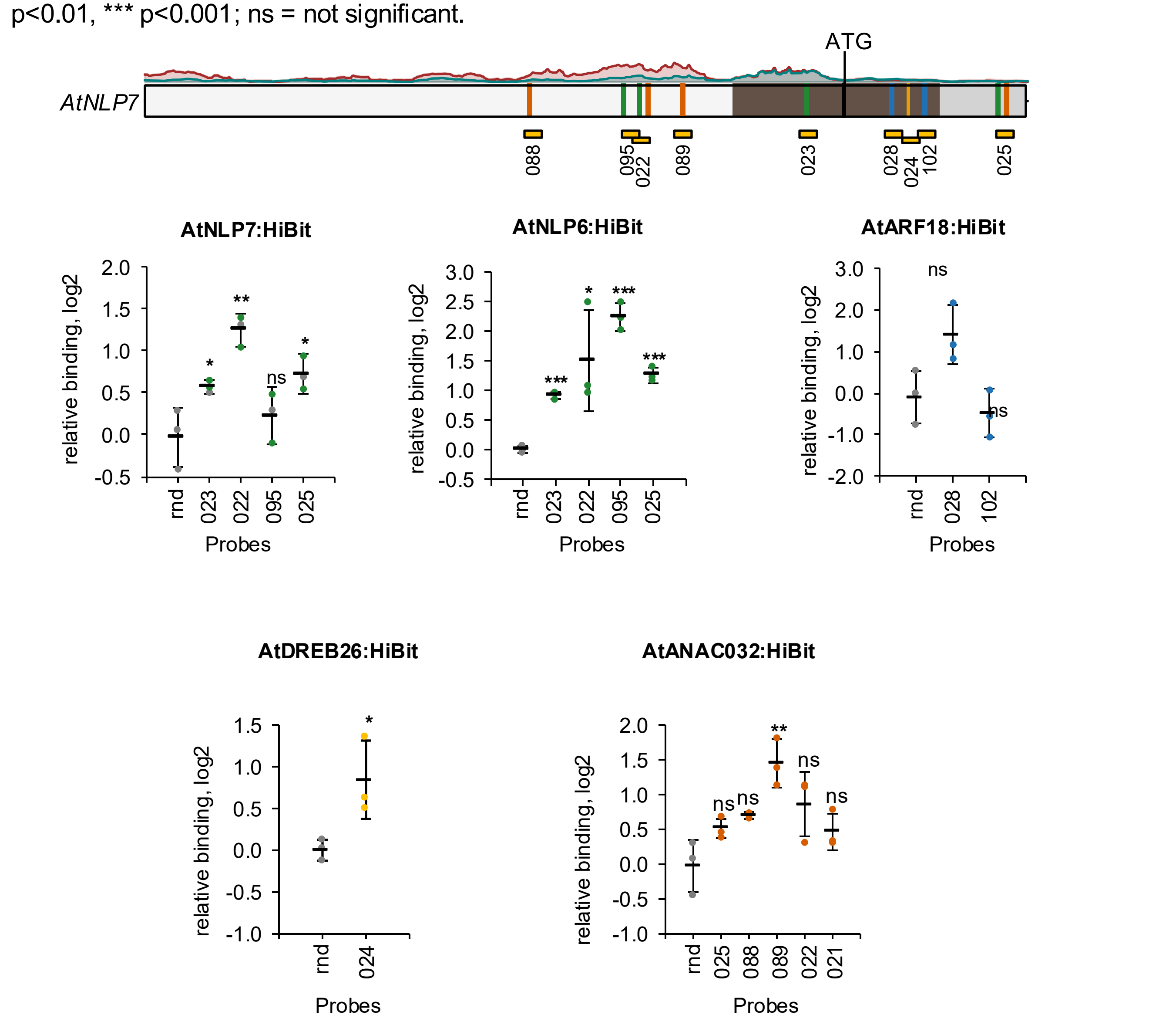

Supplementary Figure S4. Methodology for protoplast transactivation assays. To assess changes in luminescence resulting from each of the promoters tested (pTest, bent arrow) in response to the expression of a given transcription factor (TF, pentagon) with a terminator (T), luminescence values relative to an experiment calibrator (EC) are compared from samples with and without plasmids expressing each TF. To maintain equal transcriptional loads, in the absence of the TF, a control plasmid expressing Yellow Fluorescent Protein (YFP) from the same promoter is included. The relative value for pTest is then normalized to a batch calibrator (BC) to account for variation between protoplast batches providing a normalized value in arbitrary units (a.u.). Transactivation assays were performed in three biological replicates of independent protoplast transfections (*N* = 3), and luminescence was quantified in three technical replicates for each assay. Supports Figure 3.

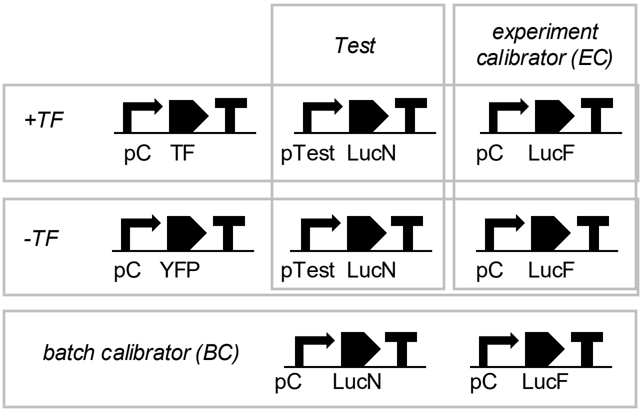

$$\text{Normalized luminescence (a.u.)} = \frac{Test_{LucN}/EC_{LucF}}{BC_{LucN}/BC_{LucF}}$$

Appropriate pCs for each experiment were determined by evaluating responses to each TF, selecting the promoter that did not show a significant change in expression following co-expression. The identities of pC in each experiment are as follows:

**TF**	**pC**
**AtANAC032**	**pCaMV35s**
**AtARF18**	**pAtuNos**
**AtDREB26**	**pAtuNos**
**AtNLP6**	**pCaMV35s**
**AtNLP7**	**pCaMV35s**

Supplementary Figure S5. Expression levels of Arabidopsis genes in modified TARGET assays. Values represent the mean and standard error of three biological replicates of independent protoplast transfections ($N = 3$) of which each value is the mean of two technical replicates. Network graphs represent circuit, and grey nodes are the ones under query in each graph. Forward arrows with arrowheads indicate activating interactions, while arrows with a perpendicular line at the end represent repressing interactions. P-values were calculated using an unpaired two-tailed Student's t-test, $N = 3$, *P<0.05, ** P<0.01, *** P<0.001, and **** P<0.0001. X-axis = expression relative to the control upon addition (+) of either cycloheximide (CHX) or dexamethasone (DEX) in the presence of the transcription factor glucocorticoid receptor fusion (gene:GR) or the empty vector. No addition is represented by a (-). Expression is calculated relative to the control gene eEF-1α using the $2^{-\Delta\Delta C_T}$ method. Supports Figure 3.

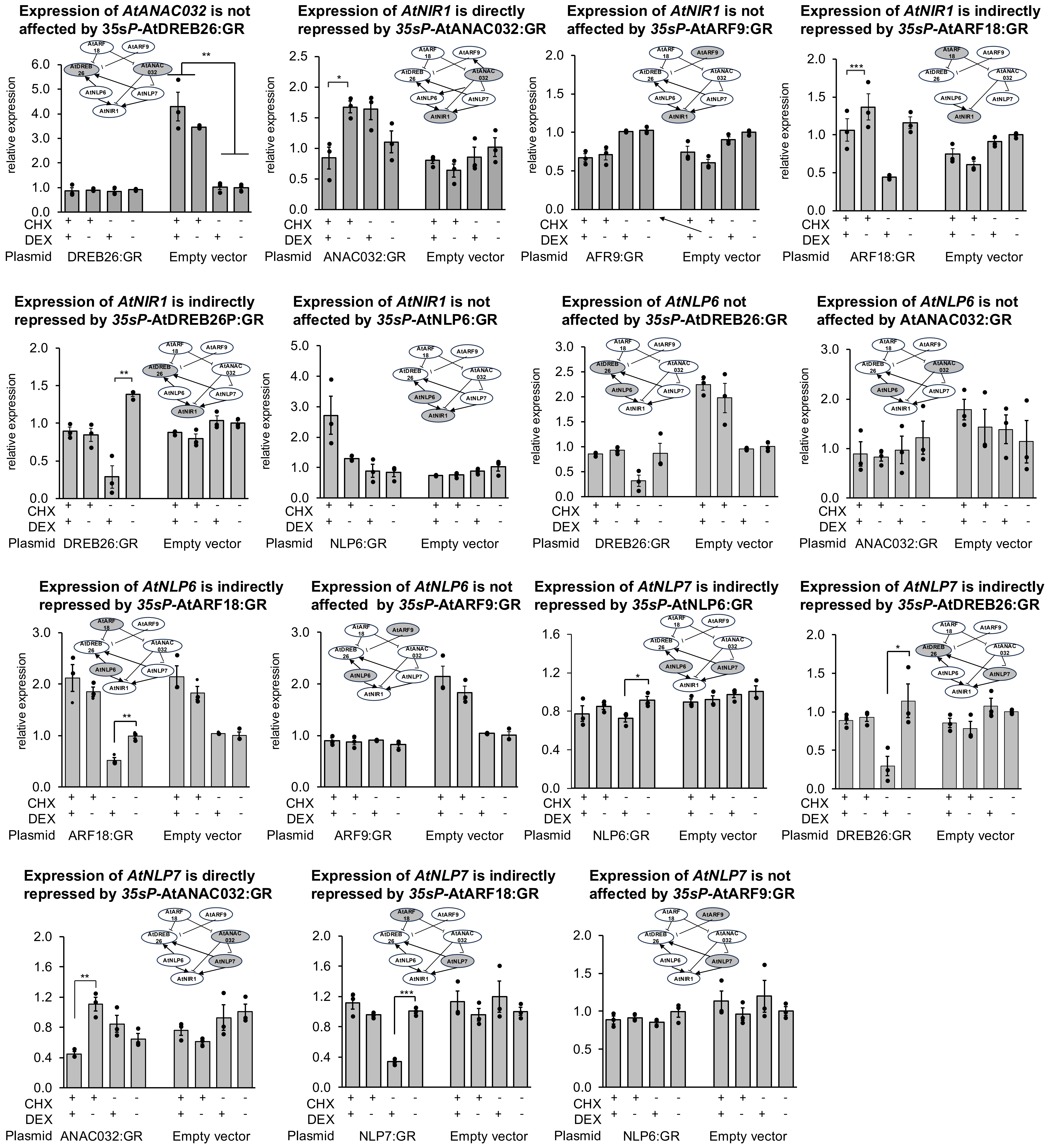

Summary of changes in gene expression of target genes in modified TARGET assays. Figures indicate fold change in expression. Upper figures indicate the effects of direct interactions (cells treated with cycloheximide). Figures below indicate indirect effects (absence of cycloheximide). Red text indicates significant repression. Blue text indicates significant activation. nt, not tested; *, expression of the target gene responded to cycloheximide.

Transcription Factor	Target Genes: *AtDREB26*	*AtNLP7*	*AtNLP6*	*AtNIR1*	*AtANAC032*
AtDREB26:GR	*nt	-0.06<br>**-2.21**	-0.12<br>**-1.60**	+0.09<br>**-2.69**	-0.08*<br>-0.14*
AtNLP7:GR	*nt	-0.17<br>-0.37	nt	**+2.07**<br>-0.41	nt
AtNLP6:GR	*nt	-0.61<br>-0.72	nt	**+0.99**<br>+0.04	nt
AtARF18:GR	*nt	-0.01<br>**-1.78**	-0.05<br>**-0.98**	-0.37<br>**-1.38**	**-3.91***<br>**-2.63***
AtARF9:GR	*nt	-0.05<br>-0.21	+0.04<br>+0.15	-0.37<br>+0.12	+0.07*<br>+0.32*
AtANAC032:GR	*nt	**-1.39**<br>-0.57	+0.02<br>-0.35	**-1.07**<br>+0.59	nt

Supplementary Figure S6. Luciferase transactivation assays. Graphs show luminescence from Arabidopsis promoter(p):nanoluciferase (LucN) constructs relative to CaMV35s:LucF and normalized CaMV35s:LucN/CaMV35s:LucF to with and without co-expression of transcription factor. Values represent the mean and standard error of three biological replicates (independent transfections). Network graphs represent the circuit being tested with the genes being tested in grey. Forward arrows with arrowhead = activating; perpendicular arrows = repressing. X-axis is representative of the protein that is co-expressed , while the y-axis refers to the normalized luminescence. *P*-values were calculated using an unpaired two-tailed Student's t-test **P*<0.05, ** *P*<0.01, *** *P*<0.001). Supports Figure 3.

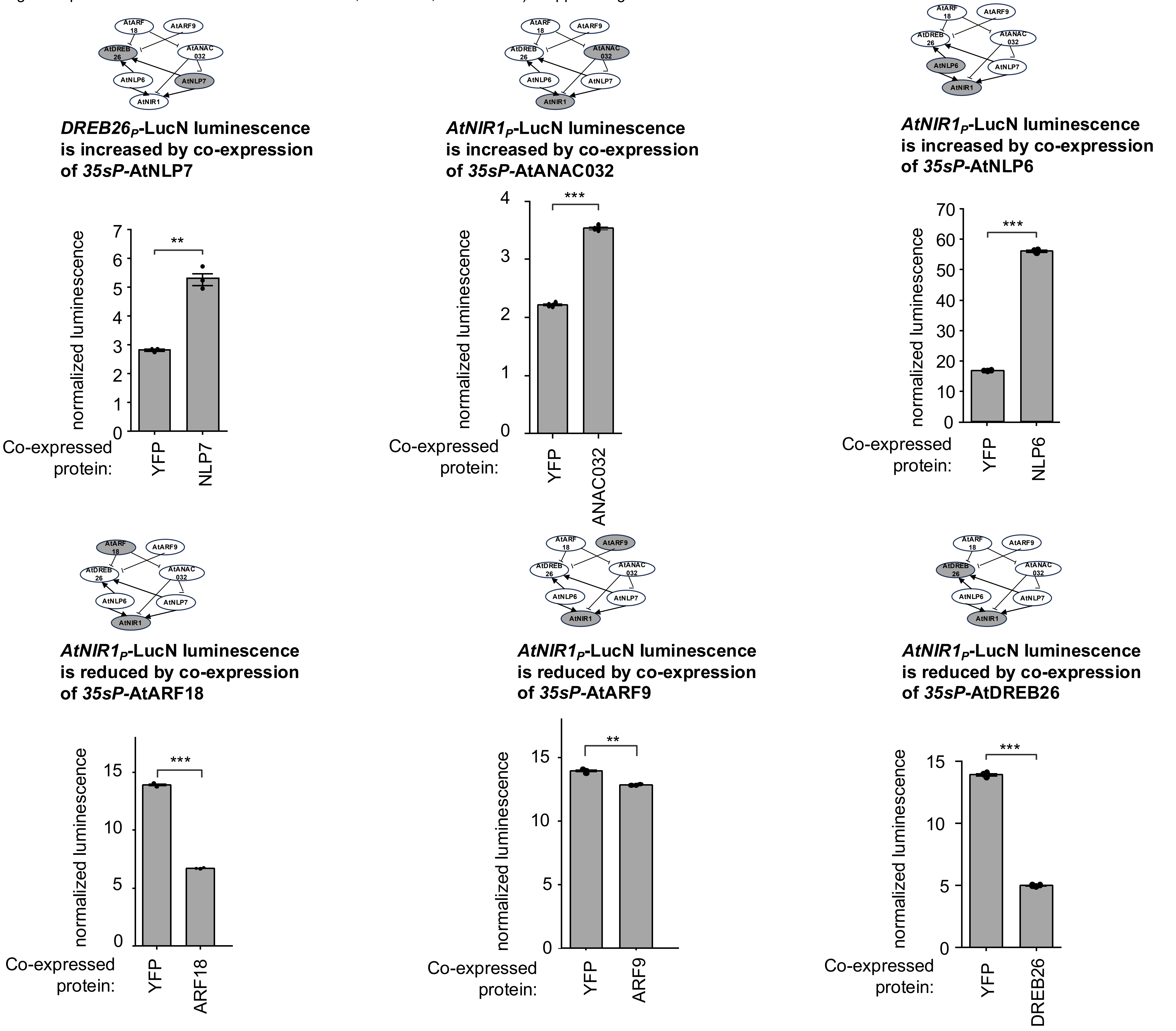

Supplementary Figure S7. Nitrate-responsive genes in tomato roots. (a) A heatmap shows scaled $\log_2$CPM (counts per million) of tomato nitrogen-responsive genes in the M82 root RNA-seq experiment. Red represents a relative increase in expression, while blue represents a relative decrease in expression. **(b-d)** GO enrichment analysis on 3 clusters of DEGs that are up-regulated at higher nitrate conditions in M82 RNA-seq experiment. Different colors represent over represented p-values. Supports Figure 4.

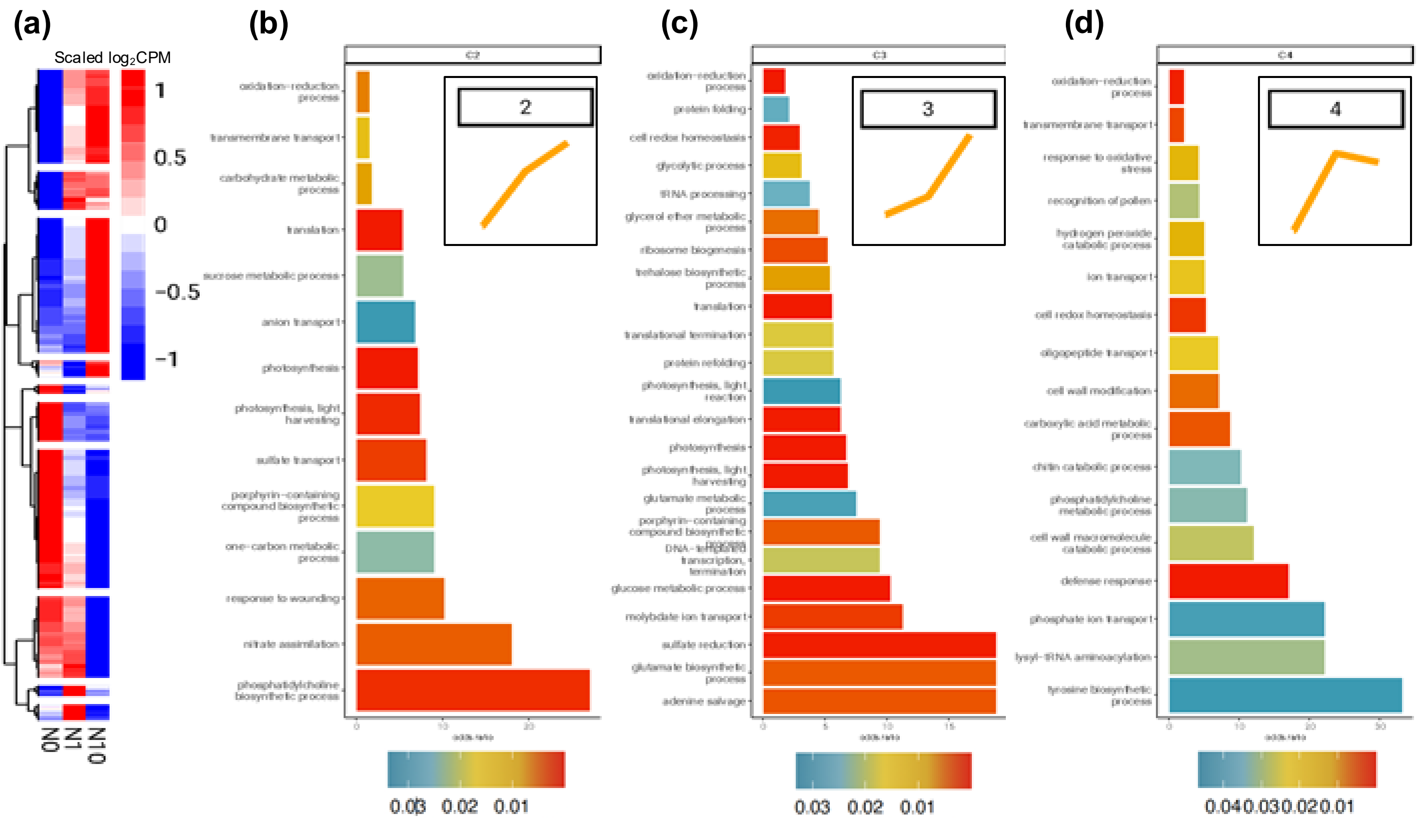

Supplementary Figure S8. Phylogenetic analysis of transcription factors. (a – g) Maximum likelihood analysis of the amino acid sequences of transcription factors. Scale bars indicate the number of substitutions per site. Numbers at nodes indicate bootstraps. Sequences from *S. lycopersicum* are highlighted in red, while those from Arabidopsis are highlighted in blue. Supports Figure 5.

(a) ARF18

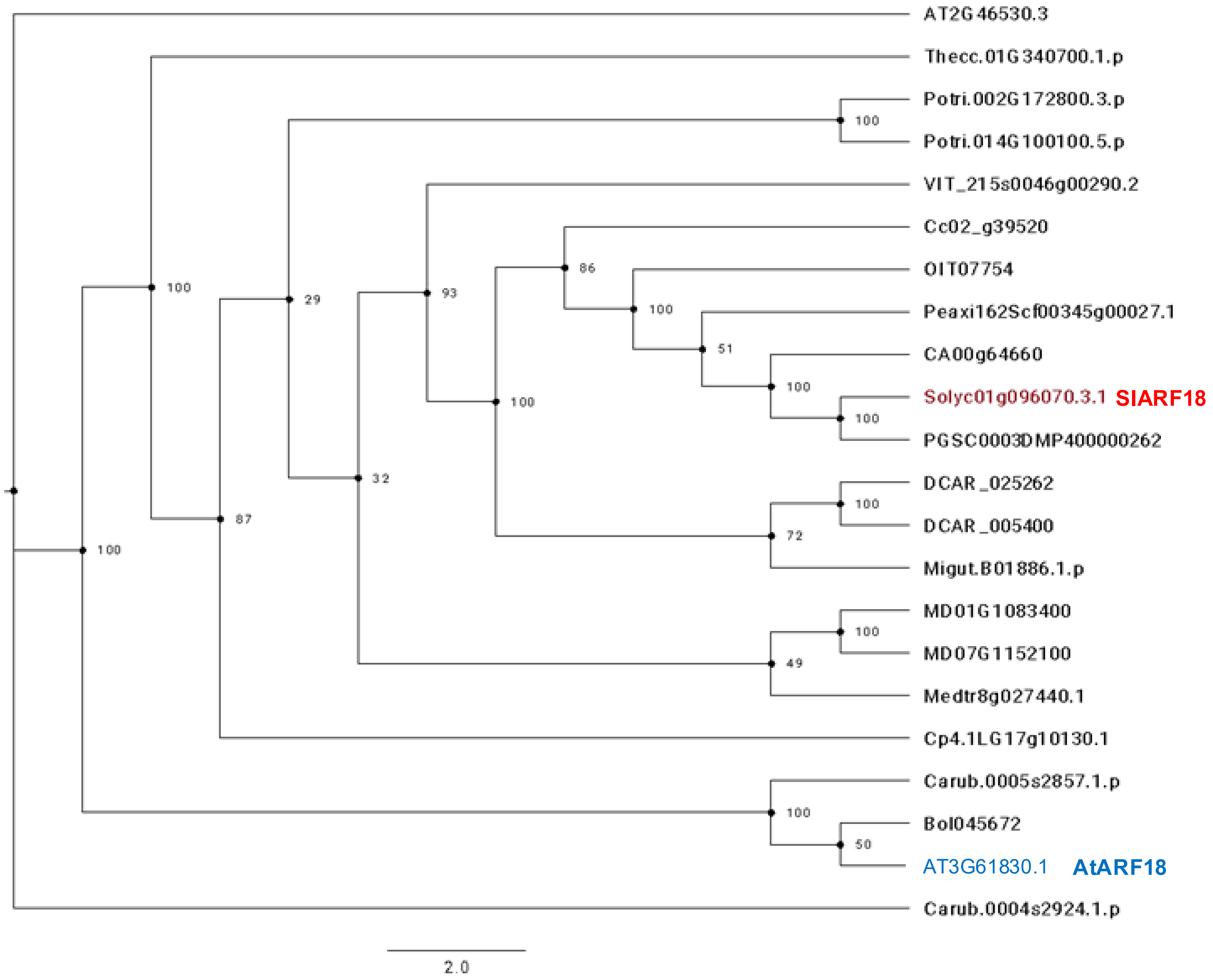

Supplementary Figure S8. Phylogenetic analysis of transcription factors in this study. (a – g) Maximum likelihood analysis of the amino acid sequences of transcription factors. Scale bars indicate the number of substitutions per site. Numbers at nodes indicate bootstraps. Sequences from *S. lycopersicum* are highlighted in red, while those from Arabidopsis are highlighted in blue. (b) The tpm expression of two tomato *ARF9* orthologs showing *SlARF9A* gene is not expressed in tomato roots, while *SlARF9B* gene shows high expression in tomato roots. The source data is from Kajala et al (23) and X axis represents different cell types. COR, cortex; EN, endodermis; EP, epidermis; EXO, exodermis; MCO, meristematic cortex; MZ, meristematic zone; PH, phloem; V, vascular initials; WOX, *SlWOX5* expressed region; XY, xylem. Supports Figure 5.

(b) ARF9

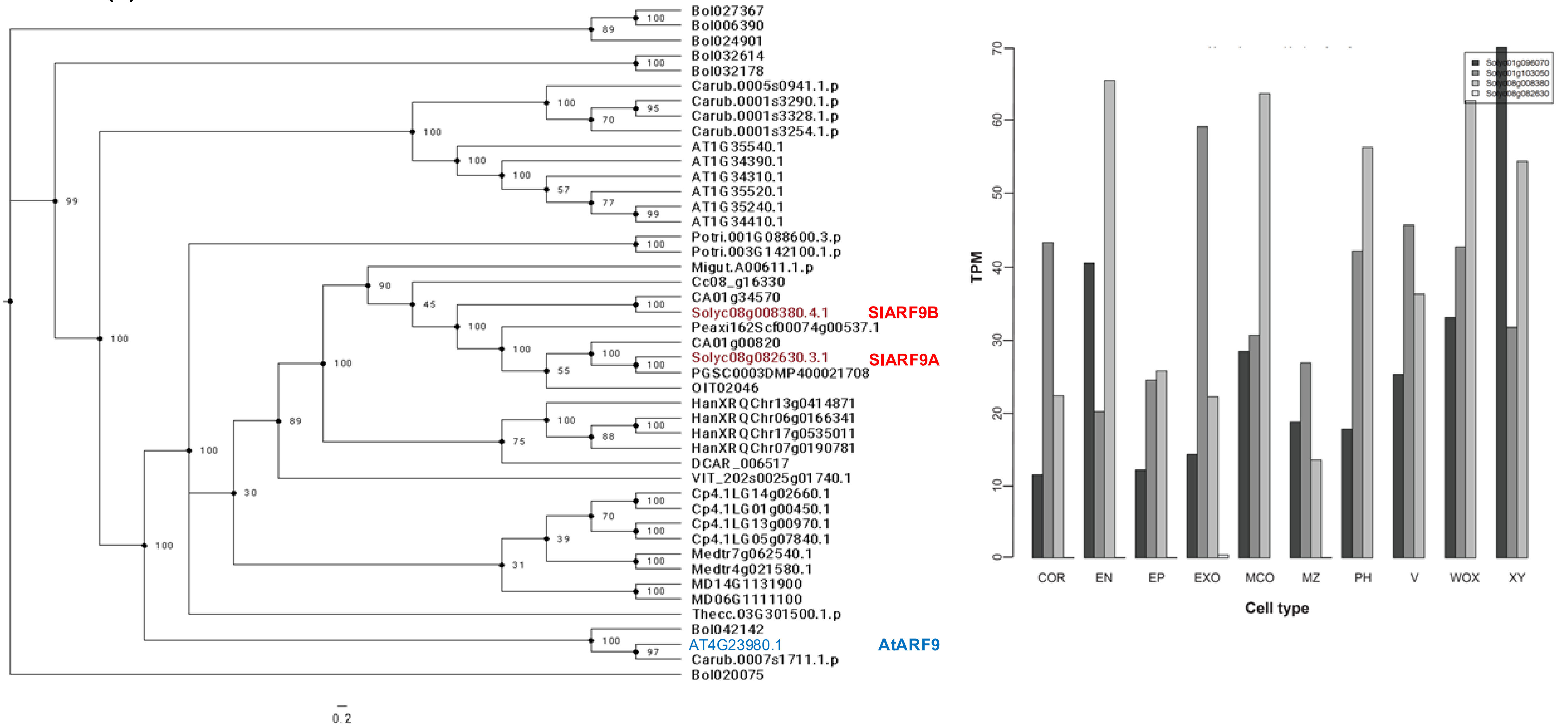

Supplementary Figure S8. Phylogenetic analysis of transcription factors in this study. (a – g) Maximum likelihood analysis of the amino acid sequences of transcription factors. Scale bars indicate the number of substitutions per site. Numbers at nodes indicate bootstraps. Sequences from *S. lycopersicum* are highlighted in red, while those from Arabidopsis are highlighted in blue. Supports Figure 5.

(c) DREB26

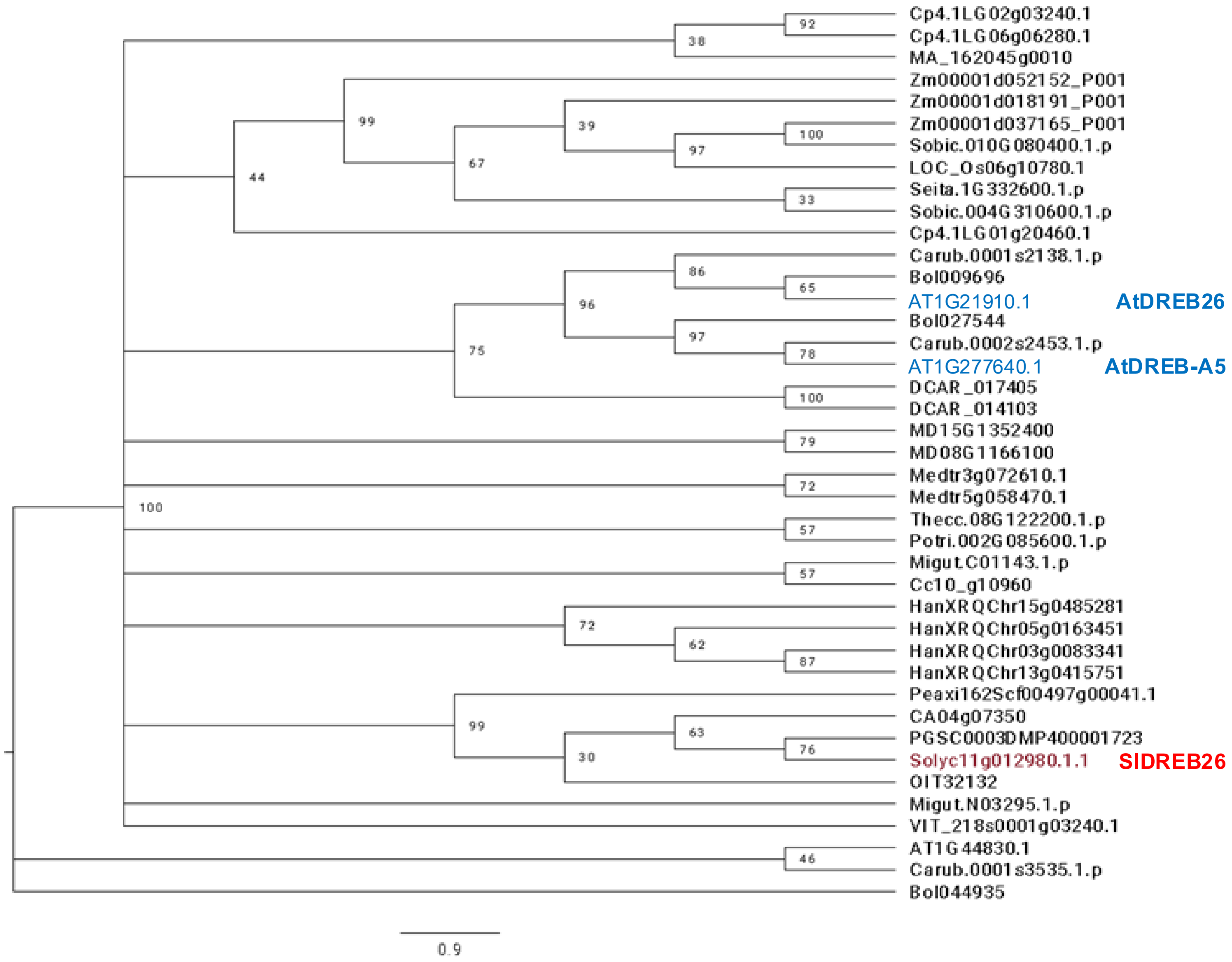

Supplementary Figure S8. Phylogenetic analysis of transcription factors in this study. (a – g) Maximum likelihood analysis of the amino acid sequences of transcription factors. Scale bars indicate the number of substitutions per site. Numbers at nodes indicate bootstraps. Sequences from *S. lycopersicum* are highlighted in red, while those from Arabidopsis are highlighted in blue. Supports Figure 5.

(d) NLP6/7

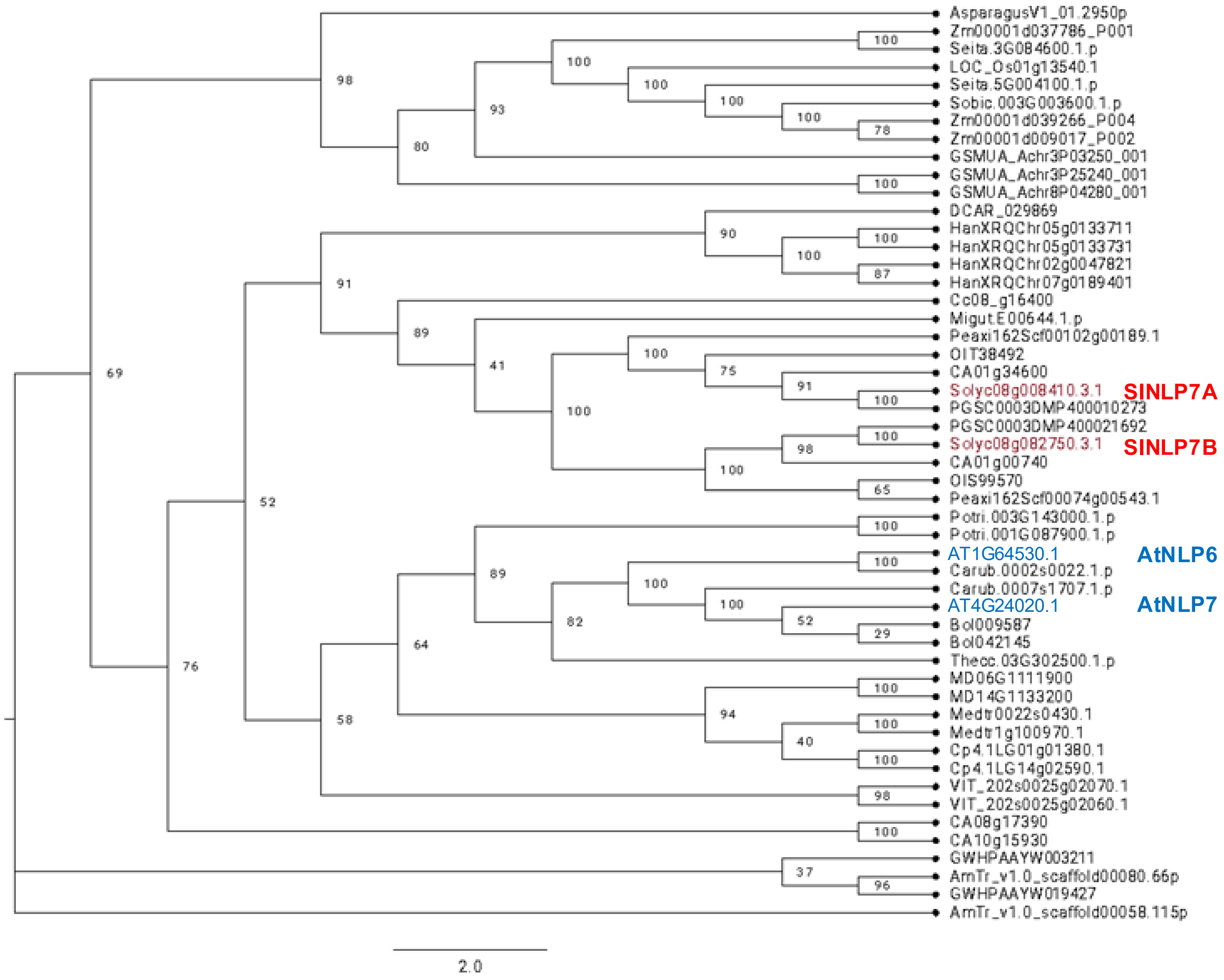

Supplementary Figure S8. Phylogenetic analysis of transcription factors in this study. (a – g) Maximum likelihood analysis of the amino acid sequences of transcription factors. Scale bars indicate the number of substitutions per site. Numbers at nodes indicate bootstraps. Seq uences from *S. lycopersicum* are highlighted in red, while those from Arabidopsis are highlighted in blue. Supports Figure 5.

(c) ANAC032

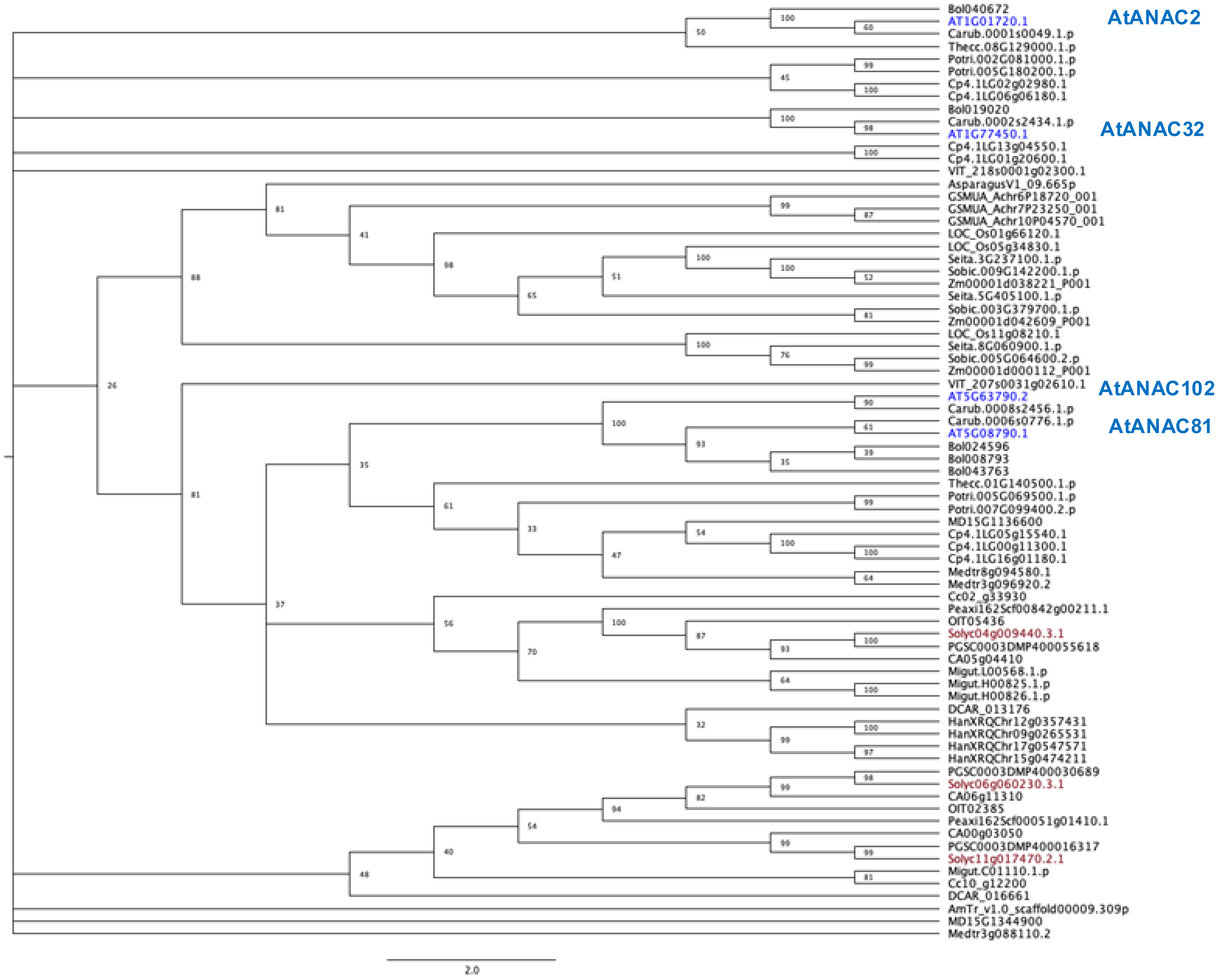

Supplementary Figure S8. Phylogenetic analysis of transcription factors in this study. (a – g) Maximum likelihood analysis of the amino acid sequences of transcription factors. Scale bars indicate the number of substitutions per site. Numbers at nodes indicate bootstraps. Sequences from *S. lycopersicum* are highlighted in red, while those from Arabidopsis are highlighted in blue. Supports Figure 5.

(f) NIR1

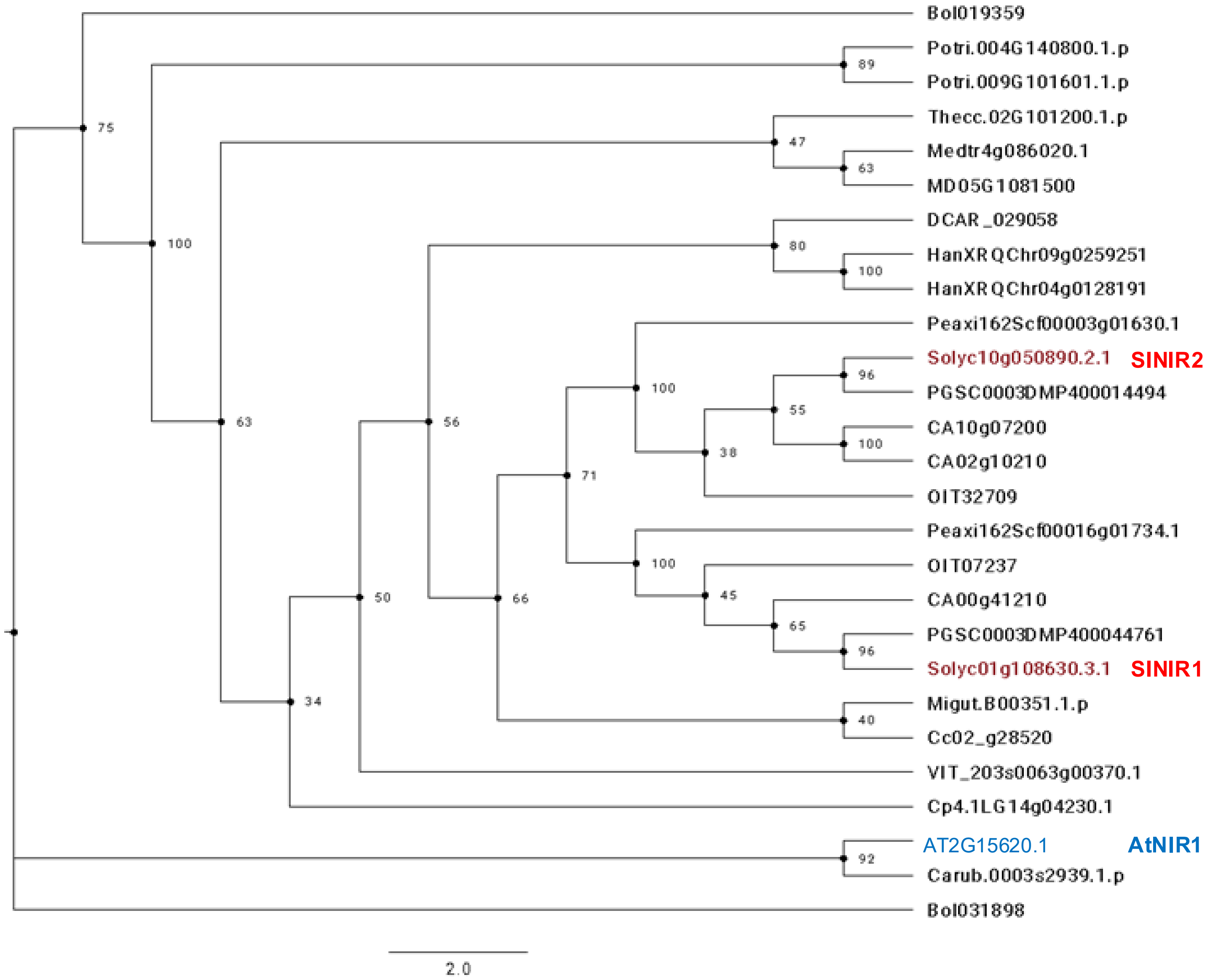

Supplementary Figure S9. Domain analysis of tomato orthologs of *AtNLP7.*
(A-B) RNAseq reads from Kajala, *et al.*, 2021 (*Cell* **184**, 3333-3348.e19) were mapped to predicted NLP7 orthologs in the tomato genome. *Solyc08g008410* appears mis-annotated and contains duplicate genes. We name these *SlNLP7A* and *SlNLP7C*, the latter of which has relatively low expression (B) RNAseq reads mapped to the annotated SlNLP7B gene. (C) Amino acid sequence alignment of SlNLP7A, SlNLP7B, and SlNLP7C showing similarity. (D) Domain analysis of SlNLP7A, SlNLP7B, and AtNLP7. All contain RWP-RK and PB1 domains. (E) Nitrate binding domains as previously reported are framed by red rectangles (Liu *et al.,* 2022. *Science* 377, 1419–1425). Supports Figure 5.

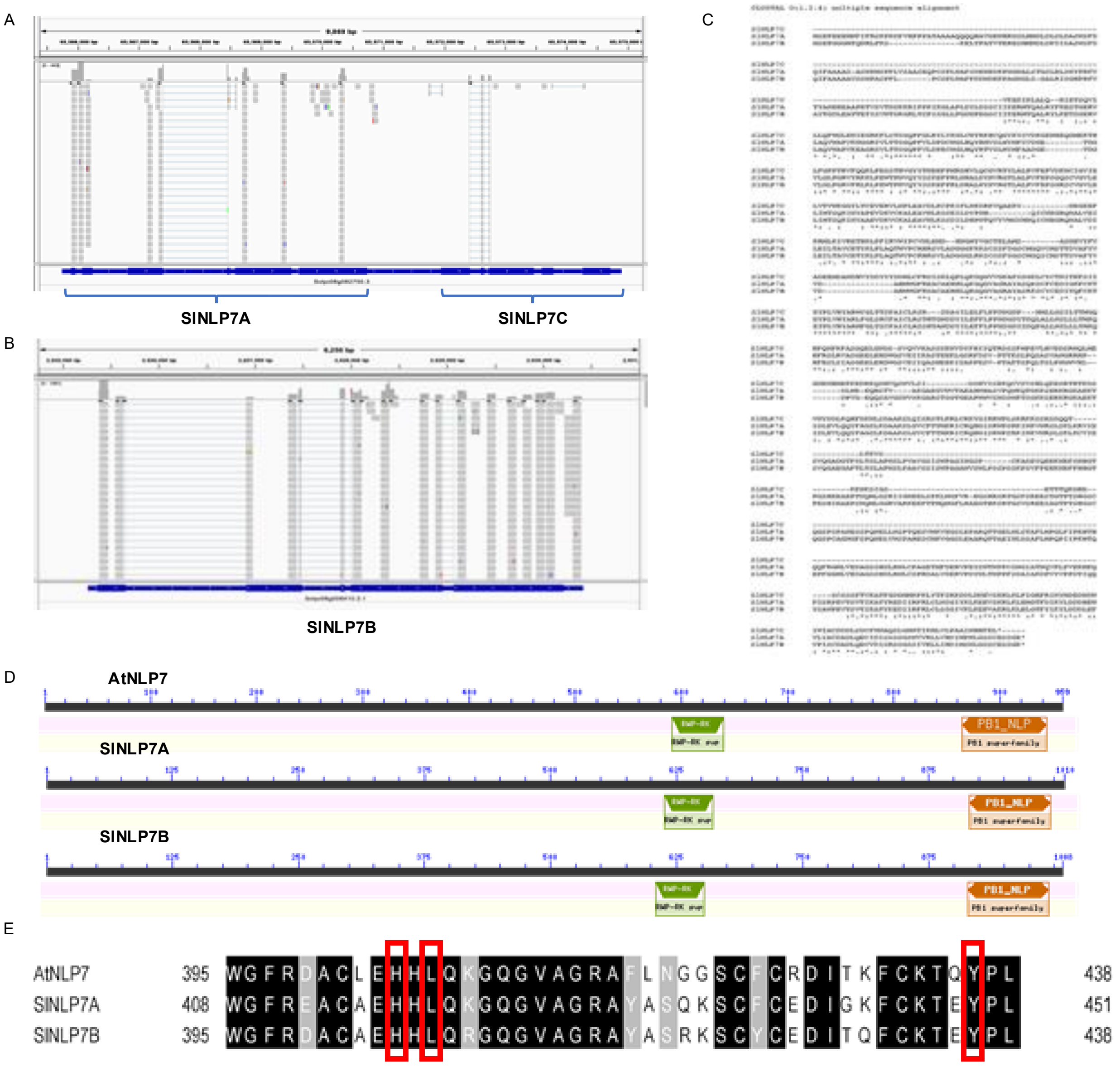

Supplementary Figure S10
Table of Arabidopsis and tomato mutant lines.
Bold text with subscript ‘c’ indicates mutation was created by CRISPR/Cas9 (details in Supplementary data 12). All other lines are T-DNA insertion lines. Supports Figures 5, 8.

Arabidopsis (At)	Tomato (Sl)
nlp6	***nlp7a***
nlp7-1	***nlp7b***
nlp6/nlp7-1	***nlp7a/nlp7b***
anac032	
anac032$_c$	
arf9b	***arf9b***
arf18-2	
arf18-3	
arf18$_c$	***arf18***
arf18-2/nlp7-1	***arf9b/arf18***
dreb26$_c$	***dreb26***
dreb26$_c$***/nlp7***$_c$	***arf9b/arf18/dreb26***
anac032/nlp7-1	***dreb26/nlp7a/nlp7b***
nlp7-1/arf18-2	***arf9b/arf18/dreb26/nlp7a/nlp7b***
arf18-2/anac032	

Supplementary Figure S11. Genotypes of Arabidopsis and tomato CRISPR lines. Supports Figures 5, 8.

Genotypes of Arabidopsis CRISPR/Cas9 lines

Plant ID	Target TF(s)	sgRNA1	sgRNA2
dreb26-cc-1<br>(OZ0033T1-10T2-05T3-14)	AtDREB26	GAAGATGGTGATGATGACAA**AGG**<br><br>**wild type**	TTAAGAAGTACAAAGGA-GTG**AGG**<br>TTAAGAAGTACAAAGGA**A**GTG**AGG**<br>**homozygous**
Anac032-cc-1<br>(OZ0033T1-19T2-29)	AtANAC032	ATTTACGACAGAGATACATG**AGG**<br><br>**wild type**	AATATCCAGTACCAGCTGCA**CGG**<br>AAT--------------GCA**CGG**<br>**homozygous**
Arf18-cc-1<br>(OZ0033T1-19T2-02T3-21)	AtARF18	GTTGAAGGTGATGATGATTT**CGG**<br><br>**wild type**	AGAGTTTTCTACTTCCC-TCA**GGG**<br>AGAGTTTTCTACTTCCC**T**TCA**GGG**<br>**homozygous**
dreb26-nlp7-cc-1<br>(OZ0103T1-47T2-35T3-06)	AtDREB26<br><br><br><br>AtNLP7	CGTGTAAGAACAAGAACAAG**AAGAGT**<br>CGTGTAAGAACAAGAAC---------<br>**homozygous**<br><br>GTAGAGCTCGGACTCCCGGG**TCGAGT**<br>GTAGAGCTCGGACTCCC---------<br>**homozygous**	GATTAATTGAAACTCCAAAG**CGGAAT**<br>GATTAATTGAAACTCCA---------<br>**homozygous**<br><br>GTCTGAGCGAGAGGCAAGTT**ATGGGT**<br>GTCTGAGCGAGAGGCAA---------<br>**homozygous**

Supplementary Figure S11. Genotypes of Arabidopsis and tomato CRISPR lines.

Sequencing chromatograms of homozygous CRISPR/Cas9 knockout lines. Exons are schematically displayed as blue boxes and introns and untranslated regions as grey boxes. The sgRNA target sites are represented with pink arrowheads. The DNA sequence of sgRNAs are highlighted in grey and PAMs are shown in bold case. CRISPR/Cas9-induced mutations are shown below the chromatogram in red bold case letters or dashes. HMZ - homozygous.

DREB26-cc-1[HMZ]
OZ0033T1-10T2-05T3-14

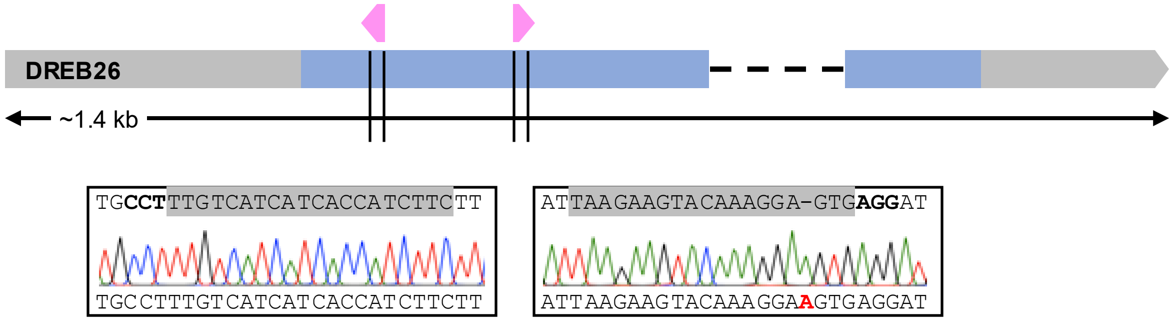

ANAC032-cc-1[HMZ]
OZ0033T1-19T2-29

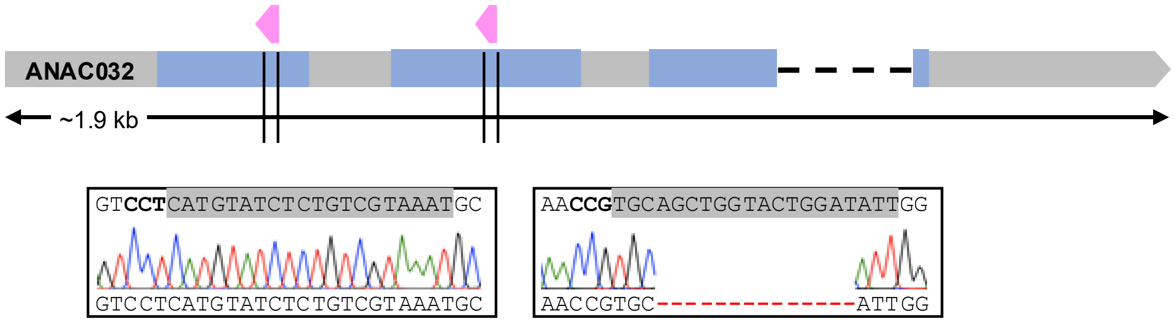

ARF18-cc-1
OZ0033T1-19T2-02T3-21

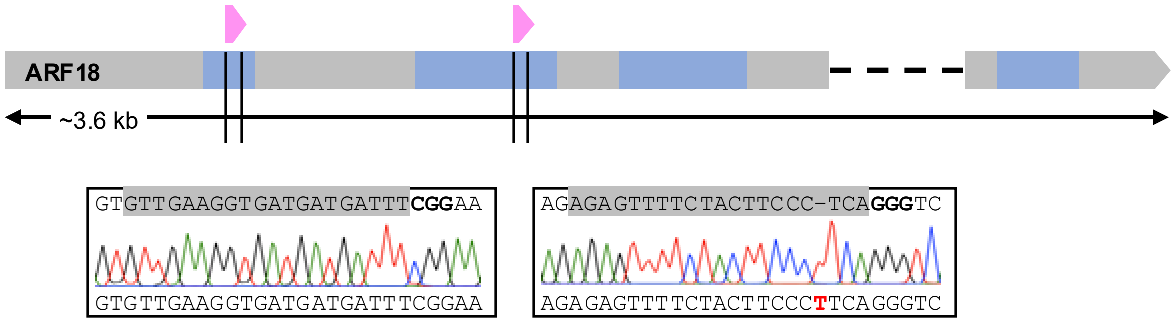

Supplementary Figure S11. Genotypes of Arabidopsis and tomato CRISPR lines.

Sequencing chromatograms of homozygous CRISPR/Cas9 knockout lines. Exons are schematically displayed as blue boxes and introns and untranslated regions as grey boxes. The sgRNA target sites are represented with pink arrowheads. The DNA sequence of sgRNAs are highlighted in grey and PAMs are shown in bold case. CRISPR/Cas9-induced mutations are shown below the chromatogram in red bold case letters or dashes.

DREB26[HMZ] NLP7[HMZ]
OZ0103T1-47T2-35T3-06

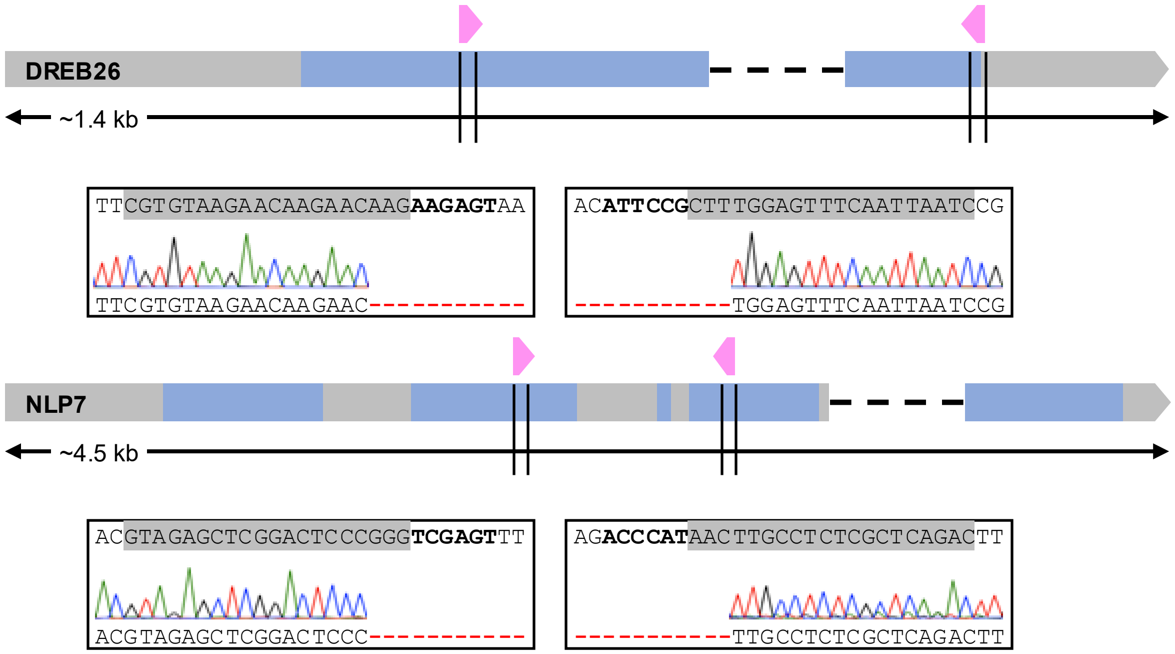

Genotypes of Tomato CRISPR/Cas9 hairy root lines

Red font = mutation type

Plant ID	Target TF	sgRNA1	sgRNA2
slarf9b #8	SlARF9B	GAGTTATGGAGATTGTGTTGC**AGG** **+1bp**<br>**homozygous**	AGCGTAATTTAGGAGGGTTTG**TGG** **+1bp**<br>**homozygous**
slarf18 #2	SlARF18	GATCTCTGGAAGGCA----TGC**AGG** **-2bp**<br>**homozygous**	GATCACATTGCACCCT--AGG**CGG** **-1bp**<br>**homozygous**
slarf9b/18 #4	SlARF9B<br><br><br><br>SlARF18	GAGTTATGGAGATTGTG----------- **-13bp**<br>GAGTTATGGAGAT----TGTGC**AGG** **-2bp**<br>GAGTTATGGAGATTGTTGTGC**AGG** **+1bp**<br>**mosaic**<br><br>GATCTCTGGAA------------TGC**AGG** **-6bp**<br>GATCTCTGGAAGGC-----TGC**AGG** **-3bp**<br>**biallelic**	AGCGTAATTTAGGAGGGTTTG**TGG** **+1bp**<br>AGCGTAATTTAGGAGGGTTTTG**TGG** **+2bp**<br>**biallelic**<br><br>GATCACATTGCACCCTGTAGG**CGG** **+1bp**<br>GATCACATTGCACCCT----AGG**CGG** **-1bp**<br>**biallelic**
sldreb26 #10	SlDREB26	GAAGAACAAG--------------------------- **-71bp**<br>**homozygous**	**CCT**----AAGGCCCCTCTGCTTCCT **-2bp**<br>**homozygous**
slnlp7a #1	SlNLP7A	GGACTTTGTACATCCA--CGC**CGG** **-1bp**<br>GGACTTTGTACATCC----------------- **-174bp**<br>**biallelic**	TATGTCGCCGTTTC----------T**CGG** **-5bp**<br>---------------------------------GTAT**CGG** **-174bp**<br>**biallelic**
slnlp7b #13	SlNLP7B	AAAGGAGTTGACTCCGGCGA**CGG** **WT**<br>**wild type**	**CCT**CT-----------------TTTCCCGATGA **-7bp**<br>**CCT**CTTTTGGGCATTTCCCGATGA **+1bp**<br>**biallelic**
slnlp7a/b #21	SlNLP7A<br><br><br>SlNLP7B	GGACTTTGTACATCCACCGC**CGG** **WT**<br>**wild type**<br><br>AAAGGAGTTGACTCCGGCGA**CGG** **WT**<br>AAAGGAGTTGACTCCGGTCGA**CGG** **+1bp**<br>**heterozygous**	TATGTCGCCGTTTCTCGATAT**CGG** **+1bp**<br>**homozygous**<br><br>**CCT**C----------------TTTCCCGATGA **-8bp**<br>**homozygous**
slarf9b/18/dreb26 #9	SlARF9B<br><br>SlARF18<br><br>SlDREB26	GAGTTATGGAGAT----TGTGC**AGG** **-2bp**<br>**homozygous**<br><br>GATCTCTGGAAGGCATG--GC**AGG** **-1bp**<br>**homozygous**<br><br>CAAAGGAGT----------------------------- **-130bp**<br>GAATGAGAAGCTGGGGA--CA**TGG** **-1bp**<br>**biallelic**	AGCGTAATTTAGGA------------------ **-11bp**<br>**homozygous**<br><br>---------------------------------AGG**CGG** **-20bp**<br>**homozygous**<br><br>------------AGGCCCCTCTGCTTCCT **-130bp**<br>**CCT**A---------------CCTCTGCTTCCT **-7bp**<br>**biallelic**
sldreb26/nlp7a/b #6	SlDREB26<br><br><br>SlNLP7A<br><br><br>SlNLP7B	GAATGAGAAGC-------------TCA**TGG** **-6bp**<br>GAATGAGAAGCT-----------TCA**TGG** **-5bp**<br>**biallelic**<br><br>GGACTTTGTACATCCACCGC**CGG** **WT**<br>GGACTTTGTA---------------CGC**CGG** **-7bp**<br>**heterozygous**<br><br>AAAGGAGTTGACTCCGGCGA**CGG** **WT**<br>AAAGGAGTTGACT--CGGCGA**CGG** **-1bp**<br>**heterozygous**	**CCT**----AAGG--CCCTCTGCTTCCT **-3bp**<br>**CCT**AAAAGGGCCCCTCTGCTTCCT **+1bp**<br>**biallelic**<br><br>TATGTCGCCGTTTCTCGTTAT**CGG** **+1bp**<br>**homozygous**<br><br>**CCT**C------------------TTTCCCGATGA **-8bp**<br>**CCT**CT----------------TTTCCCGATGA **-7bp**<br>**biallelic**
slarf9b/18/dreb26/nlp7a/b #2	SlARF9B<br><br>SlARF18<br><br>SlDREB26<br><br>SlNLP7A<br><br>SlNLP7B	GAGTTATGGAGATTGTG------**AGG** **-3bp**<br>GAGTTATGGAGATTGTGTGCT**AGG** **+1bp**<br>**biallelic**<br>GATCTCTGGAAGGCAT--TGC**AGG** **-1bp**<br>**homozygous**<br>GAAGAACAAGTAC--------------------- **-101bp**<br>**homozygous**<br>GGACTTTGTACATCCA--CGC**CGG** **-1bp**<br>**homozygous**<br>AAAGGAGTTGACTCCGGCGA**CGG** **WT**<br>**wild type**	AGCGTAATTTAGGAGGG----G**TGG** **-2bp**<br>AGCGTAATTTAGGAGGGTTTG**TGG** **+1bp**<br>**biallelic**<br>GATCACATTGCACCCT--AGG**CGG** **-1bp**<br>**homozygous**<br>**CCT**AAAAGGCCCCTCTGCTTCCT **WT**<br>**wild type**<br>------------------------TGCTTCTGAACAG **-111bp**<br>**homozygous**<br>**CCT**CTTTTGGGCATTTCCCGATGA **+1bp**<br>**homozygous**

Genotypes of Tomato CRISPR/Cas9 stable lines

Red font = mutation type

Plant ID	Target TF	sgRNA1	sgRNA2
slarf9b/18 #4-17	SlARF9B	GAGTTATGGAGATTGTGT----------- **-140bp**<br>**homozygous**	--TG**TGG** **-140bp**<br>**homozygous**
	SlARF18	GATCTCTGGAAGGCATGCAGGGT<br>**399bp inversion**<br>**homozygous**	CAACTAAAGGCCCTGCAAGG**CGG**<br>**399bp inversion**<br>**homozygous**
slnlp7b #8-3	SlNLP7B	AAAGGAGTTGACTCCGGCGA**CGG WT**<br>**wild type**	**CCT**CTT--GGGCATTTCCCGATGA **-1bp**<br>**homozygous**

gRNAs used to create Tomato CRISPR/Cas9 lines

Gene	gRNA1	gRNA2
**SlDREB26**	GAATGAGAAGCTGGGATCA	GAGGAAGCAGAGGGCCTTTT
**SlNLP7B**	AAAGGAGTTGACTCCGGCGA	TCATCGGGAAATGCCCAAAG
**SlNLP7A**	TATGTCGCCGTTTCTCGTAT	GGACTTTGTACATCCACCGC
**SlARF18**	GATCTCTGGAAGGCATGTGC	GATCACATTGCACCCTGAGG
**SlARF9B**	GAGTTATGGAGATTGTGTGC	GAGCGTAATTTAGGAGGGTTG

Data supporting tomato CRISPR/Cas9 knockout lines. Red arrowhead = location of guide RNA; purple magnified line = Insertion; red rectangles = substitutions.

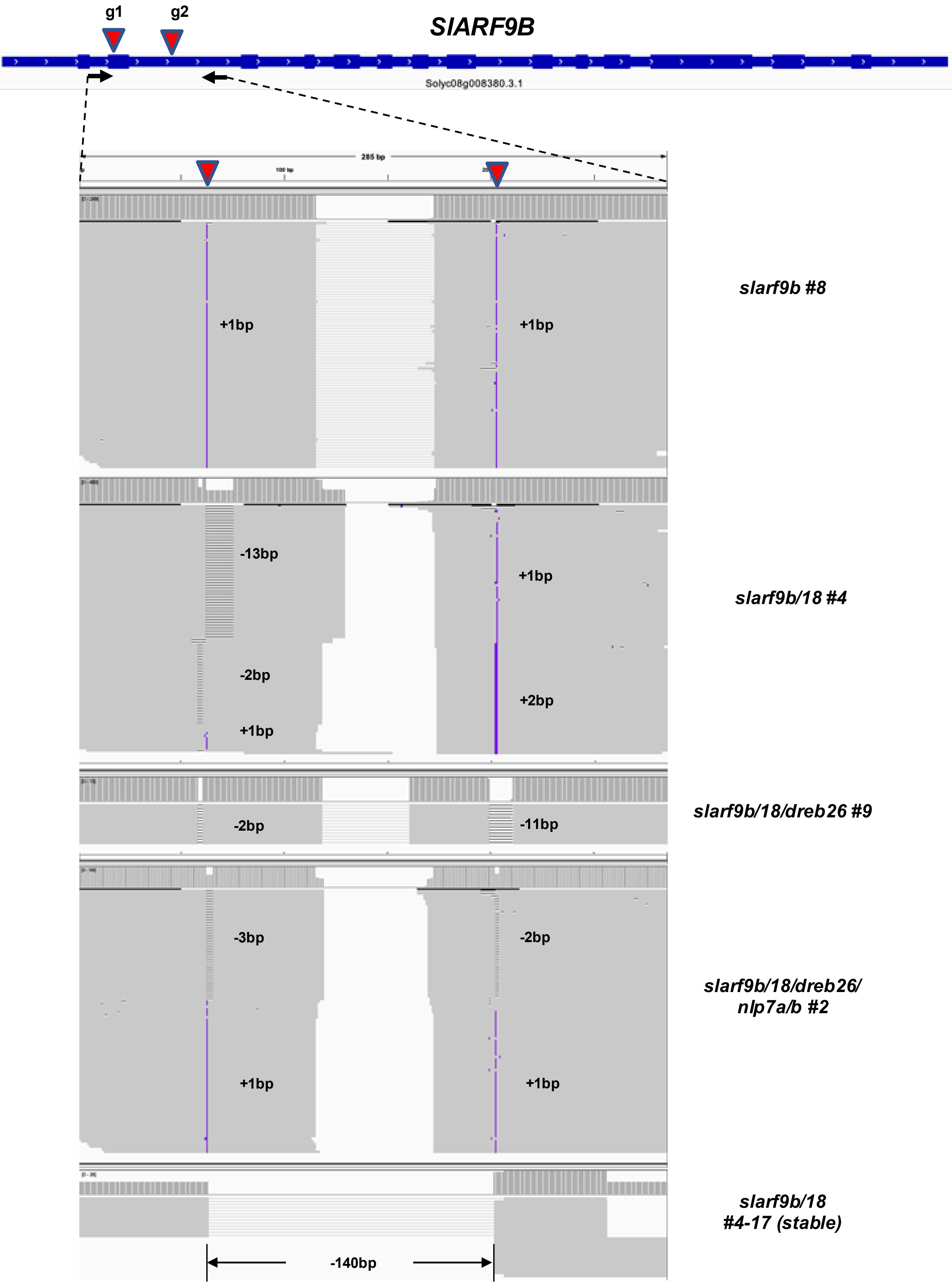

Data supporting tomato CRISPR/Cas9 knockout lines. Red arrowhead = location of guide RNA; purple magnified line = Insertion; red rectangles = substitutions.

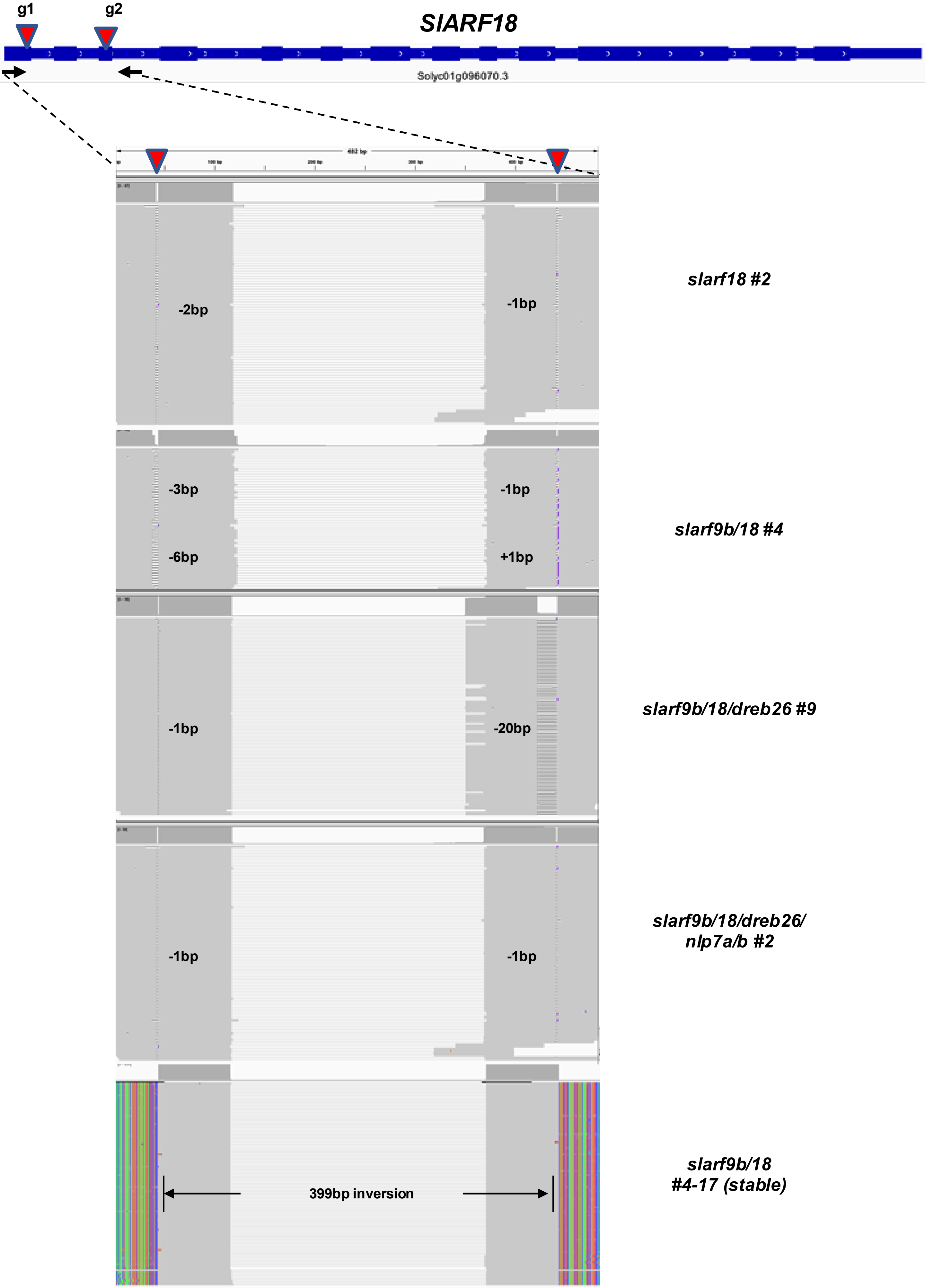

Data supporting tomato CRISPR/Cas9 knockout lines. Red arrowhead = location of guide RNA; purple magnified line = Insertion; red rectangles = substitutions.

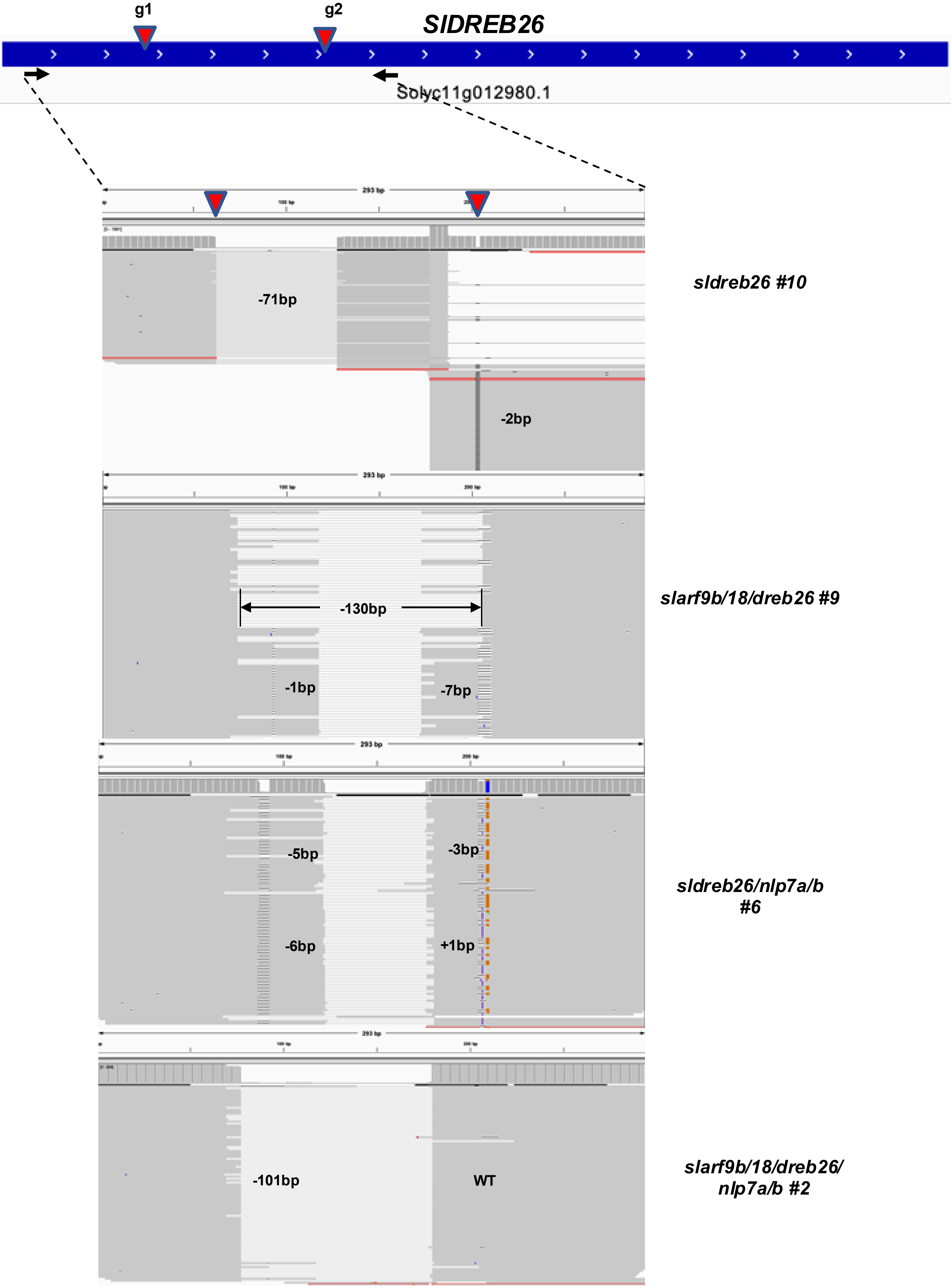

Data supporting tomato CRISPR/Cas9 knockout lines. Red arrowhead = location of guide RNA; purple magnified line = Insertion; red rectangles = substitutions.

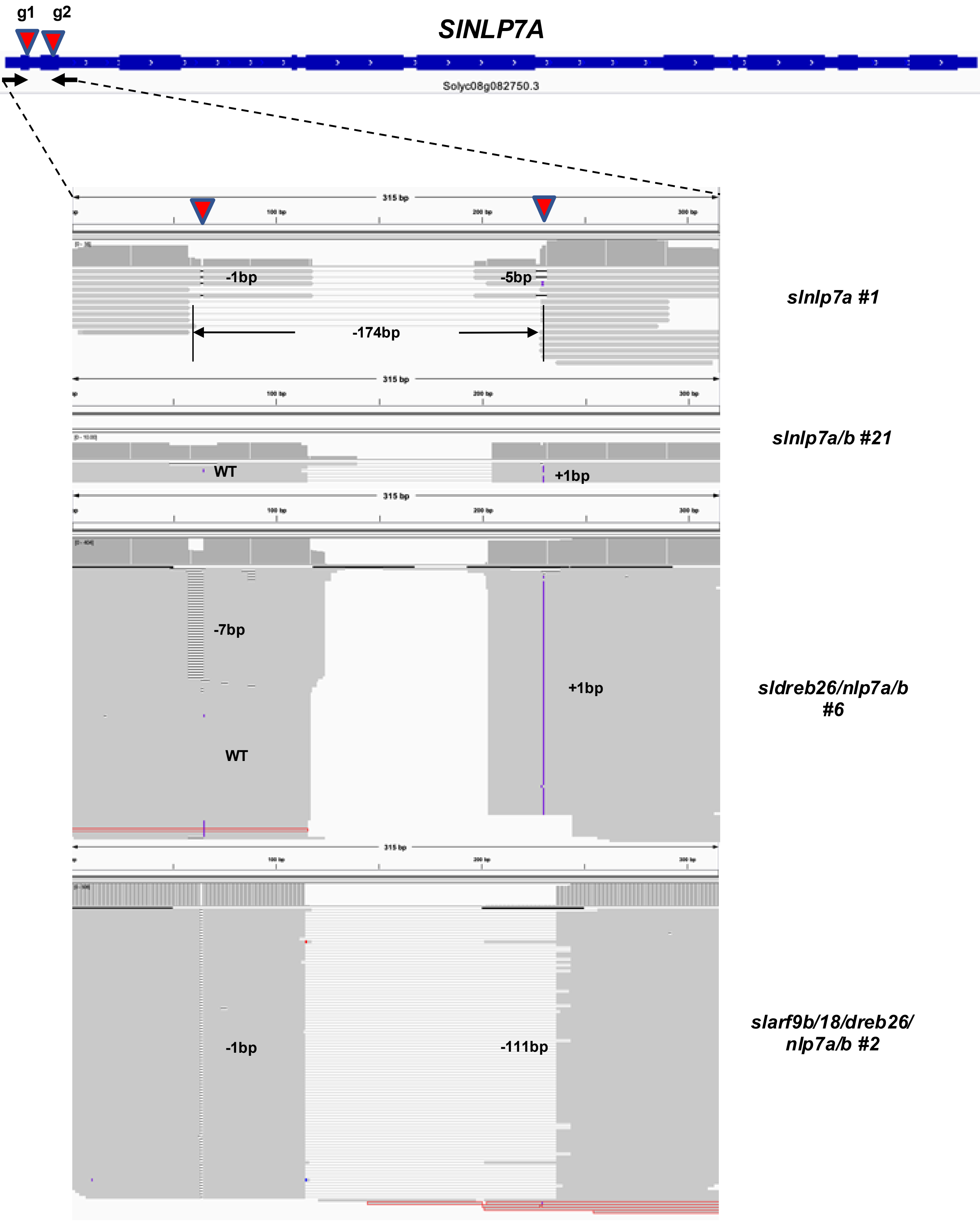

Data supporting tomato CRISPR/Cas9 knockout lines. Red arrowhead = location of guide RNA; purple magnified line = Insertion; red rectangles = substitutions.

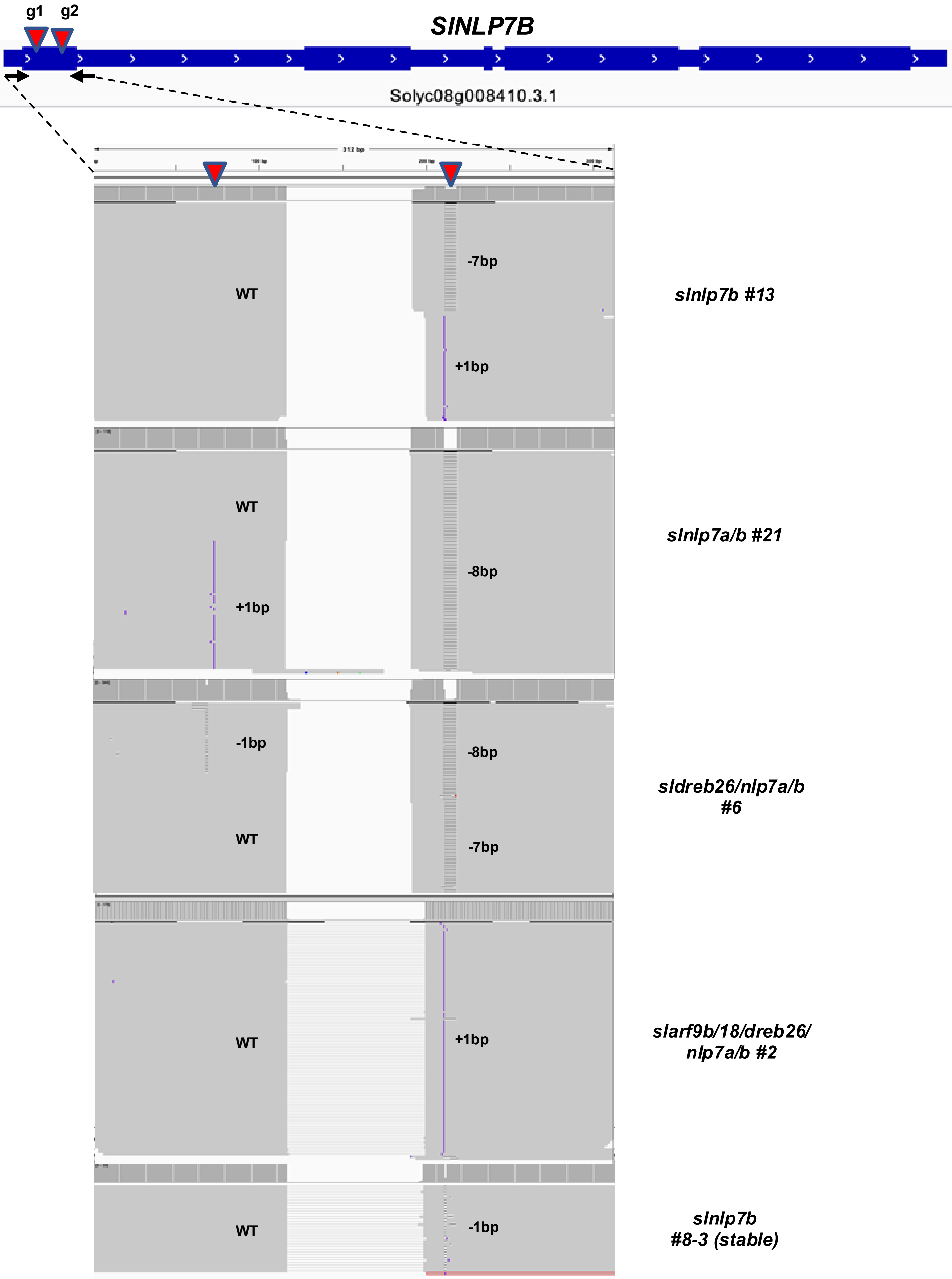

Supplementary Figure S12. Analysis of root system architecture of Arabidopsis and tomato mutant alleles of *NLP7, ANAC032 and ARF18/9*. (a) RSA phenotypes of all Traits measured include the natural logarithm-transformed primary root length (logPR), number of lateral roots (logLR), total lateral root length (logLRL), average lateral root length (logALRL), total root length (logTRL), lateral root density (logLRD) and the ratio of lateral root length to total root length (logLRP). Traits were measured in 0, 1 and 10 mM KNO_3 for tomato, and 1 and 10 mM KNO_3 for Arabidopsis. Light green represents the trait as significantly smaller relative to wild type (wt), dark green represents the trait as significantly larger relative to wild type, white indicates no significant difference and grey indicates that this phenotype was not tested at this concentration (t-test, p<.05). A black bar indicates a significant genotype x treatment interaction as measured by a two-way ANOVA (p<.05). Supports Figure 5.

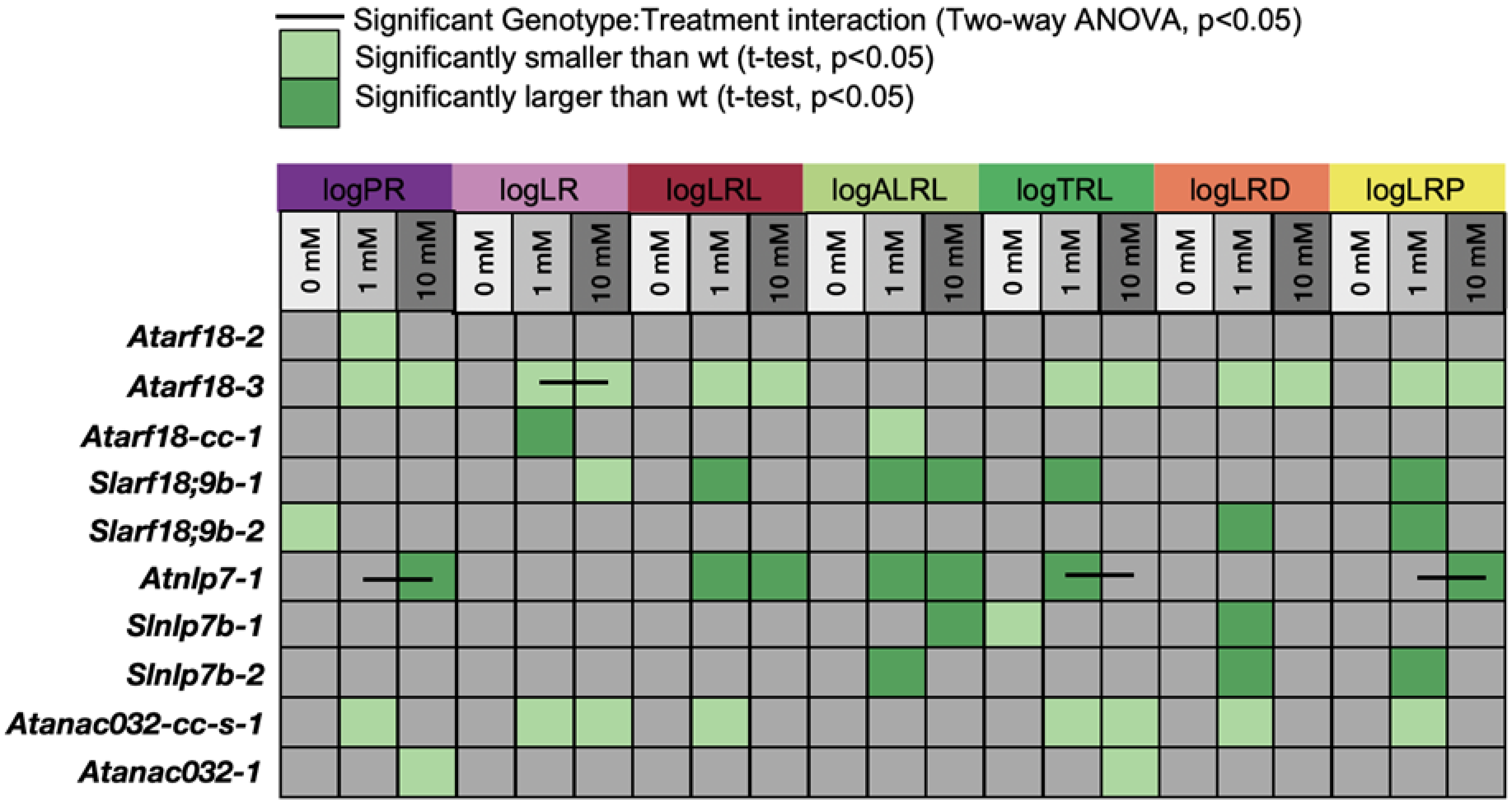

(b) Root system architecture traits of Arabidopsis alleles of *NLP7* and *ARF18*. Traits measured include the natural logarithm-transformed primary root length (logPR), number of lateral roots (logLR), total lateral root length (logLRL), average lateral root length (logALRL), total root length (logTRL), lateral root density (logLRD) and the ratio of lateral root length to total root length (logLRP). Boxplots display the distribution of the trait dataset using five key summary statistics. The lower and upper edges of the box represent the first (Q1) and third (Q3) quartiles, respectively, while the line inside the box indicates the median (Q2). The whiskers extend to the smallest and largest data points within 1.5 times the interquartile range (IQR) from the quartiles. Data points outside this range are shown individually as outliers. The minimum and maximum values, excluding outliers, mark the ends of the whiskers. Traits were measured in 1 and 10 mM KNO_3. ***=p<.001, **=p<.01, *=p<.1 as determined using a t-test. Col-0: n=536; *arf18-2:* n=43; *arf18-3:* n=34; *arf18-cc-1*: n=30. Supports Figure 5.

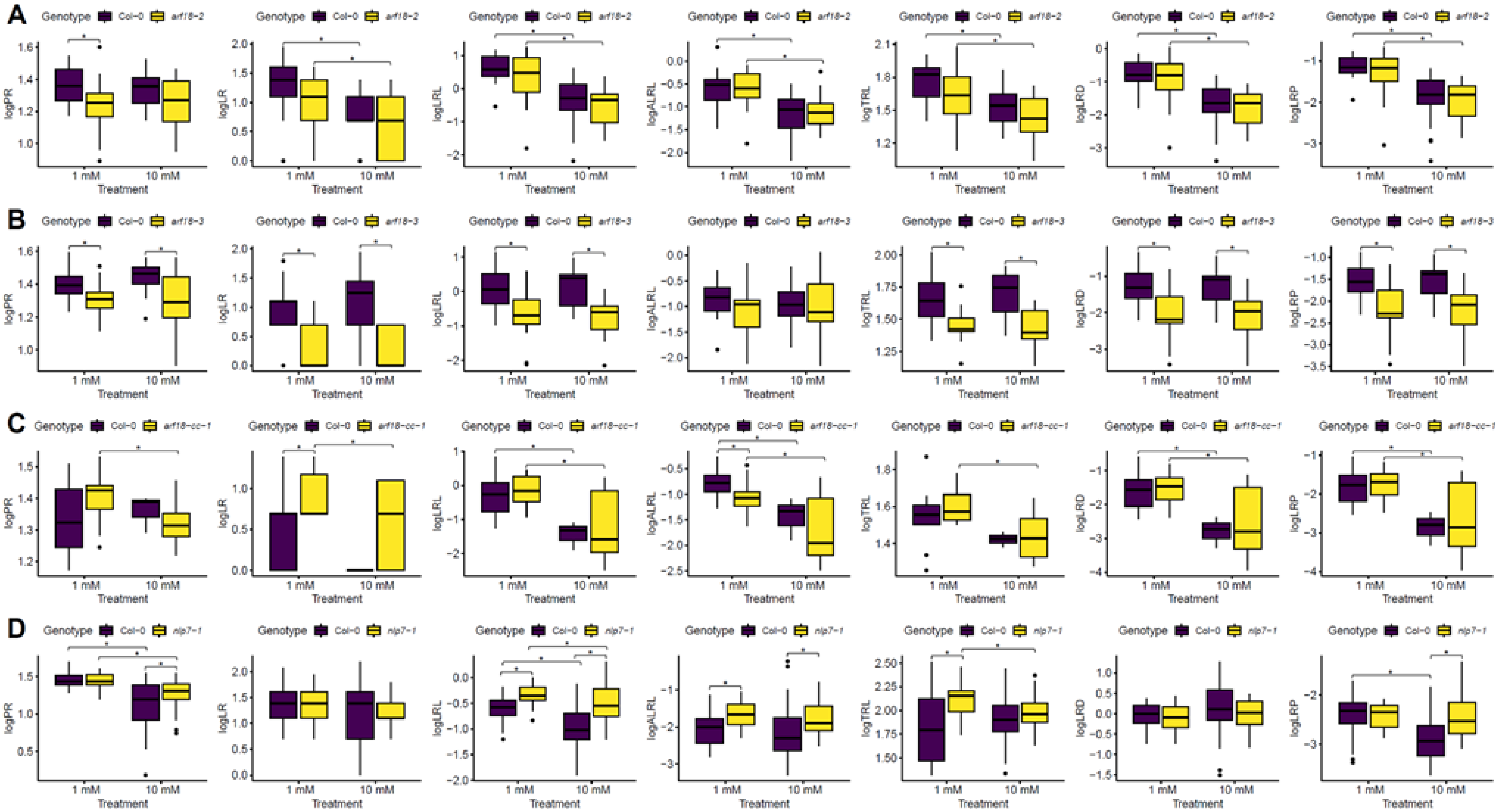

(c) Arabidopsis *ANAC032* Mutant alleles display defects in root system architecture relative to wild type. Traits measured include the natural logarithm-transformed primary root length (logPR), number of lateral roots (logLR), total lateral root length (logLRL), average lateral root length (logALRL), total root length (logTRL), lateral root density (logLRD) and the ratio of lateral root length to total root length (logLRP). Boxplots display the distribution of the trait dataset using five key summary statistics. The lower and upper edges of the box represent the first (Q1) and third (Q3) quartiles, respectively, while the line inside the box indicates the median (Q2). The whiskers extend to the smallest and largest data points within 1.5 times the interquartile range (IQR) from the quartiles. Data points outside this range are shown individually as outliers. The minimum and maximum values, excluding outliers, mark the ends of the whiskers.Traits were measured in 1 and 10 mM KNO_3. ***=p<.001, **=p<.01, *=p<.1 as supported by a t-test. Col-0: n=536; *anac032*-1: n=43; *anac032-cc-1*: n=33; *anac032-cc-s-1*: n=44. Supports Figure 5.

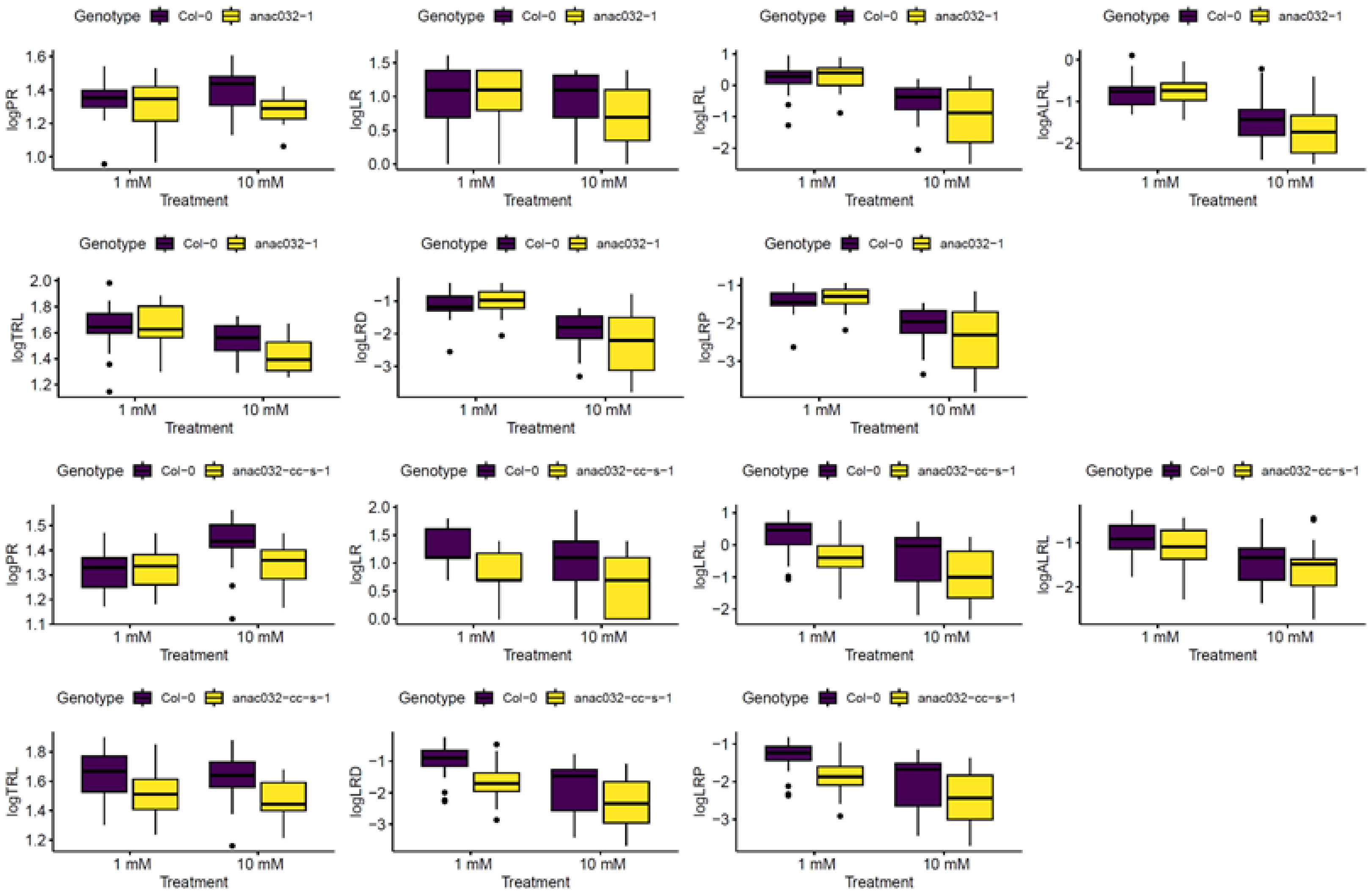

(d) Analysis of root system architecture of tomato mutant alleles of *NLP7* and *ARF18*. Traits measured include the natural logarithm-transformed primary root length (logPR), number of lateral roots (logLR), total lateral root length (logLRL), average lateral root length (logALRL), total root length (logTRL), lateral root density (logLRD) and the ratio of lateral root length to total root length (logLRP). Boxplots display the distribution of the trait dataset using five key summary statistics. The lower and upper edges of the box represent the first (Q1) and third (Q3) quartiles, respectively, while the line inside the box indicates the median (Q2). The whiskers extend to the smallest and largest data points within 1.5 times the interquartile range (IQR) from the quartiles. Data points outside this range are shown individually as outliers. The minimum and maximum values, excluding outliers, mark the ends of the whiskers.Traits were measured in 0, 1 and 10 mM KNO_3.***=p<.001, **=p<.01, *=p<.05 as supported by a t-test. WT: n=30; *arf18-9b-1*: n=15; *arf18-9b-2*: n=15; *nlp7b-1*: n=15, *nlp7b-2*: n=15;. Supports Figure 5.

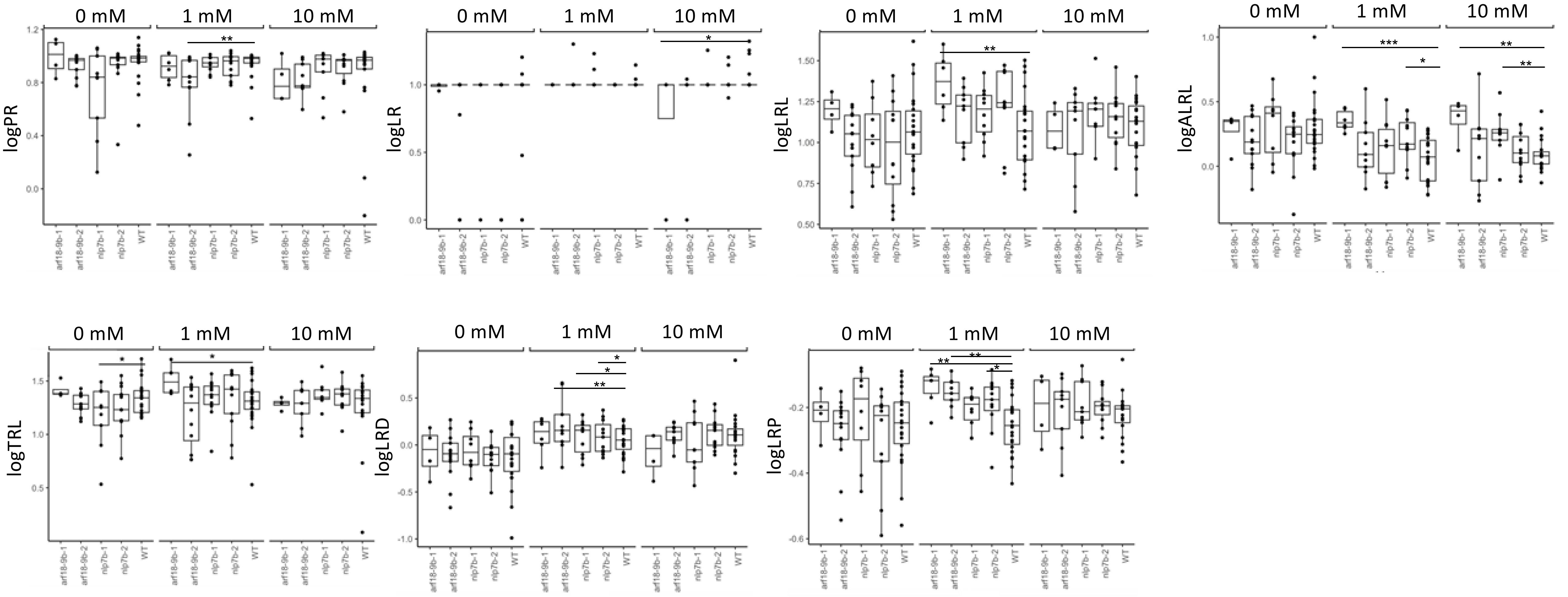

Supplementary Figure S13.
Alignments of the DNA binding domains of tomato and Arabidopsis transcription factors. Grey highlight indicates divergent amino acids. Alignment files are in Supplementary File 3. Supports Figure 6.

Alignment of ARFDNA binding domains

```
AtARF9   FSKVLTASDTSTHGGFSVLRKHATECLPPLDMTQQTPTQELVAEDVHGYQWKFKHIFRGQ
SlARF9B  FCKVLTASDTSTHGGFSILRKHANECLPPLDMTQATPAQELVAKDLHGFEWHFKHIFRGQ
AtARF18  FVKILTASDTSTHGGFSVLRKHATECLPSLDMTQATPTQELVTRDLHGFEWRFKHIFRGQ
SlARF18  FCKILTASDTSTHGGFSVLRKHANECLPQLDMTQATPTQDLVAKDLHGYEWRFKHIFRGQ
AtARF2   FCKTLTASDTSTHGGFSVLRRHADECLPPLDMSRQPPTQELVAKDLHANEWRFRHIFRGQ

AtARF9   PRRHLLTTGWSTFVTSKRLVAGDTFVFLRGENGELRVGVRRAN
SlARF9B  PRRHLLTTGWSTFVSSKRLVTGDSFVFLRSGKGEVRIGIRRLA
AtARF18  PRRHLLTTGWSTFVSSKRLVAGDAFVFLRGENGDLRVGVRRLA
SlARF18  PRRHLLTTGWSTFVTSKRLVAGDAFVFLRDDSGELRVGVRRLA
AtARF2   PRRHLLQSGWSVFVSSKRLVAGDAFIFLRGENGELRVGVRRAM
```

Alignment DREB26 DNA binding domains

```
AtDREB26 KYKGVRMRSWGSWVSEIRAPNQKTRIWLGSYSTAEAAARAYDVALLCLKGPQA--NLNFP
SlDREB26 KYKGVRMRSWGSWVSEIRAPNQKTRIWLGSYSTPEAAARAYDAALLCLKGPSASSNLNFP
```

Alignment of DNA binding domains

```
AtNLP7_DBD/1-82  ----KKKTEKKRGKTEKTISLDVLQQYFTGSLKDAAKSLGVCPTTMKRICRQHGISRWPS
AtNLP6_DBD/1-86  EAKTVKKSERKRGKTEKTISLEVLQQYFAGSLKDAAKSLGVCPTTMKRICRQHGISRWPS
SlNLP7a_DBD/1-83 ---TGKKSERKRGKAEKTISLEVLQQYFAGSLKDAAKSLGVCPTTMKRICRQHGISRWPS
SlNLP7b_DBD/1-84 --TSGKKSERKRGKAEKTISLEVLQQYFAGSLKDAAKSLGVCPTTMKRICRQHGISRWPS

AtNLP7_DBD/1-82  RKIKKVNRSITKLKRVIESVQGTDGG
AtNLP6_DBD/1-86  RKINKVNRSLTRLKHVIDSVQGADGS
SlNLP7a_DBD/1-83 RKINKVNRSLSKLKRVIESVQGADGT
SlNLP7b_DBD/1-84 RKINKVNRSLSKLKCVIESVQGAEGA
```

Supplementary Figure S14. Characterization of N responses in hairy roots. (a) Quantitative Real-Time PCR (qRT-PCR) verifies that the expressions of tomato nitrogen-responsive genes *SlNIR1* and *SlNIR2* are induced by higher nitrogen levels in WT hairy roots. Statistical analysis was performed using one-way ANOVA with Tukey post-hoc test. *p<0.05, ** p<0.01, *** p<0.001; **** p < 0.0001. Values represent the mean and standard error of three biological replicates. **(b)** RNAseq data of canonical N regulatory genes in wild type M82 tomato roots, compared to **(c)** empty-vector transformed hairy roots. x-axis = Concentration of KNO_3; y-axis = Relative change in expression normalized to 0 at 0mM KNO_3. Asterisks indicate differentially expressed among N conditions by limma (false discovery rate <0.05). *** p-value < 0.001, ** p-value < 0.01,* p-value < 0.05, n.s. = not significant. Values represent the mean and standard error of three biological replicates. **(d)** Representative images of tomato hairy roots expressing nuclear localized-GFP driven by the *AtNIR1* and *AtNRP* promoters in 0, 1, and 10 mM KNO_3 (first and second row). Third row showing nuclear localized-GFP expression driven by the AtNRP promoter in the roots of stable tomato transformants. Bar = 100 um. Confocal images were captured using a Zeiss Observer Z1 LSM700 (Zeiss) Confocal Laser Scanning Microscopy (× 20 objective) with excitation at 488 nm and emission at 493–550 nm for GFP and excitation at 555 nm and emission at 560–800 nm for autofluorescence. Supports Figures 7 and 8.

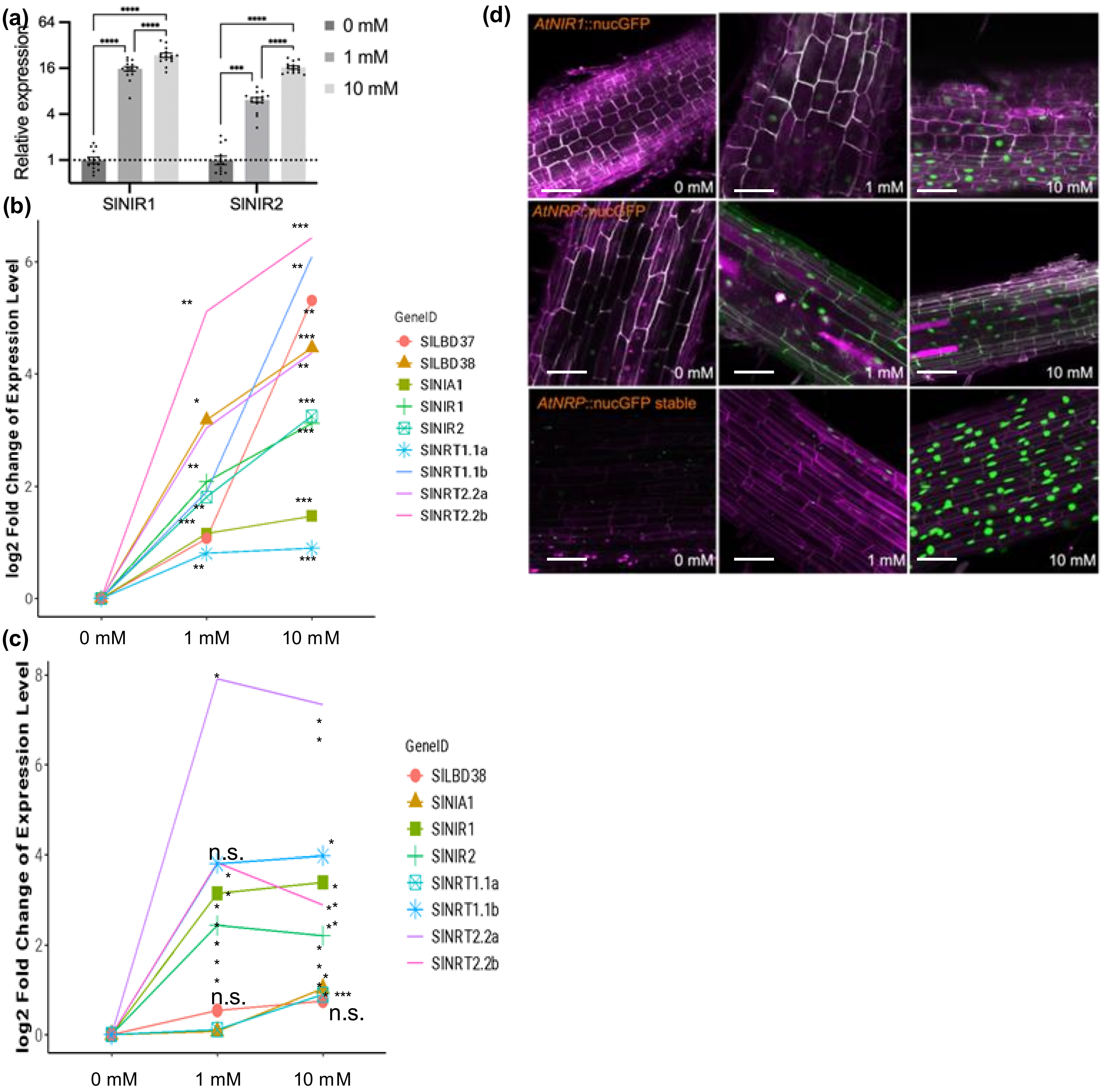

Supplementary Data 15. Expression levels of tomato genes in modified TARGET assays. Values represent the mean and standard error of three biological replicates of which each is the mean of two technical replicates. P-values were calculated using an unpaired two-tailed Student's t-test, *P<0.05, ** P<0.01, *** P<0.001, and **** P<0.0001. N = 3. Supports Figure 7. Arrowheads = activating, Perpendicular arrows = repressing, grey circles indicate gene pairs being tested.

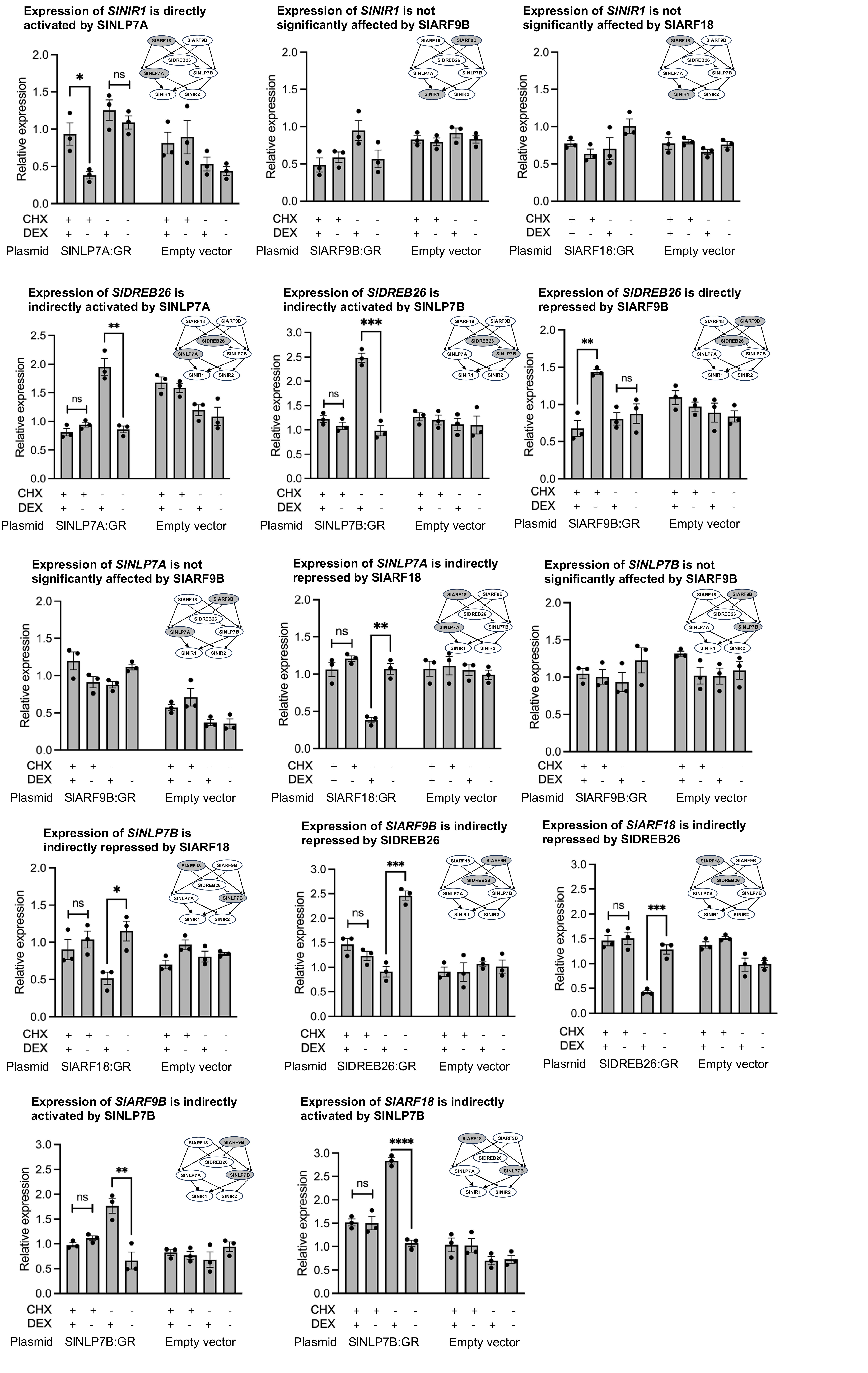

Supplementary Figure S16. Exemplification of the PAROT assay. (a) In PAROT assay, protoplasts are transfected with (i) synthetic promoter (*AtNRP* or 4xNRE) driving the expression of emerald luciferase (ELUC) and (ii) the constitutive NOS promoter driving expression of red luciferase (RLUC). **(b-c)** Viability and representative images of Arabidopsis **(b)** and tomato **(c)** protoplasts before transfection, after transfection, and after overnight incubation in PAROT assay. Protoplasts are stained with fluorescein diacetate (FDA) and observed under a confocal fluorescence microscope with an Argon ion laser (Zeiss, Germany). Viable protoplasts show fluorescence with an excitation at 470/22 nm and emission at 525/50 nm and detector gains used for imaging all were 650. **(d)** Expression levels of endogenous N-responsive genes in WT plants after PAROT assay via qRT-PCR. Values represent the mean and standard error of three biological replicates (independent transfections) and two technical replicates (qPCR assays). **(e)** Expression levels of AtNIR1 in Arabidopsis *nlp7-1* roots and SINIA in tomato *nlp7a/7b* root protoplasts via qRT-PCR. Values represent the mean and standard error of three biological replicates. Values are normalized to 1 mM nitrate treatment in WT roots and protoplasts. **(f)** Changes in normalized luminescence of *NRP*:ELUC and *4xNRE*:ELUC in response to nitrate concentrations in WT Arabidopsis and tomato protoplasts (left) and in mutant protoplasts. Values represent the mean and standard error of three biological replicates, significance tested by t-test (*: p<0.05, **: p<0.01, ***: p<0.001, ****: p<0.0001). Scale bars = 200µm. Supports Figure 8.

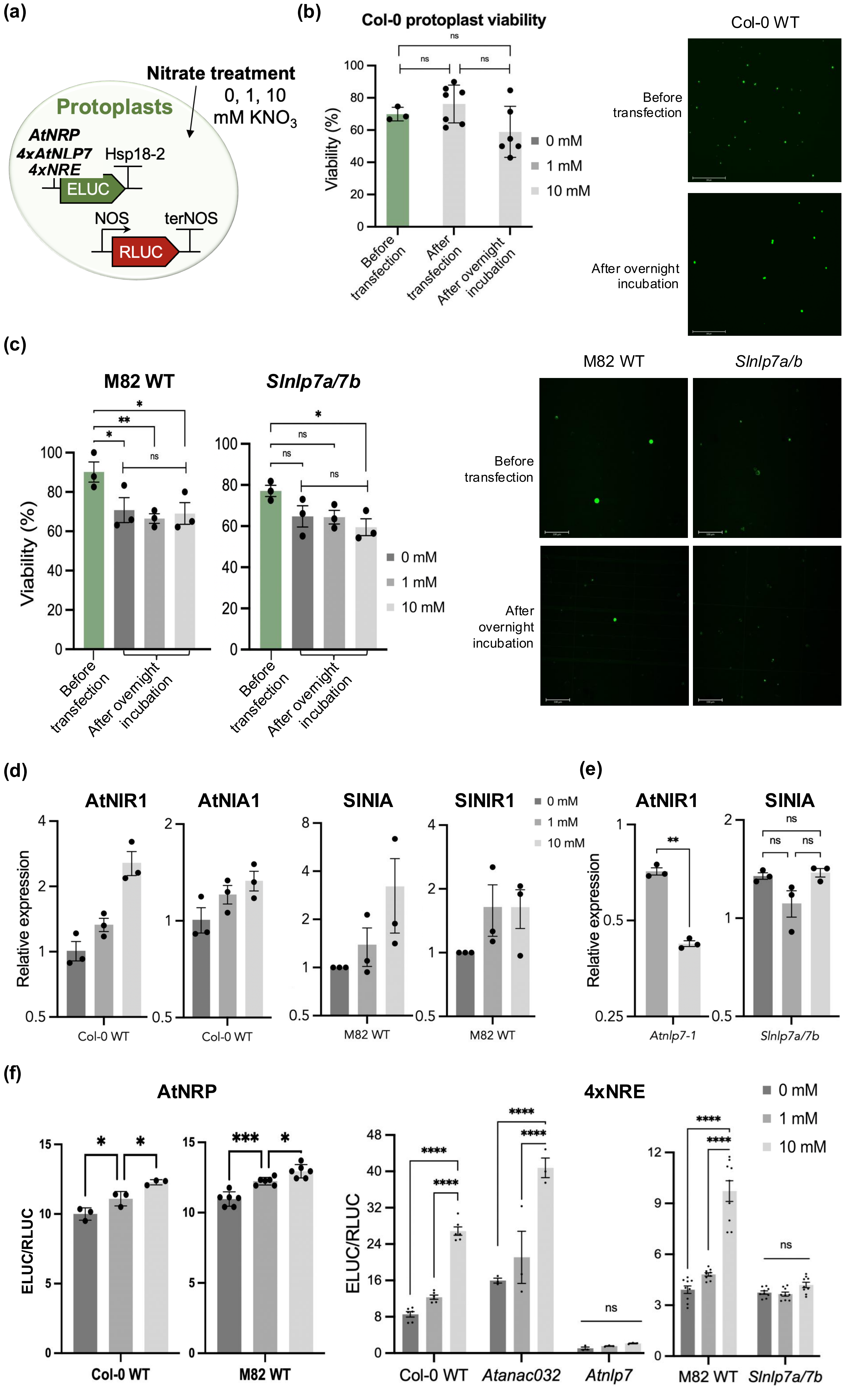

Supplementary Figure S17. Protoplast Assay Reporting Overall Effects of Transcription Factors (PAROT) assay in Arabidopsis and tomato mutants. Eluc/Rluc response ratio of (**a**) Arabidopsis Col-0 WT, *Atanac032*, *Atarf9b, Atarf18-2, dreb26$_c$, Atnlp6,* and *Atnlp7-1* (**b**) Arabidopsis Col-0 WT, *arf18-2/anac032 , anac032/nlp7-1, dreb26$_c$/nlp7$_c$,* and *anac032/nlp7-1* (**c**) tomato M82 WT, *Slarf9b, Slarf18, Sldreb26, Slnlp7a, and Slnlp7b* (**d**) tomato M82 WT, *Slarf9b/18, Slarf9b/18/dreb26, Slnlp7a/7b, Sldreb26/nlp7a/7b, and Slarf9b/18/dreb26/nlp7a/7b* to 0, 1, 10 mM KNO_3. Error bars are standard error; N=4 biological replicates; P-values were calculated using two-way ANOVA with Tukey's multiple comparisons test; *p<0.05, ** p<0.01, *** p<0.001; **** p < 0.0001, ns = not significant. Supports Figure 8.

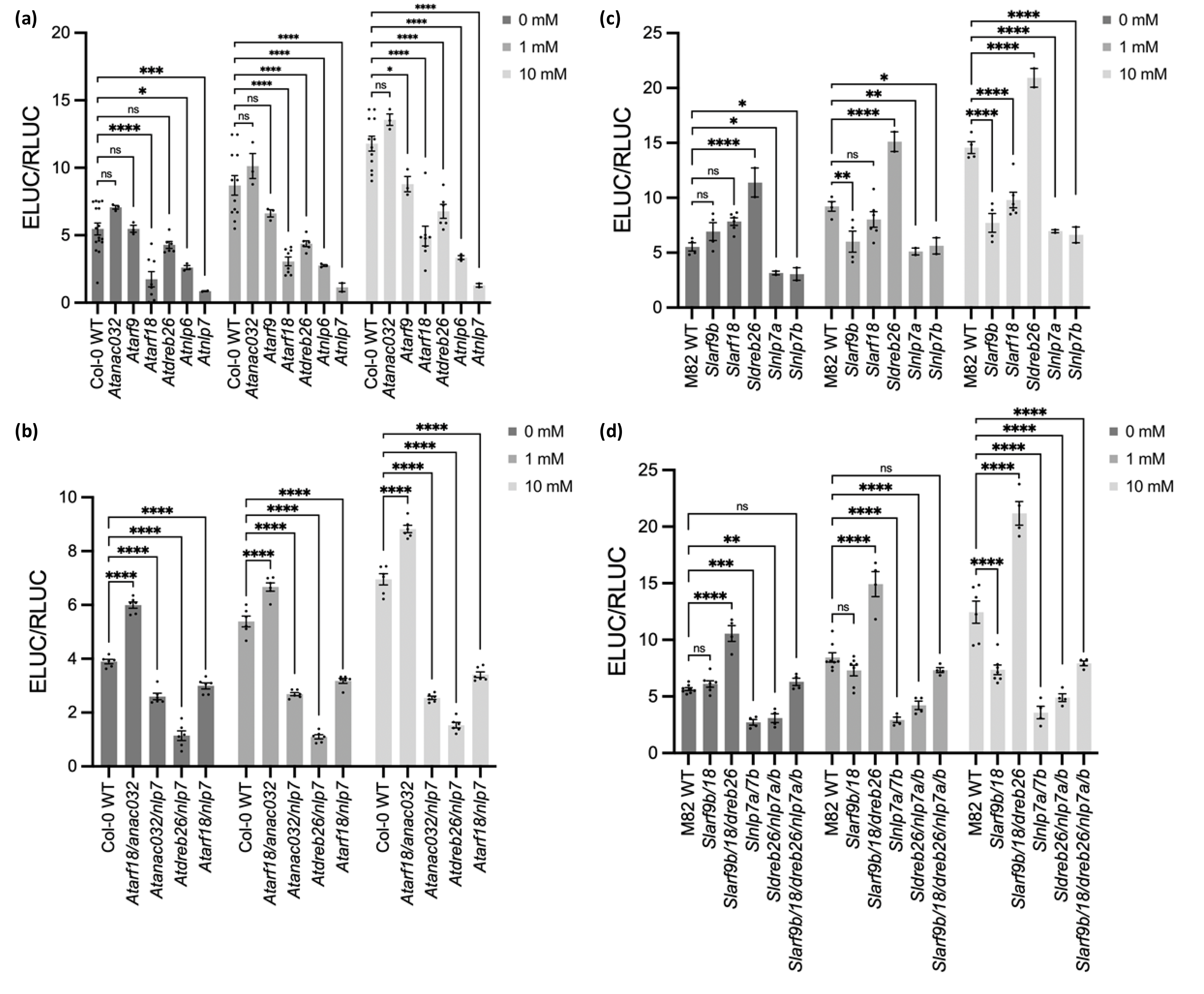

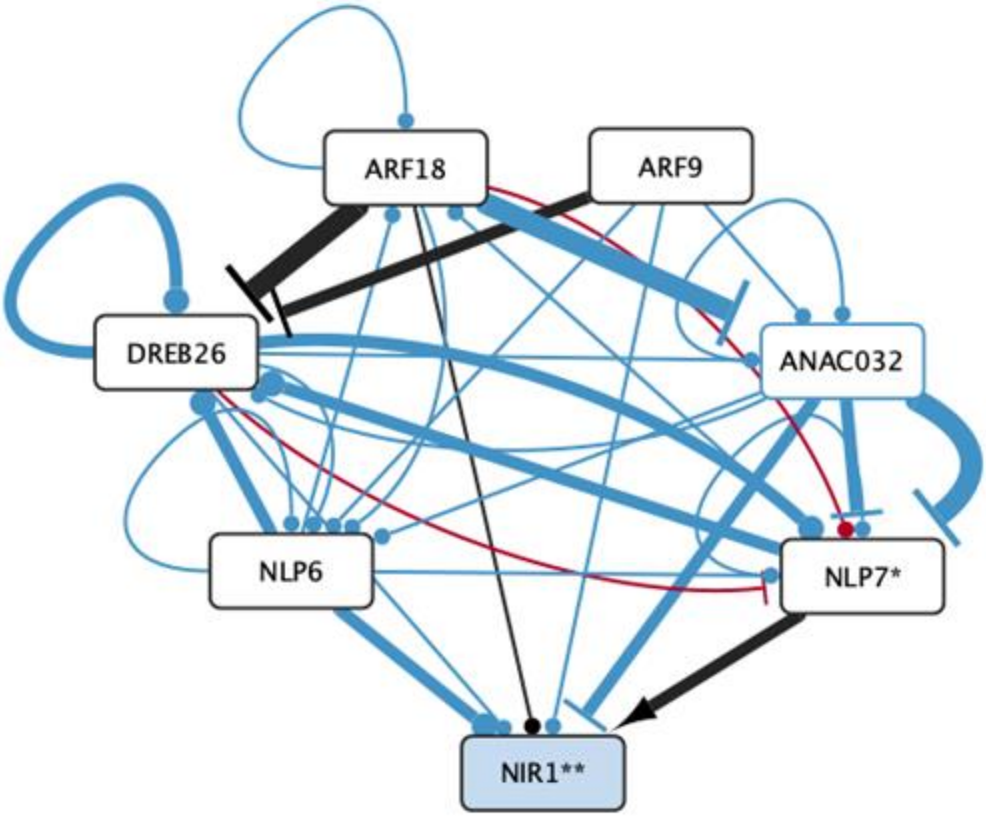

Supplementary Figure S18. Summary of all evidence for regulatory sub-networks. Conserved interactions are indicated with black edges, Arabidopsis-specific interactions in blue, and tomato-specific interactions in red. The thickness of the edge representing the interactions indicates the number of assays in which this interaction was identified, with the thickest edge representing three (yeast one hybrid, in vitro binding assay and Transient Transformation System for Genome-Wide Transcription Factor Target Discovery (TARGET)), and the thinnest edge representing one interaction. A perpendicular line or an arrow indicate that the interaction was demonstrated to be direct and regulatory in nature as determined by TARGET, with the perpendicular line indicating a repressive interaction, while an arrow with an arrowhead indicates activating. The * in NLP7 indicates that two orthologs of NLP7 in tomato appear to be NLP7 paralogs, and the same for NIR1. Supports Figure 9.

Supplementary Figure S19. Primers used in this study. Supports Figures 2, 3, 4, 5, 6, 7, 8

Primers used for qPCR in modified TARGET assays

Gene	Forward primer (5' - 3')	Reverse primer (5' - 3')
AtNIR1	GGCTTATCGACGAACTTGGTG	AGTCTCTCCTCTCCCACTGTT
AtNLP7	TGACCTGATAATTCATTCGAACTCT	AATTTGTCCAAAGAAACCCTAAACA
AtNLP6	TGGATCTGAGAGTTTTGTTTCT	TGATGTGGAGAAGTGTGCTT
eEF-1α	AGATCAACGAGCCCAAGA	CCGTTCCAATACCACCAAT
AtANAC032	TCTGTCGTAAATGCGCGTCG	ACAAAGCCATGTCGGGAAGG
AtDREB26	ACCCTTTTGTCCCCCAAATCC	GAGACGGCTGATGAAGTAGGG
AtARF18	CGACGTCGTTTTAGTGCGTA	TCGCCAACTTGAAAAATTGACAT
AtARF9	CTGTGTTTTGTTGCGCATGG	AACCCTCTCCCCATAAACCC
SlDREB26	AATGAGAAGCTGGGGATCATGG	TGCAGCATCATAGGCTCTAGC
SlNLP7A	TCGTGGAACTTTGGCATTGC	TCACTGCCTCAAGTGCTTTG
SlNLP7B	GATTTTTGCTGCTGCTGCTG	AGCACCGGAAAGTCCATTTC
SlARF9B	ACCATTTGTGGCATCAGTCG	AAATTGCGCTGAGGGAATCC
SlARF18	ATGGAAGGGGTTTGTGTTGG	TTGTTTCGAGGGCAAAGCTC
SlANR1	ATGTCCGCTGATGACACTGG	ACAAACGCTGCACCATTGTC
SlEXP	GCTAAGAACGCTGGACCTAATG	TGGGTGTGCCTTTCTGAATG

Primers used for qPCR in expression assays

Gene	Forward primer (5' - 3')	Reverse primer (5' - 3')
SlEXP	GCTAAGAACGCTGGACCTAATG	TGGGTGTGCCTTTCTGAATG
SlNIR1	TGATGTGGTTGATCGATGAACTGGG	TAGCTCTCCTGAGCCGTACTC
SlNIR2	GCTGATGATGTAGTCCCTGTTTGC	GGCATTCTCTTCACAACTTCTGC
AtNIA1	AGGCTACGCTTATTCTGGAGG	TGTTCGGTTTCTCCTGGTGG

Primers used for nitrate-responsive reporter cloning

Gene	Forward primer (5' - 3')	Reverse primer (5' - 3')
AtNIR1	CCT TTT TTC CAA ACA CGC ACT AAA CC	GAT GAT GGC GGA AGA AGG AGT TG

Primers used for amplification of coding regions for cloning into pENTR™ /D-TOPO™

Gene	Forward primer (5' - 3')	Reverse primer (5' - 3')
SlDREB26	CACCATGGTGAAGACAGAGCAAA	TTAGCAAAAACTCCATAAAGGTA
SlNLP7B	CACCATGTCGGAACCGGGAGGA	TCATTTACCTGAACTCTCACAGGA
SlARF9B	CACCATGGAGAATCAAGGGTCTTT	TCATGTTGAAGCAGTTGTCT

Primers used for genotyping Arabidopsis T-DNA insertional mutant lines

Gene	Reaction	Forward primer (5' - 3')	Reverse primer (5' - 3')
AtNLP7	Wild type reaction	GTTTTTCTTTAGACCGCCACC	AAGAATCAACCGAACAACACG
AtNLP7	T-DNA reaction	ATTTTGCCGATTTCGGAAC	AAGAATCAACCGAACAACACG
AtARF18	Wild type reaction	TCGGAAGTTCTTCGTCAAGTG	TCACCCCTGAAAAACACAAAG
AtARF18	T-DNA reaction	ATATTGACCATCATACTCATTGC	TCACCCCTGAAAAACACAAAG
AtANAC032	Wild type reaction	ACCAACAATTGTGGAAGCAAG	CTCTCCATTTGGAGGTTTTCC
AtANAC032	T-DNA reaction	ATTTTGCCGATTTCGGAAC	CTCTCCATTTGGAGGTTTTCC

Primers used for genotyping Arabidopsis CRISPR edited lines

Gene	Forward primer (5' - 3')	Reverse primer (5' - 3')
AtDREB26	TCACTCTACAAAGCCTGTACG	TCCGAATTGTACCGAGTTTGC
AtANAC032	TGCTTCCTGAAAATAACAACAC	AAACAACAATAAAGGGGAAAATAGG
AtARF18	CAACACGGAATCTGAAAGGAAAGG	TAAACCTCATCTGTTTCATGCTCC
AtNLP7	TGATGAGCCTAGTCCCACCAGA	GCCCCAGACATGAGCATCCACG

Primers used for genotyping of CRISPR lines in tomato hairy roots and stable lines

	Gene	Forward primer (5' - 3')	Reverse primer (5' - 3')
	SlNLP7A	TCCTCTGTCACGGAAGCG TCC GAG CCG GAA GAA GAA ATG	TTTAGCCTCCCCACCGAC GTA GAG AGA GCA TTT CCG TTA GGC
	SlNLP7B	TCCTCTGTCACGGAAGCG GAA CAA AAA TGT CGG AAC CGG G	TTTAGCCTCCCCACCGAC GGC TTA CAT ACA AGC TAC AAA TCT AGG
For Illumina sequencing	SlDREB26	TCCTCTGTCACGGAAGCG CAA TGT CAA TAT CAT CAA TAG CAA CAA GC	TTTAGCCTCCCCACCGAC GGA GAG AGA GTT GTA CAA GTG TGA TG
	SlARF18	TCCTCTGTCACGGAAGCG GTA TGG GAA CTG AGG ATT TGT ATA G	TTTAGCCTCCCCACCGAC GCA CAC AAA CTA AAA GCA ATT ACT ATA G
	SlARF9B	TCCTCTGTCACGGAAGCG GAA TTT GTT GTG GAA AAA GTT TCA GGT G	TTTAGCCTCCCCACCGAC CTT AAC AAA GGC GAT CCC GTA TC
	SlANR1	TCCTCTGTCACGGAAGCG CTT GCT TCT TGG TTA GGG TTT CTC C	TTTAGCCTCCCCACCGAC GCA TTT TCT CCT TTG ACT AGG TGA CC
	SlNLP7A	CAA GTC TCA ATG GAA TCA CAC TCC TC	GTA TAA GCC CGA AGC TTA CAA GTG AC
	SlNLP7B	GCA AGT GGC AAC TTA ATA AGA TTC C	GCT TTC CAT TGT TTC TGT TTC TGC
For Sanger sequencing	SlDREB26	ATG GTG AAG ACA GAG CAA AAA AGT CTA TC	GCA AAA ACT CCA TAA AGG TAT ATC TGC TG
	SlARF18	AGT TCT GTA ACA ACG ACT GCT GAG TTC	AAC AAA GTG AAA GAA CTA CCA GTT GCG
	SlARF9B	GTC AAA CCA GCA GCA TCA GCA AC	GAA TGA GAG GTG GAA CTA ACA ATG GTG G
	SlANR1	ATG TCC GCT GAT GAC ACT GGA TC	CTT CAG CAT TAC AGT CTG TCC GTA G
