# Supplementary material for: Conservation and divergence of regulatory architecture in nitrate-responsive plant gene circuits
Source: Plant Cell. 2025 May 22;37(6):koaf124. doi: 10.1093/plcell/koaf124 (PMC12205479; doi:10.1093/plcell/koaf124)
Supplement: koaf124_Supplementary_Data [file koaf124_supplementary_data.zip › Supplementary Dataset 1.pdf]

Supplemetary Dataset 1				
Sequences of DNA probes used in itranscription factor-DNA binding assays. Supports Figure				
**Species**	**Promoter**	**Probe I**	**Sequence (forward strand)**	
Synthetic	n/a (random	Rnd	TAGCGAAGTACGATCCCATGAA	
Synthetic	Synthetic (DAP-	69	TAGCGAAGTACGATCCCATGAA	(O'Malley et al.,2016)
Arabidopsis	NRE element	20	TAGCGAAGTACGATCCCATCAA	(Konishi and
Arabidopsis	ANAC032SO1 -	71	TAGCGAAGTACGATCCCATGAA	(Allu et al., 2016)
Arabidopsis	DR5 (positive	74	CCTTTTGTCTCCCTTTTGTCTCCC	(Liu et al., 2015)
Arabidopsis	ANAC032	42	TAGCGAAGTACGATCCCCAAGT	
Arabidopsis	ANAC032	43	TAGCGAAGTACGATCCCGGACC	
Arabidopsis	ANAC032	44	TAGCGAAGTACGATCCCGTTAG	
Arabidopsis	ANAC032	50	TAGCGAAGTACGATCCCGTTGG	
Arabidopsis	ANAC032	51	TAGCGAAGTACGATCCCTTGAT	
Arabidopsis	ANAC032	52	TAGCGAAGTACGATCCCTGGTT	
Arabidopsis	ANAC032	53	TAGCGAAGTACGATCCCTGAAC	
Arabidopsis	ANAC032	54	TAGCGAAGTACGATCCCCCAAC	
Arabidopsis	ANAC032	55	TAGCGAAGTACGATCCCTCATG	
Arabidopsis	ANAC032	56	TAGCGAAGTACGATCCCTAAAT	
Arabidopsis	ANAC032	105	TAGCGAAGTACGATCCCCGGGT	
Arabidopsis	ANAC032	107	TAGCGAAGTACGATCCCGAGTT	
Arabidopsis	ANAC032	108	TAGCGAAGTACGATCCCAATCG	
Arabidopsis	ARF18	90	TAGCGAAGTACGATCCCAATTC	
Arabidopsis	ARF18	98	TAGCGAAGTACGATCCCCTGCA	
Arabidopsis	DREB26	61	TAGCGAAGTACGATCCCGAAGA	
Arabidopsis	DREB26	62	TAGCGAAGTACGATCCCGTTTG	
Arabidopsis	DREB26	63	TAGCGAAGTACGATCCCGAGAA	
Arabidopsis	DREB26	64	TAGCGAAGTACGATCCCGTTCA	
Arabidopsis	DREB26	65	TAGCGAAGTACGATCCCCTCCA	
Arabidopsis	DREB26	66	TAGCGAAGTACGATCCCATCAA	
Arabidopsis	DREB26	67	TAGCGAAGTACGATCCCTAAAT	
Arabidopsis	DREB26	79	TAGCGAAGTACGATCCCTATGT	
Arabidopsis	DREB26	80	TAGCGAAGTACGATCCCCTTTA	
Arabidopsis	DREB26	81	TAGCGAAGTACGATCCCGTAAT	
Arabidopsis	DREB26	82	TAGCGAAGTACGATCCCCTCCT	
Arabidopsis	DREB26	83	TAGCGAAGTACGATCCCACATG	
Arabidopsis	DREB26	84	TAGCGAAGTACGATCCCCACTC	
Arabidopsis	DREB26	91	TAGCGAAGTACGATCCCTTGTG	
Arabidopsis	DREB26	103	TAGCGAAGTACGATCCCTGAGA	
Arabidopsis	DREB26	106	TAGCGAAGTACGATCCCAGCTT	
Arabidopsis	NIR1	11	TAGCGAAGTACGATCCCTGTCC	
Arabidopsis	NIR1	12	TAGCGAAGTACGATCCCCAAAT	
Arabidopsis	NIR1	13	TAGCGAAGTACGATCCCAAGAC	
Arabidopsis	NIR1	15	TAGCGAAGTACGATCCCGAAGC	
Arabidopsis	NIR1	16	TAGCGAAGTACGATCCCCACCA	
Arabidopsis	NIR1	17	TAGCGAAGTACGATCCCCTCAG	
Arabidopsis	NIR1	18	TAGCGAAGTACGATCCCAAGAT	
Arabidopsis	NIR1	20	TAGCGAAGTACGATCCCATCAA	
Arabidopsis	NIR1	85	TAGCGAAGTACGATCCCTCACG	
Arabidopsis	NIR1	86	TAGCGAAGTACGATCCCCCCTC	
Arabidopsis	NIR1	92	TAGCGAAGTACGATCCCTATAA	
Arabidopsis	NIR1	99	TAGCGAAGTACGATCCCGAACC	
Arabidopsis	NIR1	100	TAGCGAAGTACGATCCCGAAGT	
Arabidopsis	NLP6	29	TAGCGAAGTACGATCCCTTTACT	
Arabidopsis	NLP6	30	TAGCGAAGTACGATCCCAACAC	
Arabidopsis	NLP6	31	TAGCGAAGTACGATCCCTTTGTT	
Arabidopsis	NLP6	32	TAGCGAAGTACGATCCCTTTGA	
Arabidopsis	NLP6	33	TAGCGAAGTACGATCCCAATAT	
Arabidopsis	NLP6	37	TAGCGAAGTACGATCCCGAAAA	
Arabidopsis	NLP6	38	TAGCGAAGTACGATCCCAACAC	
Arabidopsis	NLP6	39	TAGCGAAGTACGATCCCGATTC	
Arabidopsis	NLP6	87	TAGCGAAGTACGATCCCAATAC	

Arabidopsis	NLP6	93	TAGCGAAGTACGATCCCTGGTT	
Arabidopsis	NLP6	94	TAGCGAAGTACGATCCCGAAGG	
Arabidopsis	NLP6	101	TAGCGAAGTACGATCCCTCTCC	
Arabidopsis	NLP7	22	TAGCGAAGTACGATCCCTTCTC	
Arabidopsis	NLP7	23	TAGCGAAGTACGATCCCGAATC	
Arabidopsis	NLP7	24	TAGCGAAGTACGATCCCCTGGG	
Arabidopsis	NLP7	25	TAGCGAAGTACGATCCCTGTTT	
Arabidopsis	NLP7	26	TAGCGAAGTACGATCCCACTTC	
Arabidopsis	NLP7	27	TAGCGAAGTACGATCCCTGGTA	
Arabidopsis	NLP7	28	TAGCGAAGTACGATCCCGAATC	
Arabidopsis	NLP7	88	TAGCGAAGTACGATCCCAAGCA	
Arabidopsis	NLP7	89	TAGCGAAGTACGATCCCATTCC	
Arabidopsis	NLP7	95	TAGCGAAGTACGATCCCGAATC	
Arabidopsis	NLP7	102	TAGCGAAGTACGATCCCCGATG	
Tomato	SlNIR1	S01	TAGCGAAGTACGATCCCCTTATT	
Tomato	SlNIR1	S02	TAGCGAAGTACGATCCCGACCA	
Tomato	SlNIR2	S03	TAGCGAAGTACGATCCCGTAAT	
Tomato	SlNIR2	S04	TAGCGAAGTACGATCCCGTATG	
Tomato	SlNLP7-1	S05	TAGCGAAGTACGATCCCCTTCG	
Tomato	SlNLP7-1	S06	TAGCGAAGTACGATCCCAGGAC	
Tomato	SlNLP7-1	S07	TAGCGAAGTACGATCCCTTTGC	
Tomato	SlNLP7-3	S08	TAGCGAAGTACGATCCCCAAGT	
Tomato	SlNLP7-3	S09	TAGCGAAGTACGATCCCATTTC	
Tomato	SlNLP7-3	S10	TAGCGAAGTACGATCCCTAAAT	
Tomato	SlNLP7-3	S11	TAGCGAAGTACGATCCCCAACC	
Tomato	SlNLP7-3	S12	TAGCGAAGTACGATCCCATTTG	
Tomato	SlDREB26	S13	TAGCGAAGTACGATCCCCAACA	
Tomato	SlDREB26	S14	TAGCGAAGTACGATCCCCTGGA	
Tomato	SlDREB26	S15	TAGCGAAGTACGATCCCAGCTA	
Tomato	SlDREB26	S16	TAGCGAAGTACGATCCCGAAAT	
Tomato	SlDREB26	S17	TAGCGAAGTACGATCCCAGGTA	
