# Supplementary material for: Conservation and divergence of regulatory architecture in nitrate-responsive plant gene circuits
Source: Plant Cell. 2025 May 22;37(6):koaf124. doi: 10.1093/plcell/koaf124 (PMC12205479; doi:10.1093/plcell/koaf124)
Supplement: koaf124_Supplementary_Data [file koaf124_supplementary_data.zip › Supplementary Dataset 2.pdf]

Supplementary Dataset 2. Plasmids used in this study Supports Figures 2, 3, 5, 6, 7, 8

Plasmids for expression of transcription factor recombinant proteins used for *in vitro* binding assays

Addgene#	Plasmid code	Description	Acceptor	Plasmid type	Source of plasmid
196143	pEPYCeGM0009	pENTR_AtNLP6_HiBit	pDONR207	Gateway Entry	This study
196144	pEPYCeGM0010	pENTR_AtNLP7_HiBit	pDONR207	Gateway Entry	This study
196145	pEPYCeGM0011	pENTR_AtDREB26_HiBit	pDONR207	Gateway Entry	This study
196146	pEPYCeGM0012	pENTR_AtNAC032_HiBit	pDONR207	Gateway Entry	This study
196147	pEPYCeGM0022	pENTR_AtARF18_HiBit	pDONR207	Gateway Entry	This study
196150	pEPYCdKN0009	pH9GW_9xHis_AtNLP6_HiBit	pH9GW	Gateway Expressio	This study
196151	pEPYCdKN0010	pH9GW_9xHis_AtNLP7_HiBit	pH9GW	Gateway Expressio	This study
196152	pEPYCdKN0011	pH9GW_9xHis_AtDREB26_HiBit	pH9GW	Gateway Expressio	This study
196153	pEPYCdKN0012	pH9GW_9xHis_AtNAC032_HiBit	pH9GW	Gateway Expressio	This study
196154	pEPYCdKN0022	pH9GW_9xHis_AtARF18_HiBit	pH9GW	Gateway Expressio	This study

Plasmids used for modified TARGET assays

Arabidopsis

Addgene#	Plasmid code	Description	Level 0 Parts					Cloning overhang		Source of plasmid
			PROM	CDS	CTAG	3UTR/TERM	Acceptor	5'	3'	
197549	pEPOZ1KN0142	2xCaMV35s::TMV::AtNLP6:GR::35sT	pICH51288	pEPSW0CM0073	pEPOZ0CM0137	pICH41414	pCK2 (Addgene 136696)	GCA	TAC	This study
197550	pEPOZ1KN0143	2xCaMV35s::TMV::AtNLP7:GR::35sT	pICH51288	pEPSW0CM0074	pEPOZ0CM0137	pICH41414	pCK2 (Addgene 136696)	GCA	TAC	This study
197551	pEPOZ1KN0144	2xCaMV35s::TMV::AtDREB26:GR::35sT	pICH51288	pEPSW0CM0075	pEPOZ0CM0137	pICH41414	pCK2 (Addgene 136696)	GCA	TAC	This study
197552	pEPOZ1KN0145	2xCaMV35s::TMV::AtANAC032:GR::35s	pICH51288	pEPSW0CM0076	pEPOZ0CM0137	pICH41414	pCK2 (Addgene 136696)	GCA	TAC	This study
197554	pEPOZ1KN0147	2xCaMV35s::TMV::AtARF18:GR::35sT	pICH51288	pEPOZ0CM0138	pEPOZ0CM0137	pICH41414	pCK2 (Addgene 136696)	GCA	TAC	This study
197555	pEPOZ1KN0148	2xCaMV35s::TMV::AtARF9:GR::35sT	pICH51288	pEPOZ0CM0139	pEPOZ0CM0137	pICH41414	pCK2 (Addgene 136696)	GCA	TAC	This study

Tomato

Addgene#	Plasmid code	Description	Acceptor	Plasmid type	Source of
	pGD0001	pBEACON_SlNLP7A_GR	pBEACON	Gateway Expressio	This study
	pGD0002	pBEACON_SlNLP7B_GR	pBEACON	Gateway Expressio	This study
	pGD0003	pBEACON_SlARF9B_GR	pBEACON	Gateway Expressio	This study
	pGD0004	pBEACON_SlARF18_GR	pBEACON	Gateway Expressio	This study
	pGD0005	pBEACON_DREB26_GR	pBEACON	Gateway Expressio	This study
	pENTR-D-SlNLP7A-CDS	SlNLP7A-CDS sequence in cloning	pENTRY	Gateway Entry	This study
	pBEACON-5C-SlNLP7A-CDS	35S::GR-SlNLP7A-CDS, correct	pBEACON	Gateway Expressio	This study
	pUC57-SlARF18-CDS	SlARF18-CDS sequence in cloning	pUC57	Gateway Entry	This study
	pBEACON-5C-SlARF18-CDS	35S::GR-SlARF18-CDS	pBEACON	Gateway Expressio	This study
	pENTR-D-SlNLP7B-CDS	SlNLP7B-CDS in cloning vector	pENTRY	Gateway Entry	This study
	pBEACON-5C-SlNLP7B-CDS	35S::GR-SlNLP7B-CDS	pBEACON	Gateway Expressio	This study

Plasmids used for protoplast co-expression luciferase assays

Addgene#	Plasmid code	Description	Level 0 Parts						Cloning overhang		Source of plasmid
			PROM	5UTR	CDS	CTAG	3UTR/TERM	Acceptor	5'	3'	
154629	pEPYC1CB0003	AtuNos::TMV::LucF::FLAG::nosT	pICH42211	pICH41402	pEPAS0CM0008	pICSL50007	pICH41421	pICH47732 (Addgene 48000	TGCC	GCAA	Cat et al., 2020
197536	pEPSW1KN0070	CaMV35s::TMV::LucN::FLAG::35sT	pICH51277	-	pEPYC0CM0133	pICSL50007	pICH41414	pCK1 (Addgene 136695)	ATG	GCA	This study
196178	pEPSW1KN0034	CaMV35s::TMV::LucF::FLAG::35sT	pICH51277	-	pEPAS0CM0008	pICSL50007	pICH41414	pCK1 (Addgene 136695)	ATG	GCA	This study
196177	pEPSW1KN0035	AtuNos::TMV::LucN::FLAG::nosT	pICH42211	pICH41402	pEPYC0CM0133	pICSL50007	pICH41421	pCK1 (Addgene 136695)	ATG	GCA	This study
196174	pEPSW1KN0014	AtANAC32::LucN::FLAG::nosT	pEPSW0CM0014	-	pEPYC0CM0133	pICSL50007	pICH41421	pCK1 (Addgene 136695)	ATG	GCA	This study
197537	pEPSW1KN0016	ANR1::LucN::FLAG::nosT	pEPSW0CM0016	-	pEPYC0CM0133	pICSL50007	pICH41421	pCK1 (Addgene 136695)	ATG	GCA	This study
196176	pEPSW1KN0018	ARF18::LucN::FLAG::nosT	pEPSW0CM0018	-	pEPYC0CM0133	pICSL50007	pICH41421	pCK1 (Addgene 136695)	ATG	GCA	This study
196175	pEPSW1KN0020	NLP6::LucN::FLAG::nosT	pEPSW0CM0020	-	pEPYC0CM0133	pICSL50007	pICH41421	pCK1 (Addgene 136695)	ATG	GCA	This study
197538	pEPSW1KN0022	DREB26::LucN::FLAG::nosT	pEPSW0CM0022	-	pEPYC0CM0133	pICSL50007	pICH41421	pCK1 (Addgene 136695)	ATG	GCA	This study
197539	pEPSW1KN0024	NLP7::LucN::FLAG::nosT	pEPSW0CM0024	-	pEPYC0CM0133	pICSL50007	pICH41421	pCK1 (Addgene 136695)	ATG	GCA	This study
197540	pEPSW1KN0025	NIR1::LucN::FLAG::nosT	pEPSW0CM0025	-	pEPYC0CM0133	pICSL50007	pICH41421	pCK1 (Addgene 136695)	ATG	GCA	This study
197541	pEPSW1KN0027	CaMV35s::TMV::NLP6::35sT	pICH51277	-	pEPSW0CM0027	-	pICH41414	pCK2 (Addgene 136696)	GCA	TAC	This study
197542	pEPSW1KN0029	CaMV35s::TMV::NLP7::35sT	pICH51277	-	pEPSW0CM0029	-	pICH41414	pCK2 (Addgene 136696)	GCA	TAC	This study
197543	pEPSW1KN0030	CaMV35s::TMV::DREB26::35sT	pICH51277	-	pEPSW0CM0030	-	pICH41414	pCK2 (Addgene 136696)	GCA	TAC	This study
197544	pEPSW1KN0031	CaMV35s::TMV::ANAC032::35sT	pICH51277	-	pEPSW0CM0031	-	pICH41414	pCK2 (Addgene 136696)	GCA	TAC	This study
197545	pEPSW1KN0032	CaMV35s::TMV::ARF18::35sT	pICH51277	-	pEPSW0CM0032	-	pICH41414	pCK2 (Addgene 136696)	GCA	TAC	This study
197547	pEPSW1KN0113	CaMV35s::TMV::ARF9::35sT	pICH51277	-	pEPSW0CM0112	-	pICH41414	pCK2 (Addgene 136696)	GCA	TAC	This study
196179	pEPOR1CB0068	CaMV35s::TMV::YFP:NLS::35sT	pICH51277	-	pEPOR0CM0010	-	pICH41414	pICH47761 (Addgene 48003	TTAC	CAGA	This study

Plasmids used for nitrate reporter assay

Addgene#	Plasmid code	Description	Acceptor	Plasmid type	Source of
	pENTR5'-NRPpro	NRP promoter in cloning vector	pENTRY	Gateway Entry	This study
	pMR105-NRPpro	NRP promoter reporter	pMR105	Gateway Expressio	This study
	pENTR5'-AtNIR1pro	AtNIR1 promoter in cloning vector	pENTRY	Gateway Entry	This study
	pMR105-AtNIR1pro	AtNIR1 promoter reporter	pMR105	Gateway Expressio	This study

Plasmids used for PAROT assays

Addgene#	Plasmid code	Description	Acceptor	Plasmid type	Source of
170887	pGD0006	pDGB2a_pNOS_RedF_NOS	pDGB2a	Expression	Gonzalez-
	pGD0007	pDGBa1_NRP_ELuc_THsp18-2	pDGBa1	Expression	This study

Plasmids used to create CRISPR knockout lines

Arabidopsis

Plasmid code	Description	Cloning overhang		Source of plasmid
		5'	3'	
pEPOZ1KN0004	AtuNOSpro::TMV::NPTII_domesticated(pEPOZ0CM0001)::AtuOCSter (backbone pCk4)	CAG (SapI)	GGT (SapI)	This study
pEPOZ1KN0005	expression cassette for SpCas9, AtYAOpro::SpCas9::RBCS-E9ter (reverse orientation)	GCA (SapI)	TAC (SapI)	This study
pEPOZ1KN0008	expression cassette for SaCas9, AtRPS5Apro::SaCas9::RBCS-E9ter (reverse orientation)	GCA (SapI)	TAC (SapI)	This study

pEPOZ3KN0033	SpCas9, KanR, and 8 guides (2 guides per gene, 4 genes, ARF18, DREB26, NLP7, ANAC032)	GCA	TAC	This study
pEPOZ3KN0103	Level 3 contains FASTred, SaCas9, KanR, and 10 guides (2 guides per gene, 5 genes, ARF18, DREB26, NLP7,	GCA	TAC	This study
**Tomato**				
**Plasmid code**	**Description**	**Cloning overhang**		**Source of plasmid**
		**5'**	**3'**	
pUAP4-AtHSP18.2 ter	L0 plasmid of AtHSP18.2	GCTT	CGCT	This study
pCK4-35S-BAR-NOS	L1 plasmid of Basta resistence	CAG	GGT	This study
pCK2R-proRPS5A-SpCas9-HSP18	L1 plasmid of Cas9 expression	GCA	TAC	This study
pCsA-SlNLP7A-NPTII	Binary plasmid of CRISPR-SlNLP7A, includes FASTgreen, SpCas9, 2 gRNAs, Kanamycin selection	GGAG	TACT	This study
pCsB-SlNLP7B-NPTII	Binary plasmid of CRISPR-SlNLP7B, includes FASTgreen, SpCas9, 2 gRNAs, Kanamycin selection	TACT	AATG	This study
pCsA-SlARF18-NPTII	Binary plasmid of CRISPR-SlARF18, includes FASTgreen, SpCas9, 2 gRNAs, Kanamycin selection	GGAG	TACT	This study
pCsB-SlARF9B-NPTII	Binary plasmid of CRISPR-SlARF9B, includes FASTgreen, SpCas9, 2 gRNAs, Kanamycin selection	TACT	AATG	This study
pCSA-SlANR1-NPTII	Binary plasmid of CRISPR-SlANR1, includes FASTgreen, SpCas9, 2 gRNAs, Kanamycin selection	GGAG	TACT	This study
PCsA-DREB26-NPTII	Binary plasmid of CRISPR-SlDREB26, includes FASTgreen, SpCas9, 2 gRNAs, Kanamycin selection	GGAG	TACT	This study
PCsA-ANR1-NPTII	Binary plasmid of CRISPR-SlANR1, includes FASTgreen, SpCas9, 2 gRNAs, Kanamycin selection	GGAG	TACT	This study
pCsA-ARF-NPTII	Binary plasmid of CRISPR-SlARF18-SlARF9B, includes FASTgreen, SpCas9, 2 gRNAs each gene, Kanamycin selection	GGAG	TACT	This study
pCsA-ARF-BASTA	Binary plasmid of CRISPR-SlARF18-SlARF9B, includes FASTgreen, SpCas9, 2 gRNAs each gene, Basta selection	GGAG	TACT	This study
pCsA-NLP-BASTA	Binary plasmid of CRISPR-SlNLP7A-SlNLP7B, includes FASTgreen, SpCas9, 2 gRNAs each gene, Basta selection	GGAG	TACT	This study
pCsA-NLP-NPTII	Binary plasmid of CRISPR-SlNLP7A-SlNLP7B, includes FASTgreen, SpCas9, 2 gRNAs each gene, Kanamycin selection	GGAG	TACT	This study
PCSA-SlANR1-SlDREB26-NPTII	Binary plasmid of CRISPR-SlDREB26-SlANR1, includes FASTgreen, SpCas9, 2 gRNAs each gene, Kanamycin selection	GGAG	TACT	This study
pCSA-SlARF9B-DREB26-ANR1-NPTII	Binary plasmid of CRISPR-SlARF9B-SlDREB26-SlANR1, includes FASTgreen, SpCas9, 2 gRNAs each gene, Kanamycin selection	GGAG	TACT	This study
pCSA-SlARF9B-DREB26-ANR1-BAR	Binary plasmid of CRISPR-SlARF9B-SlDREB26-SlANR1, includes FASTgreen, SpCas9, 2 gRNAs each gene, Basta selection	GGAG	TACT	This study
pCSA-SlARF18-DREB26-ANR1-NPTII	Binary plasmid of CRISPR-SlARF18-SlDREB26-SlANR1, includes FASTgreen, SpCas9, 2 gRNAs each gene, Kanamycin selection	GGAG	TACT	This study

pCSA-SlARF18-DREB26-ANR1-BAR	Binary plasmid of CRISPR-SlARF18-SlDREB26-SlANR1, includes FASTgreen, SpCas9, 2 gRNAs each gene, Basta selection	GGAG	TACT	This study
PCSB-SlNLP7A-7B-SlDREB26-NPTII	Binary plasmid of CRISPR-SlDREB26-SlNLP7A-7B, includes FASTgreen, SpCas9, 2 gRNAs each gene, Kanamycin selection	TACT	AATG	This study
PCSA-SlARF18-9B-SlDREB26-NPTII	Binary plasmid of CRISPR-SlARF18-9B-SlDREB26, includes FASTgreen, SpCas9, 2 gRNAs each gene, Kanamycin selection	GGAG	TACT	This study
PCSA-SlARF18-9B-SlNLP7A-7B-NPTII	Binary plasmid of CRISPR-SlARF18-9B-SlNLP7A-7B, includes FASTgreen, SpCas9, 2 gRNAs each gene, Kanamycin selection	GGAG	TACT	This study
pCSA-SlARF18-SlARF9B-DREB26-ANR1-	Binary plasmid of CRISPR-SlARF18-9B-SlDREB26-SlANR1, includes FASTgreen, SpCas9, 2 gRNAs each gene, Kanamycin selection	GGAG	TACT	This study
pCSA-SLARF18-SlARF9B-DREB26-ANR1-	Binary plasmid of CRISPR-SlARF18-9B-SlDREB26-SlANR1, includes FASTgreen, SpCas9, 2 gRNAs each gene, Basta selection	GGAG	TACT	This study
pCSA-SlARF9B-DREB26-NLP7A-7B-NPTII	Binary plasmid of CRISPR-SlARF9B-SlDREB26-SlNLP7A-7B, includes FASTgreen, SpCas9, 2 gRNAs each gene, Kanamycin selection	GGAG	TACT	This study
pCSA-SlARF9B-DREB26-NLP7A-7B-BAR	Binary plasmid of CRISPR-SlARF9B-SlDREB26-SlNLP7A-7B, includes FASTgreen, SpCas9, 2 gRNAs each gene, Basta selection	GGAG	TACT	This study
pCSA-SlARF18-DREB26-NLP7A-7B-NPTII	Binary plasmid of CRISPR-SlARF18-SlDREB26-SlNLP7A-7B, includes FASTgreen, SpCas9, 2 gRNAs each gene, Kanamycin selection	GGAG	TACT	This study
pCSA-SlARF18-DREB26-NLP7A-7B-BAR	Binary plasmid of CRISPR-SlARF18-SlDREB26-SlNLP7A-7B, includes FASTgreen, SpCas9, 2 gRNAs each gene, Basta selection	GGAG	TACT	This study
pCSA-SlARF18-9B-DREB26-NLP7A-7B-N	Binary plasmid of CRISPR-SlARF18-9B-SlDREB26-SlNLP7A-7B, includes FASTgreen, SpCas9, 2 gRNAs each gene, Kanamycin selection	GGAG	TACT	This study
pCSA-SlARF18-9B-DREB26-NLP7A-7B-B	Binary plasmid of CRISPR-SlARF18-9B-SlDREB26-SlNLP7A-7B, includes FASTgreen, SpCas9, 2 gRNAs each gene, Basta selection	GGAG	TACT	This study

Level 0 Phytobricks used in the assemblies above

Addgene#	Plasmid code	Part type	Description	Compatibility with Assembly Systems	Cloning overhang (top strand) 5'	3'	Source of plasmid
	pEPOZ0CM0001	CDS	NPTII CDS (Kanamycin resistance	MoClo, Loop GB	AATG	GCTT	This study
	pEPOZ0CM0039	PROM+5UTR	NRP promoter, 364 bp, Level 0, nitrogen responsive promoter	MoClo, Loop GB	GGAG	AATG	This study
196162	pEPSW0CM0014	PROM+5UTR	AtANAC032 (AT1G77450) 1000 bp upstream of TSS (1 SNP to remove BsaI)	MoClo, Loop GB	GGAG	TACT	This study
196164	pEPSW0CM0018	PROM+5UTR	AtARF18 (AT3G61830) 1000 bp upstream of TSS	MoClo, Loop GB	GGAG	TACT	This study
196163	pEPSW0CM0020	PROM+5UTR	AtNLP6 (AT1G64530) 1000 bp upstream of TSS	MoClo, Loop GB	GGAG	TACT	This study

197517	pEPSW0CM0022	PROM+5UTR	AtDREB26 (AT1G21910) 1000 bp upstream of TSS	MoClo, Loop GB	GGAG	TACT	This study
197518	pEPSW0CM0024	PROM+5UTR	AtNLP7 (AT4G24020) 1000 bp upstream of TSS	MoClo, Loop GB	GGAG	TACT	This study
197519	pEPSW0CM0025	PROM+5UTR	AtNIR1 (AT2G15620) 936 bp upstream of TSS	MoClo, Loop GB	GGAG	TACT	This study
197520	pEPSW0CM0027	CDS	AtNLP6	MoClo, Loop GB	AATG	GCTT	This study
197521	pEPSW0CM0029	CDS	AtNLP7	MoClo, Loop GB	AATG	GCTT	This study
197522	pEPSW0CM0030	CDS	AtDREB26	MoClo, Loop GB	AATG	GCTT	This study
197523	pEPSW0CM0031	CDS	AtANAC032	MoClo, Loop GB	AATG	GCTT	This study
197524	pEPSW0CM0032	CDS	AtARF18	MoClo, Loop GB	AATG	GCTT	This study
197526	pEPSW0CM0112	CDS	AtARF9	MoClo, Loop GB	AATG	GCTT	This study
197528	pEPSW0CM0073	CDS	NLP6 (no stop codon)	MoClo, Loop GB	AATG	TTCG	This study
197529	pEPSW0CM0074	CDS	NLP7 (no stop codon)	MoClo, Loop GB	AATG	TTCG	This study
197530	pEPSW0CM0075	CDS	DREB26 (no stop codon)	MoClo, Loop GB	AATG	TTCG	This study
197531	pEPSW0CM0076	CDS	ANAC032 (no stop codon)	MoClo, Loop GB	AATG	TTCG	This study
197533	pEPOZ0CM0138	CDS	ARF18 (no stop codon)	MoClo, Loop GB	AATG	TTCG	This study
197534	pEPOZ0CM0139	CDS	ARF9 (no stop codon)	MoClo, Loop GB	AATG	TTCG	This study
197535	pEPOZ0CM0137	CTAG	C-terminal glucocorticoid receptor, GR	MoClo, Loop GB	TTCG	GCTT	This study
50268	pICH51277	PROM+5UTR	CaMV35s_TMV	MoClo, Loop GB	GGAG	AATG	Engler et al 2014
50255	pICH42211	PROM	NOS promoter (Agrobacterium tumefac	MoClo, Loop GB	GGAG	TACT	Engler et al 2014
50271	pICH87633	PROM+5UTR	AtuNos_TMV	MoClo, Loop GB	GGAG	AATG	Engler et al 2014
50269	pICH51288	PROM+5UTR	2xCaMV35s_TMV	MoClo, Loop GB	GGAG	AATG	Engler et al 2014
50285	pICH41402	5UTR	TMVΩ (Tobacco Mosaic Virus)	MoClo, Loop GB	TACT	AATG	Engler et al 2014
154595	pEPYC0CM0133	CDS	LucN (NanoLuc)	MoClo, Loop GB	AATG	TTCG	Cai et al 2020
154594	pEPAS0CM0008	CDS	LucF (Firefly luciferase)	MoClo, Loop GB	AATG	TTCG	Cai et al 2020
50308	pICSL50007	CTAG	C terminal FLAG tag	MoClo, Loop GB	TTCG	GCTT	Engler et al 2014
50337	pICH41414	3UTR_TERM	35S terminator (Cauliflower Mosaic Vir	MoClo, Loop GB	GCTT	CGCT	Engler et al 2014
50339	pICH41421	3UTR_TERM	NOS terminator (Agrobacterium tumefa	MoClo, Loop GB	GCTT	CGCT	Engler et al 2014
50343	pICH41432	3UTR_TERM	OCS terminator (Agrobacterium tumefa	MoClo, Loop GB	GCTT	CGCT	Engler et al 2014
