# Supplementary material for: Conservation and divergence of regulatory architecture in nitrate-responsive plant gene circuits
Source: Plant Cell. 2025 May 22;37(6):koaf124. doi: 10.1093/plcell/koaf124 (PMC12205479; doi:10.1093/plcell/koaf124)
Supplement: koaf124_Supplementary_Data [file koaf124_supplementary_data.zip › Supplementary File S1.pdf]

Supplementary File S1. Statistics for the root system architecture analysis.
Supports Figure 5.

```
##################################################
#                                                #
#T-tests between each genotype within a treatment#
#                                                #
##################################################
[1] "anac032-1"
$logPR

	Two Sample t-test

data:  x by dataset_anac0321_1mM$Genotype
t = -0.44838, df = 36, p-value = 0.6566
alternative hypothesis: true difference in means between group anac032-1 and group Col-0 is not equal to 0
95 percent confidence interval:
 -0.11119815  0.07093187
sample estimates:
mean in group anac032-1     mean in group Col-0 
               1.325065                1.345198 


$logLR

	Two Sample t-test

data:  x by dataset_anac0321_1mM$Genotype
t = 0.22151, df = 36, p-value = 0.8259
alternative hypothesis: true difference in means between group anac032-1 and group Col-0 is not equal to 0
95 percent confidence interval:
 -0.2899639  0.3610712
sample estimates:
mean in group anac032-1     mean in group Col-0 
               1.036825                1.001272 


$logLRL

	Two Sample t-test

data:  x by dataset_anac0321_1mM$Genotype
```

t = 0.69392, df = 36, p-value = 0.4922
alternative hypothesis: true difference in means between group anac032-1 and group Col-0 is not equal to 0
95 percent confidence interval:
 -0.2053797 0.4190221
sample estimates:
mean in group anac032-1   mean in group Col-0
            0.2964885   0.1896673

$logALRL

          Two Sample t-test

data: x by dataset_anac0321_1mM$Genotype
t = 0.58434, df = 36, p-value = 0.5626
alternative hypothesis: true difference in means between group anac032-1 and group Col-0 is not equal to 0
95 percent confidence interval:
 -0.176082 0.318617
sample estimates:
mean in group anac032-1   mean in group Col-0
           -0.7403369   -0.8116044

$logTRL

          Two Sample t-test

data: x by dataset_anac0321_1mM$Genotype
t = 0.17596, df = 36, p-value = 0.8613
alternative hypothesis: true difference in means between group anac032-1 and group Col-0 is not equal to 0
95 percent confidence interval:
 -0.1050800 0.1250457
sample estimates:
mean in group anac032-1   mean in group Col-0
             1.645463   1.635480

$logLRD

          Two Sample t-test

data: x by dataset_anac0321_1mM$Genotype
t = 0.89401, df = 36, p-value = 0.3773
alternative hypothesis: true difference in means between group anac032-1 and group Col-0 is not equal to 0
95 percent confidence interval:
 -0.1610473 0.4149559
sample estimates:
mean in group anac032-1   mean in group Col-0
            -1.028577   -1.155531

$logLRP

Two Sample t-test

data: x by dataset_anac0321_1mM$Genotype
t = 0.87435, df = 36, p-value = 0.3877
alternative hypothesis: true difference in means between group anac032-1 and group Col-0 is not equal to 0
95 percent confidence interval:
 -0.1277836 0.3214602
sample estimates:
mean in group anac032-1   mean in group Col-0
            -1.348974   -1.445813

$logPR

Two Sample t-test

data: x by dataset_anac0321_10mM$Genotype
t = -2.8449, df = 31, p-value = 0.007802
alternative hypothesis: true difference in means between group anac032-1 and group Col-0 is not equal to 0
95 percent confidence interval:
 -0.18490163 -0.03048934
sample estimates:
mean in group anac032-1   mean in group Col-0
             1.285574   1.393269

$logLR

Two Sample t-test

```
data:  x by dataset_anac0321_10mM$Genotype
t = -1.1772, df = 31, p-value = 0.2481
alternative hypothesis: true difference in means between group anac032-1 and group Col-0 is
not equal to 0
95 percent confidence interval:
 -0.5491565  0.1472122
sample estimates:
mean in group anac032-1     mean in group Col-0
              0.6818206               0.8827927


$logLRL

	Two Sample t-test

data:  x by dataset_anac0321_10mM$Genotype
t = -1.8481, df = 31, p-value = 0.07415
alternative hypothesis: true difference in means between group anac032-1 and group Col-0 is
not equal to 0
95 percent confidence interval:
 -1.09057200  0.05370227
sample estimates:
mean in group anac032-1     mean in group Col-0
             -1.0349740              -0.5165391


$logALRL

	Two Sample t-test

data:  x by dataset_anac0321_10mM$Genotype
t = -1.4433, df = 31, p-value = 0.159
alternative hypothesis: true difference in means between group anac032-1 and group Col-0 is
not equal to 0
95 percent confidence interval:
 -0.7660784  0.1311530
sample estimates:
mean in group anac032-1     mean in group Col-0
              -1.716795               -1.399332


$logTRL
```

```
	Two Sample t-test

data:  x by dataset_anac0321_10mM$Genotype
t = -2.8313, df = 31, p-value = 0.00807
alternative hypothesis: true difference in means between group anac032-1 and group Col-0 is not equal to 0
95 percent confidence interval:
 -0.21653012 -0.03519891
sample estimates:
mean in group anac032-1     mean in group Col-0 
               1.420799                1.546664 


$logLRD

	Two Sample t-test

data:  x by dataset_anac0321_10mM$Genotype
t = -1.4607, df = 31, p-value = 0.1542
alternative hypothesis: true difference in means between group anac032-1 and group Col-0 is not equal to 0
95 percent confidence interval:
 -0.9842428  0.1627641
sample estimates:
mean in group anac032-1     mean in group Col-0 
              -2.320548               -1.909808 


$logLRP

	Two Sample t-test

data:  x by dataset_anac0321_10mM$Genotype
t = -1.5616, df = 31, p-value = 0.1285
alternative hypothesis: true difference in means between group anac032-1 and group Col-0 is not equal to 0
95 percent confidence interval:
 -0.9052967  0.1201560
sample estimates:
mean in group anac032-1     mean in group Col-0 
              -2.455773               -2.063203 
```

[1] "anac032-cc-s-1"
$logPR

Two Sample t-test

data: x by dataset_anac032ccs1_1mM$Genotype
t = 0.38095, df = 39, p-value = 0.7053
alternative hypothesis: true difference in means between group anac032-cc-s-1 and group Col-0 is not equal to 0
95 percent confidence interval:
 -0.04253148 0.06226983
sample estimates:
mean in group anac032-cc-s-1   mean in group Col-0
                    1.324432   1.314563

$logLR

Two Sample t-test

data: x by dataset_anac032ccs1_1mM$Genotype
t = -3.6585, df = 39, p-value = 0.0007487
alternative hypothesis: true difference in means between group anac032-cc-s-1 and group Col-0 is not equal to 0
95 percent confidence interval:
 -0.7583294 -0.2183516
sample estimates:
mean in group anac032-cc-s-1   mean in group Col-0
                   0.7480778   1.2364183

$logLRL

Two Sample t-test

data: x by dataset_anac032ccs1_1mM$Genotype
t = -3.4477, df = 39, p-value = 0.00137
alternative hypothesis: true difference in means between group anac032-cc-s-1 and group Col-0 is not equal to 0
95 percent confidence interval:
 -1.0275769 -0.2676717
sample estimates:
mean in group anac032-cc-s-1   mean in group Col-0
                  -0.3584359   0.2891883

```
$logALRL

	Two Sample t-test

data:  x by dataset_anac032ccs1_1mM$Genotype
t = -1.1288, df = 39, p-value = 0.2659
alternative hypothesis: true difference in means between group anac032-cc-s-1 and group Col-0
is not equal to 0
95 percent confidence interval:
 -0.4446970  0.1261294
sample estimates:
mean in group anac032-cc-s-1         mean in group Col-0
                -1.106514                  -0.947230


$logTRL

	Two Sample t-test

data:  x by dataset_anac032ccs1_1mM$Genotype
t = -2.6122, df = 39, p-value = 0.01271
alternative hypothesis: true difference in means between group anac032-cc-s-1 and group Col-0
is not equal to 0
95 percent confidence interval:
 -0.23210999 -0.02951977
sample estimates:
mean in group anac032-cc-s-1         mean in group Col-0
                 1.516343                   1.647158


$logLRD

	Two Sample t-test

data:  x by dataset_anac032ccs1_1mM$Genotype
t = -3.7004, df = 39, p-value = 0.0006631
alternative hypothesis: true difference in means between group anac032-cc-s-1 and group Col-0
is not equal to 0
95 percent confidence interval:
 -1.0168918 -0.2980951
sample estimates:
mean in group anac032-cc-s-1         mean in group Col-0
```

-1.682868 -1.025374

$logLRP

Two Sample t-test

data: x by dataset_anac032ccs1_1mM$Genotype
t = -3.6135, df = 39, p-value = 0.0008529
alternative hypothesis: true difference in means between group anac032-cc-s-1 and group Col-0 is not equal to 0
95 percent confidence interval:
-0.8061010 -0.2275178
sample estimates:
mean in group anac032-cc-s-1 mean in group Col-0
-1.874779 -1.357969

$logPR

Two Sample t-test

data: x by dataset_anac032ccs1_10mM$Genotype
t = -2.6932, df = 36, p-value = 0.01068
alternative hypothesis: true difference in means between group anac032-cc-s-1 and group Col-0 is not equal to 0
95 percent confidence interval:
-0.14891225 -0.02097682
sample estimates:
mean in group anac032-cc-s-1 mean in group Col-0
1.346393 1.431337

$logLR

Two Sample t-test

data: x by dataset_anac032ccs1_10mM$Genotype
t = -2.0124, df = 36, p-value = 0.0517
alternative hypothesis: true difference in means between group anac032-cc-s-1 and group Col-0 is not equal to 0
95 percent confidence interval:
-0.741937595 0.002876125
sample estimates:

```
mean in group anac032-cc-s-1         mean in group Col-0
                   0.6325825                   1.0021133


$logLRL

	Two Sample t-test

data:  x by dataset_anac032ccs1_10mM$Genotype
t = -1.8511, df = 36, p-value = 0.07237
alternative hypothesis: true difference in means between group anac032-cc-s-1 and group Col-0
is not equal to 0
95 percent confidence interval:
 -1.1077716  0.0505435
sample estimates:
mean in group anac032-cc-s-1         mean in group Col-0
                  -0.9795225                  -0.4509084


$logALRL

	Two Sample t-test

data:  x by dataset_anac032ccs1_10mM$Genotype
t = -0.84036, df = 36, p-value = 0.4063
alternative hypothesis: true difference in means between group anac032-cc-s-1 and group Col-0
is not equal to 0
95 percent confidence interval:
 -0.5430069  0.2248403
sample estimates:
mean in group anac032-cc-s-1         mean in group Col-0
                   -1.612105                   -1.453022


$logTRL

	Two Sample t-test

data:  x by dataset_anac032ccs1_10mM$Genotype
t = -2.7993, df = 36, p-value = 0.00818
alternative hypothesis: true difference in means between group anac032-cc-s-1 and group Col-0
is not equal to 0
95 percent confidence interval:
 -0.24548121 -0.03921632
```

sample estimates:
mean in group anac032-cc-s-1 mean in group Col-0
1.467237 1.609585

$logLRD

Two Sample t-test

data: x by dataset_anac032ccs1_10mM$Genotype
t = -1.6189, df = 36, p-value = 0.1142
alternative hypothesis: true difference in means between group anac032-cc-s-1 and group Col-0 is not equal to 0
95 percent confidence interval:
-0.9994937 0.1121547
sample estimates:
mean in group anac032-cc-s-1 mean in group Col-0
-2.325915 -1.882246

$logLRP

Two Sample t-test

data: x by dataset_anac032ccs1_10mM$Genotype
t = -1.5834, df = 36, p-value = 0.1221
alternative hypothesis: true difference in means between group anac032-cc-s-1 and group Col-0 is not equal to 0
95 percent confidence interval:
-0.8810091 0.1084786
sample estimates:
mean in group anac032-cc-s-1 mean in group Col-0
-2.446759 -2.060494

[1] "arf18-2"
$logPR

Two Sample t-test

data: x by dataset_arf182_1mM$Genotype
t = -2.5094, df = 35, p-value = 0.01687
alternative hypothesis: true difference in means between group arf18-2 and group Col-0 is not equal to 0

```
95 percent confidence interval:
 -0.21236721 -0.02242129
sample estimates:
mean in group arf18-2   mean in group Col-0
             1.236667              1.354062


$logLR

	Two Sample t-test

data:  x by dataset_arf182_1mM$Genotype
t = -1.6946, df = 35, p-value = 0.09903
alternative hypothesis: true difference in means between group arf18-2 and group Col-0 is not equal to 0
95 percent confidence interval:
 -0.63566534  0.05726017
sample estimates:
mean in group arf18-2   mean in group Col-0
            0.9271543             1.2163568


$logLRL

	Two Sample t-test

data:  x by dataset_arf182_1mM$Genotype
t = -1.3791, df = 35, p-value = 0.1766
alternative hypothesis: true difference in means between group arf18-2 and group Col-0 is not equal to 0
95 percent confidence interval:
 -0.7159524  0.1367134
sample estimates:
mean in group arf18-2   mean in group Col-0
            0.3164538             0.6060733


$logALRL

	Two Sample t-test

data:  x by dataset_arf182_1mM$Genotype
t = -0.0030519, df = 35, p-value = 0.9976
alternative hypothesis: true difference in means between group arf18-2 and group Col-0 is not
```

equal to 0
95 percent confidence interval:
 -0.2777559 0.2769221
sample estimates:
mean in group arf18-2   mean in group Col-0
          -0.6107005   -0.6102835

$logTRL

Two Sample t-test

data: x by dataset_arf182_1mM$Genotype
t = -1.8592, df = 35, p-value = 0.07143
alternative hypothesis: true difference in means between group arf18-2 and group Col-0 is not equal to 0
95 percent confidence interval:
 -0.28466941 0.01251203
sample estimates:
mean in group arf18-2   mean in group Col-0
            1.621235   1.757313

$logLRD

Two Sample t-test

data: x by dataset_arf182_1mM$Genotype
t = -0.86333, df = 35, p-value = 0.3938
alternative hypothesis: true difference in means between group arf18-2 and group Col-0 is not equal to 0
95 percent confidence interval:
 -0.5772088 0.2327582
sample estimates:
mean in group arf18-2   mean in group Col-0
          -0.9202135   -0.7479883

$logLRP

Two Sample t-test

data: x by dataset_arf182_1mM$Genotype
t = -1.0173, df = 35, p-value = 0.316

alternative hypothesis: true difference in means between group arf18-2 and group Col-0 is not equal to 0
95 percent confidence interval:
-0.4599348 0.1528532
sample estimates:
mean in group arf18-2 mean in group Col-0
-1.304781 -1.151240

$logPR

Two Sample t-test

data: x by dataset_arf182_10mM$Genotype
t = -1.9774, df = 36, p-value = 0.05569
alternative hypothesis: true difference in means between group arf18-2 and group Col-0 is not equal to 0
95 percent confidence interval:
-0.170998826 0.002162052
sample estimates:
mean in group arf18-2 mean in group Col-0
1.252731 1.337149

$logLR

Two Sample t-test

data: x by dataset_arf182_10mM$Genotype
t = -1.0199, df = 36, p-value = 0.3146
alternative hypothesis: true difference in means between group arf18-2 and group Col-0 is not equal to 0
95 percent confidence interval:
-0.4998818 0.1653498
sample estimates:
mean in group arf18-2 mean in group Col-0
0.5809599 0.7482259

$logLRL

Two Sample t-test

data: x by dataset_arf182_10mM$Genotype

t = -0.57636, df = 36, p-value = 0.568
alternative hypothesis: true difference in means between group arf18-2 and group Col-0 is not equal to 0
95 percent confidence interval:
 -0.5850152 0.3260900
sample estimates:
mean in group arf18-2   mean in group Col-0
          -0.5204753   -0.3910127

$logALRL

Two Sample t-test

data: x by dataset_arf182_10mM$Genotype
t = 0.28284, df = 36, p-value = 0.7789
alternative hypothesis: true difference in means between group arf18-2 and group Col-0 is not equal to 0
95 percent confidence interval:
 -0.2332669 0.3088736
sample estimates:
mean in group arf18-2   mean in group Col-0
           -1.101435   -1.139239

$logTRL

Two Sample t-test

data: x by dataset_arf182_10mM$Genotype
t = -1.7298, df = 36, p-value = 0.09223
alternative hypothesis: true difference in means between group arf18-2 and group Col-0 is not equal to 0
95 percent confidence interval:
 -0.22696116 0.01801652
sample estimates:
mean in group arf18-2   mean in group Col-0
            1.424491   1.528964

$logLRD

Two Sample t-test

data: x by dataset_arf182_10mM$Genotype
t = -0.22118, df = 36, p-value = 0.8262
alternative hypothesis: true difference in means between group arf18-2 and group Col-0 is not equal to 0
95 percent confidence interval:
 -0.4580752 0.3679867
sample estimates:
mean in group arf18-2   mean in group Col-0
          -1.773206   -1.728162

$logLRP

          Two Sample t-test

data: x by dataset_arf182_10mM$Genotype
t = -0.14147, df = 36, p-value = 0.8883
alternative hypothesis: true difference in means between group arf18-2 and group Col-0 is not equal to 0
95 percent confidence interval:
 -0.3832423 0.3332617
sample estimates:
mean in group arf18-2   mean in group Col-0
          -1.944967   -1.919976

[1] "arf18-3"
$logPR

          Two Sample t-test

data: x by dataset_arf183_1mM$Genotype
t = -2.5794, df = 34, p-value = 0.0144
alternative hypothesis: true difference in means between group arf18-3 and group Col-0 is not equal to 0
95 percent confidence interval:
 -0.14395360 -0.01707881
sample estimates:
mean in group arf18-3   mean in group Col-0
           1.309138   1.389654

$logLR

```
	Two Sample t-test

data:  x by dataset_arf183_1mM$Genotype
t = -3.916, df = 34, p-value = 0.0004117
alternative hypothesis: true difference in means between group arf18-3 and group Col-0 is not
equal to 0
95 percent confidence interval:
 -0.848039 -0.268569
sample estimates:
mean in group arf18-3   mean in group Col-0
            0.4045617             0.9628657


$logLRL

	Two Sample t-test

data:  x by dataset_arf183_1mM$Genotype
t = -3.8893, df = 34, p-value = 0.0004442
alternative hypothesis: true difference in means between group arf18-3 and group Col-0 is not
equal to 0
95 percent confidence interval:
 -1.2522816 -0.3927341
sample estimates:
mean in group arf18-3   mean in group Col-0
           -0.7165428             0.1059651


$logALRL

	Two Sample t-test

data:  x by dataset_arf183_1mM$Genotype
t = -1.6862, df = 34, p-value = 0.1009
alternative hypothesis: true difference in means between group arf18-3 and group Col-0 is not
equal to 0
95 percent confidence interval:
 -0.5826310  0.0542232
sample estimates:
mean in group arf18-3   mean in group Col-0
           -1.1211045            -0.8569006


$logTRL
```

Two Sample t-test

data: x by dataset_arf183_1mM$Genotype
t = -3.7202, df = 34, p-value = 0.0007163
alternative hypothesis: true difference in means between group arf18-3 and group Col-0 is not equal to 0
95 percent confidence interval:
 -0.30838354 -0.09049128
sample estimates:
mean in group arf18-3   mean in group Col-0
1.455710   1.655147

$logLRD

Two Sample t-test

data: x by dataset_arf183_1mM$Genotype
t = -3.6928, df = 34, p-value = 0.0007734
alternative hypothesis: true difference in means between group arf18-3 and group Col-0 is not equal to 0
95 percent confidence interval:
 -1.150324 -0.333659
sample estimates:
mean in group arf18-3   mean in group Col-0
-2.025680   -1.283689

$logLRP

Two Sample t-test

data: x by dataset_arf183_1mM$Genotype
t = -3.6971, df = 34, p-value = 0.0007643
alternative hypothesis: true difference in means between group arf18-3 and group Col-0 is not equal to 0
95 percent confidence interval:
 -0.9655634 -0.2805776
sample estimates:
mean in group arf18-3   mean in group Col-0
-2.172253   -1.549182

$logPR

Two Sample t-test

```
data:  x by dataset_arf183_10mM$Genotype
t = -2.6374, df = 27, p-value = 0.01369
alternative hypothesis: true difference in means between group arf18-3 and group Col-0 is not equal to 0
95 percent confidence interval:
 -0.25510414 -0.03185486
sample estimates:
mean in group arf18-3   mean in group Col-0
             1.296939              1.440418
```

$logLR

Two Sample t-test

```
data:  x by dataset_arf183_10mM$Genotype
t = -5.527, df = 27, p-value = 7.419e-06
alternative hypothesis: true difference in means between group arf18-3 and group Col-0 is not equal to 0
95 percent confidence interval:
 -1.2643030 -0.5797303
sample estimates:
mean in group arf18-3   mean in group Col-0
            0.2132761             1.1352927
```

$logLRL

Two Sample t-test

```
data:  x by dataset_arf183_10mM$Genotype
t = -4.3528, df = 27, p-value = 0.0001731
alternative hypothesis: true difference in means between group arf18-3 and group Col-0 is not equal to 0
95 percent confidence interval:
 -1.3662865 -0.4908565
sample estimates:
mean in group arf18-3   mean in group Col-0
           -0.7635761             0.1649954
```

$logALRL

Two Sample t-test

data: x by dataset_arf183_10mM$Genotype
t = -0.035108, df = 27, p-value = 0.9723
alternative hypothesis: true difference in means between group arf18-3 and group Col-0 is not equal to 0
95 percent confidence interval:
 -0.3896403 0.3765306
sample estimates:
mean in group arf18-3   mean in group Col-0
-0.9768521   -0.9702973

$logTRL

Two Sample t-test

data: x by dataset_arf183_10mM$Genotype
t = -4.4662, df = 27, p-value = 0.0001278
alternative hypothesis: true difference in means between group arf18-3 and group Col-0 is not equal to 0
95 percent confidence interval:
 -0.3953973 -0.1464592
sample estimates:
mean in group arf18-3   mean in group Col-0
1.436161   1.707089

$logLRD

Two Sample t-test

data: x by dataset_arf183_10mM$Genotype
t = -3.5963, df = 27, p-value = 0.001274
alternative hypothesis: true difference in means between group arf18-3 and group Col-0 is not equal to 0
95 percent confidence interval:
 -1.2330157 -0.3371684
sample estimates:
mean in group arf18-3   mean in group Col-0
-2.060515   -1.275423

$logLRP

Two Sample t-test

data: x by dataset_arf183_10mM$Genotype
t = -3.5431, df = 27, p-value = 0.001462
alternative hypothesis: true difference in means between group arf18-3 and group Col-0 is not equal to 0
95 percent confidence interval:
 -1.0384899 -0.2767966
sample estimates:
mean in group arf18-3   mean in group Col-0
          -2.199737   -1.542093

[1] "arf18-cc-1"
$logPR

Two Sample t-test

data: x by dataset_arf18cc1_1mM$Genotype
t = 1.9506, df = 27, p-value = 0.06155
alternative hypothesis: true difference in means between group arf18-cc-1 and group Col-0 is not equal to 0
95 percent confidence interval:
 -0.003501654 0.138480286
sample estimates:
mean in group arf18-cc-1   mean in group Col-0
              1.398978   1.331489

$logLR

Two Sample t-test

data: x by dataset_arf18cc1_1mM$Genotype
t = 2.803, df = 27, p-value = 0.009259
alternative hypothesis: true difference in means between group arf18-cc-1 and group Col-0 is not equal to 0
95 percent confidence interval:
 0.1088146 0.7032982
sample estimates:

mean in group arf18-cc-1   mean in group Col-0
0.9171171   0.5110607

$logLRL

Two Sample t-test

data: x by dataset_arf18cc1_1mM$Genotype
t = 0.52646, df = 27, p-value = 0.6029
alternative hypothesis: true difference in means between group arf18-cc-1 and group Col-0 is not equal to 0
95 percent confidence interval:
-0.3046678 0.5149699
sample estimates:
mean in group arf18-cc-1   mean in group Col-0
-0.170124   -0.275275

$logALRL

Two Sample t-test

data: x by dataset_arf18cc1_1mM$Genotype
t = -2.5738, df = 27, p-value = 0.01587
alternative hypothesis: true difference in means between group arf18-cc-1 and group Col-0 is not equal to 0
95 percent confidence interval:
-0.54078812 -0.06102247
sample estimates:
mean in group arf18-cc-1   mean in group Col-0
-1.0872411   -0.7863358

$logTRL

Two Sample t-test

data: x by dataset_arf18cc1_1mM$Genotype
t = 1.422, df = 27, p-value = 0.1665
alternative hypothesis: true difference in means between group arf18-cc-1 and group Col-0 is not equal to 0
95 percent confidence interval:
-0.02966532 0.16361131

sample estimates:
mean in group arf18-cc-1   mean in group Col-0
1.603999   1.537026

$logLRD

Two Sample t-test

data: x by dataset_arf18cc1_1mM$Genotype
t = 0.18704, df = 27, p-value = 0.853
alternative hypothesis: true difference in means between group arf18-cc-1 and group Col-0 is not equal to 0
95 percent confidence interval:
-0.3754968 0.4508203
sample estimates:
mean in group arf18-cc-1   mean in group Col-0
-1.569102   -1.606764

$logLRP

Two Sample t-test

data: x by dataset_arf18cc1_1mM$Genotype
t = 0.22933, df = 27, p-value = 0.8203
alternative hypothesis: true difference in means between group arf18-cc-1 and group Col-0 is not equal to 0
95 percent confidence interval:
-0.3034047 0.3797609
sample estimates:
mean in group arf18-cc-1   mean in group Col-0
-1.774123   -1.812301

$logPR

Two Sample t-test

data: x by dataset_arf18cc1_10mM$Genotype
t = -0.9226, df = 12, p-value = 0.3744
alternative hypothesis: true difference in means between group arf18-cc-1 and group Col-0 is not equal to 0
95 percent confidence interval:

-0.13930418 0.05642454
sample estimates:
mean in group arf18-cc-1   mean in group Col-0
1.318437   1.359877

$logLR

Two Sample t-test

data: x by dataset_arf18cc1_10mM$Genotype
t = 1.6852, df = 12, p-value = 0.1178
alternative hypothesis: true difference in means between group arf18-cc-1 and group Col-0 is not equal to 0
95 percent confidence interval:
-0.1539401 1.2049844
sample estimates:
mean in group arf18-cc-1   mean in group Col-0
0.5255221   0.0000000

$logLRL

Two Sample t-test

data: x by dataset_arf18cc1_10mM$Genotype
t = 0.52214, df = 12, p-value = 0.6111
alternative hypothesis: true difference in means between group arf18-cc-1 and group Col-0 is not equal to 0
95 percent confidence interval:
-1.016252 1.656844
sample estimates:
mean in group arf18-cc-1   mean in group Col-0
-1.119129   -1.439425

$logALRL

Two Sample t-test

data: x by dataset_arf18cc1_10mM$Genotype
t = -0.50311, df = 12, p-value = 0.624
alternative hypothesis: true difference in means between group arf18-cc-1 and group Col-0 is not equal to 0

95 percent confidence interval:
 -1.0939946 0.6835428
sample estimates:
mean in group arf18-cc-1   mean in group Col-0
              -1.644651   -1.439425

$logTRL

Two Sample t-test

data: x by dataset_arf18cc1_10mM$Genotype
t = 0.22598, df = 12, p-value = 0.825
alternative hypothesis: true difference in means between group arf18-cc-1 and group Col-0 is not equal to 0
95 percent confidence interval:
 -0.1494229 0.1840043
sample estimates:
mean in group arf18-cc-1   mean in group Col-0
               1.439899   1.422608

$logLRD

Two Sample t-test

data: x by dataset_arf18cc1_10mM$Genotype
t = 0.58354, df = 12, p-value = 0.5703
alternative hypothesis: true difference in means between group arf18-cc-1 and group Col-0 is not equal to 0
95 percent confidence interval:
 -0.9889163 1.7123883
sample estimates:
mean in group arf18-cc-1   mean in group Col-0
              -2.437566   -2.799302

$logLRP

Two Sample t-test

data: x by dataset_arf18cc1_10mM$Genotype
t = 0.53984, df = 12, p-value = 0.5992
alternative hypothesis: true difference in means between group arf18-cc-1 and group Col-0 is

```
not equal to 0
95 percent confidence interval:
 -0.9199339  1.5259449
sample estimates:
mean in group arf18-cc-1      mean in group Col-0
              -2.559028                -2.862033



[1] "nlp7-1"
$logPR

	Two Sample t-test

data:  x by dataset_nlp71_1mM$Genotype
t = 0.68971, df = 54, p-value = 0.4933
alternative hypothesis: true difference in means between group Col-0 and group nlp7-1 is not equal to 0
95 percent confidence interval:
 -0.03670224  0.07519748
sample estimates:
 mean in group Col-0 mean in group nlp7-1
           1.462431             1.443183


$logLR

	Two Sample t-test

data:  x by dataset_nlp71_1mM$Genotype
t = 0.77956, df = 54, p-value = 0.4391
alternative hypothesis: true difference in means between group Col-0 and group nlp7-1 is not equal to 0
95 percent confidence interval:
 -0.1138927  0.2588129
sample estimates:
 mean in group Col-0 mean in group nlp7-1
           1.431742             1.359282


$logLRL

	Two Sample t-test
```

data: x by dataset_nlp71_1mM$Genotype
t = -4.5546, df = 54, p-value = 3.032e-05
alternative hypothesis: true difference in means between group Col-0 and group nlp7-1 is not equal to 0
95 percent confidence interval:
 -0.4072426 -0.1582991
sample estimates:
 mean in group Col-0 mean in group nlp7-1
          -0.6100701   -0.3272993

$logALRL

Two Sample t-test

data: x by dataset_nlp71_1mM$Genotype
t = -3.0911, df = 54, p-value = 0.003152
alternative hypothesis: true difference in means between group Col-0 and group nlp7-1 is not equal to 0
95 percent confidence interval:
 -0.5856357 -0.1248262
sample estimates:
 mean in group Col-0 mean in group nlp7-1
           -2.041812   -1.686581

$logTRL

Two Sample t-test

data: x by dataset_nlp71_1mM$Genotype
t = -3.8135, df = 54, p-value = 0.0003539
alternative hypothesis: true difference in means between group Col-0 and group nlp7-1 is not equal to 0
95 percent confidence interval:
 -0.4509091 -0.1401640
sample estimates:
 mean in group Col-0 mean in group nlp7-1
            1.811622   2.107158

$logLRD

Two Sample t-test

data: x by dataset_nlp71_1mM$Genotype
t = 0.64354, df = 54, p-value = 0.5226
alternative hypothesis: true difference in means between group Col-0 and group nlp7-1 is not equal to 0
95 percent confidence interval:
 -0.1125659 0.2189909
sample estimates:
 mean in group Col-0 mean in group nlp7-1
       -0.03068906   -0.08390156

$logLRP

          Two Sample t-test

data: x by dataset_nlp71_1mM$Genotype
t = 0.13624, df = 54, p-value = 0.8921
alternative hypothesis: true difference in means between group Col-0 and group nlp7-1 is not equal to 0
95 percent confidence interval:
 -0.1750950 0.2006264
sample estimates:
 mean in group Col-0 mean in group nlp7-1
         -2.421692   -2.434457

$logPR

          Two Sample t-test

data: x by dataset_nlp71_10mM$Genotype
t = -2.2297, df = 57, p-value = 0.02972
alternative hypothesis: true difference in means between group Col-0 and group nlp7-1 is not equal to 0
95 percent confidence interval:
 -0.31753795 -0.01704951
sample estimates:
 mean in group Col-0 mean in group nlp7-1
           1.101995   1.269289

$logLR

```
        Two Sample t-test

data:  x by dataset_nlp71_10mM$Genotype
t = -0.2521, df = 57, p-value = 0.8019
alternative hypothesis: true difference in means between group Col-0 and group nlp7-1 is not
equal to 0
95 percent confidence interval:
 -0.2577887  0.2001386
sample estimates:
 mean in group Col-0 mean in group nlp7-1
           1.218915            1.247740


$logLRL

        Two Sample t-test

data:  x by dataset_nlp71_10mM$Genotype
t = -4.1233, df = 57, p-value = 0.0001226
alternative hypothesis: true difference in means between group Col-0 and group nlp7-1 is not
equal to 0
95 percent confidence interval:
 -0.6679732 -0.2312622
sample estimates:
 mean in group Col-0 mean in group nlp7-1
         -0.9663224           -0.5167047


$logALRL

        Two Sample t-test

data:  x by dataset_nlp71_10mM$Genotype
t = -2.5043, df = 57, p-value = 0.01515
alternative hypothesis: true difference in means between group Col-0 and group nlp7-1 is not
equal to 0
95 percent confidence interval:
 -0.75725774 -0.08432756
sample estimates:
 mean in group Col-0 mean in group nlp7-1
          -2.185237            -1.764445


$logTRL
```

```
	Two Sample t-test

data:  x by dataset_nlp71_10mM$Genotype
t = -0.87627, df = 57, p-value = 0.3846
alternative hypothesis: true difference in means between group Col-0 and group nlp7-1 is not equal to 0
95 percent confidence interval:
 -0.17205802  0.06731077
sample estimates:
 mean in group Col-0 mean in group nlp7-1 
           1.915368            1.967742 


$logLRD

	Two Sample t-test

data:  x by dataset_nlp71_10mM$Genotype
t = 0.93213, df = 57, p-value = 0.3552
alternative hypothesis: true difference in means between group Col-0 and group nlp7-1 is not equal to 0
95 percent confidence interval:
 -0.1589981  0.4359354
sample estimates:
 mean in group Col-0 mean in group nlp7-1 
         0.11691964         -0.02154901 


$logLRP

	Two Sample t-test

data:  x by dataset_nlp71_10mM$Genotype
t = -3.3399, df = 57, p-value = 0.001482
alternative hypothesis: true difference in means between group Col-0 and group nlp7-1 is not equal to 0
95 percent confidence interval:
 -0.6354130 -0.1590752
sample estimates:
 mean in group Col-0 mean in group nlp7-1 
          -2.881690           -2.484446 
```

```
#################################################
#                                               #
#Two-way ANOVA for Genotype:Treatment Interaction#
#                                               #
#################################################
[1] "anac032-1"
logPR ~ Genotype * Treatment + Image
<environment: 0x0000029574b8e9c0>
Analysis of Variance Table

Response: logPR
                    Df Sum Sq  Mean Sq F value  Pr(>F)
Genotype             1 0.0388 0.038809  2.8675 0.09111 .
Treatment            1 0.0308 0.030751  2.2721 0.13246
Image               73 7.1824 0.098390  7.2699 < 2e-16 ***
Genotype:Treatment   1 0.0399 0.039897  2.9479 0.08671 .
Residuals          428 5.7925 0.013534
---
Signif. codes:  0 ‘***’ 0.001 ‘**’ 0.01 ‘*’ 0.05 ‘.’ 0.1 ‘ ’ 1
logLR ~ Genotype * Treatment + Image
<environment: 0x0000029574b8e9c0>
Analysis of Variance Table

Response: logLR
                    Df Sum Sq Mean Sq F value    Pr(>F)
Genotype             1  0.012  0.0119  0.0572    0.8110
Treatment            1  3.661  3.6608 17.5455 3.408e-05 ***
Image               73 71.656  0.9816  4.7045 < 2.2e-16 ***
Genotype:Treatment   1  0.170  0.1699  0.8144    0.3673
Residuals          428 89.301  0.2086
---
Signif. codes:  0 ‘***’ 0.001 ‘**’ 0.01 ‘*’ 0.05 ‘.’ 0.1 ‘ ’ 1
logLRL ~ Genotype * Treatment + Image
<environment: 0x0000029574b8e9c0>
Analysis of Variance Table

Response: logLRL
                    Df  Sum Sq Mean Sq  F value    Pr(>F)
Genotype             1   0.819   0.819   2.2659   0.13299
Treatment            1  42.956  42.956 118.7953 < 2.2e-16 ***
```

```
Image              73  92.439   1.266   3.5019  4.31e-16 ***
Genotype:Treatment   1   1.216   1.216   3.3634   0.06735 .
Residuals         428 154.764   0.362
---
Signif. codes:  0 ‘***’ 0.001 ‘**’ 0.01 ‘*’ 0.05 ‘.’ 0.1 ‘ ’ 1
logALRL ~ Genotype * Treatment + Image
<environment: 0x0000029574b8e9c0>
Analysis of Variance Table

Response: logALRL
                 Df  Sum Sq Mean Sq F value  Pr(>F)
Genotype           1   1.029  1.0291  3.9198 0.04836 *
Treatment          1  21.537 21.5367 82.0305 < 2e-16 ***
Image             73 110.124  1.5086  5.7459 < 2e-16 ***
Genotype:Treatment   1   0.477  0.4769  1.8166 0.17843
Residuals         428 112.369  0.2625
---
Signif. codes:  0 ‘***’ 0.001 ‘**’ 0.01 ‘*’ 0.05 ‘.’ 0.1 ‘ ’ 1
logTRL ~ Genotype * Treatment + Image
<environment: 0x0000029574b8e9c0>
Analysis of Variance Table

Response: logTRL
                 Df  Sum Sq Mean Sq F value    Pr(>F)
Genotype           1  0.2690 0.26899  9.5246 0.0021591 **
Treatment          1  0.4148 0.41482 14.6882 0.0001459 ***
Image             73 12.8346 0.17582  6.2254 < 2.2e-16 ***
Genotype:Treatment   1  0.0733 0.07327  2.5944 0.1079808
Residuals         428 12.0874 0.02824
---
Signif. codes:  0 ‘***’ 0.001 ‘**’ 0.01 ‘*’ 0.05 ‘.’ 0.1 ‘ ’ 1
logLRD ~ Genotype * Treatment + Image
<environment: 0x0000029574b8e9c0>
Analysis of Variance Table

Response: logLRD
                 Df  Sum Sq Mean Sq F value    Pr(>F)
Genotype           1   4.420  4.4205 13.0162 0.0003453 ***
Treatment          1  30.433 30.4331 89.6114 < 2.2e-16 ***
Image             73 184.262  2.5241  7.4324 < 2.2e-16 ***
Genotype:Treatment   1   0.816  0.8155  2.4014 0.1219643
Residuals         428 145.354  0.3396
---
Signif. codes:  0 ‘***’ 0.001 ‘**’ 0.01 ‘*’ 0.05 ‘.’ 0.1 ‘ ’ 1
```

logLRP ~ Genotype * Treatment + Image
<environment: 0x0000029574b8e9c0>
Analysis of Variance Table

Response: logLRP

	Df	Sum Sq	Mean Sq	F value	Pr(>F)	
Genotype	1	0.149	0.149	0.6068	0.43641	
Treatment	1	34.928	34.928	141.8710	< 2e-16	***
Image	73	86.914	1.191	4.8360	< 2e-16	***
Genotype:Treatment	1	0.692	0.692	2.8126	0.09426	.
Residuals	428	105.373	0.246			

-

Signif. codes: 0 ‘***’ 0.001 ‘**’ 0.01 ‘*’ 0.05 ‘.’ 0.1 ‘ ’ 1
[1] "arf18-cc-1"
logPR ~ Genotype * Treatment + Image
<environment: 0x00000295738c0158>
Analysis of Variance Table

Response: logPR

	Df	Sum Sq	Mean Sq	F value	Pr(>F)	
Genotype	1	0.0142	0.014198	1.0996	0.29495	
Treatment	1	0.0379	0.037909	2.9359	0.08736	.
Image	74	7.2179	0.097539	7.5540	< 2e-16	***
Genotype:Treatment	1	0.0150	0.014971	1.1594	0.28220	
Residuals	421	5.4360	0.012912			

Signif. codes: 0 ‘***’ 0.001 ‘**’ 0.01 ‘*’ 0.05 ‘.’ 0.1 ‘ ’ 1
logLR ~ Genotype * Treatment + Image
<environment: 0x00000295738c0158>
Analysis of Variance Table

Response: logLR

	Df	Sum Sq	Mean Sq	F value	Pr(>F)	
Genotype	1	0.246	0.2463	1.1921	0.2755	
Treatment	1	3.589	3.5891	17.3719	3.732e-05	***
Image	74	71.404	0.9649	4.6703	< 2.2e-16	***
Genotype:Treatment	1	0.106	0.1063	0.5143	0.4737	
Residuals	421	86.981	0.2066			

Signif. codes: 0 ‘***’ 0.001 ‘**’ 0.01 ‘*’ 0.05 ‘.’ 0.1 ‘ ’ 1
logLRL ~ Genotype * Treatment + Image
<environment: 0x00000295738c0158>
Analysis of Variance Table

```
Response: logLRL
                   Df  Sum Sq Mean Sq  F value    Pr(>F)    
Genotype            1   4.314   4.314  12.1396 0.0005454 ***
Treatment           1  38.124  38.124 107.2689 < 2.2e-16 ***
Image              74  90.743   1.226   3.4503 8.753e-16 ***
Genotype:Treatment   1   0.435   0.435   1.2226 0.2694803    
Residuals         421 149.626   0.355                       
---
Signif. codes:  0 ‘***’ 0.001 ‘**’ 0.01 ‘*’ 0.05 ‘.’ 0.1 ‘ ’ 1
logALRL ~ Genotype * Treatment + Image
<environment: 0x00000295738c0158>
Analysis of Variance Table

Response: logALRL
                   Df  Sum Sq Mean Sq F value    Pr(>F)    
Genotype            1   2.499  2.4991  9.5707  0.002109 ** 
Treatment           1  18.318 18.3180 70.1518 8.254e-16 ***
Image              74 108.061  1.4603  5.5924 < 2.2e-16 ***
Genotype:Treatment   1   0.111  0.1110  0.4252  0.514692    
Residuals         421 109.932  0.2611                      
---
Signif. codes:  0 ‘***’ 0.001 ‘**’ 0.01 ‘*’ 0.05 ‘.’ 0.1 ‘ ’ 1
logTRL ~ Genotype * Treatment + Image
<environment: 0x00000295738c0158>
Analysis of Variance Table

Response: logTRL
                   Df  Sum Sq Mean Sq F value    Pr(>F)    
Genotype            1  0.2533 0.25328  9.2080 0.0025589 ** 
Treatment           1  0.3355 0.33551 12.1976 0.0005292 ***
Image              74 12.8492 0.17364  6.3126 < 2.2e-16 ***
Genotype:Treatment   1  0.0002 0.00018  0.0067 0.9350032    
Residuals         421 11.5803 0.02751                      
---
Signif. codes:  0 ‘***’ 0.001 ‘**’ 0.01 ‘*’ 0.05 ‘.’ 0.1 ‘ ’ 1
logLRD ~ Genotype * Treatment + Image
<environment: 0x00000295738c0158>
Analysis of Variance Table

Response: logLRD
                   Df  Sum Sq Mean Sq F value    Pr(>F)    
Genotype            1  12.007 12.0066 35.7672 4.752e-09 ***
Treatment           1  26.131 26.1310 77.8429 < 2.2e-16 ***
```

```
Image              74 180.758  2.4427  7.2766 < 2.2e-16 ***
Genotype:Treatment   1   0.611  0.6108  1.8195    0.1781
Residuals          421 141.325  0.3357
---
Signif. codes:  0 '***' 0.001 '**' 0.01 '*' 0.05 '.' 0.1 ' ' 1
logLRP ~ Genotype * Treatment + Image
<environment: 0x00000295738c0158>
Analysis of Variance Table

Response: logLRP
                    Df  Sum Sq Mean Sq  F value    Pr(>F)
Genotype             1   2.477  2.4770  10.1759  0.001529 **
Treatment            1  31.306 31.3065 128.6103 < 2.2e-16 ***
Image               74  85.442  1.1546   4.7433 < 2.2e-16 ***
Genotype:Treatment   1   0.453  0.4525   1.8591  0.173456
Residuals          421 102.480  0.2434

---
Signif. codes:  0 '***' 0.001 '**' 0.01 '*' 0.05 '.' 0.1 ' ' 1
[1] "nlp7-1"
logPR ~ Genotype * Treatment + Image
<environment: 0x00000295720d08c8>
Analysis of Variance Table

Response: logPR
                    Df Sum Sq  Mean Sq F value    Pr(>F)
Genotype             1 0.0078 0.007775  0.5453  0.460623
Treatment            1 0.1341 0.134104  9.4052  0.002293 **
Image               73 7.5808 0.103846  7.2831 < 2.2e-16 ***
Genotype:Treatment   1 0.1943 0.194302 13.6271  0.000250 ***
Residuals          454 6.4733 0.014258
---
Signif. codes:  0 '***' 0.001 '**' 0.01 '*' 0.05 '.' 0.1 ' ' 1
logLR ~ Genotype * Treatment + Image
<environment: 0x00000295720d08c8>
Analysis of Variance Table

Response: logLR
                    Df Sum Sq Mean Sq F value    Pr(>F)
Genotype             1 10.469 10.4688 52.6463 1.740e-12 ***
Treatment            1  3.081  3.0810 15.4940 9.575e-05 ***
Image               73 69.890  0.9574  4.8147 < 2.2e-16 ***
Genotype:Treatment   1  0.101  0.1008  0.5068    0.4769
Residuals          454 90.278  0.1989
```

```
---
Signif. codes:  0 ‘***’ 0.001 ‘**’ 0.01 ‘*’ 0.05 ‘.’ 0.1 ‘ ’ 1
logLRL ~ Genotype * Treatment + Image
<environment: 0x00000295720d08c8>
Analysis of Variance Table

Response: logLRL
                    Df  Sum Sq Mean Sq  F value    Pr(>F)
Genotype             1   4.049   4.049  12.7361 0.0003969 ***
Treatment            1  32.295  32.295 101.5747 < 2.2e-16 ***
Image               73  87.957   1.205   3.7896 < 2.2e-16 ***
Genotype:Treatment   1   0.189   0.189   0.5939 0.4413283
Residuals          454 144.346   0.318
---
Signif. codes:  0 ‘***’ 0.001 ‘**’ 0.01 ‘*’ 0.05 ‘.’ 0.1 ‘ ’ 1
logALRL ~ Genotype * Treatment + Image
<environment: 0x00000295720d08c8>
Analysis of Variance Table

Response: logALRL
                    Df  Sum Sq Mean Sq  F value    Pr(>F)
Genotype             1  27.540 27.5399 105.8896 < 2.2e-16 ***
Treatment            1  15.426 15.4260  59.3121 8.509e-14 ***
Image               73 105.879  1.4504   5.5767 < 2.2e-16 ***
Genotype:Treatment   1   0.014  0.0137   0.0527    0.8185
Residuals          454 118.077  0.2601
---
Signif. codes:  0 ‘***’ 0.001 ‘**’ 0.01 ‘*’ 0.05 ‘.’ 0.1 ‘ ’ 1
logTRL ~ Genotype * Treatment + Image
<environment: 0x00000295720d08c8>
Analysis of Variance Table

Response: logTRL
                    Df  Sum Sq Mean Sq  F value    Pr(>F)
Genotype             1  8.3718  8.3718 259.2125 < 2.2e-16 ***
Treatment            1  0.4183  0.4183  12.9523 0.0003547 ***
Image               73 11.0748  0.1517   4.6974 < 2.2e-16 ***
Genotype:Treatment   1  0.4751  0.4751  14.7117 0.0001430 ***
Residuals          454 14.6628  0.0323
---
Signif. codes:  0 ‘***’ 0.001 ‘**’ 0.01 ‘*’ 0.05 ‘.’ 0.1 ‘ ’ 1
logLRD ~ Genotype * Treatment + Image
<environment: 0x00000295720d08c8>
Analysis of Variance Table
```

```
Response: logLRD
                  Df  Sum Sq Mean Sq  F value    Pr(>F)
Genotype           1  73.652  73.652 242.2278 < 2.2e-16 ***
Treatment          1  19.152  19.152  62.9864 1.643e-14 ***
Image             73 179.294   2.456   8.0776 < 2.2e-16 ***
Genotype:Treatment   1   0.015   0.015   0.0500    0.8231
Residuals        454 138.043   0.304
---
Signif. codes:  0 '***' 0.001 '**' 0.01 '*' 0.05 '.' 0.1 ' ' 1
logLRP ~ Genotype * Treatment + Image
<environment: 0x00000295720d08c8>
Analysis of Variance Table

Response: logLRP
                  Df  Sum Sq Mean Sq  F value  Pr(>F)
Genotype           1  24.066 24.0659 106.8679 < 2e-16 ***
Treatment          1  25.362 25.3622 112.6245 < 2e-16 ***
Image             73  82.584  1.1313   5.0236 < 2e-16 ***
Genotype:Treatment   1   1.263  1.2630   5.6086 0.01829 *
Residuals        454 102.237  0.2252
---
Signif. codes:  0 '***' 0.001 '**' 0.01 '*' 0.05 '.' 0.1 ' ' 1
[1] "anac032-1/arf18-2"
logPR ~ Genotype * Treatment + Image
<environment: 0x0000029574f21aa8>
Analysis of Variance Table

Response: logPR
                  Df Sum Sq  Mean Sq F value    Pr(>F)
Genotype           1 0.1567 0.156685 10.3209  0.001409 **
Treatment          1 0.0037 0.003694  0.2433  0.622066
Image             74 7.4037 0.100050  6.5903 < 2.2e-16 ***
Genotype:Treatment   1 0.0099 0.009891  0.6515  0.419986
Residuals        454 6.8923 0.015181
---
Signif. codes:  0 '***' 0.001 '**' 0.01 '*' 0.05 '.' 0.1 ' ' 1
logLR ~ Genotype * Treatment + Image
<environment: 0x0000029574f21aa8>
Analysis of Variance Table

Response: logLR
                  Df Sum Sq Mean Sq F value    Pr(>F)
Genotype           1  6.853  6.8531 31.8630 2.919e-08 ***
```

```
Treatment           1  3.478  3.4781 16.1711 6.776e-05 ***
Image              74 70.577  0.9537  4.4344 < 2.2e-16 ***
Genotype:Treatment   1  0.219  0.2191  1.0186    0.3134
Residuals          454 97.646  0.2151
---
Signif. codes:  0 ‘***’ 0.001 ‘**’ 0.01 ‘*’ 0.05 ‘.’ 0.1 ‘ ’ 1
logLRL ~ Genotype * Treatment + Image
<environment: 0x0000029574f21aa8>
Analysis of Variance Table

Response: logLRL
                    Df  Sum Sq Mean Sq  F value    Pr(>F)
Genotype             1   1.075   1.075   2.8750   0.09065 .
Treatment            1  40.376  40.376 107.9416 < 2.2e-16 ***
Image               74  84.382   1.140   3.0485 4.082e-13 ***
Genotype:Treatment   1   0.127   0.127   0.3390   0.56071
Residuals          454 169.821   0.374
---
Signif. codes:  0 ‘***’ 0.001 ‘**’ 0.01 ‘*’ 0.05 ‘.’ 0.1 ‘ ’ 1
logALRL ~ Genotype * Treatment + Image
<environment: 0x0000029574f21aa8>
Analysis of Variance Table

Response: logALRL
                    Df  Sum Sq Mean Sq F value    Pr(>F)
Genotype             1   2.499  2.4990  9.0190  0.002819 **
Treatment            1  20.153 20.1534 72.7343 2.227e-16 ***
Image               74 107.295  1.4499  5.2328 < 2.2e-16 ***
Genotype:Treatment   1   0.679  0.6792  2.4513  0.118123
Residuals          454 125.796  0.2771
---
Signif. codes:  0 ‘***’ 0.001 ‘**’ 0.01 ‘*’ 0.05 ‘.’ 0.1 ‘ ’ 1
logTRL ~ Genotype * Treatment + Image
<environment: 0x0000029574f21aa8>
Analysis of Variance Table

Response: logTRL
                    Df  Sum Sq Mean Sq F value    Pr(>F)
Genotype             1  0.7958 0.79580 27.1752 2.828e-07 ***
Treatment            1  0.2956 0.29560 10.0943  0.001589 **
Image               74 12.6999 0.17162  5.8606 < 2.2e-16 ***
Genotype:Treatment   1  0.0008 0.00077  0.0261  0.871630
Residuals          454 13.2949 0.02928
---
```

```
Signif. codes:  0 ‘***’ 0.001 ‘**’ 0.01 ‘*’ 0.05 ‘.’ 0.1 ‘ ’ 1
logLRD ~ Genotype * Treatment + Image
<environment: 0x0000029574f21aa8>
Analysis of Variance Table

Response: logLRD
                   Df  Sum Sq Mean Sq F value    Pr(>F)    
Genotype            1   6.115  6.1155 17.2887  3.84e-05 ***
Treatment           1  29.730 29.7304 84.0496 < 2.2e-16 ***
Image              74 175.757  2.3751  6.7145 < 2.2e-16 ***
Genotype:Treatment   1   0.208  0.2075  0.5867    0.4441    
Residuals         454 160.591  0.3537                      
---
Signif. codes:  0 ‘***’ 0.001 ‘**’ 0.01 ‘*’ 0.05 ‘.’ 0.1 ‘ ’ 1
logLRP ~ Genotype * Treatment + Image
<environment: 0x0000029574f21aa8>
Analysis of Variance Table

Response: logLRP
                   Df  Sum Sq Mean Sq  F value Pr(>F)    
Genotype            1   0.021   0.021   0.0821 0.7746    
Treatment           1  33.762  33.762 131.9704 <2e-16 ***
Image              74  81.157   1.097   4.2869 <2e-16 ***
Genotype:Treatment   1   0.108   0.108   0.4216 0.5165    
Residuals         454 116.148   0.256                    
---

##########################
#                        #
#One-way ANOVA for Col-0#
#                        #
##########################
logPR ~ Treatment
<environment: 0x000001f047039840>
Analysis of Variance Table

Response: logPR
           Df Sum Sq  Mean Sq F value Pr(>F)
Treatment   1  0.023 0.022956  0.8627 0.3535
Residuals 470 12.506 0.026609               
logLR ~ Treatment
<environment: 0x000001f047039840>
Analysis of Variance Table
```

```
Response: logLR
          Df  Sum Sq Mean Sq F value   Pr(>F)
Treatment   1   2.926 2.92600  8.9326 0.002948 **
Residuals 470 153.956 0.32757
---
Signif. codes:  0 ‘***’ 0.001 ‘**’ 0.01 ‘*’ 0.05 ‘.’ 0.1 ‘ ’ 1
logLRL ~ Treatment
<environment: 0x000001f047039840>
Analysis of Variance Table

Response: logLRL
          Df  Sum Sq Mean Sq F value    Pr(>F)
Treatment   1  33.318  33.318  69.275 9.393e-16 ***
Residuals 470 226.049   0.481
---
Signif. codes:  0 ‘***’ 0.001 ‘**’ 0.01 ‘*’ 0.05 ‘.’ 0.1 ‘ ’ 1
logALRL ~ Treatment
<environment: 0x000001f047039840>
Analysis of Variance Table

Response: logALRL
          Df  Sum Sq Mean Sq F value    Pr(>F)
Treatment   1  16.497 16.4969  36.641 2.907e-09 ***
Residuals 470 211.612  0.4502
---
Signif. codes:  0 ‘***’ 0.001 ‘**’ 0.01 ‘*’ 0.05 ‘.’ 0.1 ‘ ’ 1
logTRL ~ Treatment
<environment: 0x000001f047039840>
Analysis of Variance Table

Response: logTRL
          Df  Sum Sq  Mean Sq F value  Pr(>F)
Treatment   1  0.2463 0.246267  4.8114 0.02876 *
Residuals 470 24.0567 0.051184
---
Signif. codes:  0 ‘***’ 0.001 ‘**’ 0.01 ‘*’ 0.05 ‘.’ 0.1 ‘ ’ 1
logLRD ~ Treatment
<environment: 0x000001f047039840>
Analysis of Variance Table

Response: logLRD
          Df  Sum Sq Mean Sq F value    Pr(>F)
Treatment   1  22.363  22.363  34.192 9.355e-09 ***
Residuals 470 307.392   0.654
```

```
---
Signif. codes:  0 ‘***’ 0.001 ‘**’ 0.01 ‘*’ 0.05 ‘.’ 0.1 ‘ ’ 1
logLRP ~ Treatment
<environment: 0x000001f047039840>
Analysis of Variance Table

Response: logLRP
           Df  Sum Sq Mean Sq F value    Pr(>F)
Treatment   1  27.836  27.836  74.036 < 2.2e-16 ***
Residuals 470 176.707   0.376
---
Signif. codes:  0 ‘***’ 0.001 ‘**’ 0.01 ‘*’ 0.05 ‘.’ 0.1 ‘ ’ 1

**************************************************************

Two-Way ANOVA
---
Signif. codes:  0 ‘***’ 0.001 ‘**’ 0.01 ‘*’ 0.05 ‘.’ 0.1 ‘ ’ 1
Signif. codes:  0 ‘***’ 0.001 ‘**’ 0.01 ‘*’ 0.05 ‘.’ 0.1 ‘ ’ 1
[1] "anac032-cc-s-1"
logPR ~ Genotype * Treatment + Image
<environment: 0x000002957087c9e0>
Analysis of Variance Table

Response: logPR
                    Df Sum Sq  Mean Sq F value  Pr(>F)
Genotype             1 0.0020 0.002002  0.1559 0.69316
Treatment            1 0.0160 0.016018  1.2470 0.26474
Image               73 7.1620 0.098109  7.6379 < 2e-16 ***
Genotype:Treatment   1 0.0380 0.038047  2.9620 0.08596 .
Residuals          434 5.5747 0.012845
---
Signif. codes:  0 ‘***’ 0.001 ‘**’ 0.01 ‘*’ 0.05 ‘.’ 0.1 ‘ ’ 1
logLR ~ Genotype * Treatment + Image
<environment: 0x000002957087c9e0>
Analysis of Variance Table

Response: logLR
                    Df Sum Sq Mean Sq F value    Pr(>F)
Genotype             1  0.969 0.96853  4.4933 0.0345946 *
Treatment            1  3.040 3.03960 14.1016 0.0001968 ***
Image               73 70.113 0.96045  4.4558 < 2.2e-16 ***
Genotype:Treatment   1  0.131 0.13128  0.6090 0.4355770
Residuals          434 93.549 0.21555
```

```
---
Signif. codes:  0 ‘***’ 0.001 ‘**’ 0.01 ‘*’ 0.05 ‘.’ 0.1 ‘ ’ 1
logLRL ~ Genotype * Treatment + Image
<environment: 0x000002957087c9e0>
Analysis of Variance Table

Response: logLRL
                   Df  Sum Sq Mean Sq F value    Pr(>F)    
Genotype            1   9.565   9.565 25.7929 5.654e-07 ***
Treatment           1  37.008  37.008 99.7986 < 2.2e-16 ***
Image              73  85.360   1.169  3.1533 1.262e-13 ***
Genotype:Treatment   1   0.070   0.070  0.1893    0.6637    
Residuals         434 160.939   0.371                      
---
Signif. codes:  0 ‘***’ 0.001 ‘**’ 0.01 ‘*’ 0.05 ‘.’ 0.1 ‘ ’ 1
logALRL ~ Genotype * Treatment + Image
<environment: 0x000002957087c9e0>
Analysis of Variance Table

Response: logALRL
                   Df  Sum Sq Mean Sq F value    Pr(>F)    
Genotype            1   4.446  4.4460 16.7093 5.192e-05 ***
Treatment           1  18.835 18.8355 70.7895 5.807e-16 ***
Image              73 107.195  1.4684  5.5188 < 2.2e-16 ***
Genotype:Treatment   1   0.009  0.0095  0.0356    0.8504    
Residuals         434 115.478  0.2661                      
---
Signif. codes:  0 ‘***’ 0.001 ‘**’ 0.01 ‘*’ 0.05 ‘.’ 0.1 ‘ ’ 1
logTRL ~ Genotype * Treatment + Image
<environment: 0x000002957087c9e0>
Analysis of Variance Table

Response: logTRL
                   Df  Sum Sq Mean Sq F value    Pr(>F)    
Genotype            1  0.7502 0.75021 26.5609 3.886e-07 ***
Treatment           1  0.2697 0.26967  9.5476  0.002131 ** 
Image              73 12.5735 0.17224  6.0981 < 2.2e-16 ***
Genotype:Treatment   1  0.0002 0.00017  0.0062  0.937432    
Residuals         434 12.2583 0.02824                      
---
Signif. codes:  0 ‘***’ 0.001 ‘**’ 0.01 ‘*’ 0.05 ‘.’ 0.1 ‘ ’ 1
logLRD ~ Genotype * Treatment + Image
<environment: 0x000002957087c9e0>
Analysis of Variance Table
```

```
Response: logLRD
                  Df  Sum Sq Mean Sq F value    Pr(>F)
Genotype           1  20.746 20.7462 60.1677 6.278e-14 ***
Treatment          1  26.011 26.0108 75.4360 < 2.2e-16 ***
Image             73 176.304  2.4151  7.0043 < 2.2e-16 ***
Genotype:Treatment   1   0.212  0.2116  0.6138    0.4338
Residuals        434 149.646  0.3448
---
Signif. codes:  0 ‘***’ 0.001 ‘**’ 0.01 ‘*’ 0.05 ‘.’ 0.1 ‘ ’ 1
logLRP ~ Genotype * Treatment + Image
<environment: 0x000002957087c9e0>
Analysis of Variance Table

Response: logLRP
                  Df  Sum Sq Mean Sq  F value    Pr(>F)
Genotype           1   4.957  4.9575  19.8339 1.076e-05 ***
Treatment          1  30.960 30.9596 123.8630 < 2.2e-16 ***
Image             73  82.200  1.1260   4.5050 < 2.2e-16 ***
Genotype:Treatment   1   0.077  0.0774   0.3096    0.5782
Residuals        434 108.478  0.2500
```

****

Tomato Data

LogPR
0mM

Arf18-9b-1
The t-value is 0.61231. The p-value is .546092. The result is not significant at $p < .05$.

Arf18-9b-2
The t-value is -0.41715. The p-value is .679273. The result is not significant at $p < .05$.

Nlp7-1
The t-value is 2.00315. The p-value is .058901. The result is not significant at $p < .05$.

Nlp7-2
The t-value is 1.64683. The p-value is .115217. The result is not significant at $p < .05$.

1mM Arf18-9b-1
The t-value is -0.57884. The p-value is .567327. The result is not significant at $p < .05$.

Nlp7-1

The t-value is 0.06521. The p-value is .948402. The result is not significant at p < .05.

Nlp7-2
The t-value is -0.3964. The p-value is .69422. The result is not significant at p < .05.

10mM Arf18-9b-1
The t-value is -0.21882. The p-value is .828809. The result is not significant at p < .05.

Arf18-9b-2
The t-value is -0.20423. The p-value is .839707. The result is not significant at p < .05.

Nlp7-1
The t-value is 0.39643. The p-value is .694908. The result is not significant at p < .05.

Nlp7-2
The t-value is 0.56488. The p-value is .576497. The result is not significant at p < .05.
LogLR 0mM Arf18-9b-1
The t-value is 0.85284. The p-value is .401844. The result is not significant at p < .05.

Arf18-9b-2
The t-value is 0.1612. The p-value is .87289. The result is not significant at p < .05.

Nlp7-1
The t-value is -0.78371. The p-value is .439564. The result is not significant at p < .05.

Nlp7-2
The t-value is 0.02031. The p-value is .983922. The result is not significant at p < .05.

1mM Arf18-9b-1
The t-value is 0.01174. The p-value is .990721. The result is not significant at p < .05. Arf18-9b-2
The t-value is 0.29096. The p-value is .773084. The result is not significant at p < .05. Nlp7-1
The t-value is 0.41708. The p-value is .679493. The result is not significant at p < .05. Nlp7-2
The t-value is 0.28014. The p-value is .781175. The result is not significant at p < .05.

10mM Arf18-9b-1
The t-value is -2.52796. The p-value is .020001. The result is significant at p < .05. Arf18-9b-2
The t-value is -0.707. The p-value is .486105. The result is not significant at p < .05. Nlp7-1
The t-value is -0.7709. The p-value is .447989. The result is not significant at p < .05. Nlp7-2
The t-value is 0.54191. The p-value is .592322. The result is not significant at p < .05.
LogLRL 0mM Arf18-9b-1
The t-value is 0.96786. The p-value is .342767. The result is not significant at p < .05. Arf18-9b-2
The t-value is -1.00858. The p-value is .320517. The result is not significant at p < .05. Nlp7-1

The t-value is -0.53523. The p-value is .596715. The result is not significant at p < .05. Nlp7-2
The t-value is -1.16495. The p-value is .252649. The result is not significant at p < .05.

1mM Arf18-9b-1
The t-value is 2.93724. The p-value is .006698. The result is significant at p < .05. Arf18-9b-2
The t-value is 1.18104. The p-value is .246866. The result is not significant at p < .05 Nlp7-1
The t-value is 1.30648. The p-value is .200999. The result is not significant at p < .05. Nlp7-2
The t-value is 1.96043. The p-value is .0587. The result is not significant at p < .05.

10mM Arf18-9b-1
The t-value is -0.02898. The p-value is .977154. The result is not significant at p < .05. Arf18-9b-2
The t-value is -0.18655. The p-value is .853464. The result is not significant at p < .05. Nlp7-1
The t-value is 1.30355. The p-value is .203812. The result is not significant at p < .05 Nlp7-2
The t-value is 0.87754. The p-value is .387663. The result is not significant at p < .05.
LogALRL
0mM Arf18-9b-1
The t-value is -0.1351. The p-value is .893662. The result is not significant at p < .05. Arf18-9b-2
The t-value is -1.30956. The p-value is .19939. The result is not significant at p < .05. Nlp7-1
The t-value is 0.25596. The p-value is .799852. The result is not significant at p < .05. Nlp7-2
The t-value is -1.44371. The p-value is .158539. The result is not significant at p < .05.

1mM Arf18-9b-1
The t-value is 3.97043. The p-value is .000479. The result is significant at p < .05. Arf18-9b-2
The t-value is 1.03755. The p-value is .307771. The result is not significant at p < .05. Nlp7-1
The t-value is 1.05941. The p-value is .297599. The result is not significant at p < .05. Nlp7-2
The t-value is 2.26187. The p-value is .030634. The result is significant at p < .05.

10mM Arf18-9b-1
The t-value is 3.74574. The p-value is .001191. The result is significant at p < .05. Arf18-9b-2
The t-value is 0.56733. The p-value is .575356. The result is not significant at p < .05. Nlp7-1
The t-value is 2.80398. The p-value is .00942. The result is significant at p < .05. Nlp7-2
The t-value is 0.49664. The p-value is .623323. The result is not significant at p < .05.
LogTRL 0mM Arf18-9b-1
The t-value is 0.98852. The p-value is .332759. The result is not significant at p < .05. Arf18-9b-2
The t-value is -1.2308. The p-value is .227099. The result is not significant at p < .05. Nlp7-1
The t-value is -2.11631. The p-value is .043022. The result is significant at p < .05. Nlp7-2
The t-value is -1.54. The p-value is .133097. The result is not significant at p < .05.

1mM Arf18-9b-1
The t-value is 2.20049. The p-value is .036187. The result is significant at p < .05. Arf18-9b-2
The t-value is -1.23322. The p-value is .225952. The result is not significant at p < .05. Nlp7-1

The t-value is 0.47944. The p-value is .634788. The result is not significant at p < .05. Nlp7-2
The t-value is 0.46245. The p-value is .646621. The result is not significant at p < .05.

10mM Arf18-9b-1
The t-value is 0.2305. The p-value is .819836. The result is not significant at p < .05. Arf18-9b-2
The t-value is 0.28743. The p-value is .77598. The result is not significant at p < .05. Nlp7-1
The t-value is 1.11468. The p-value is .274812. The result is not significant at p < .05. Nlp7-2
The t-value is 0.95943. The p-value is .345273. The result is not significant at p < .05.
LogLRD 0mM Arf18-9b-1
The t-value is 0.38967. The p-value is .700219. The result is not significant at p < .05. Arf18-9b-2
The t-value is 0.17567. The p-value is .861626. The result is not significant at p < .05. Nlp7-1
The t-value is 0.69905. The p-value is .490289. The result is not significant at p < .05. Nlp7-2
The t-value is 0.21295. The p-value is .832714. The result is not significant at p < .05.

1mM Arf18-9b-1
The t-value is 1.78373. The p-value is .086145. The result is not significant at p < .05. Arf18-9b-2
The t-value is 2.93212. The p-value is .006509. The result is significant at p < .05. Nlp7-1
The t-value is 2.12874. The p-value is .041601. The result is significant at p < .05 Nlp7-2
The t-value is 2.23989. The p-value is .032409. The result is significant at p < .05.

10mM Arf18-9b-1
The t-value is -1.50694. The p-value is .146722. The result is not significant at p < .05. Arf18-9b-2
The t-value is 0.01089. The p-value is .991397. The result is not significant at p < .05. Nlp7-1
The t-value is -0.72229. The p-value is .476561. The result is not significant at p < .05. Nlp7-2
The t-value is 0.19374. The p-value is .847777. The result is not significant at p < .05.
LogLRP
0mM Arf18-9b-1
The t-value is 0.65378. The p-value is .519469. The result is not significant at p < .05. Arf18-9b-2
The t-value is -0.50565. The p-value is .616462. The result is not significant at p < .05. Nlp7-1
The t-value is 0.74586. The p-value is .461969. The result is not significant at p < .05. Nlp7-2
The t-value is -0.77361. The p-value is .444841. The result is not significant at p < .05.

1mM Arf18-9b-1
The t-value is 3.20669. The p-value is .003441. The result is significant at p < .05. Arf18-9b-2
The t-value is 3.38466. The p-value is .002003. The result is significant at p < .05. Nlp7-1
The t-value is 1.69731. The p-value is .099657. The result is not significant at p < .05. Nlp7-2
The t-value is 2.18101. The p-value is .036645. The result is significant at p < .05.

10mM Arf18-9b-1
The t-value is 0.48567. The p-value is .632229. The result is not significant at p < .05. Arf18-9b-

2

The t-value is 0.36774. The p-value is .716043. The result is not significant at p < .05. Nlp7-1
The t-value is 1.06344. The p-value is .297361. The result is not significant at p < .05. Nlp7-2
The t-value is 0.89422. The p-value is .378827. The result is not significant at p < .05.

```
mod1 <- lm(LogPR~Genotype * Condition, contrasts=list(Genotype=contr.sum,
Condition=contr.sum), data=x)
Anova(mod1, type=3) Anova Table (Type III tests)

Response: LogPR
Sum Sq Df F value Pr(>F) (Intercept)          108.955 1 3357.6824 < 2e-16 ***
Genotype        0.147  4          1.1290 0.34465
Condition       0.072  2          1.1129 0.33105
Genotype:Condition  0.450  81.7325 0.09435 .
Residuals       5.354 165
---
Signif. codes: 0 ‘***’ 0.001 ‘**’ 0.01 ‘*’ 0.05 ‘.’ 0.1 ‘ ’ 1
mod1 <- lm(LogLR~Genotype * Condition, contrasts=list(Genotype=contr.sum,
Condition=contr.sum), data=x)
Anova(mod1, type=3) Anova Table (Type III tests)

Response: LogLR
Sum Sq Df F value Pr(>F) (Intercept)          118.708 1 2184.3471 < 2e-16 ***
Genotype        0.131  4          0.6030 0.66103
Condition       0.390  2          3.5859 0.03003 *
Genotype:Condition  0.399  80.9170 0.50411
Residuals       8.423 155
---
Signif. codes: 0 ‘***’ 0.001 ‘**’ 0.01 ‘*’ 0.05 ‘.’ 0.1 ‘ ’ 1
mod1 <- lm(LogLRL~Genotype * Condition, contrasts=list(Genotype=contr.sum,
Condition=contr.sum), data=x)
Anova(mod1, type=3) Anova Table (Type III tests)

Response: LogLRL
Sum Sq Df F value Pr(>F) (Intercept)          167.019 1 3717.0283 < 2e-16 ***
Genotype        0.236  4          1.3138 0.26725
Condition       0.531  2          5.9041 0.00338 **
Genotype:Condition  0.483  81.3437 0.22588
Residuals       6.965 155
---
Signif. codes: 0 ‘***’ 0.001 ‘**’ 0.01 ‘*’ 0.05 ‘.’ 0.1 ‘ ’ 1
mod1 <- lm(LogALRL~Genotype * Condition, contrasts=list(Genotype=contr.sum,
Condition=contr.sum), data=x)
Anova(mod1, type=3) Anova Table (Type III tests)
```

```
Response: LogALRL
Sum Sq Df F value Pr(>F) (Intercept)          5.7633  1 146.0570 < 2e-16 ***
Genotype        0.4842  4  3.0679 0.01822 *
Condition       0.1309  2  1.6582 0.19385
Genotype:Condition 0.4804  8  1.5217 0.15376
Residuals       6.1162 155
---
Signif. codes: 0 ‘***’ 0.001 ‘**’ 0.01 ‘*’ 0.05 ‘.’ 0.1 ‘ ’ 1
mod1 <- lm(LogTRL~Genotype * Condition, contrasts=list(Genotype=contr.sum,
Condition=contr.sum), data=x)
Anova(mod1, type=3) Anova Table (Type III tests)

Response: LogTRL
Sum Sq Df F value Pr(>F) (Intercept)          236.836 1 5004.3532 <2e-16 ***
Genotype        0.222  4         1.1740 0.3242
Condition       0.050  2         0.5326 0.5881
Genotype:Condition  0.545  81.4383 0.1841
Residuals       7.809 165
---
Signif. codes: 0 ‘***’ 0.001 ‘**’ 0.01 ‘*’ 0.05 ‘.’ 0.1 ‘ ’ 1
mod1 <- lm(LogLRD~Genotype * Condition, contrasts=list(Genotype=contr.sum,
Condition=contr.sum), data=x)
Anova(mod1, type=3) Anova Table (Type III tests)

Response: LogLRD
Sum Sq Df F value     Pr(>F) (Intercept)       0.0548 1 1.0562                  0.3057
Genotype        0.1060  4 0.5105        0.7281
Condition       1.0774  2 10.3751 5.906e-05 ***
Genotype:Condition 0.3169  8 0.7630          0.6358
Residuals       8.0479 155
---
Signif. codes: 0 ‘***’ 0.001 ‘**’ 0.01 ‘*’ 0.05 ‘.’ 0.1 ‘ ’ 1
mod1 <- lm(LogLRP~Genotype * Condition, contrasts=list(Genotype=contr.sum,
Condition=contr.sum), data=x)
Anova(mod1, type=3) Anova Table (Type III tests)

Response: LogLRP
Sum Sq Df F value     Pr(>F) (Intercept)       6.1749 1 695.6815 < 2.2e-16 ***
Genotype        0.0594  4  1.6732 0.158947
Condition       0.0982  2  5.5326 0.004776 **
Genotype:Condition 0.0899  8  1.2655 0.265395
Residuals       1.3758 155
---
Signif. codes: 0 ‘***’ 0.001 ‘**’ 0.01 ‘*’ 0.05 ‘.’ 0.1 ‘ ’ 1
```
