# Supplementary material for: Conservation and divergence of regulatory architecture in nitrate-responsive plant gene circuits
Source: Plant Cell. 2025 May 22;37(6):koaf124. doi: 10.1093/plcell/koaf124 (PMC12205479; doi:10.1093/plcell/koaf124)
Supplement: koaf124_Supplementary_Data [file koaf124_supplementary_data.zip › Supplementary Files S2.pdf]

```
 2  86
AtNLP6    EAKTVKKSERKRGKTEKTISLEVLQQYFAGSLKDAAKSLGVCPTTMKRIC
AtNLP7    ----KKKTEKKRGKTEKTISLDVLQQYFTGSLKDAAKSLGVCPTTMKRIC

RQHGISRWPSRKINKVNRSLTRLKHVIDSVQGADGS
RQHGISRWPSRKIKKVNRSITKLKRVIESVQGTDGG
```

```
 3  103
AtARF9    FSKVLTASDTSTHGGFSVLRKHATECLPPLDMTQQTPTQELVAEDVHGYQ
AtARF18   FVKILTASDTSTHGGFSVLRKHATECLPSLDMTQATPTQELVTRDLHGFE
AtARF2    FCKTLTASDTSTHGGFSVLRRHADECLPPLDMSRQPPTQELVAKDLHANE

WKFKHIFRGQPRRHLLTTGWSTFVTSKRLVAGDTFVFLRGENGELRVGVR
WRFKHIFRGQPRRHLLTTGWSTFVSSKRLVAGDAFVFLRGENGDLRVGVR
WRFRHIFRGQPRRHLLQSGWSVFVSSKRLVAGDAFIFLRGENGELRVGVR

RAN
RLA
RAM
```

```
 2  152
AtANAC032 FPPGFRFHPTDEELVLMYLCRKCASQPIPAPIITELDLYRYDPWDLPDMA
ATAF1     LPPGFRFHPTDEELVMHYLCRKCASQSIAVPIIAEIDLYKYDPWELPGLA

LYGEKEWYFFSPRDRKYPNGSRPNRAAGTGYWKATGADKPIGRPKPVGIK
LYGEKEWYFFSPRDRKYPNGSRPNRSAGSGYWKATGADKPIGLPKPVGIK

KALVFYSGKPPNGEKTNWIMHEYRLADVDRSVRK-KNSLRLDDWVLCRIY
KALVFYAGKAPKGEKTNWIMHEYRLADVDRSVRKKKNSLRLDDWVLCRIY

NK
NK
```
