# Supplementary material for: Conservation and divergence of regulatory architecture in nitrate-responsive plant gene circuits
Source: Plant Cell. 2025 May 22;37(6):koaf124. doi: 10.1093/plcell/koaf124 (PMC12205479; doi:10.1093/plcell/koaf124)
Supplement: koaf124_Supplementary_Data [file koaf124_supplementary_data.zip › Supplementary Files S4.pdf]

```
 5  103
AtARF2    FCKTLTASDTSTHGGFSVLRRHADECLPPLDMSRQPPTQELVAKDLHANE
SlARF9B   FCKVLTASDTSTHGGFSILRKHANECLPPLDMTQATPAQELVAKDLHGFE
AtARF9    FSKVLTASDTSTHGGFSVLRKHATECLPPLDMTQQTPTQELVAEDVHGYQ
AtARF18   FVKILTASDTSTHGGFSVLRKHATECLPSLDMTQATPTQELVTRDLHGFE
SlARF18   FCKILTASDTSTHGGFSVLRKHANECLPQLDMTQATPTQDLVAKDLHGYE

WRFRHIFRGQPRRHLLQSGWSVFVSSKRLVAGDAFIFLRGENGELRVGVR
WHFKHIFRGQPRRHLLTTGWSTFVSSKRLVTGDSFVFLRSGKGEVRIGIR
WKFKHIFRGQPRRHLLTTGWSTFVTSKRLVAGDTFVFLRGENGELRVGVR
WRFKHIFRGQPRRHLLTTGWSTFVSSKRLVAGDAFVFLRGENGDLRVGVR
WRFKHIFRGQPRRHLLTTGWSTFVTSKRLVAGDAFVFLRDDSGELRVGVR

RAM
RLA
RAN
RLA
RLA
```

2 60

```
AtDREB26  KYKGVRMRSWGSWVSEIRAPNQKTRIWLGSYSTAEAAARAYDVALLCLKG
SlDREB26  KYKGVRMRSWGSWVSEIRAPNQKTRIWLGSYSTPEAAARAYDAALLCLKG

PQAN--LNFP
PSASSNLNFP
```

```
 4  86
AtNLP7    ----KKKTEKKRGKTEKTISLDVLQQYFTGSLKDAAKSLGVCPTTMKRIC
AtNLP6    EAKTVKKSERKRGKTEKTISLEVLQQYFAGSLKDAAKSLGVCPTTMKRIC
SlNLP7a   ---TGKKSERKRGKAEKTISLEVLQQYFAGSLKDAAKSLGVCPTTMKRIC
SlNLP7b   --TSGKKSERKRGKAEKTISLEVLQQYFAGSLKDAAKSLGVCPTTMKRIC

RQHGISRWPSRKIKKVNRSITKLKRVIESVQGTDGG
RQHGISRWPSRKINKVNRSLTRLKHVIDSVQGADGS
RQHGISRWPSRKINKVNRSLSKLKRVIESVQGADGT
RQHGISRWPSRKINKVNRSLSKLKCVIESVQGAEGA
```
